# Predictable C–H Functionalization of Complex *beta*-Fused Azines: A Mechanistically Bound Site-Specific Oxidation

**DOI:** 10.1021/acscentsci.5c00797

**Published:** 2025-07-01

**Authors:** Carla Obradors, Christopher A. Reiher, Cristina Grosanu, Mikko Muuronen, Romain Tessier, Egor M. Larin, Valentin Lehuédé

**Affiliations:** † Chemical Process Research and Development at Johnson & Johnson Innovative Medicine, 50148 Janssen Research and Development, A Division of Janssen Pharmaceutica NV, Turnhoutseweg 30, 2340 Beerse, Belgium; ‡ Chemistry Capabilities, Analytical and Purification at Johnson & Johnson Innovative Medicine, 6808 Janssen Research and Development LLC, 1400 McKean Road, Spring House, Pennsylvania 19477, United States; § Analytical Development Synthetics at Johnson & Johnson Innovative Medicine, Janssen Research and Development, A Division of Janssen Pharmaceutica NV, Turnhoutseweg 30, 2340 Beerse, Belgium; ∥ Global Discovery Chemistry at Johnson & Johnson Innovative Medicine, Janssen Research and Development, A Division of Janssen Pharmaceutica NV, Turnhoutseweg 30, 2340 Beerse, Belgium

## Abstract

Direct manipulation of C–H bonds enclosed in complex scaffolds persists today as an elusive disconnection when aiming for high and predictable site-selectivity. Its development toward the late-stage diversification of heterocycles remains of the upmost interest due to their ubiquitous presence in synthetic drugs and new methods consistently emerge to facilitate more versatile routes. The underlying challenge of activating a single C–H bond often leads to isomeric mixtures and a limited scope, which gets magnified in polycyclic frameworks, and the biased selectivity depending on the ring decoration recurrently hampers reliable retrosynthetic analyses. Here we report the straightforward C–H functionalization of multiple *beta*-fused azines toward a C–O bond formation with exclusive as well as predictable regiocontrol. Mild conditions enable the presence of a vast variety of motifs with orthogonal reactivity to transition-metals and highly sensitive moieties while also adding a divergent synthetic handle for further derivatizations in >10 distinct heterocyclic scaffolds.

## Graphical abstract

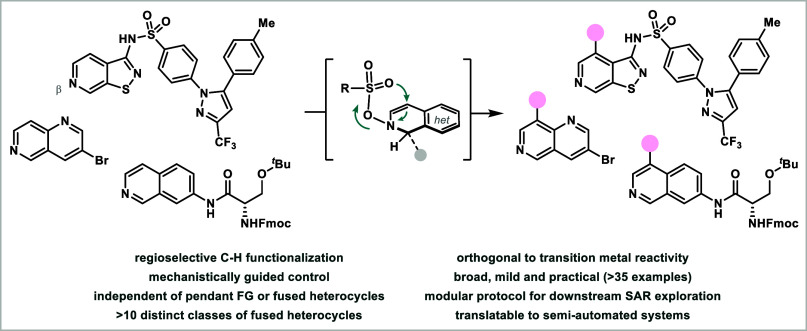

## Introduction

Drug discovery has long relied on the intricate assembly of heterocycles.^1–3^ Engineered ring condensations along with derivatization of prefunctionalized building blocks constitute the most robust approaches for the preparation of *N*-based scaffolds.^4^ Nevertheless, constant development of novel methodologies is crucial to keep expanding the synthetic chemist’s toolkit toward a rapid diversification to new chemical space.^5–9^ In the case of azines, a large variety of late-stage functionalization protocols has gradually arisen to unlock the direct manipulation of C–H bonds (Figure 1a); capitalizing on electrophilic substitutions,^10–24^ radical transformations,^25–28^ and transition-metal mediated C–H activation/deprotonation processes.^29–41^ In spite of the tremendous progress, these often present recurrent pitfalls such as harsh conditions leading to a restricted scope, the necessity of directing groups, or limited selectivity. In parallel, preactivation strategies have also emerged to overcome some of these challenges.^42–51^ An additional level of complexity that has usually been relegated to complementary examples of these methodologies, however, is the *direct and efficient functionalization of polycyclic structures*. Fused heterocycles are unequivocally cardinal pharmacophores in a vast array of biologically active products with further intrinsic synthetic hurdles.^4–9^ Notably, complete regiocontrol is particularly challenging usually requiring either electronic or steric biases, plus a shift of selectivity can occur depending on each specific scaffold and/or the functional groups decorating the rings.

**Figure 1:**
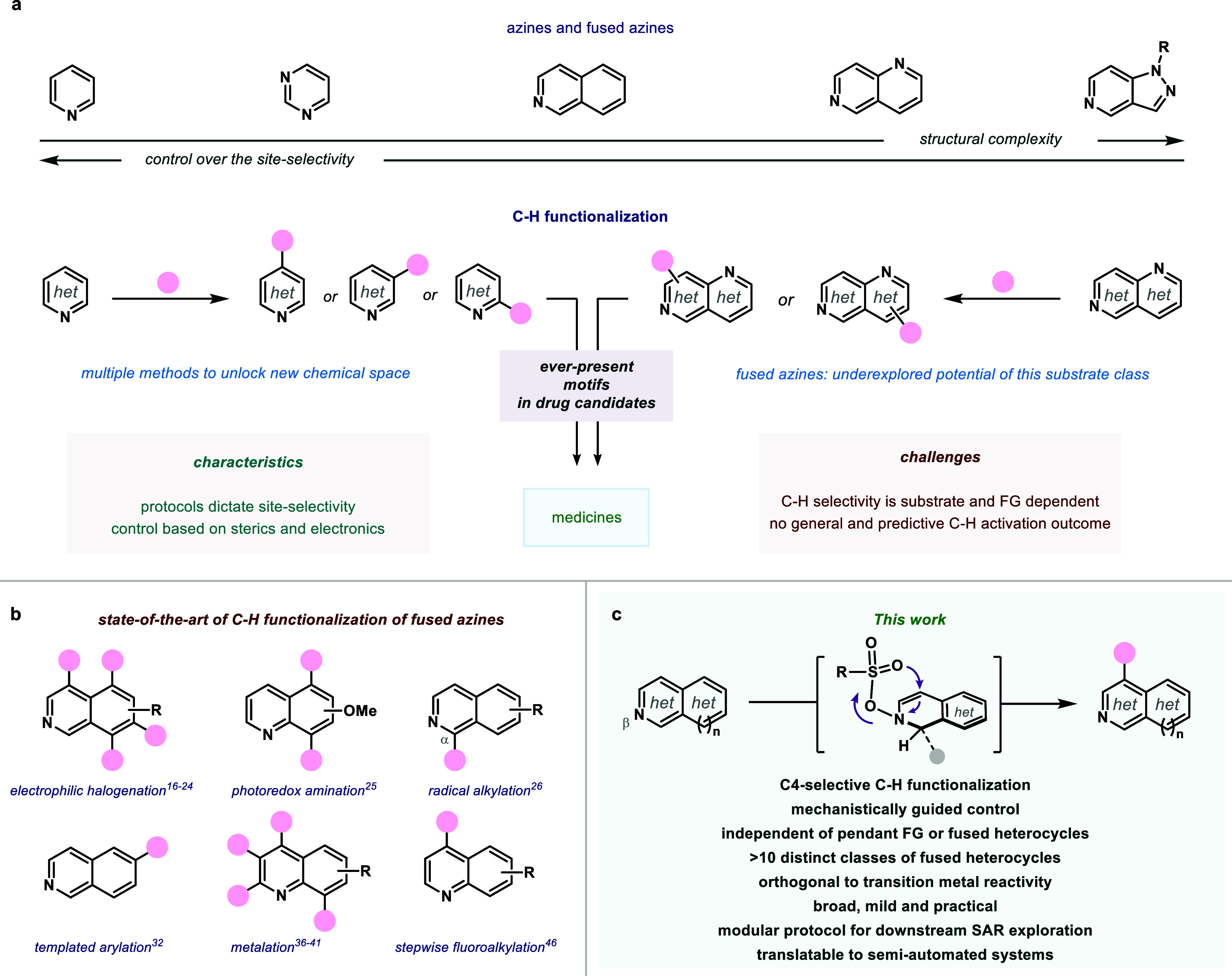
State-of-the-art of C–H functionalization of azines. (a) Abundance in synthetic drugs and reactivity challenges when increasing structural complexity. (b) Specific examples of fused analogs. (c) *This work*: straightforward site-specific functionalization of multiple *beta*-fused azines with predictive regiocontrol.

For instance, one of the most common strategies utilized is electrophilic halogenation (Figure 1b).^16–24^ Regioselectivity of this approach is strictly dictated by the electronics of the aromatic ring, changing with the substitution pattern, thus hampering its use as a reliable and selective late-stage retrosynthetic disconnection. Additionally, harsh conditions are often required for these electron deficient scaffolds that can lead to polyfunctionalization of the substrate, as in the isoquinoline pictured. These challenges are a general burden for direct C–H functionalization of multiple azines.^10–15^ Namely, C–H amination enabled by photoredox catalysis required electron rich substrates, *i.e*., MeO-groups on the quinoline scaffold, where the regioselectivity is ring and substituent dependent.^25^ Similarly, predictability of radical additions is complex for many substrates.^26^ Regioselectivity tends to be favored in the α-position in the case of *beta*-fused azines, usually consuming superstoichiometric amounts of the coupling partner. In contrast, design of a directing template did enable distal C–H arylation with exclusive regioselectivity in isoquinolines.^32^ This method provided novel regiocontrol to the carbocyclic ring by using stoichiometric Pd. In analogy, Li/Mg/Zn-metalation of heterocycles facilitates a wide diversification of numerous scaffolds.^36–41^ In this case, regioselectivity is also determined by the electronic bias of the substrate to favor deprotonation. Finally, effective preactivation strategies usually require superstoichiometric amounts of reagents as well generating significant waste, for example, in the phosphorus-mediated fluoroalkylation of azines.^46^ These precedents manifest the challenges in this area and the significant gap in providing general C–H functionalization strategies with a predictable selectivity independent of the heterocycle.

We disclose here the development of a *broad, facile, and regiospecific C–H functionalization of distinct beta-fused azines* that capitalizes on the intrinsic reactivity of the heterocyclic moiety (Figure 1c). The functionalization is characterized by the efficient and exclusive installation of sulfonates and chlorides at C4 that enable further derivatizations. The mild conditions permit its application to a wide chemical space, including densely functionalized drug-like scaffolds with sensitive functional groups and also to transition metals. It is worth pointing out that the protocol provides access to C–O bonds from the parent C–H, which has been a particularly demanding goal in the literature especially for electron poor rings.^52–56^ Currently, the preparation of C4-oxidized *beta*-fused analogs involves multistep sequences initiated by deprotonation or electrophilic halogenation, wherein the regioselectivity is dictated by the substrate. In contrast, the reaction reported herein proceeds via a transient *N*-oxide that governs the regiocontrol of the transformation by completely migrating to C4. Thus, the method enables a predictable regioselectivity over >10 distinct heterocyclic scaffolds with diverse FG decoration through a mechanistically bound site-specificity, leading to a rare functionalization of *beta*-fused rings that has remained largely underexplored. Straightforward access to a versatile handle in such an elusive position streamlines targeted structure–activity relationship (SAR) explorations across multiple complex heterocycles. Furthermore, the method can be successfully transferred to an semi-automated platform for compound library synthesis. This capability is nowadays an essential feature in drug discovery to accelerate the medicinal chemistry’s design-test cycle; these ultimately enable the rapid generation of the key data to evolve a hit into a drug.^57–61^

## Results and Discussion

### Reaction Development

Typical conditions to functionalize isoquinolines at position 1 toward C–X bonds rely on the preparation of the *N*-oxide to facilitate nucleophilic addition and subsequent rearomatization of the azine (Figure 2a).^62^ During the derivatization of substrate **1a** with TsCl, we observed that assembling compound **2** competes with heterocycle tosylation. Reaction in the presence of a base and a nucleophile such as *tert*-butyl amine, saccharine or *para*-hydroxyanisole undergoes both the expected nucleophilic addition in position 1 along with tosyl addition toward an isomeric mixture. Particularly, this competition becomes more pronounced when decreasing the nucleophilicity of NuH, leading to traces of bromination when tetrabutylammonium bromide (TBABr) is used along with hydration and dimerization. Thus, reaction of the *N*-oxide with TsCl forms the ion pair **Int I** where the nucleophiles add to C1 leading to **Int II** and product **2** after deprotonation (Figure 2b). However, instead of compound **2**, ^1^H NMR analysis suggested the formation of another regioisomer as the major product of tosyl addition. Indeed, *in the absence of a nucleophile and base, a distinct selectivity to C4 is obtained*, affording isoquinoline **3a** as a single isomer. We envisioned that this unusual disconnection could be a straightforward solution to a long-standing challenge in the direct C–H functionalization of complex polyaromatic scaffolds. Albeit the transformation is rather slow when utilizing TsCl, we were able to optimize the formation of **3a** in excellent yield using Ts_2_O instead. Moreover, the reaction proceeds at multigram scale without significant erosion of its efficiency.

**Figure 2:**
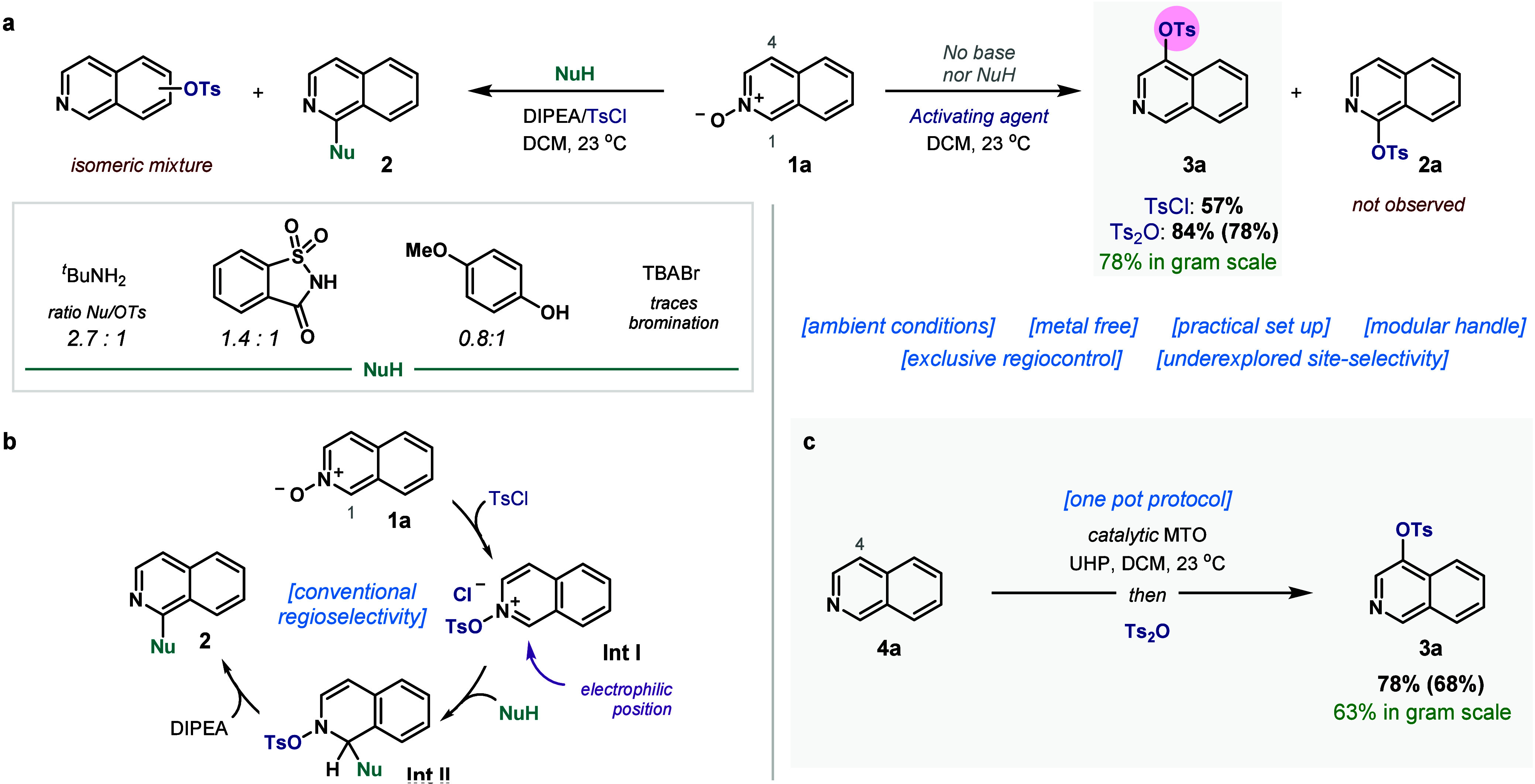
Reaction development. (a) Nucleophilic addition to *N*-oxides and unusual TsO-regioselectivity. (b) Mechanistic hypothesis of conventional reactivity. (c) Direct transformation to C4 from the azine via mild oxidation *in situ*. Ratios measured by UPLC-UV. Yields measured by quantitative NMR, isolated yields in parentheses or in green.

To overcome the limited commercial availability of *N*-oxides, we reasoned that it would be key to develop a protocol that was *practical and generated the N-oxide in situ from any fused azine*, while remaining compatible with the subsequent activation/addition steps (Figure 2c). Complete oxidation proceeds under mild conditions with urea-hydrogen peroxide (UHP) and 5 mol % of methyltrioxorhenium (MTO).^63,64^ Then, the immediate addition of Ts_2_O leads to the formation of isoquinoline **3a** in 78% yield. This design effectively enables a simple one pot approach toward the straightforward and facile C–H functionalization of a *beta*-fused azine such as **4a** with complete regiocontrol to C4. The one-pot procedure is similarly scalable.

### Semi-Automated Scope Evaluation

We next examined the generality of the transformation by leveraging our high throughput experimentation (HTE) and purification (HTP) platforms.^65^ In drug discovery, these technologies use automation and miniaturization to enable rapid data generation from large compound libraries in parallel (10–10^3^ substrates).^57–61^ Here, we first identified readily available azine-containing fused building blocks, clustered them computationally by structural similarity, and then selected substrates to cover a wide range of medicinal chemistry relevant scaffolds and diverse functional groups (96- compounds library, 0.1 mmol scale). UPLC-MS analysis upon reaction completion demonstrated that a varied subset successfully underwent single tosylation, as depicted in Figure 3, as well as the feasibility of executing the protocol in a parallel mode (over 30% success; see SI for complete details). Besides multiple substitution patterns, the transformation tolerates esters, ketones, carboxylic acids, alkyl, alkoxy, polyfluoromethyl, bromide, chloride, nitro, and nitrile moieties as well as amine and hydroxyl groups. Notably, the reactivity observed for isoquinolines can efficiently be extended to multiple *beta*-fused *N*-heterocyclic scaffolds including naphthyridines and pyrido-isoxazoles, -thiophenes, -thiazoles, -isothiazoles, -triazoles, and -pyrazoles. In contrast, quinolines, quinazolines, monocyclic pyridines and 4-susbtituted isoquinolines do not undergo tosylation under these conditions, which establishes orthogonality between heterocycles. In addition, compounds with readily oxidizable functionalities—for example, aldehydes or boronic esters—are not tolerated. More importantly, subsequent HTP enabled the confirmation of a *predictable regioselectivity regardless of the scaffold used* (>95% purity).

**Figure 3:**
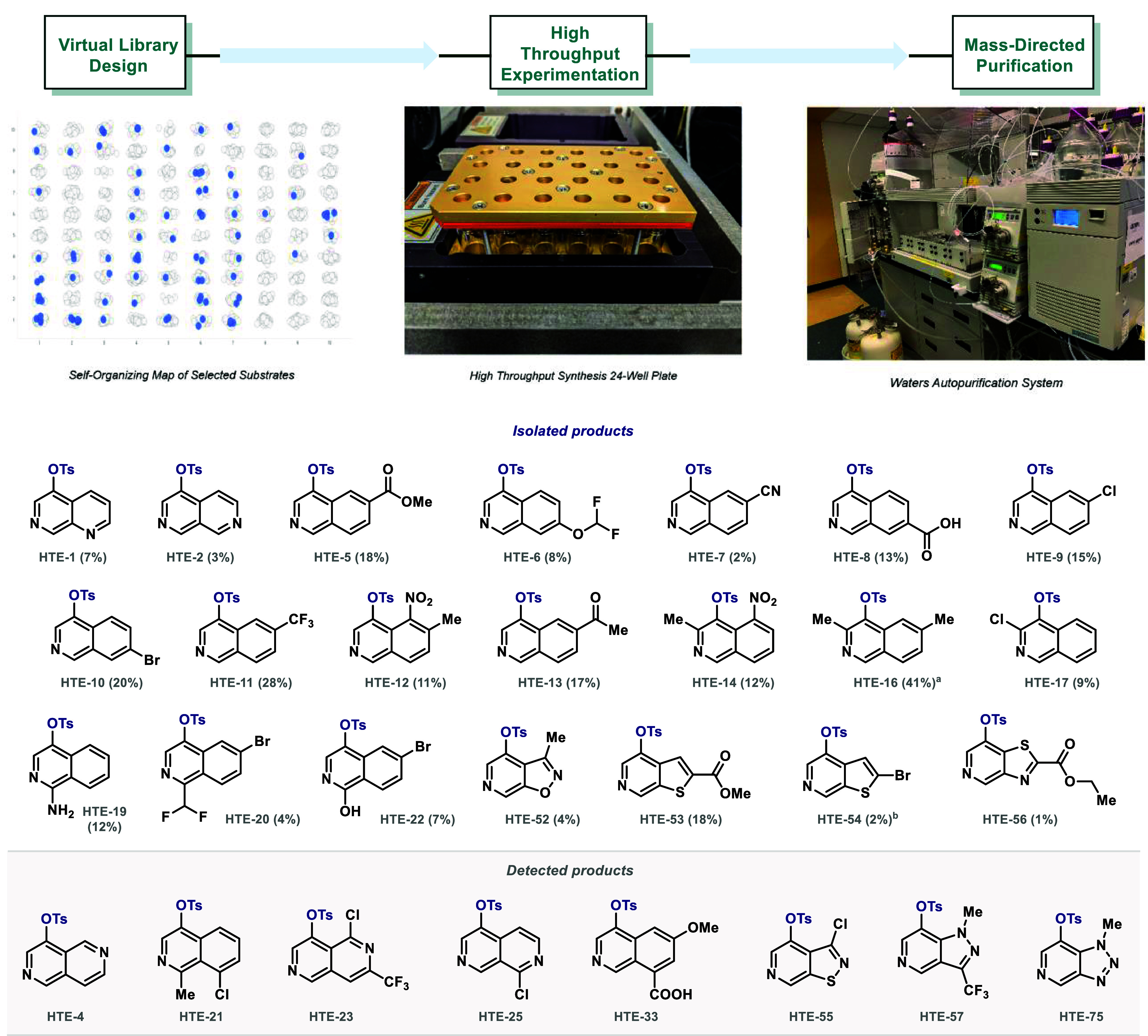
Assessment of the generality of the transformation, selected examples from the HTE evaluation (over 30% success). Compounds detected by UPLC-MS were isolated by reverse phase HTP to >95% purity for characterization. ^a^94% purity. ^b^83% purity.

### Reaction Scope

Consecutively, we contrasted these exploratory results to bench scale synthesis further assessing the procedure’s potential (Figure 4). Under the optimized conditions, the purification of the *N*-containing products endured as the main challenge. Nevertheless, isoquinoline **3b** bearing a nitrile (HTE-7) is obtained in 70% isolated yield. Investigation of the peripheral functional groups led analogously to distinct decorations in good yields (**3c**, **3e**, **3f**, **3g**, **3i**, and **3j**), also incorporating fluoride groups and 8-substitution. Albeit with lower yield, the presence of electron-donating groups is also tolerated (**3d**). Moreover, the reaction allows fine-tuning of the conditions to increase the yield, such as higher temperatures during the oxidation step, as exemplified by the electron poor substrate **3h** (see SI for details). Notably, reaction with 1-methylisoquinoline builds compound **3** as a minor product, instead favoring the Boekelheide transformation (**5**). Functionalization of more complex azines bearing multiple heteroatoms highlights further the prospects of the method. Preparation of naphthyridines **3k** and **3l** with bromide and chloride handles, respectively, validates the orthogonality of the protocol to transition-metal catalyzed reactions. In addition, this leads to polyfunctionalized scaffolds set for concurrent SAR diversifications. Similarly, as detected in the HTE, highly decorated [6,5]-fused heterocycles build the analogous products also with exclusive regiocontrol. Thus, substrates **3m**, **3n**, **3o**, and **3p** could be efficiently obtained and isolated here.

**Figure 4:**
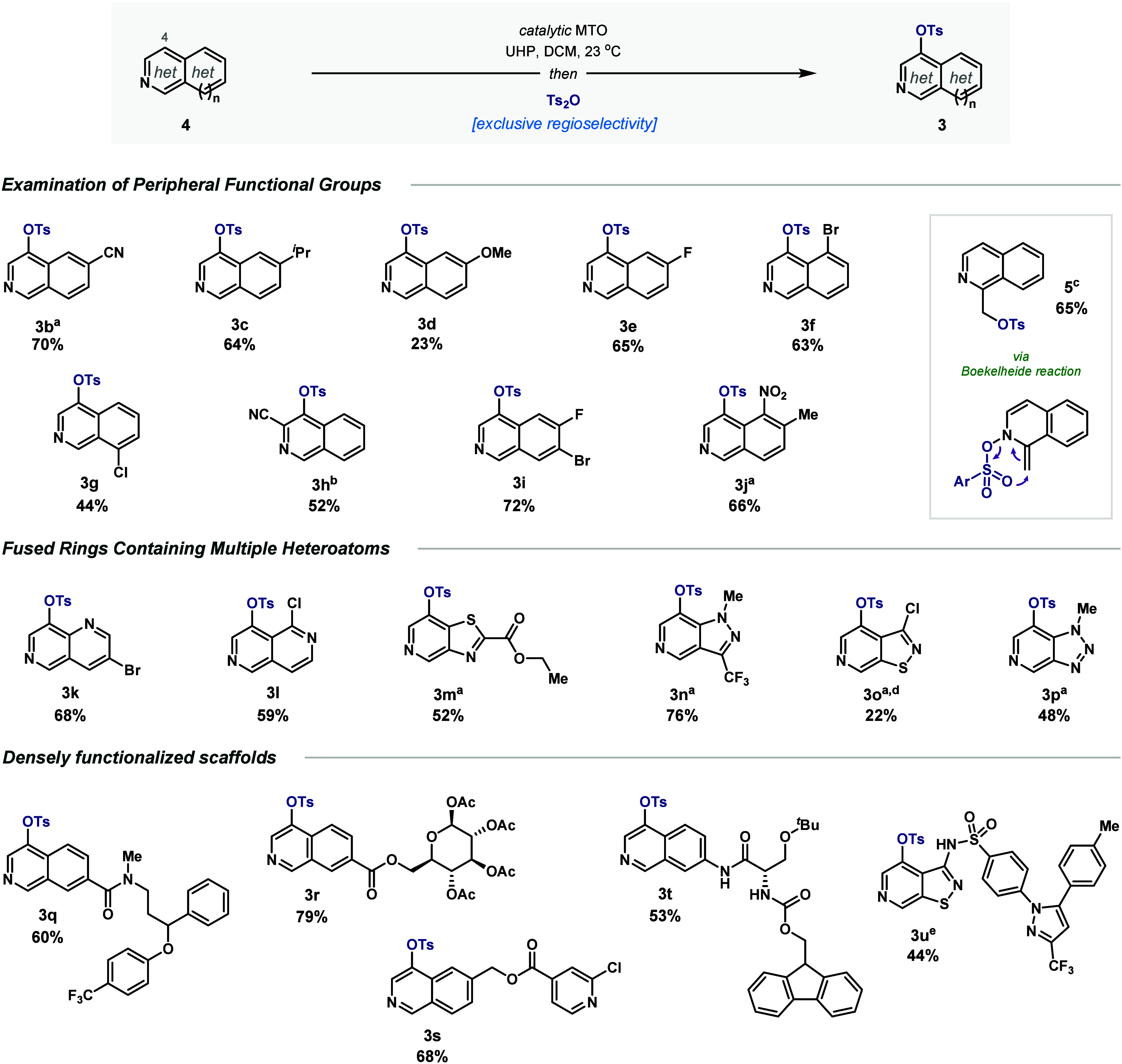
Site-specific functionalization of a wide variety of *N*-heterocycles (isolated yields). ^a^Cross-checked examples from HTE. ^b^Oxidation under reflux. ^c^Compound **3** observed as byproduct (19% LCAP). ^d^Slightly unstable on silica gel. ^e^Oxidation with *meta-*chloroperbenzoic acid.

Tosylation of several densely functionalized compounds was also carried out, demonstrating its potential toward additionally complex drug-like scaffold*s*. A fluoxetine derivative underwent C–H functionalization to forge compound **3q**, which introduced amides to the scope. Beyond discrimination from position 1, the reaction solely occurs on the heterocyclic ring, in contrast to alternative methods based on steric biases. The mild conditions of the protocol also permit the presence of highly sensitive moieties such as acetals, carbamates or epimerizable stereocenters as exemplified by the excellent yields for both glucoside and amino acid scaffolds **3r** and **3t**, respectively. The transformation shows orthogonal reactivity with monocyclic azines, such as pyridine, enabling the simultaneous presence of several *N*-heterocycles (**3s**). Finally, the more complex pyrido-isothiazole-sulfonamide of Celecoxib leads exclusively to C4 tosylation after major oxidation of the *beta*-fused azine with *meta*-chloroperbenzoic acid (**3u**). Beyond the various heterocyclic scaffolds, other activating agents were also interrogated (Figure 5a). Alkyl anhydrides such as Ms_2_O leads also to the corresponding product **3v**. In parallel to TsCl, other arylsulfonyl chlorides consistently react with the azine, as shown by the *para*-nosyl group in **3w**. No C–H functionalization was observed with Tf_2_O, Ac_2_O, bis­(trimethylsilyl) sulfate, tris­(trimethylsilyl) phosphate, or a sulfonyl fluoride. Finally, chlorination also occurs when using SO_2_Cl_2_ forming a mixture of **3x** and **2**.

**Figure 5:**
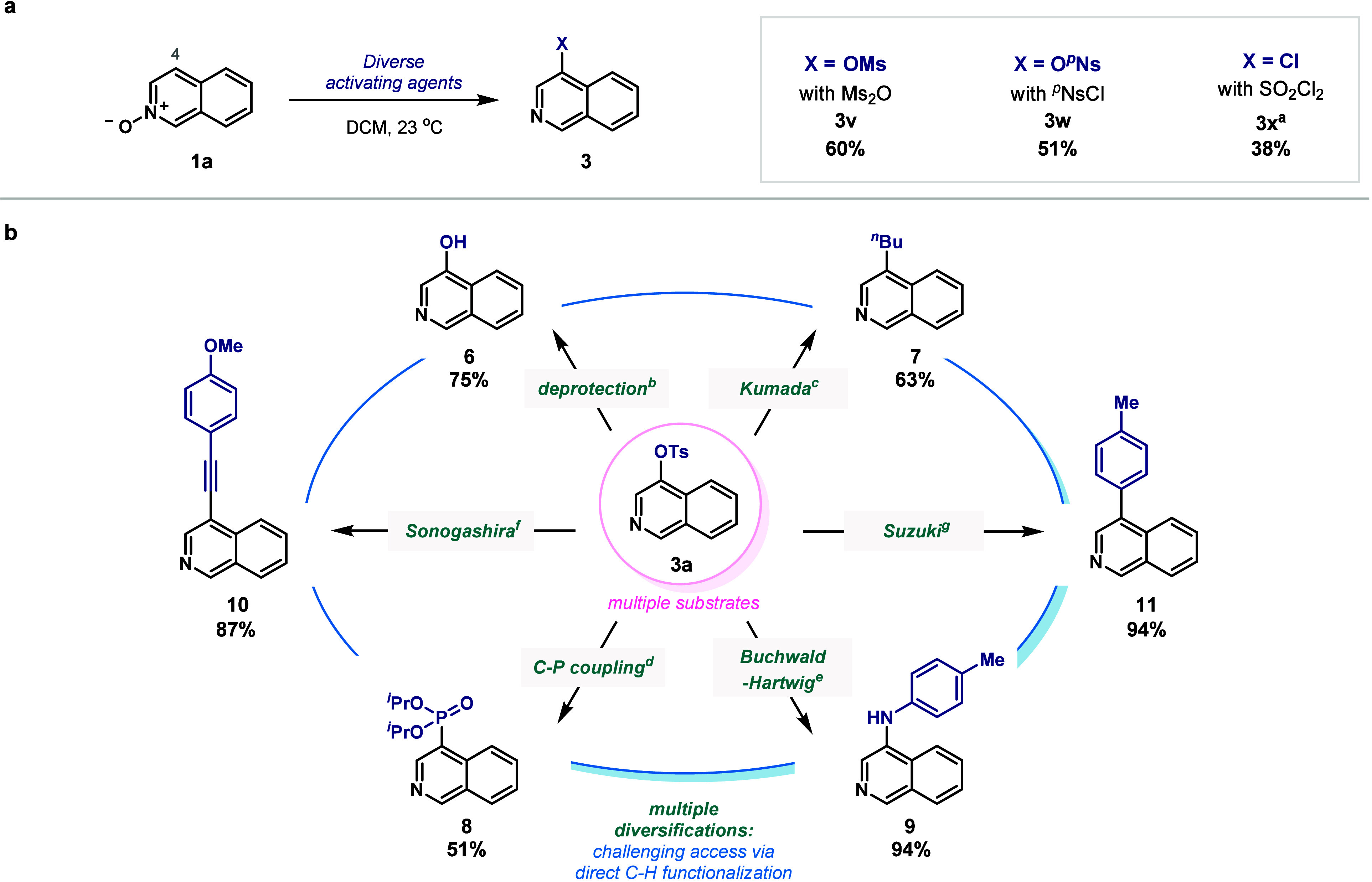
Access to product derivatives. (a) Alternative functionalization via migration of other activating groups. (b) Utility of tosylate as a synthetic handle. ^a^Compound **2** was observed as the main product. ^b^Reaction conditions for the deprotection of **3a**: KOH (5 equiv) and ^*t*^BuOH (10 equiv) in PhMe at 100 °C. ^c^For the Kumada coupling: butylmagnesium chloride (2 equiv), 10 mol % (dme)­NiCl_2_ and 20 mol % PPh_3_ in 1,4-dioxane at 25 °C. ^d^For the C–P coupling: diisopropyl phosphonate (3 equiv), 10 mol % *rac*-BINAP Pd G4 and K_2_HPO_4_ (3equiv) in DMA at 120 °C. ^e^For the Buchwald–Hartwig coupling: *p*-toluidine (2 equiv), 10 mol % GPhos Pd G4 and Cs_2_CO_4_ (3 equiv) in 4:1 DMA/H_2_O at 90 °C. ^f^For the Sonogashira coupling: 1-ethynyl-4-methoxybenzene (2 equiv), 10 mol % XPhos Pd G4 and K_3_PO_4_ (3 eqiuv) in ^*t*^BuOH at 90 °C. ^g^For the Suzuki coupling: *p*-tolyl boronic acid (2 equiv), 10 mol % PEPPSI-IPr and K_3_PO_4_ (3 equiv) in 4:1 1,4-dioxane/H_2_O at 90 °C.

### Further Derivatizations

The C–OTs functionality presents many opportunities as a derivatization handle for unique molecular construction.^66–71^ Besides deprotection to the hydroxylated product **6**, multiple cross-coupling reactions that unlock diverse SAR explorations are accessible leading to compounds that would be difficult to obtain from simple isoquinoline (Figure 5b). For example, nickel-catalyzed Kumada builds compound **7** via C­(*sp* ^2^)–C­(*sp* ^3^) formation. Palladium catalysis enables forging C–X bonds, such as in phosphonates (**8**) as well as to anilines via a Buchwald–Hartwig reaction (**9**). Finally, we also prepared C­(*sp* ^2^)–C­(*sp*) and C­(*sp* ^2^)–C­(*sp* ^2^) analogs in excellent yields via Sonogashira and Suzuki couplings, products **10** and **11** respectively.

### Mechanistic Insights

Finally, we conducted a kinetic analysis of the transformation via UPLC-UV that revealed that the reaction with the *N*-oxide generated *in situ* was faster than its isolated form (Figure 6a). Reaction of **1a** and Ts_2_O led to 70% of **3a** in 1 h, whereas TsCl consistently reached 40% (bold blue and gray lines, respectively). In contrast, *in situ* generation of the *N*-oxide from **4a** with MTO/UHP increased the yield to 85% within 15 min but rapidly evolved to **12** (yellow line), presumably via overoxidation pathways. Such degradation could be prevented by using an excess of Ts_2_O (bold green line), which corresponds to the optimized conditions of the methodology. We reasoned the main difference between both procedures relies on the presence of H_2_O. Indeed, addition of 3 equiv of H_2_O to **1a** remarkably increased the yield to 80% within 30 min with no degradation (red line). We hypothesize this behavior is due to a phase transfer effect between DCM and H_2_O.^72,73^ Moreover, we also examined a potential kinetic isotope effect via an intermolecular competition experiment (Figure 6b). Reaction up to 54% conversion of a 1:1 mixture of **4a** and *d*_***7***_ **-4a** under standard conditions afforded a mixture of **3a** and *d*_***6***_ **-3a** with 48% of D incorporation, suggesting no significant KIE and thus a fast C–H bond cleavage.^74^

**Figure 6:**
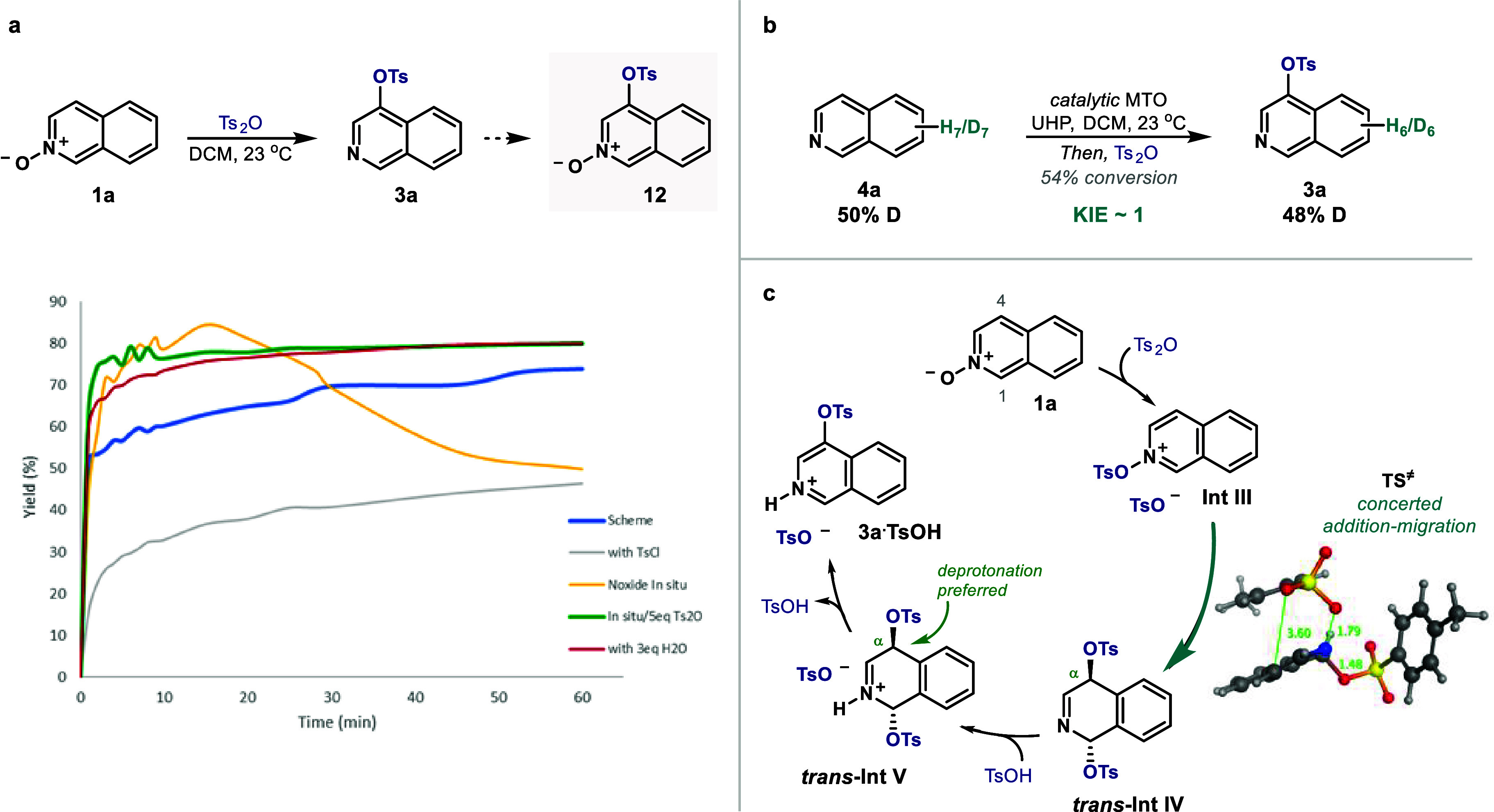
Mechanistic experiments. (a) Monitoring of the reaction under the conditions pictured in the scheme (bold blue line), reaction using TsCl instead of Ts_2_O (gray), reaction with the *N*-oxide generated *in situ* using MTO/UPH (yellow), reaction with the *N*-oxide generated *in situ* and with an excess of Ts_2_O (bold green), and reaction as pictured but in the presence of 3 equiv of H_2_O (red). (b) Intermolecular KIE experiment. (c) Hypothesis supported by NMR analysis and DFT calculations.

Combined with previous studies,^75–80^ these experiments led us to postulate the mechanistic scenario depicted in Figure 6c. Initially, the *N*-oxide reacts with Ts_2_O forming the ion pair **Int III**. ^1^H NMR monitoring revealed an intermediate with a downfield shift of H1 in **1a**, comparable to its protonation with TsOH (see SI for details). At this point, the TsO- attached to the nitrogen undergoes an unusual migration to C4 upon nucleophilic attack of the TsO^–^ counterion to position 1. We performed DFT calculations to understand this step further, which suggested that the nucleophilic attack and the migration forming *trans* **-Int IV** occur here in a concerted fashion (TS‡). Dynamic reaction coordinate calculations revealed that simple addition of TsO^–^ at C1 did not lead to a stable intermediate but triggers the elongation of the N–O bond while approaching the sulfonate to C4 in a [3,3]-sigmatropic rearrangement. Analysis of TS‡’s bond lengths shows these are formed/broken asynchronously (O–C1 1.48 Å, N–O 1.79 Å, and O–C4 3.6 Å). Subsequently, rearomatization of the heterocycle is achieved via a preferred deprotonation of the α-position of the iminium in *trans* **-Int V** (deprotonation from *trans* **-Int IV** cannot be excluded), which results in the regioselectivity observed in **3a**. Overall, intramolecular migration of the activating group in the fused azine instills a mechanistically bound regiocontrol and, therefore, the predictable C–H functionalization. Favored deprotonation of H4 in the dearomatized intermediate—being at the α-position of an iminium—translates to the exclusive C4 regiospecificity observed.

## Conclusions

In summary, we have developed a novel method for the straightforward C–H functionalization of multiple *beta*-fused azines transpiring with exclusive site-selectivity. Once synthetically elusive, this reaction now enables consistently the introduction of sulfonate or chloride handles at C4, unlocking multiple diversifications toward SAR exploration. The transformation proceeds under mild conditions tolerating a wide variety of functional groups including those sensitive to transition-metals and with drug-like complex scaffolds. Moreover, the method efficiently translates in an semi-automated platform toward compound libraries, which is nowadays an essential feature in drug discovery to accelerate the medicinal chemistry’s design-test cycle. Of critical importance, the protocol enables this challenging C–O bond formation with predictable selectivity in an array of >10 fused heterocycles and independent of a diverse FG decoration. The proposed intramolecular migration of the activated *N*-oxide formed *in situ* drives the complete regiocontrol achieved.

## Supplementary Material



## Notes

Experimental procedures, spectroscopic and computational data. (PDF)
