# Supplementary material for: Predictable C–H Functionalization of Complex beta-Fused Azines: A Mechanistically Bound Site-Specific Oxidation
Source: ACS Cent Sci. 2025 Jul 1;11(7):1189–98. doi: 10.1021/acscentsci.5c00797 (PMC12291144; doi:10.1021/acscentsci.5c00797)
Supplement: Supplementary file 1 [file oc5c00797_si_001.pdf]

Supporting Information

Predictable C-H Functionalization of Complex *beta*-Fused Azines: A Mechanistically Bound Site-Specific Oxidation

Carla Obradors*[a], Christopher A. Reiher[b], Cristina Grosanu[b], Mikko Muuronen[c], Romain Tessier[d], Egor M. Larin[a], and Valentin Lehuédé[a]

[a]Chemical Process Research and Development at Johnson & Johnson Innovative Medicine. Janssen Research and Development, a Division of Janssen Pharmaceutica NV. Turnhoutseweg 30, 2340 Beerse, Belgium
[b]Chemistry Capabilities, Analytical and Purification at Johnson & Johnson Innovative Medicine. Janssen Research and Development LLC. 1400 McKean Road, Spring House, PA 19477, USA
[c]Analytical Development Synthetics at Johnson & Johnson Innovative Medicine. Janssen Research and Development, a Division of Janssen Pharmaceutica NV. Turnhoutseweg 30, 2340 Beerse, Belgium
[d]Global Discovery Chemistry at Johnson & Johnson Innovative Medicine. Janssen Research and Development, a Division of Janssen Pharmaceutica NV. Turnhoutseweg 30, 2340 Beerse, Belgium

*Email: cobrador@its.jnj.com

Table of Contents

I. **General Methods**

II. **Reaction Development**
1. Nucleophile addition to *N*-oxides
2. Reaction in the absence of base and nucleophile
3. *In situ* oxidation of isoquinoline

III. **High Throughput Experimentation**
1. Screening procedure
2. Mass directed purification
3. Characterization data

IV. **General Procedure and Characterization Data**

V. **Derivatization of Isoquinolin-4-yl Tosylate**
1. Deprotection to the hydroxyl group
2. Nickel-catalyzed Kumada cross-coupling
3. Palladium-catalyzed phosphorylation reaction
4. Palladium-catalyzed Buchwald-Hartwig cross-coupling
5. Palladium-catalyzed Sonogashira cross-coupling
6. Palladium-catalyzed Suzuki cross-coupling

VI. **Preparation of Starting Materials**

VII. **Mechanistic Insights**
1. Competitive deuterium incorporation
2. Kinetic analysis
3. ^{1}H NMR monitoring
4. Computational analysis

VIII. **NMR Spectra**

I. General Methods

All reactions were carried out under air atmosphere, unless otherwise noted. All reagents and solvents were purchased commercially and used as received, unless otherwise noted. Reactions were monitored by UPLC-UV/MS using Waters Acquity UPLC I-Class Plus System coupled with PDA and SQ detectors. Column chromatography was performed on a Teledyne Isco Combiflash(R) using RediSep Rf Gold Normal-Phase Silica(R) columns (60 Å porosity, 20-40 μm). Preparative TLC was performed with TLC silica gel 60 F_{254} glass plate and stained with UV light. Mass spectra (ESI) were recorded using MeCN or MeOH as the solvent using Orbitrap Fusion Lumos mass spectrometer. NMR spectra were recorded with Bruker FT spectrometers at 300 ^{o}K, unless otherwise noted. 1H (400 MHz) and 13C {1H} (100 MHz) NMR chemical shifts are reported relative to residual protonated solvent, 19F {1H} NMR (471 MHz) chemical shifts were referenced to external CFCl3 (0.0 ppm) and 31P {1H} NMR (162 MHz) chemical shifts were referenced to external 85% H_3PO_4 in H_2O (0.0 ppm). Specific considerations regarding high throughput chemistry as well as computational analysis are included in the corresponding sections.

Acronyms used in this document: DCM (dichloromethane), DIPEA (*N,N*-diisopropylethylamine), TsCl (tosyl chloride), UPLC (ultra performance liquid chromatography), MS (mass spectrometry), UV (ultraviolet light), NMR (nuclear magnetic resonance), HRMS (high resolution mass spectrometry), Ts_2O (tosyl anhydride), Tf_2O (triflic anhydride), Ms_2O (mesyl anhydride), ^{p}Ns (*para*-nosyl group), MTO (methyl trioxorhenium), UHP (urea hydroperoxide), TEA (triethylamine), DMAP (*para*-dimethylaminopyridine), PEPPSI (pyridine enhanced precatalyst preparation stabilization and initiation), IPr (1,3-bis(2,6-diisopropylphenyl)imidazol-2-ylidene), DMA (dimethylacetamide), dme (dimethoxyethane), rac (racemic), BINAP (2,2′-bis(diphenylphosphino)-1,1′-binaphthyl), GPhos ((3-(*tert*-butoxy)-2′,6′-diisopropyl-6-methoxy-[1,1′-biphenyl]-2-yl)dicyclohexylphosphane), XPhos (dicyclohexyl[2′,4′,6′-tris(propan-2-yl)[1,1′-biphenyl]-2-yl]phosphane), ^{t}Bu (*tert*-butyl group), LCAP (liquid chromatography area of product), Fmoc (fluorenylmethoxycarbonyl), Ser (serine), ppm (parts per million), multiplicity (s = singlet, d = doublet, t = triplet), DMSO (dimethylsulfoxide), TFA (trifluoroacetic acid), ESI (electrospray ionization), DCC (*N,N′*-dicyclohexylcarbodiimide).

II. Reaction Development

1. Nucleophile addition to *N*-oxides

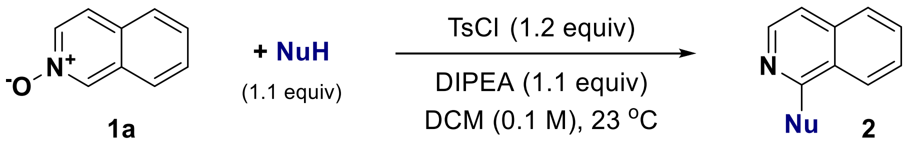

Weigh substrate **1a** (29 mg, 0.2 mmol, 1 equiv) and the nucleophile (if solid, 1.1 equiv) in a 8 ml capped vial equipped with a magnetic stirring bar. Place it under inert atmosphere by performing three cycles of high vacuum/nitrogen gas and add DCM (0.1 M) *via* syringe followed by DIPEA (1.1 equiv) and the nucleophile (if liquid, 1.1 equiv). Stir at 23 °C for 2 minutes and add TsCl (1.2 equiv). Keep stirring for 16 h and analyze the crude by UPLC-MS (5 µl of sample diluted in 195 µl of a 0.02 M solution of 4,4'-di-*tert*-butyl-1,1'-biphenyl in acetonitrile). Wash the mixture with saturated aqueous $NaHCO_3$, separate the organic phase and dry it with $MgSO_4$. Concentrate under reduced pressure and purify the desired product **2** by CombiFlash silica gel chromatography with heptane/ethyl acetate (0% to 100%). Characterize it by NMR and HRMS.

<u>When NuH is tert-butylamine:</u>

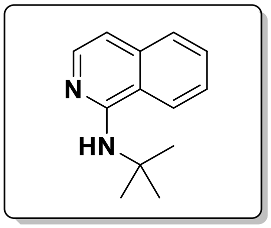

The UPLC ratio between product **2** and the isomeric mixture of tosylate addition is 2.7/1. ^{1}H NMR (400 MHz, chloroform-*d*) δ ppm 1.61 (s, 9 H), 5.15 (br s, 1 H), 6.89 (d, *J*=5.85 Hz, 1 H), 7.41 - 7.47 (m, 1 H), 7.55 (m, 1 H), 7.65 (d, *J*=8.05 Hz, 1 H), 7.69 (d, *J*=8.36 Hz, 1 H), 8.00 (d, *J*=5.85 Hz, 1 H). ^{13}C NMR (101 MHz, chloroform-*d*) δ ppm 29.19, 51.69, 110.06, 118.50, 121.24, 125.51, 127.23, 129.10, 137.02, 141.23, 154.50. HRMS (ESI$^+$) m/z calculated for $C_{13}H_{17}N_2$ $[M+H]^+$ 201.1386, found 201.1381.

When NuH is saccharin:

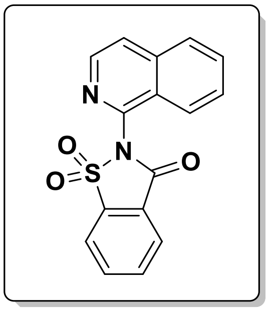

The UPLC ratio between product **2** and the isomeric mixture of tosylate addition is 1.4/1. ^{1}H NMR (400 MHz, chloroform-*d*) δ ppm 7.66 - 7.71 (m, 1 H), 7.72 - 7.83 (m, 1 H), 7.89 (d, *J*=5.72 Hz, 1 H), 7.91 - 8.02 (m, 3 H), 8.07 (d, *J*=7.48 Hz, 2 H), 8.22 (d, *J*=7.48 Hz, 1 H), 8.67 (d, *J*=5.72 Hz, 1 H). ^{13}C NMR (101 MHz, chloroform-*d*) δ ppm 121.39, 123.54, 124.92, 125.87, 127.02, 127.08, 127.40, 128.98, 131.29, 134.55, 135.44, 138.52, 138.63, 142.53, 143.01, 159.03. HRMS (ESI^{+}) m/z calculated for $C_{16}H_{11}N_2O_3S$ $[M+H]^+$ 311.0484, found 311.0477.

When NuH is para-methoxyphenol:

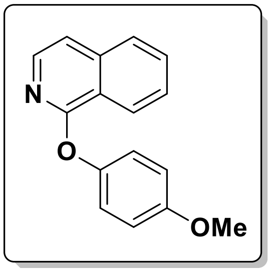

The UPLC ratio between product **2** and the isomeric mixture of tosylate addition is 0.8/1. ^{1}H NMR (400 MHz, chloroform-*d*) δ ppm 3.86 (s, 3 H), 6.97 - 7.04 (m, 2 H), 7.18 - 7.24 (m, 2 H), 7.29 - 7.34 (m, 1 H), 7.57 - 7.70 (m, 1 H), 7.71 - 7.77 (m, 1 H), 7.81 (d, *J*=8.14 Hz, 1 H), 7.99 (d, *J*=5.72 Hz, 1 H), 8.44 - 8.50 (m, 1 H). ^{13}C NMR (101 MHz, chloroform-*d*) δ ppm 55.52, 114.67, 116.01, 119.69, 122.75, 124.19, 126.14, 126.96, 130.73, 138.33, 139.81, 147.09, 156.65, 160.93. HRMS (ESI^{+}) m/z calculated for $C_{16}H_{13}NO_2$ $[M+H]^+$ 252.1019, found 252.1013.

When NuH is tetrabutylammonium bromide (TBABr):

No bromination is detected by UPLC-MS. Hydration and dimerization are observed as by-products along with the isomeric mixture of tosylate addition. *Note: the same profile is obtained in the absence of any nucleophile.*

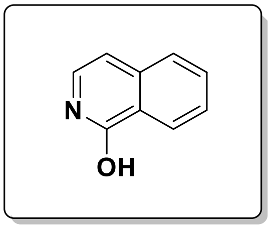

Hydration product. Comparison with authentic sample.

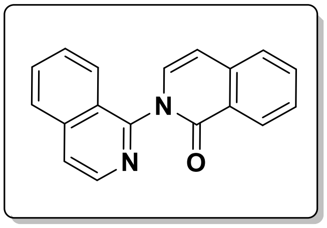

Dimerization product. ^{1}H NMR (400 MHz, chloroform-*d*) δ ppm 6.69 (d, *J*=7.48 Hz, 1 H), 7.32 (d, *J*=7.48 Hz, 1 H), 7.54 - 7.66 (m, 3 H), 7.72 - 7.80 (m, 3 H), 7.80 - 7.83 (m, 1 H), 7.96 (d, *J*=8.36 Hz, 1 H), 8.49 - 8.53 (m, 1 H), 8.55 (d, *J*=5.72 Hz, 1 H). ^{13}C NMR (101 MHz, chloroform-*d*) δ ppm 106.54, 122.34, 124.83, 125.17, 126.17, 126.40, 127.05, 127.27, 128.31, 128.47, 130.92, 131.18, 132.90, 137.44, 138.02, 141.65, 152.84, 162.28. HRMS (ESI^{+}) m/z calculated for $C_{18}H_{13}N_2O$ $[M+H]^+$ 273.1022, found 273.1016.

2. Reaction in the absence of base and nucleophile

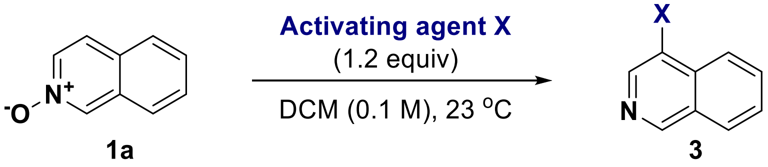

Weigh substrate **1a** (29 mg, 0.2 mmol, 1 equiv) in a 8 ml capped vial equipped with a magnetic stirring bar. Place it under inert atmosphere by performing three cycles of high vacuum/nitrogen gas and add DCM (0.1 M) *via* syringe. Stir at 23 ºC for 2 minutes and add the activating agent **X** (1.2 equiv). Keep stirring for 16 h and analyze the crude by UPLC-MS (5 μl of sample diluted in 195 μl of a 0.02 M solution of 4,4'-di-*tert*-butyl-1,1'-biphenyl in acetonitrile).

When activating agent **X** is Ts_2O:

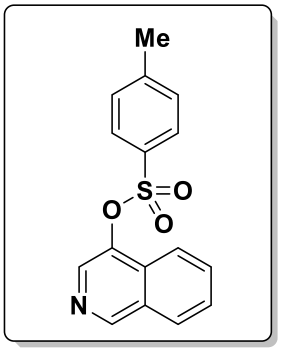

Compound 3a. Concentrate the crude under reduced pressure and add trimethoxybenzene to measure the NMR yield (84% of product **3a**). Reaction at 0.5 mmol scale: wash the mixture with saturated aqueous $NaHCO_3$, separate the organic phase and dry it with $MgSO_4$. Concentrate under reduced pressure and purify the desired product by CombiFlash silica gel chromatography (DCM injection). Separation with heptane/ethyl acetate (0% to 100%) leads to 78% yield as a beige powder (46.5 mg, 0.15 mmol). ^1H NMR (400 MHz, chloroform-*d*) δ ppm 2.44 (s, 3 H), 7.33 (d, *J*=8.14 Hz, 2 H), 7.60 - 7.68 (m, 1 H), 7.72 (m, 1 H), 7.80 (d, *J*=8.36 Hz, 2 H), 7.96 - 8.02 (m, 2 H), 8.12 (s, 1 H), 9.14 (s, 1 H). ^{13}C NMR (101 MHz, chloroform-*d*) δ ppm 21.67, 121.11, 127.21, 128.15, 128.54, 129.99, 130.46, 131.18, 132.08, 135.66, 145.90, 151.08. ^1H COSY, ^{13}C HSQC and ^{13}C HMBC included to confirm regioselectivity. HRMS (ESI$^+$) m/z calculated for $C_{16}H_{14}NO_3S$ $[M+H]^+$ 300.0688, found 300.0683. Reaction at 1 g scale leads to 78% isolated yield.

When activating agent **X** is TsCl:
Same product observed in 57% NMR yield.

When activating agent **X** is Ms_2O:

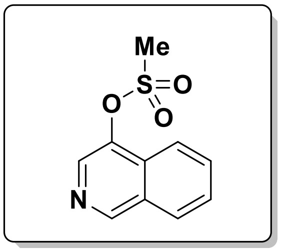

Compound 3v. Concentrate the crude under reduced pressure and add trimethoxybenzene to measure the NMR yield (62% of product **3v**). Reaction at 0.5 mmol scale: concentrate and purify the desired product directly by CombiFlash silica gel chromatography (DCM injection). Separation with heptane/ethyl acetate (0% to 100%) leads to 60% yield as a beige powder (67 mg, 0.3 mmol). ^1H NMR (400 MHz, chloroform-*d*) δ ppm 3.34 (s, 3 H), 7.67 - 7.77 (m, 1 H), 7.82 – 7.87 (m, 1 H), 8.06 (d, *J*=8.26 Hz, 1 H), 8.13 - 8.19 (m, 1 H), 8.58 (s, 1 H), 9.22 (s, 1 H). ^{13}C NMR (101 MHz, chloroform-*d*) δ ppm 38.24, 120.89, 127.49, 128.41, 129.89, 130.24, 131.62, 135.69, 141.59, 151.50. ^1H COSY, ^{13}C HSQC and ^{13}C HMBC included to confirm regioselectivity. HRMS (ESI$^+$) m/z calculated for $C_{10}H_{10}NO_3S$ $[M+H]^+$ 224.0375, found 224.0372.

When activating agent **X** is ^{p}NsCl:

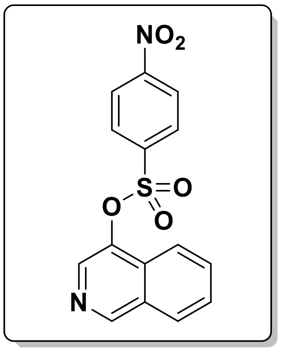

Compound 3w. Reaction at 0.5 mmol scale: wash the mixture with saturated aqueous $NaHCO_3$ and separate the organic phase. Concentrate under reduced pressure and directly purify the desired product by CombiFlash silica gel chromatography (DCM injection). Separation with heptane/ethyl acetate (0% to 100%) leads to 51% yield of product **3w** as dark yellow crystals (83.8 mg, 0.25 mmol). *Note: reaction performed in the presence of 5equiv. H_2O.* ^{1}H NMR (400 MHz, chloroform-*d*) δ ppm 7.66 - 7.72 (m, 1 H), 7.72 - 7.78 (m, 1 H), 7.86 - 7.99 (m, 1 H), 8.03 (d, *J*=8.14 Hz, 1 H), 8.13 - 8.18 (m, 2 H), 8.21 (s, 1 H), 8.32 - 8.44 (m, 2 H), 9.19 (s, 1 H). ^{13}C NMR (101 MHz, chloroform-*d*) δ ppm 120.54, 124.57, 127.50, 128.52, 129.86, 129.94, 129.97, 131.59, 135.60, 140.83, 141.75, 151.20, 151.83. ^{1}H COSY, ^{13}C HSQC and ^{13}C HMBC included to confirm regioselectivity. HRMS (ESI$^+$) m/z calculated for $C_{15}H_{10}N_2O_5S$ $[M+H]^+$ 331.0383, found 331.0382.

When activating agent **X** is SO_2Cl_2:

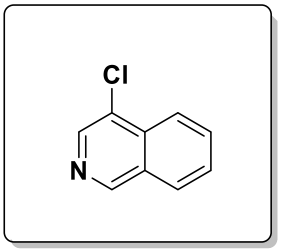

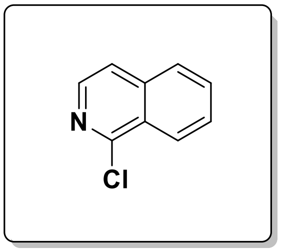

Compound 3x. Dilute the mixture with DCM, wash with saturated aqueous $NaHCO_3$, separate the organic phase and dry it with $MgSO_4$. Concentrate under reduced pressure and add trimethoxybenzene to measure the NMR yield (38% of **3x** and 47% of **2**). *Note: DCM (0.5 M), SO_2Cl_2 (18 equiv) and stirred for 3 days.* Comparison with authentic samples.

When using other activating agents **X**:
Triflic anhydride, acetic anhydride, bis(trimethylsilyl) sulfate, tris(trimethylsilyl) phosphate or 3-(fluorosulfonyl)-1,2-dimethyl-1H-imidazol-3-ium trifluoromethanesulfonate led to no C–H functionalization of isoquinoline and only partial hydration and/or dimerization were observed.

3. *In situ* oxidation of isoquinoline

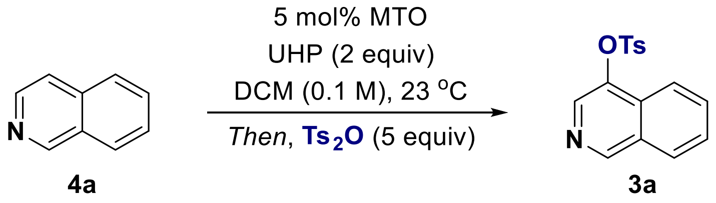

Weigh substrate **4a** (24 μl, 0.2 mmol, 1 equiv) in a 8 ml capped vial equipped with a magnetic stirring bar. Dissolve in dry DCM (0.1 M) and add MTO (5 mol%) followed by UHP (2 equiv). Stir at 23 °C for 16 h and analyze the crude by UPLC-MS (5 μl of sample diluted in 195 μl of a 0.02 M solution of 4,4'-di-*tert*-butyl-1,1'-biphenyl in acetonitrile). Add Ts_2O (5 equiv), keep stirring for 2 h and analyze the crude by UPLC-MS again. Concentrate the mixture under reduced pressure and add trimethoxybenzene to measure the NMR yield (78% of product **3a**). Reaction at 0.5 mmol scale: dilute the crude with DCM and quench with saturated aqueous $NaHCO_3$. Separate phases, concentrate under reduced pressure and directly purify the desired product by CombiFlash silica gel chromatography (DCM injection). Separation with heptane/85:15 ethyl acetate and 7N ammonia in methanol (0% to 100%) leads to 68% yield over the two steps (101 mg, 0.34 mmol). Reaction at 1 g scale leads to 63% isolated yield.

<u>Deviation from the standard conditions (LCAP):</u>

None: 84%
Overnight: 72%
1.2 equiv/overnight: 47%. *Compound **12** formed as by-product.*
4Å MS: 88%
4 Å MS/overnight: 85%
4 Å MS/overnight at 0.04 M: 46% (51% conversion)
4 Å MS/overnight at 0C: 75%
4 Å MS in THF: 24% (63% conversion)
4 Å MS in PhMe: no reaction
4 Å MS in DMA: 25%
4 Å MS in MeCN: 26% (50% conversion)

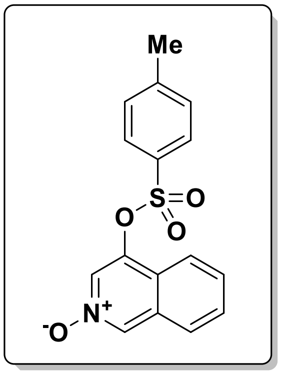

Compound 12. ^{1}H NMR (400 MHz, chloroform-*d*) δ ppm 2.47 (s, 3 H), 7.38 (d, *J*=7.92 Hz, 2 H), 7.57 - 7.67 (m, 2 H), 7.69 - 7.73 (m, 1 H), 7.82 - 7.86 (m, 2 H), 7.89 - 7.93 (m, 2 H), 8.67 (s, 1 H). ^{13}C NMR (101 MHz, chloroform-*d*) 21.77, 121.77, 124.60, 124.73, 128.53, 129.49, 130.35, 130.51, 130.83, 131.44, 135.08, 144.12, 146.70. ^{1}H COSY, ^{13}C HSQC and ^{13}C HMBC included to confirm regioselectivity. HRMS (ESI^+) m/z calculated for $C_{16}H_{13}NO_4S$ $[M+H]^+$ 316.0638, found 316.0636.

III. High Throughput Experimentation

1. Screening procedure

Available isoquinolines and quinolines (approx. 4k total) were mined from an internal database (Johnson and Johnson) and clustered by molecular fingerprints using a self-organizing map (SOM) algorithm in Pipeline Pilot to visualize the accessible chemical space. Manual substrate selections were then made with the following considerations in mind: 1) enrich for medicinal chemistry-relevant substructures; 2) incorporate matched molecular pairs to enable trend identification; 3) incorporate diverse electronic and steric features; and 4) select across multiple clusters to enhance library diversity. The compounds were purchased for a 96-well plate library (0.1 mmol scale) that led to a 30% success rate.

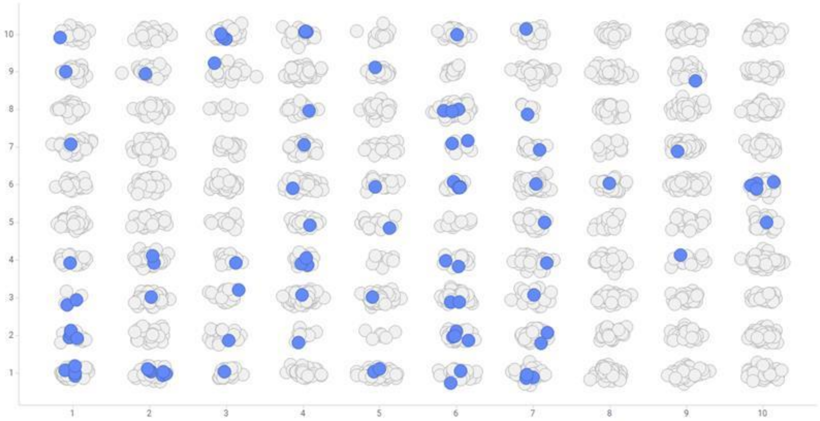

Self-organizing map representation of accessible quinolines and isoquinolines
Library selections highlighted in blue

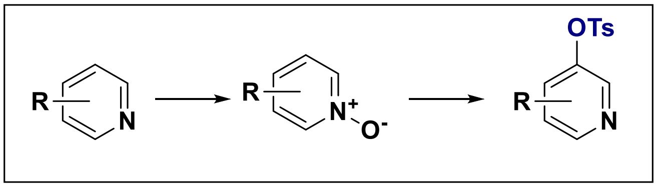

To each substrate (0.1 mmol) was added a stock solution of methyltrioxorhenium (2.492 mg, 0.01 mmol, 0.1 equiv) in DCM (0.5 mL). Approximately 18.8 mg (0.2 mmol, 2 equiv) of urea hydrogen peroxide was added to each reaction mixture as a solid. The plates were sealed and stirred at ambient temperature 16 h.

A stock solution of *p*-toluenesulfonic anhydride (16 g, 49.0 mmol) in DCM (200 mL) was prepared and stirred over 4Å molecular sieves (10 g). The plate was unsealed. Additional DCM (0.5 mL) was added to each reaction mixture, and the reactions were stirred briefly. Approximately 100 mg of 4Å molecular sieves was added to each reaction mixture, and the mixtures were stirred 15 min.

Then, the stock solution of *p*-toluenesulfonic anhydride (2 mL, 0.49 mmol) was added to each reaction mixture. The vials were sealed and stirred at ambient temperature 16 h.

Saturated $NaHCO_3$ (aq, 2 mL) was added to each reaction mixture, and the mixtures were stirred for 15 min. Stirring was stopped, the biphasic mixtures were allowed to settle, and the reaction mixtures were then transferred to Isolute Phase Separators to filter the organic phase into 8 mL vials. The reaction mixtures were concentrated under reduced pressure in a Genevac EZ-2. The crude mixtures were dissolved in DMSO, and successful reactions were purified by reverse phase HPLC using a TFA modifier.

2. Details regarding high throughput mass directed purification

Pre- and post-purification characterization completed on an Acquity I-Class UPLC-MS system from Waters running with both acidic (TFA) and basic (NH_4OH) modifiers.

Purification processes were done on an Autopurification Waters UV/MS system, with Auto Blend Technology capable to auto-generate gradients, and to monitor the elution of each targeted compound. The autopurification platform consists of: 2545 binary gradient module (HPLC pump), 2767 injector/collector, 2-515 analytical HPLC pumps for At Column Dilution sample loading and MS make-up flow, Waters static flow splitters, 2996 photodiode Array UV detector, and SQD2 mass spectrometer. High throughput purification is based on both UV absorbance and MS signal to trigger collection.

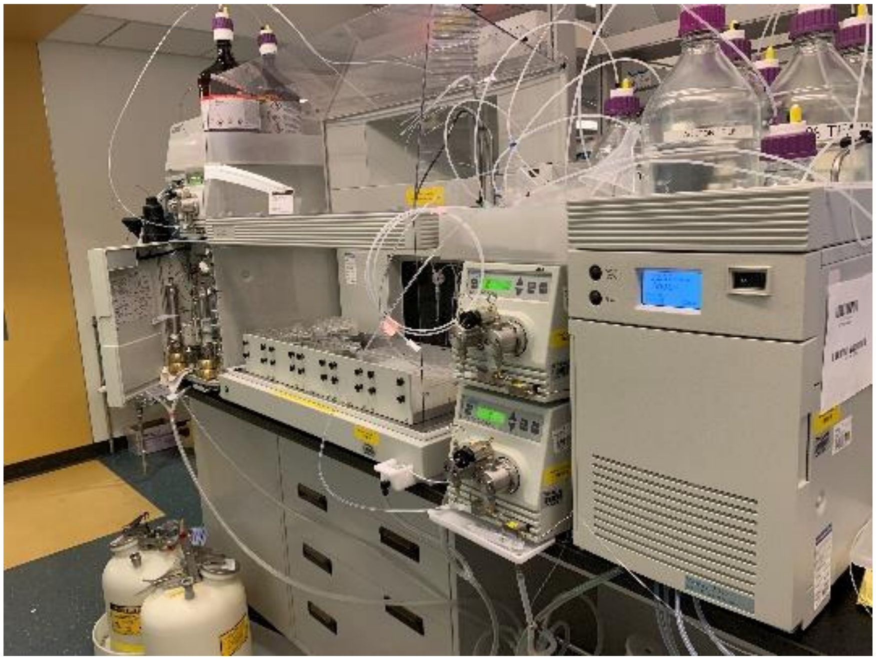

Waters Autopurification System

To validate this purification process, a standard mixture composed of commercially available compounds were subjected to purification. Generic purification methods were used, each separated peak was dried and weighed to validate robust instrument performance. Fractions were collected in vials that can hold up to 20 mL. Recoveries were consistently above 90%.

All HTE compounds were purified using TFA as a modifier. The primary stationary phase was a XSelect CSH C18 5um OBD 19x150mm column. Purification was done with appropriate linear gradients of increasing concentration of acetonitrile in water, 0.1% TFA, flow rate 25 mL/min. A few sample gradient examples are shown below:

TFA_25mL_15_50_8m_V3_C3
TFA_25mL_8_30_8m_V3_C3
TFA_25mL_10_40_8m_V3_C3

Fractions containing the desired product were combined and dried on a Genevac.

Post purification processes include post-QC plate creation and analysis of every compound, solvent evaporation, and weighing.

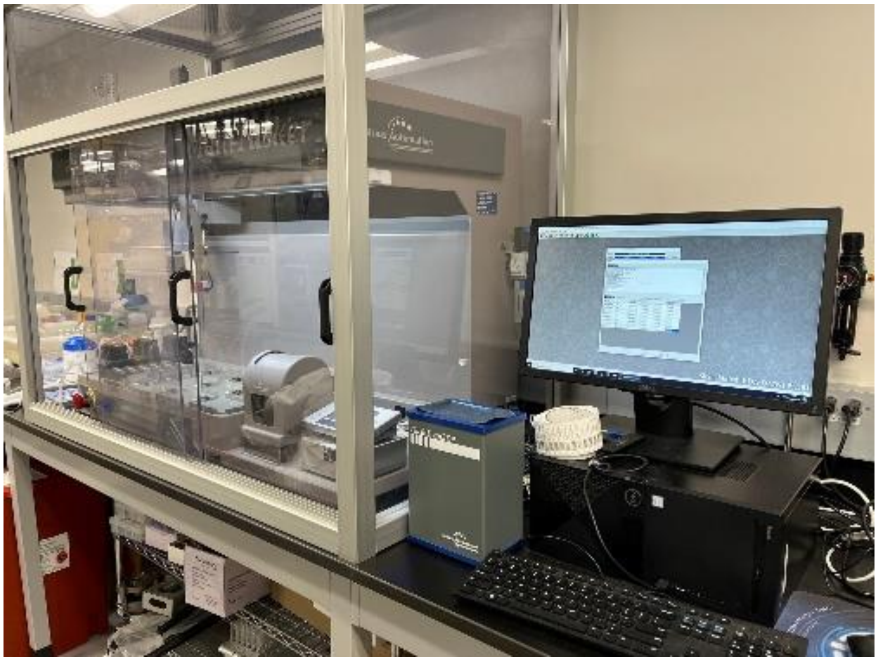

Sirius Automated Weigher

The fraction QC plate is dried and submitted to High Throughput NMR for structure validation.

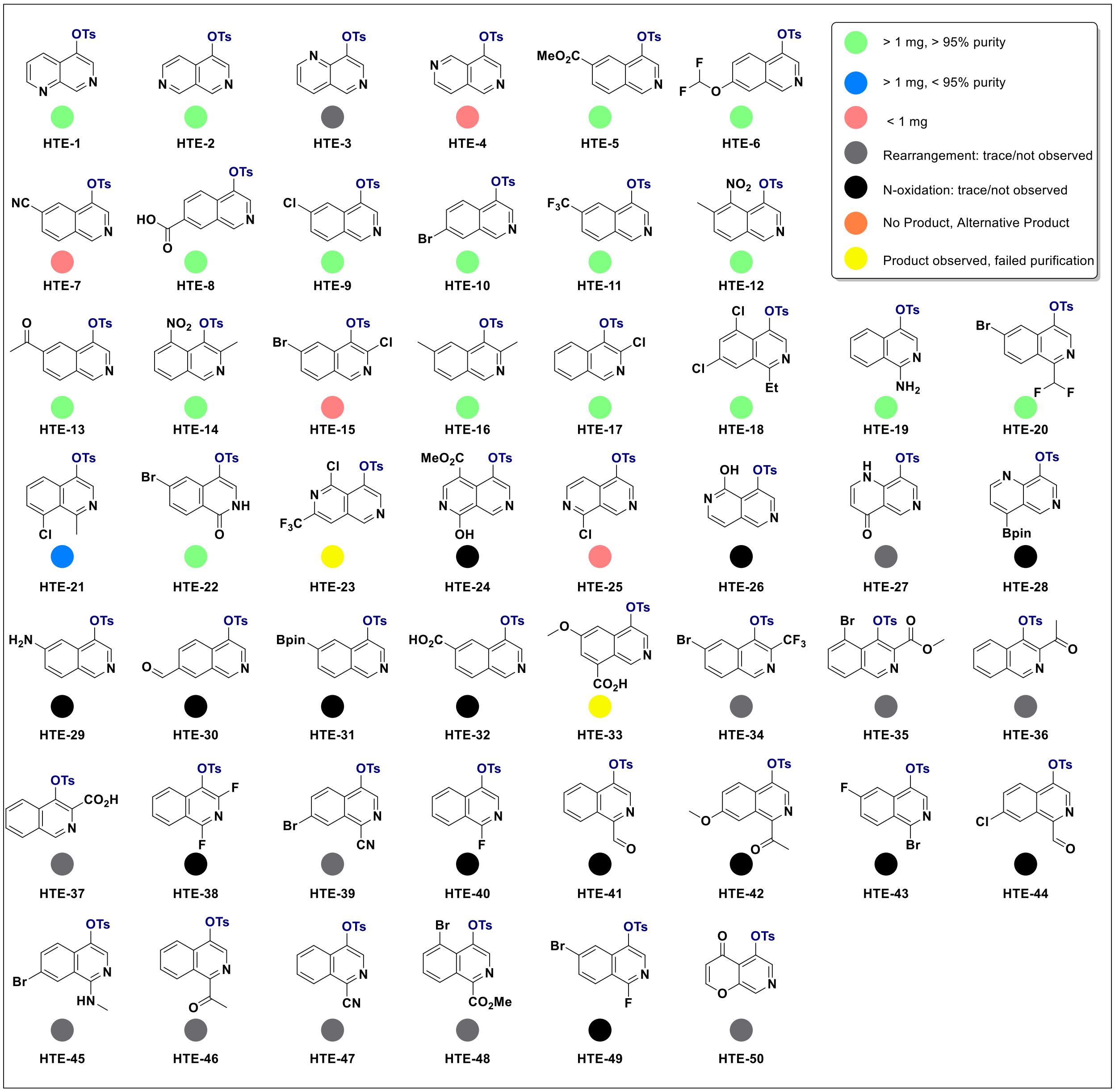

Results of HTE screen. 6,6-Fused ring systems

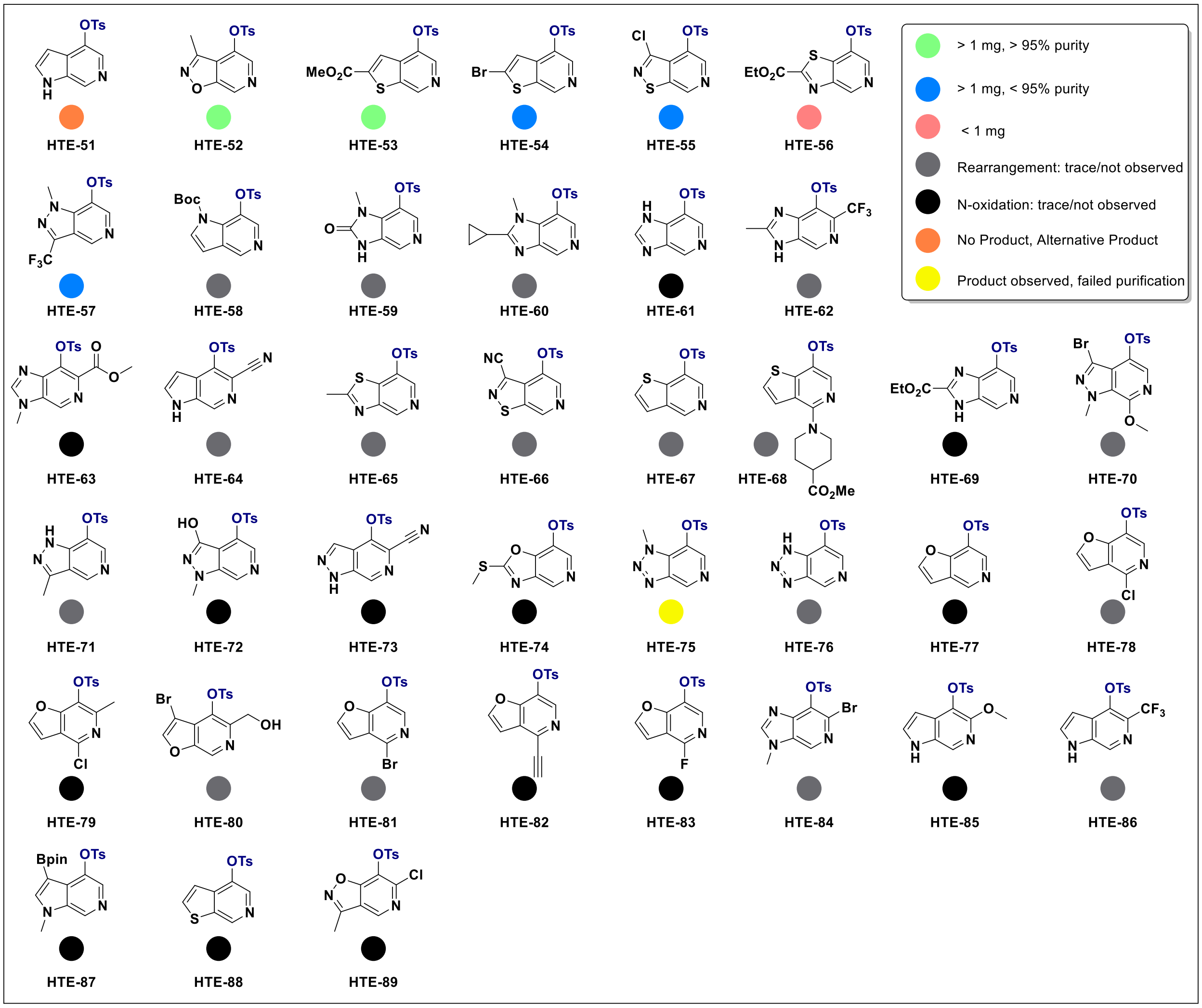

Results of HTE screen. 6,5-Fused ring systems

3. Characterization data for HTE compounds

Below is data for compounds prepared in library format (high throughput experimentation) and isolated via high-throughput purification techniques. Unless otherwise indicated, mass quantities indicate compounds isolated at >95% purity by LCMS. Proof of structure is supported via high-throughput LCMS trace data and high-throughput NMR data (with water/solvent suppression). In certain cases, suppression in this manner led to difficulties with improper integration and/or absence of peaks. In these cases, spectral images of the non-suppressed spectra are given (indicated where applicable).

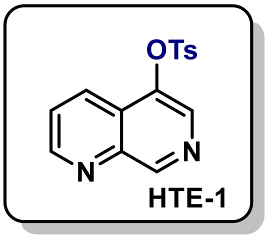

1,7-Naphthyridin-5-yl 4-methylbenzenesulfonate (HTE-1)

Quantity Obtained: 2.2 mg (7% isolated yield, 46% LCAP)

^{1}H NMR (400 MHz, DMSO-d_6) δ 9.39 (s, 1H), 9.11 (d, J=2.9 Hz, 1H), 8.37-8.20 (m, 2H), 7.73-7.93 (m, 3H), 7.47 (d, J=8.1 Hz, 2H), 2.41 (s, 3H)

^{1}H NMR Spectra (with suppression):

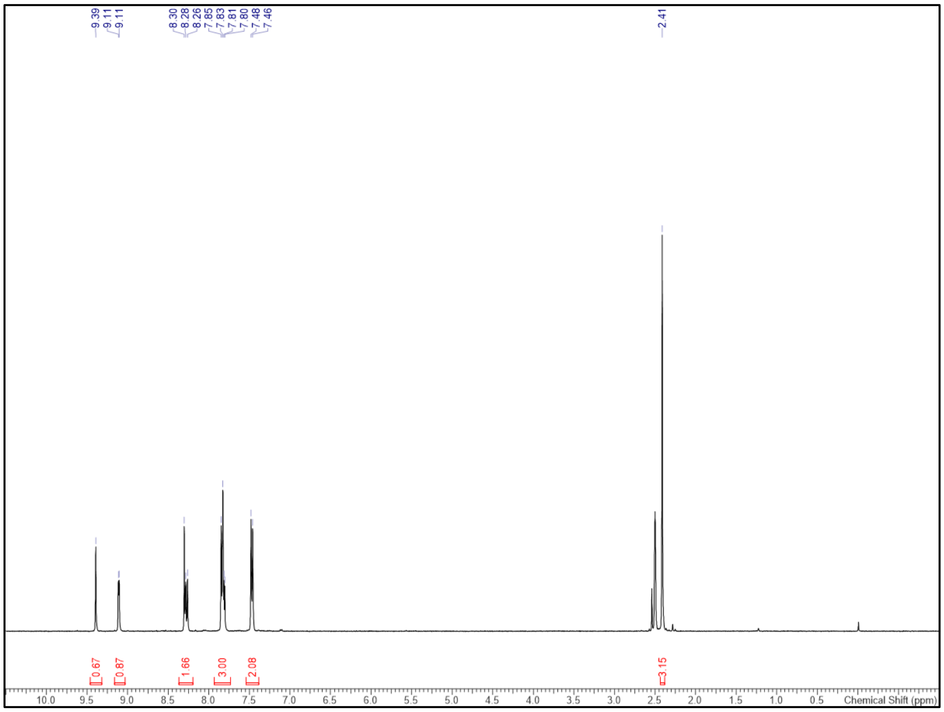

LCMS Data: Retention Time: 0.876 min; **MS ES+** ([M+H]$^+$): 300.9.

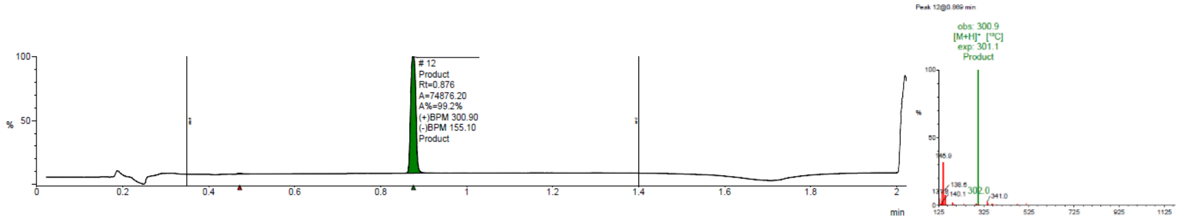

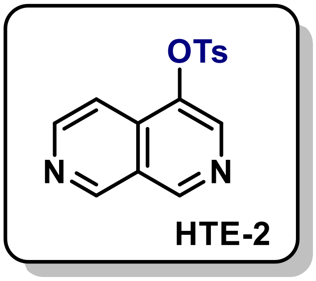

2,7-Naphthyridin-4-yl 4-methylbenzenesulfonate (HTE-2)

Quantity Obtained: 1.0 mg (3% isolated yield, LCAP not measurable)

^{1}H NMR (400 MHz, DMSO-d_6) δ 9.63 (s, 1H), 9.53 (s, 1H), 8.76 (d, J = 5.9 Hz, 1H), 8.45 (s, 1H), 7.85 (d, J = 8.3 Hz, 2H), 7.67 (d, J = 6.1 Hz, 1H), 7.48 (d, J = 8.1 Hz, 2H), 2.42 (s, 3H)

^{1}H NMR Spectra (with suppression)

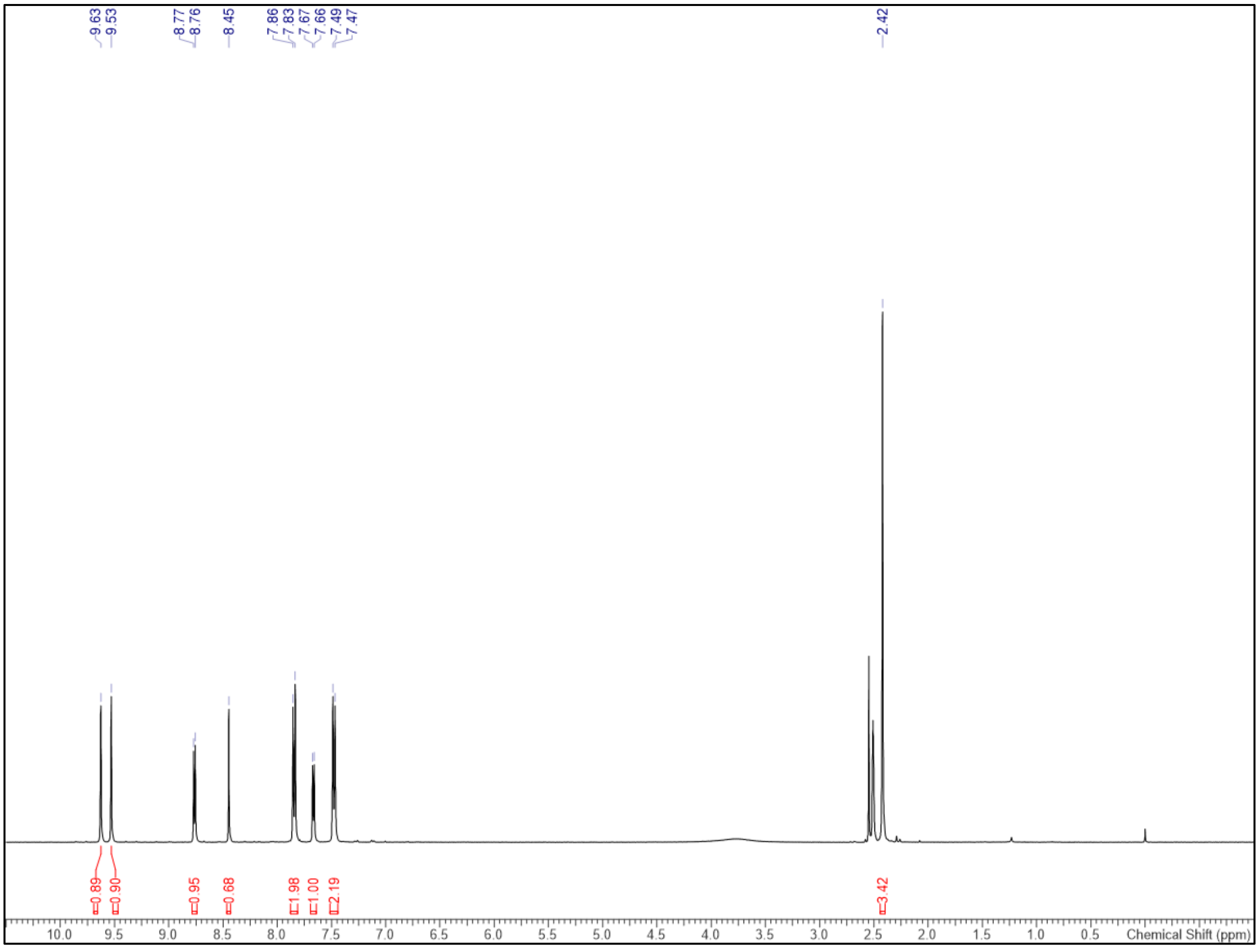

LCMS Data: Retention Time: 0.790 min; **MS ES+** ([M+H]$^+$): 301.0.

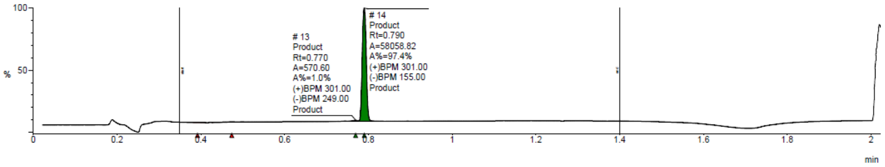

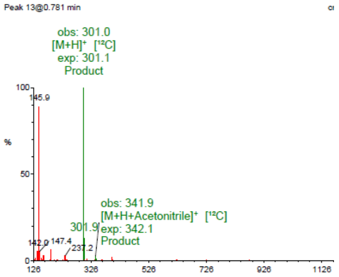

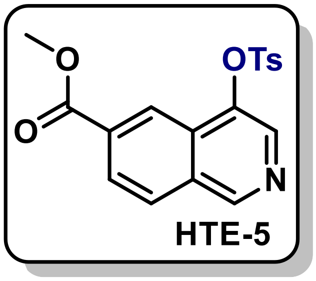

Methyl 4-(tosyloxy)isoquinoline-6-carboxylate (HTE-5)

Quantity Obtained: 6.3 mg (18% isolated yield, 51% LCAP)

^{1}H NMR (400 MHz, DMSO-d_6) δ 9.43 (s, 1H), 8.35 (d, J = 8.6 Hz, 1H), 8.32 (s, 1H), 8.23 (s, 1H), 8.17 (dd, J = 1.0, 8.6 Hz, 1H), 7.80 (d, J = 8.3 Hz, 2H), 7.47 (d, J = 8.3 Hz, 2H), 3.95 (s, 3H), 2.40 (s, 3H)

^{1}H NMR Spectra (with suppression):

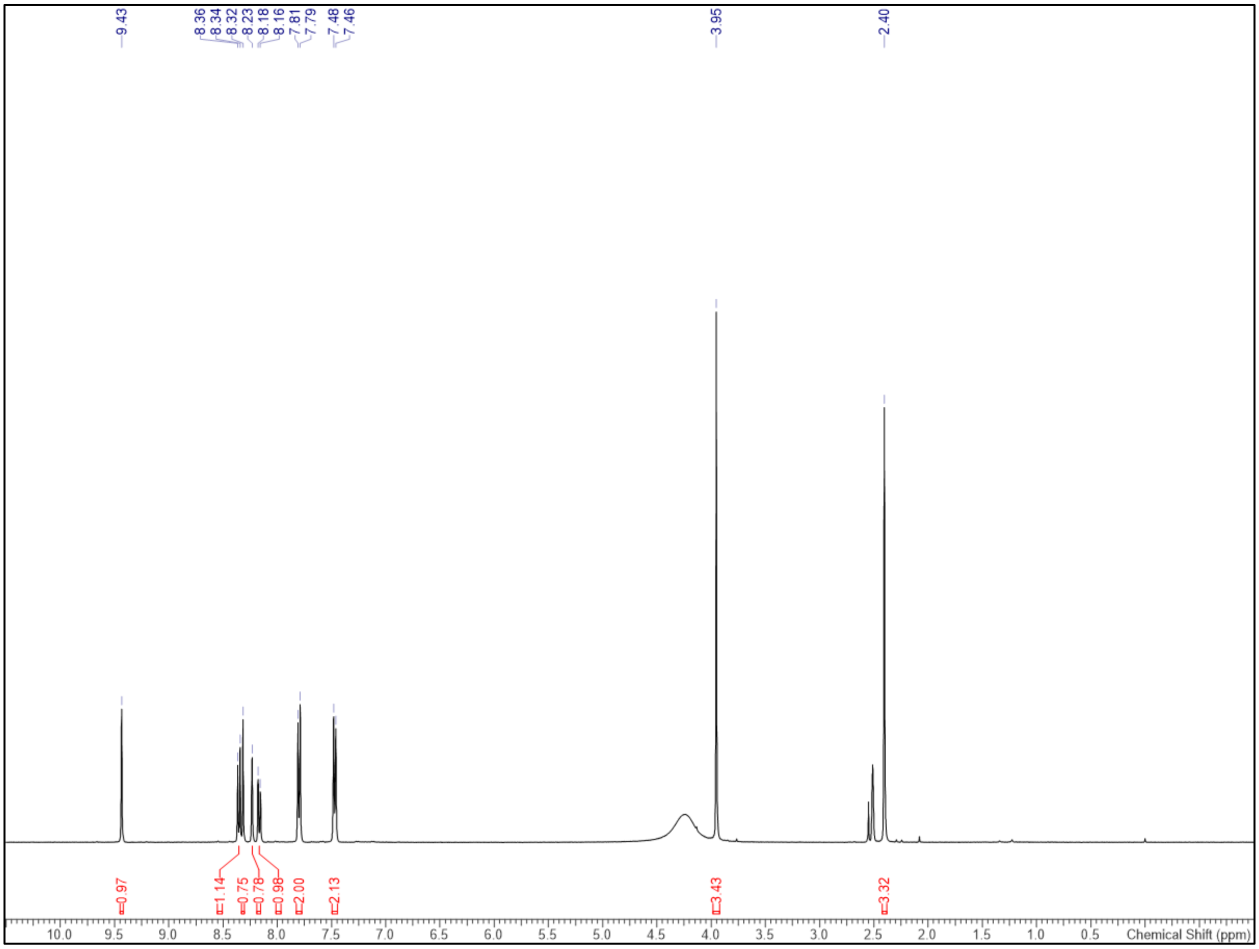

LCMS Data: Retention Time: 1.019 min; **MS ES+** ([M+H]$^{+}$): 357.8.

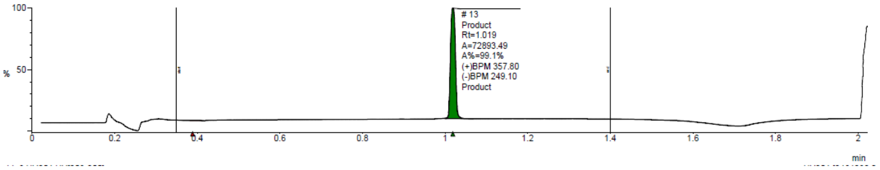

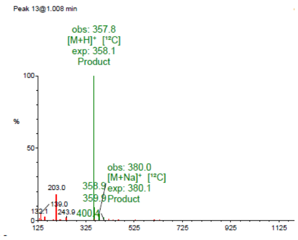

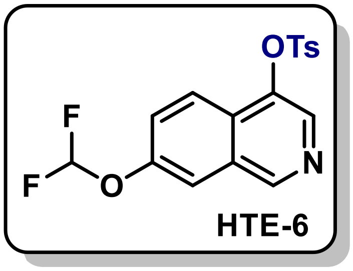

7-(Difluoromethoxy)isoquinolin-4-yl 4-methylbenzenesulfonate (HTE-6)

Quantity Obtained: 3.0 mg (8% isolated yield, 42% LCAP)

^{1}H NMR (400 MHz, DMSO-d_6) δ 9.30 (s, 1H), 8.15 (s, 1H), 7.99 (s, 1H), 7.94 (d, *J* = 9.0 Hz, 1H), 7.85 (d, *J* = 8.3 Hz, 2H), 7.68 (dd, *J* = 2.2, 9.3 Hz, 1H), 7.49 (br d, *J* = 8.1 Hz, 2H), 7.45 (t, *J* = 73.2 Hz), 2.43 (s, 3H)

^{1}H NMR Spectra (with suppression):

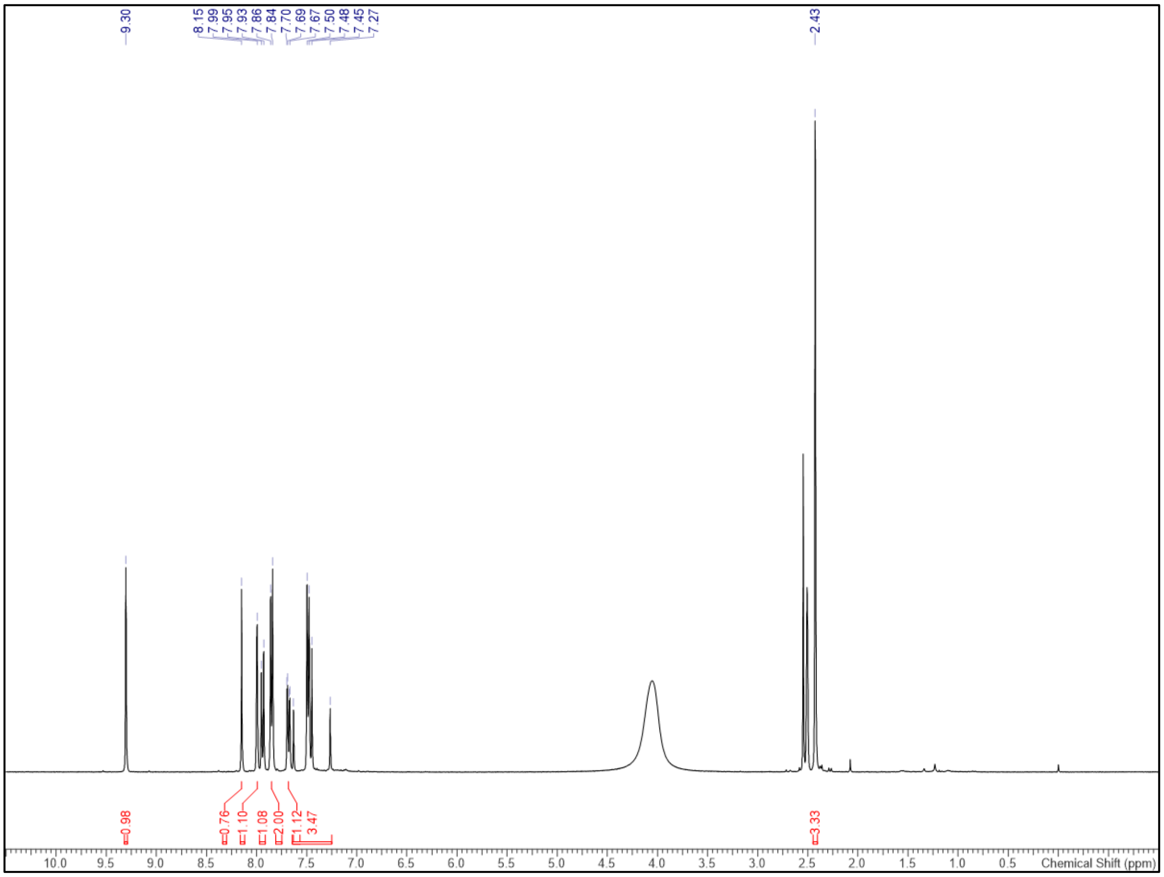

LCMS Data: Retention Time: 1.039 min; **MS ES+** ([M+H]$^+$): 366.0.

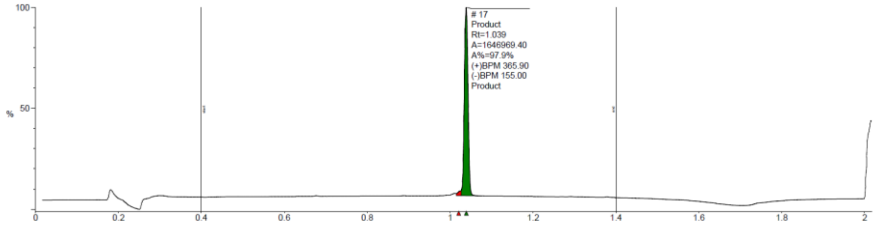

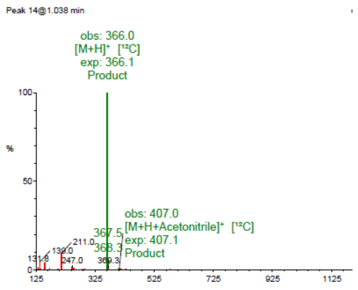

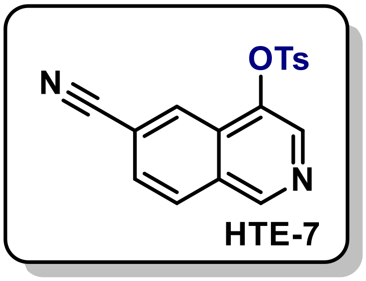

6-Cyanoisoquinolin-4-yl 4-methylbenzenesulfonate (HTE-7, 3b)

Quantity Obtained: 0.5 mg (2% isolated yield, 42% LCAP)

^{1}H NMR (400 MHz, DMSO-d_6) δ 9.46 (s, 1H), 8.46 - 8.40 (m, 2H), 8.09 - 8.02 (m, 2H), 7.83 (d, J = 8.3 Hz, 2H), 7.46 (d, J = 8.1 Hz, 2H), 2.42 (s, 3H)

^{1}H NMR Spectra (with suppression):

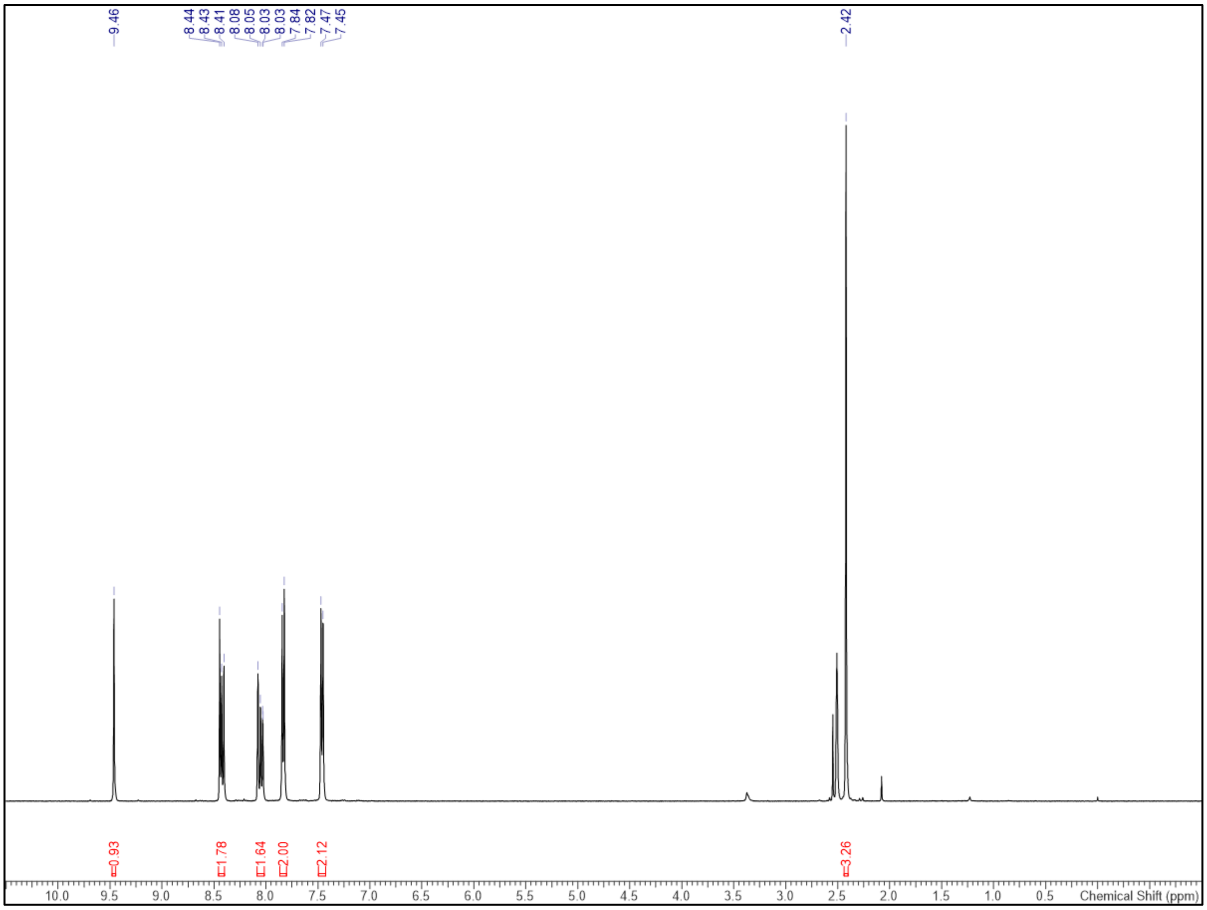

LCMS Data: Retention Time: 0.998 min; **MS ES+** ([M+H]$^+$): 325.0.

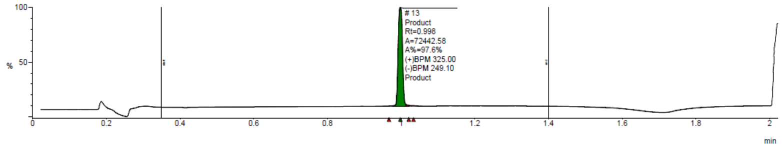

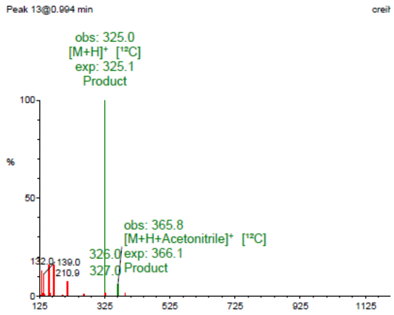

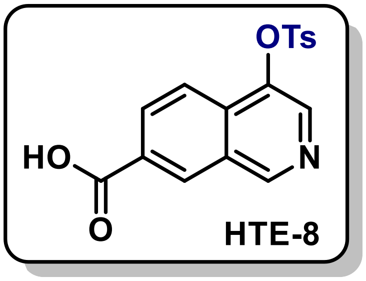

4-(Tosyloxy)isoquinoline-7-carboxylic acid (HTE-8)

Quantity Obtained: 4.4 mg (13% isolated yield, 88% LCAP)

^{1}H NMR (400 MHz, DMSO-d_6) δ 9.49 (s, 1H), 8.87 (s, 1H), 8.28 - 8.22 (m, 2H), 7.94 (d, J = 8.8 Hz, 1H), 7.85 (d, J = 8.3 Hz, 2H), 7.52 - 7.43 (m, 2H), 2.42 (s, 3H)

^{1}H NMR Spectra (with suppression):

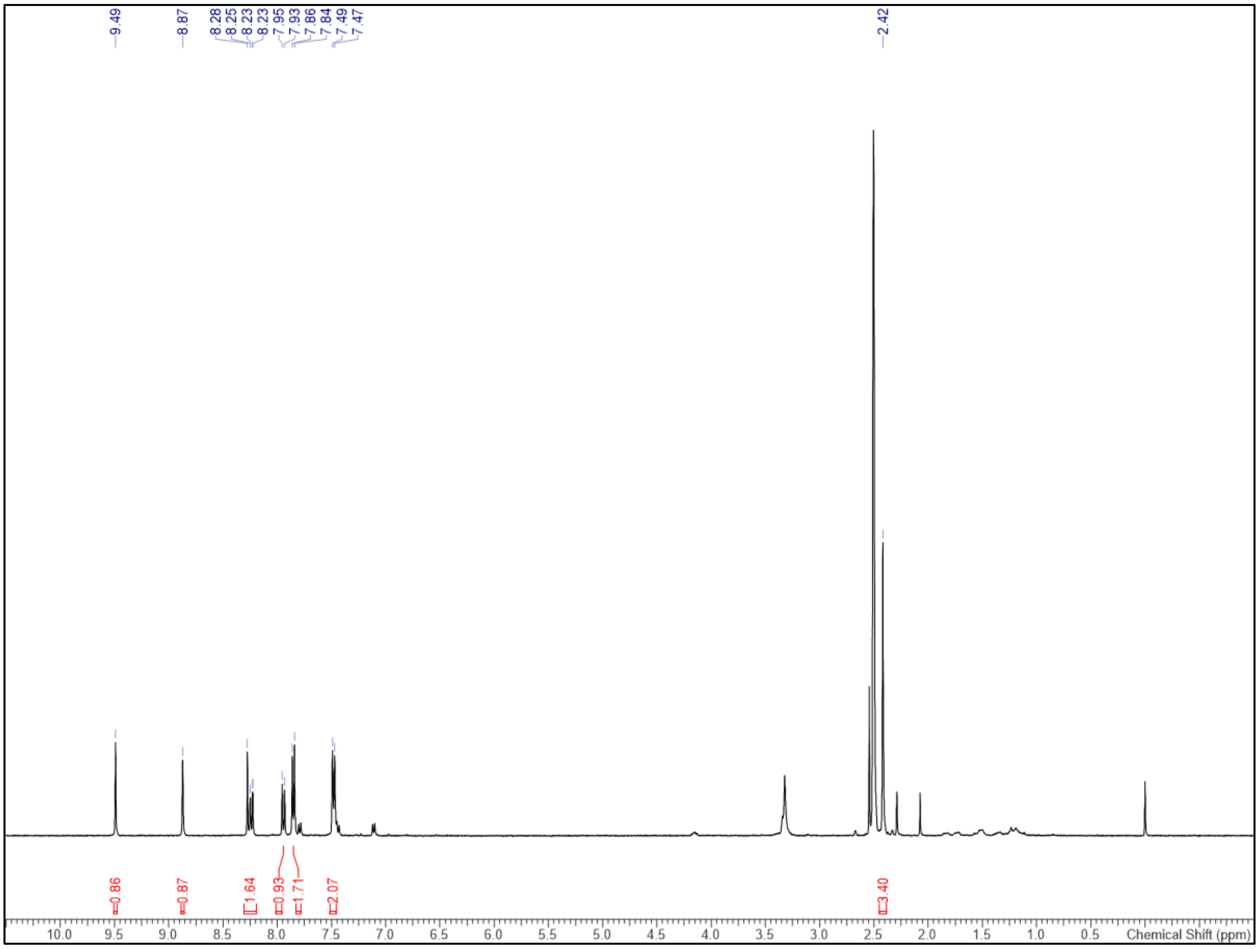

LCMS Data: Retention Time: 0.894 min; **MS ES+** ([M+H]$^+$): 344.2.

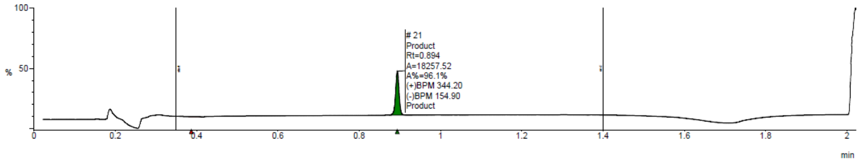

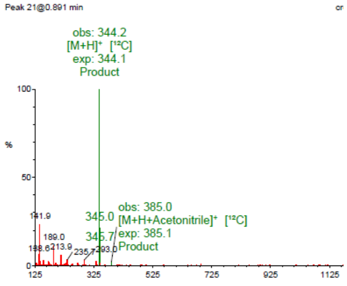

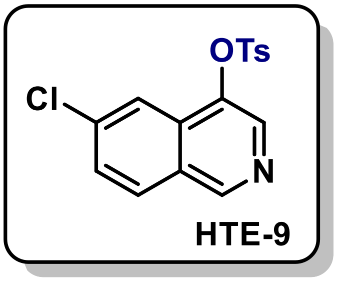

HTE-9

6-Chloroisoquinolin-4-yl 4-methylbenzenesulfonate (HTE-9)

Quantity Obtained: 5.0 mg (15% isolated yield, 47% LCAP)

^{1}H NMR (400 MHz, DMSO-d_6) δ 9.34 (s, 1H), 8.30 - 8.25 (m, 2H), 7.84 - 7.75 (m, 3H), 7.60 (s, 1H), 7.47 (d, J = 8.1 Hz, 2H), 2.42 (s, 3H)

^{1}H NMR Spectra (with suppression):

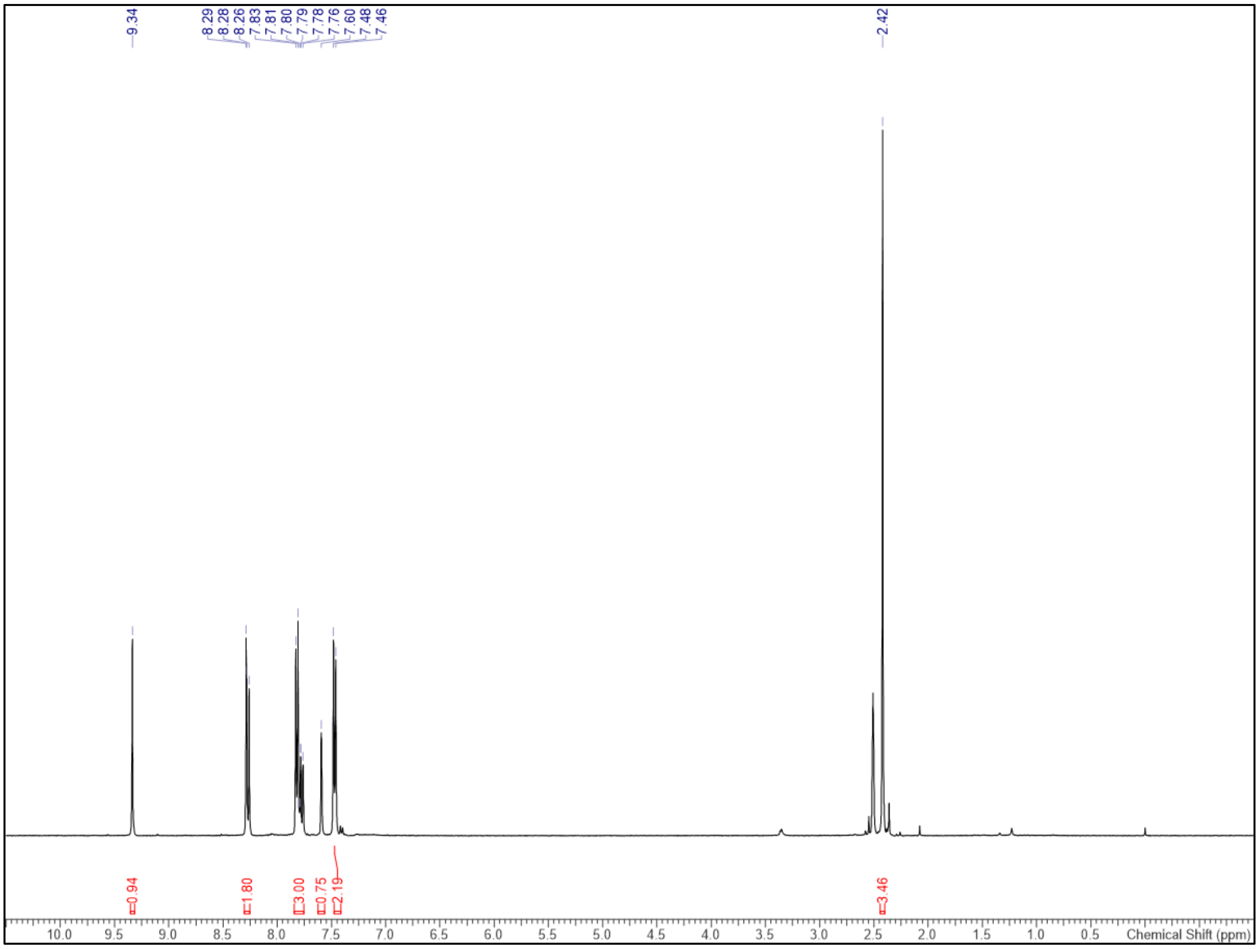

LCMS Data: Retention Time: 1.07 min; **MS ES+** ([M+H]$^+$): 333.8.

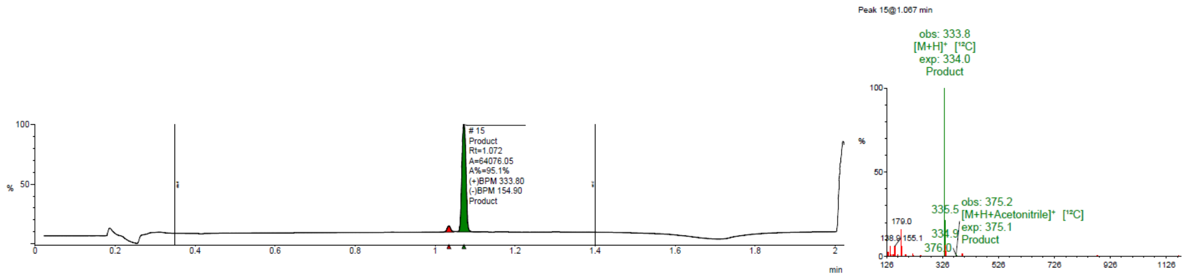

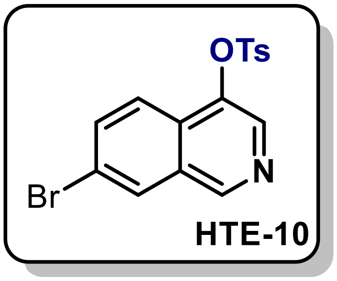

7-Bromoisoquinolin-4-yl 4-methylbenzenesulfonate (HTE-10)

Quantity Obtained: 7.4 mg (20% isolated yield, 51% LCAP)

^{1}H NMR (400 MHz, DMSO-d_6) δ 9.28 (s, 1H), 8.54 (s, 1H), 8.21 (s, 1H), 7.96 (dd, J = 1.5, 9.0 Hz, 1H), 7.87 - 7.78 (m, 3H), 7.49 (d, J = 8.1 Hz, 2H), 7.12 (br d, J = 7.8 Hz, 1H), 2.43 (s, 3H)

^{1}H NMR Spectra (with suppression):

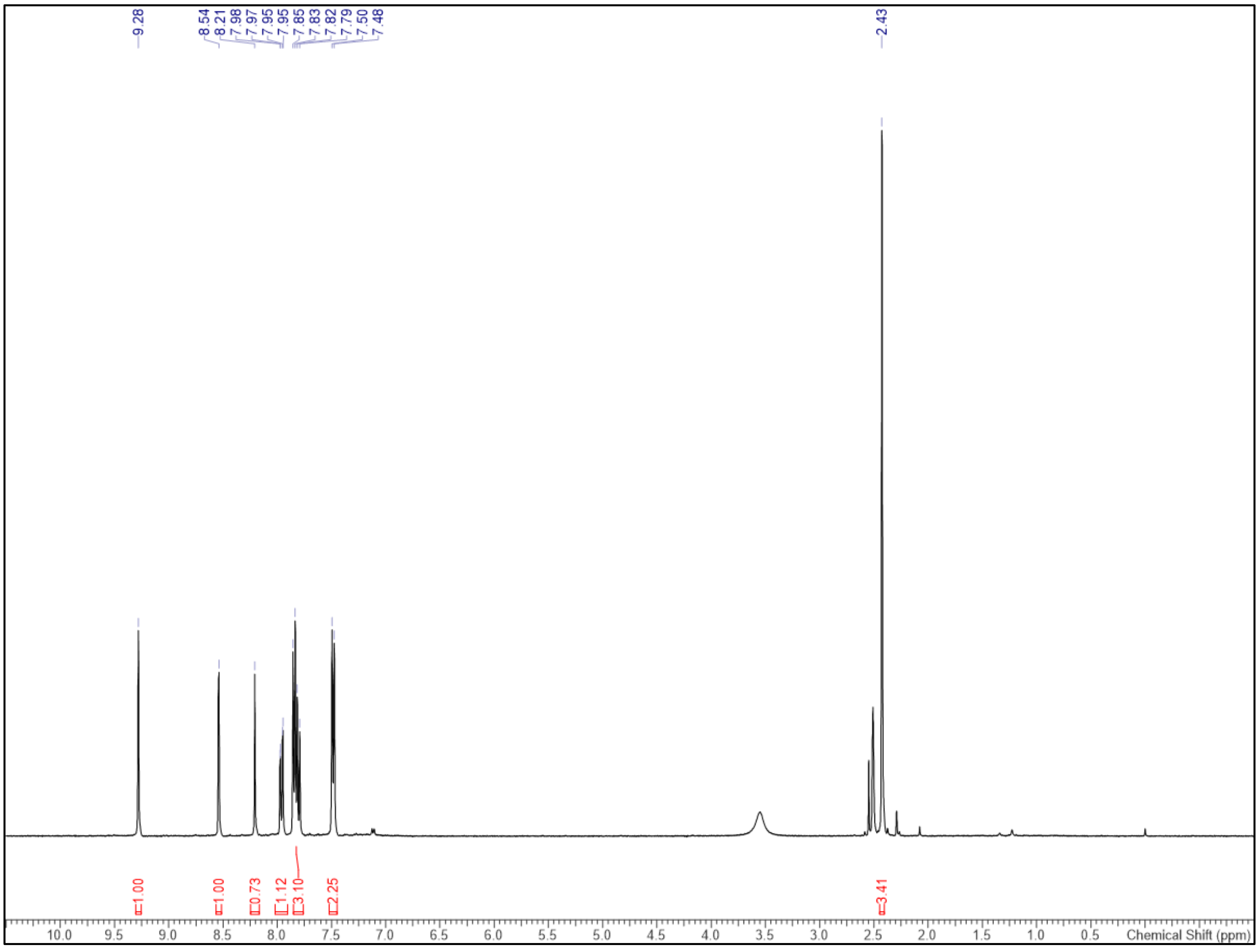

LCMS Data: Retention Time: 1.121 min; **MS ES+** ([M+H]$^+$): 377.9.

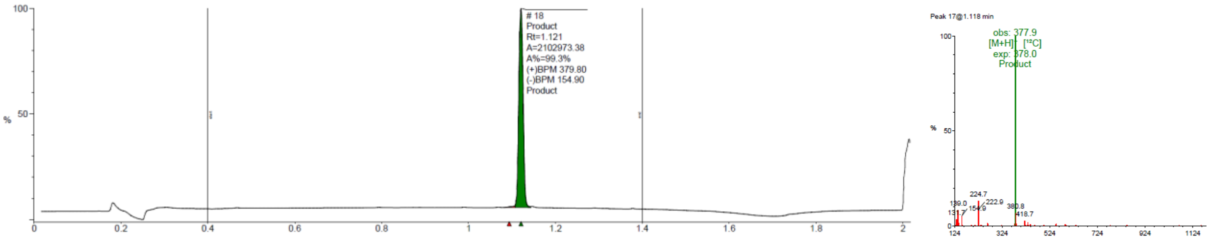

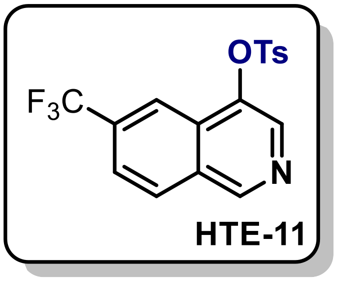

6-(Trifluoromethyl)isoquinolin-4-yl 4-methylbenzenesulfonate (HTE-11)

Quantity Obtained: 10.4 mg (28% isolated yield, 58% LCAP)

^{1}H NMR (400 MHz, DMSO-d_6) δ 9.48 (s, 1H), 8.49 - 8.44 (m, 2H), 7.98 (d, J = 8.8 Hz, 1H), 7.76 (d, J = 8.1 Hz, 3H), 7.41 (d, J = 8.1 Hz, 2H), 2.37 (s, 3H)

^{1}H NMR Spectra (with suppression):

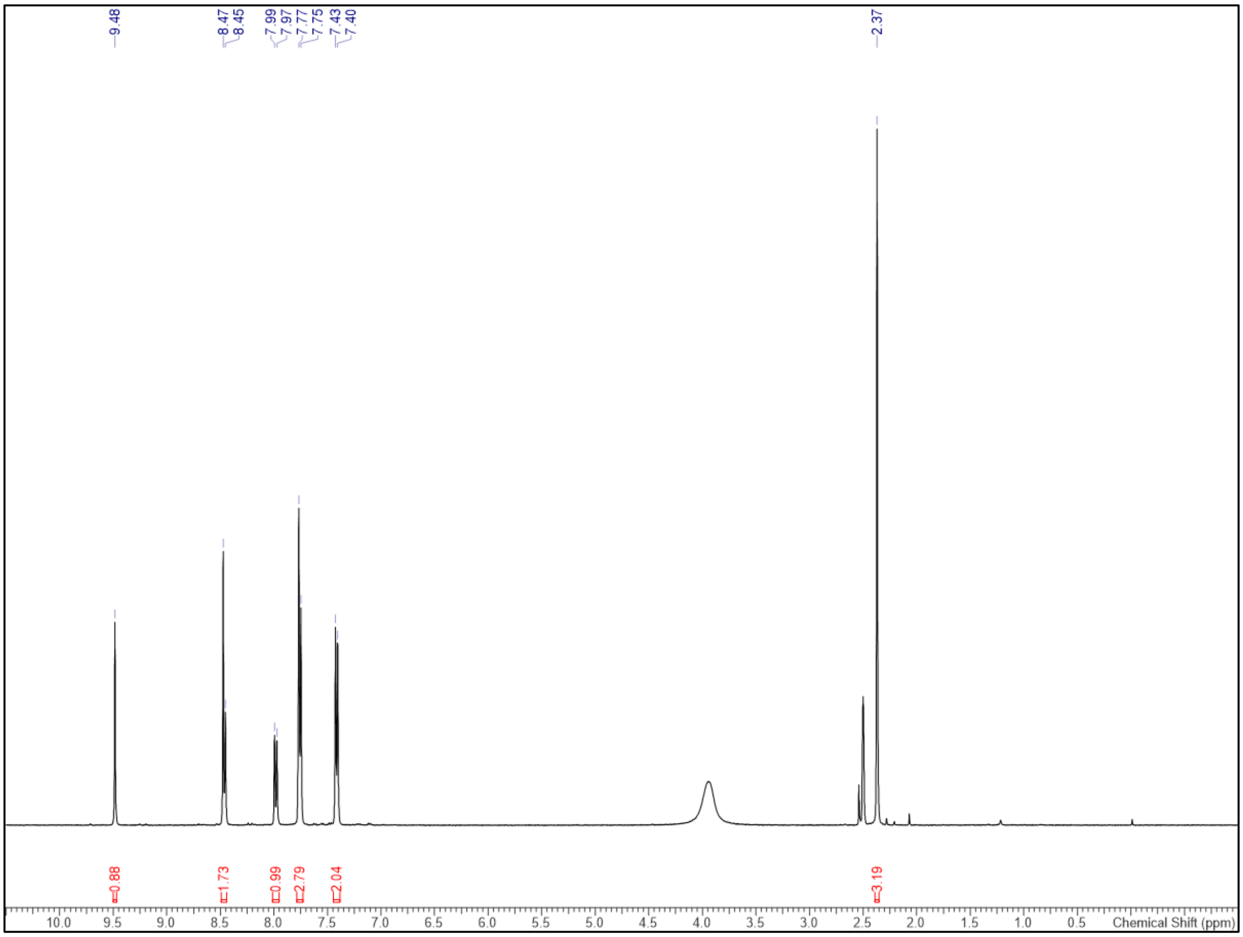

LCMS Data: Retention Time: 1.146 min; **MS ES+** ([M+H]$^+$): 368.0.

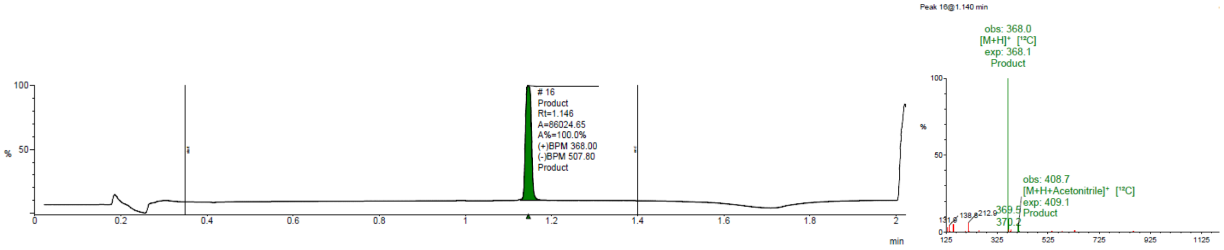

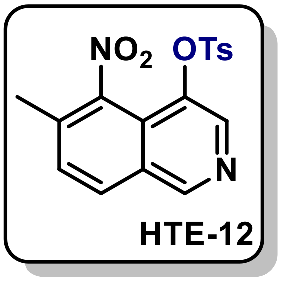

6-Methyl-5-nitroisoquinolin-4-yl 4-methylbenzenesulfonate (HTE-12, 3j)

Quantity Obtained: 4.0 mg (11% isolated yield, 40% LCAP)

^{1}H NMR (400 MHz, DMSO-d_6) δ 9.39 (s, 1H), 8.42 - 8.35 (m, 2H), 7.89 - 7.79 (m, 3H), 7.52 (d, *J* = 8.1 Hz, 2H), 2.44 (s, 3H), 2.43 (s, 3H)

^{1}H NMR Spectra (without suppression):

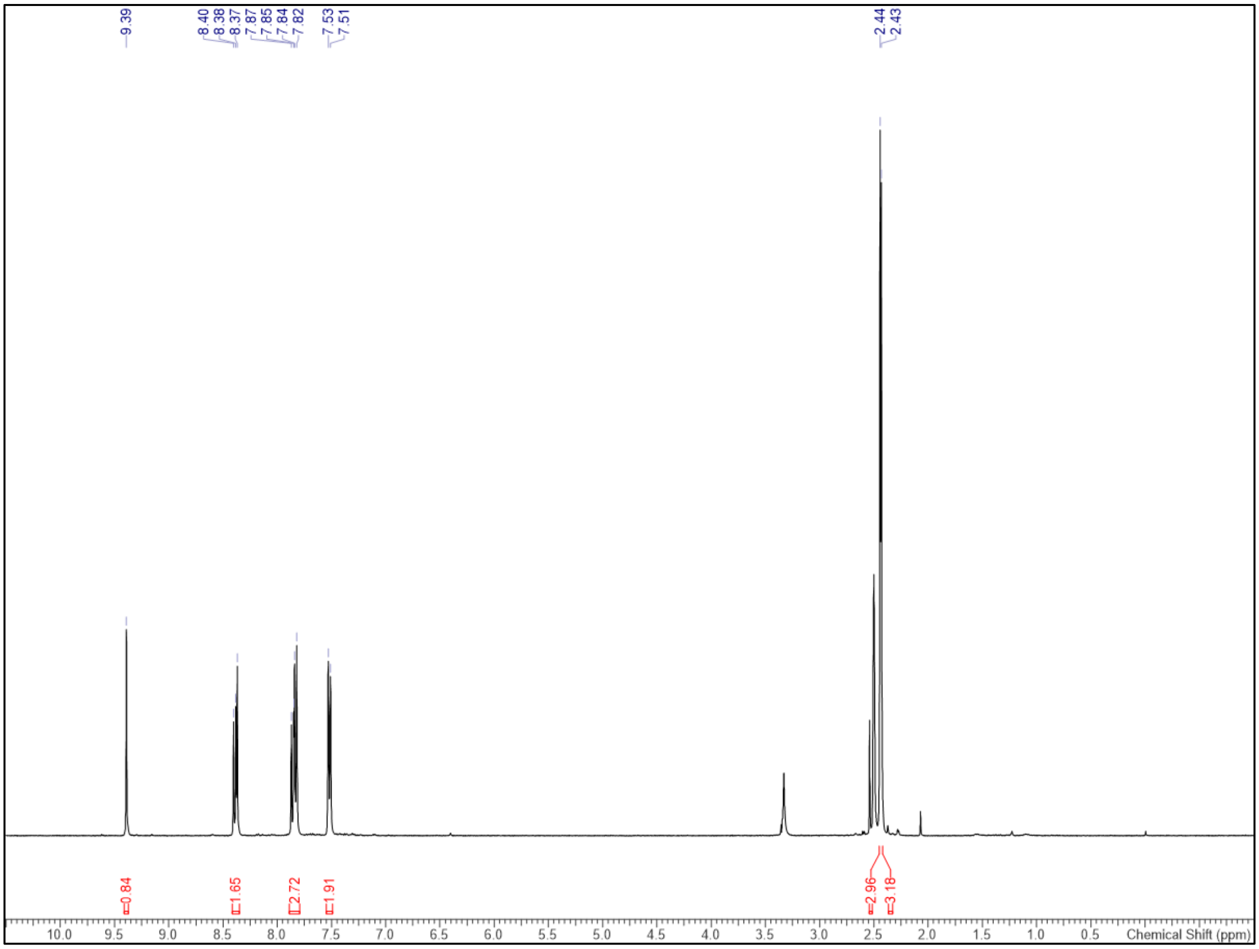

LCMS Data: Retention Time: 1.033 min; **MS ES+** ($[M+H]^+$): 358.9.

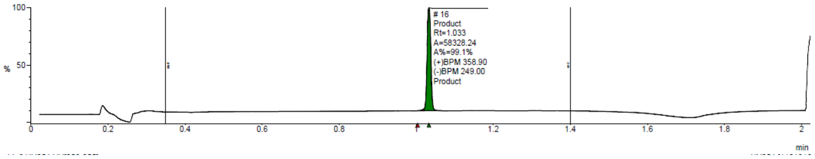

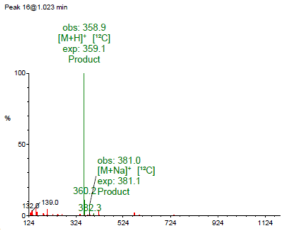

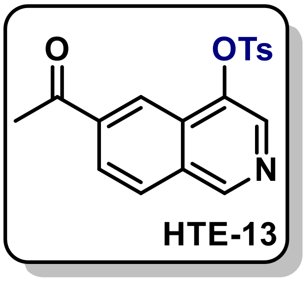

6-acetylisoquinolin-4-yl 4-methylbenzenesulfonate (HTE-13)

Quantity Obtained: 5.7 mg (17% isolated yield, 65% LCAP)

^{1}H NMR (400 MHz, DMSO-d_6) δ 9.41 (s, 1H), 8.38 (s, 1H), 8.32 (d, J = 8.6 Hz, 1H), 8.18 (s, 1H), 8.16 - 8.11 (m, 1H), 7.82 (d, J = 8.3 Hz, 2H), 7.44 (d, J = 8.1 Hz, 2H), 2.61 (s, 3H), 2.37 (s, 3H)

^{1}H NMR Spectra (with suppression):

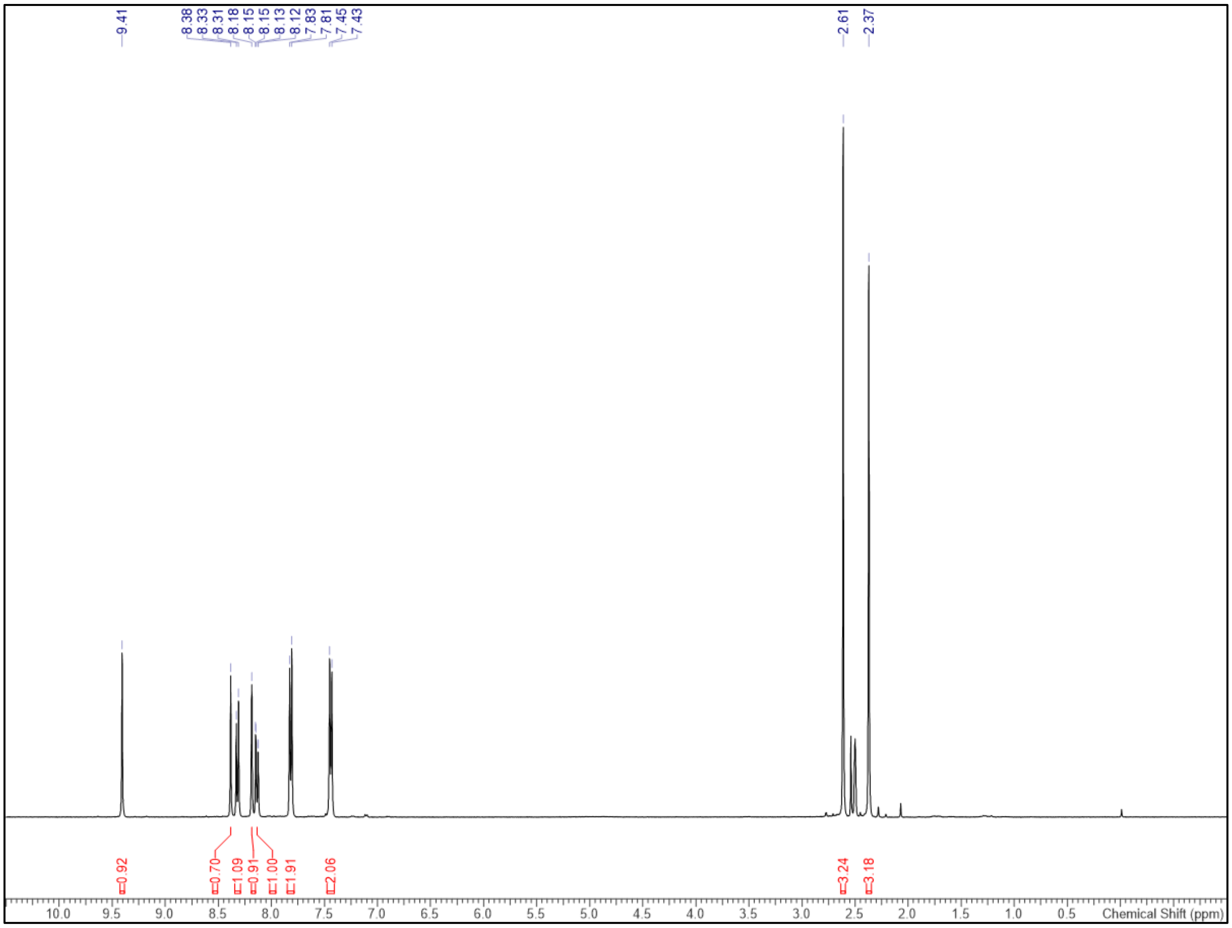

LCMS Data: Retention Time: 0.960 min; **MS ES+** ([M+H]$^+$): 342.2.

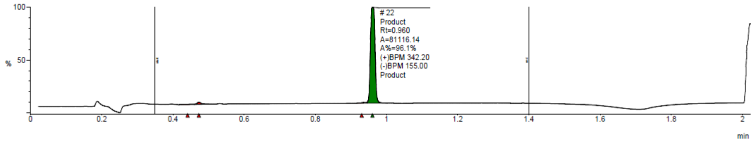

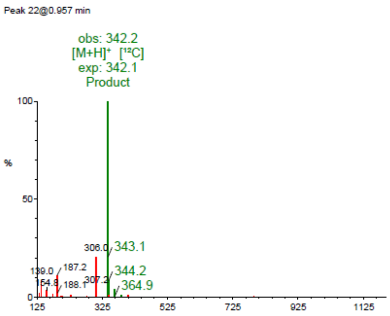

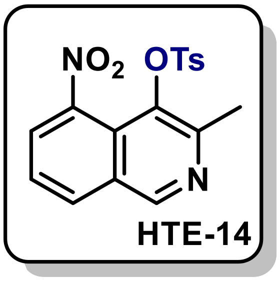

3-Methyl-5-nitroisoquinolin-4-yl 4-methylbenzenesulfonate (HTE-14)

Quantity Obtained: 4.3 mg (12% isolated yield, 47% LCAP)

^{1}H NMR (400 MHz, DMSO-d_6) δ 9.44 (s, 1H), 8.55 (d, J = 8.3 Hz, 1H), 8.41 (d, J = 7.6 Hz, 1H), 7.85 (t, J = 7.8 Hz, 1H), 7.66 (d, J = 8.3 Hz, 2H), 7.50 (d, J = 8.1 Hz, 2H), 2.45 (s, 3H), 2.23 (s, 3H)

^{1}H NMR Spectra (with suppression):

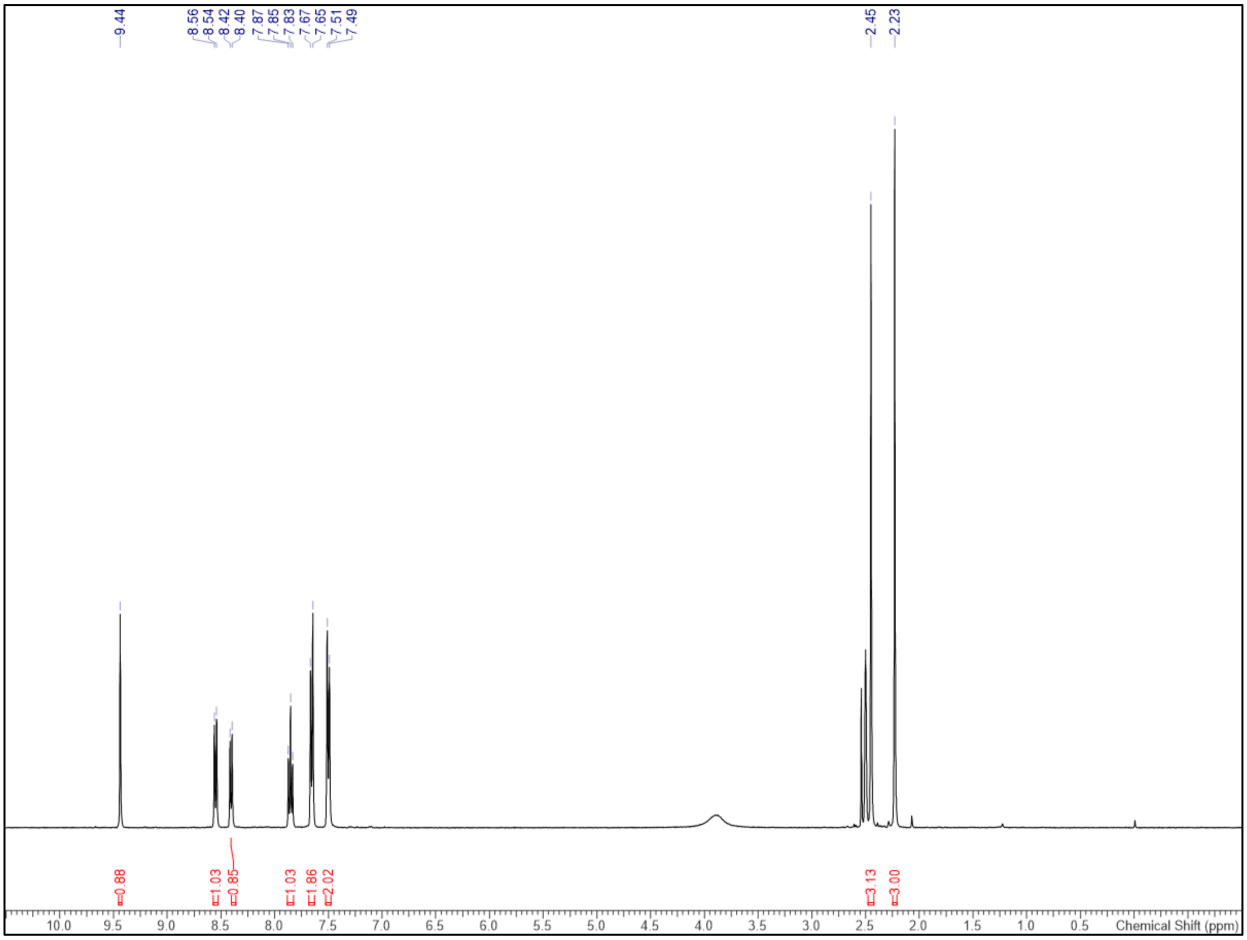

LCMS Data: Retention Time: 0.997 min; **MS ES+** ([M+H]$^+$): 358.8.

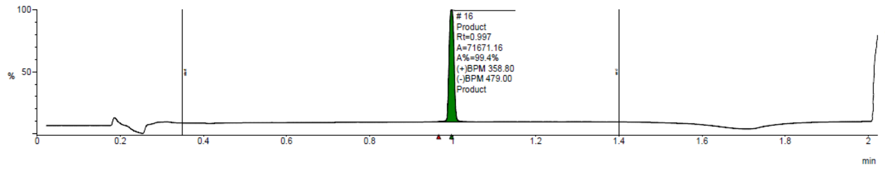

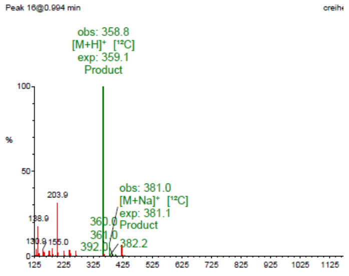

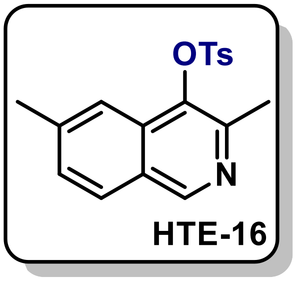

3,6-Dimethylisoquinolin-4-yl 4-methylbenzenesulfonate (HTE-16)

Quantity Obtained: 13.5 mg, 94% purity (41% isolated yield, 61% LCAP)

^{1}H NMR (400 MHz, DMSO-d_6) δ 9.17 (s, 1H), 8.05 (d, J = 8.3 Hz, 1H), 7.84 (d, J = 8.3 Hz, 2H), 7.54 - 7.47 (m, 3H), 7.15 (s, 1H), 2.45 (s, 3H), 2.43 (s, 3H), 2.32 (s, 3H)

^{1}H NMR Spectra (with suppression):

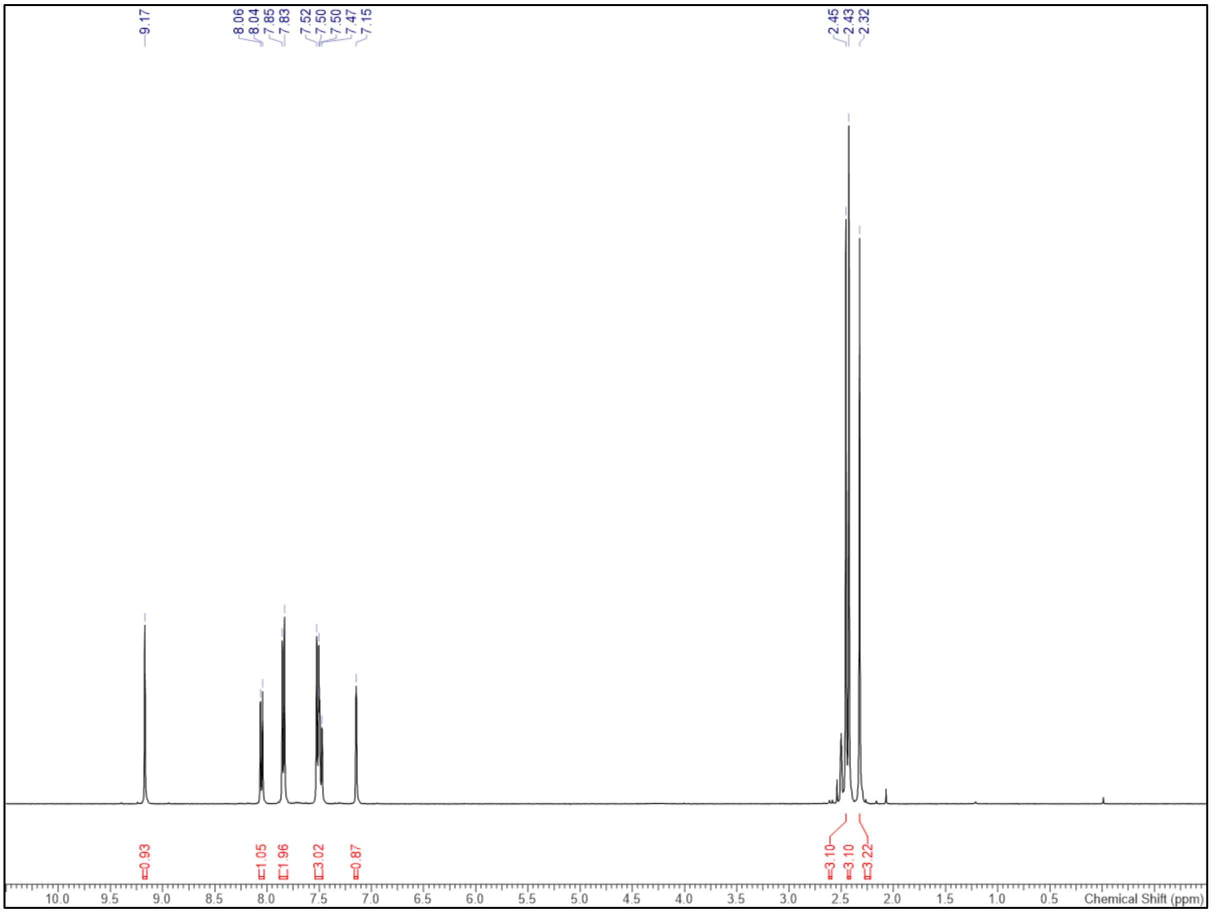

LCMS Data: Retention Time: 0.810 min; **MS ES+** ([M+H]$^+$): 328.2.

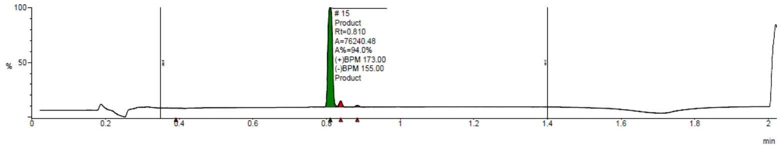

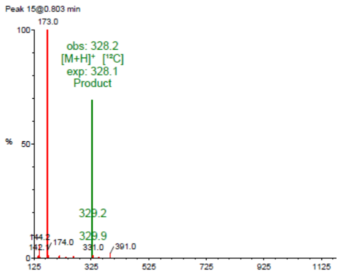

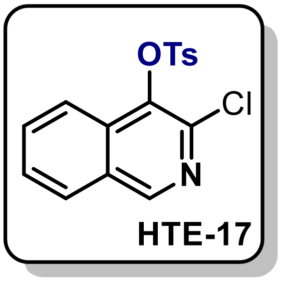

3-Chloroisoquinolin-4-yl 4-methylbenzenesulfonate (HTE-17)

Quantity Obtained: 3.1 mg (9% isolated yield, 7% LCAP)

^{1}H NMR (400 MHz, DMSO-d_6) δ 1H NMR (401 MHz, DMSO-d6) δ = 9.23 (s, 1H), 8.29 (d, J = 8.0 Hz, 1H), 7.95 - 7.87 (m, 3H), 7.85 - 7.78 (m, 2H), 7.55 (d, J = 8.3 Hz, 2H), 2.48 (s, 3H)

^{1}H NMR Spectra (with suppression):

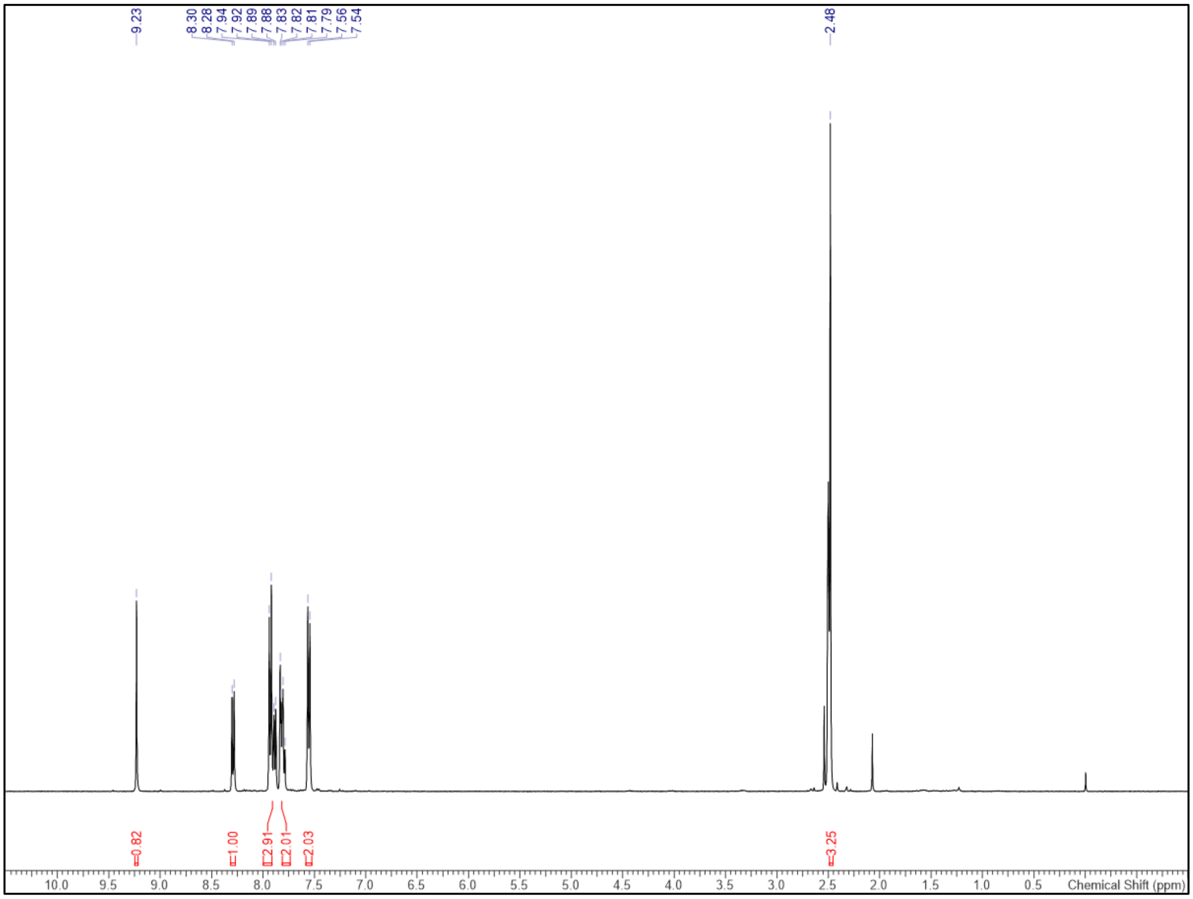

LCMS Data: Retention Time: 1.143 min; **MS ES+** ([M+H]$^+$): 333.9.

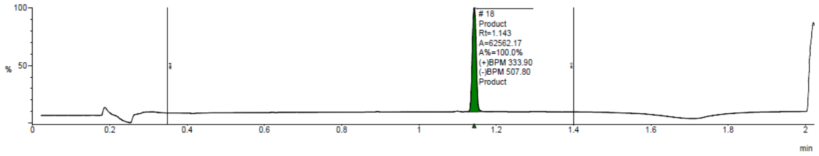

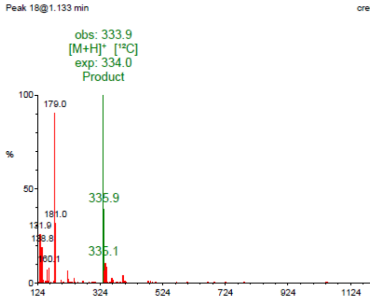

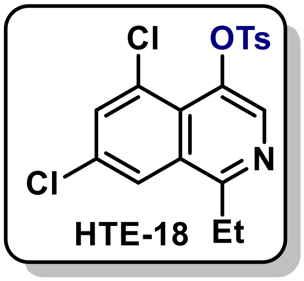

5,7-Dichloro-1-ethylisoquinolin-4-yl 4-methylbenzenesulfonate (HTE-18)

Quantity Obtained: 2.1 mg (5% isolated yield, 10% LCAP)

^{1}H NMR (400 MHz, DMSO-d_6) δ 8.40 (d, J = 1.5 Hz, 1H), 8.09 (d, J = 1.5 Hz, 1H), 7.91 (s, 1H), 7.77 (d, J = 8.1 Hz, 2H), 7.49 (d, J = 8.1 Hz, 2H), 3.29 (q, *J* = 7.4 Hz, 2H), 2.44 (s, 3H), 1.28 (t, J = 7.3 Hz, 3H)

^{1}H NMR Spectra (with suppression):

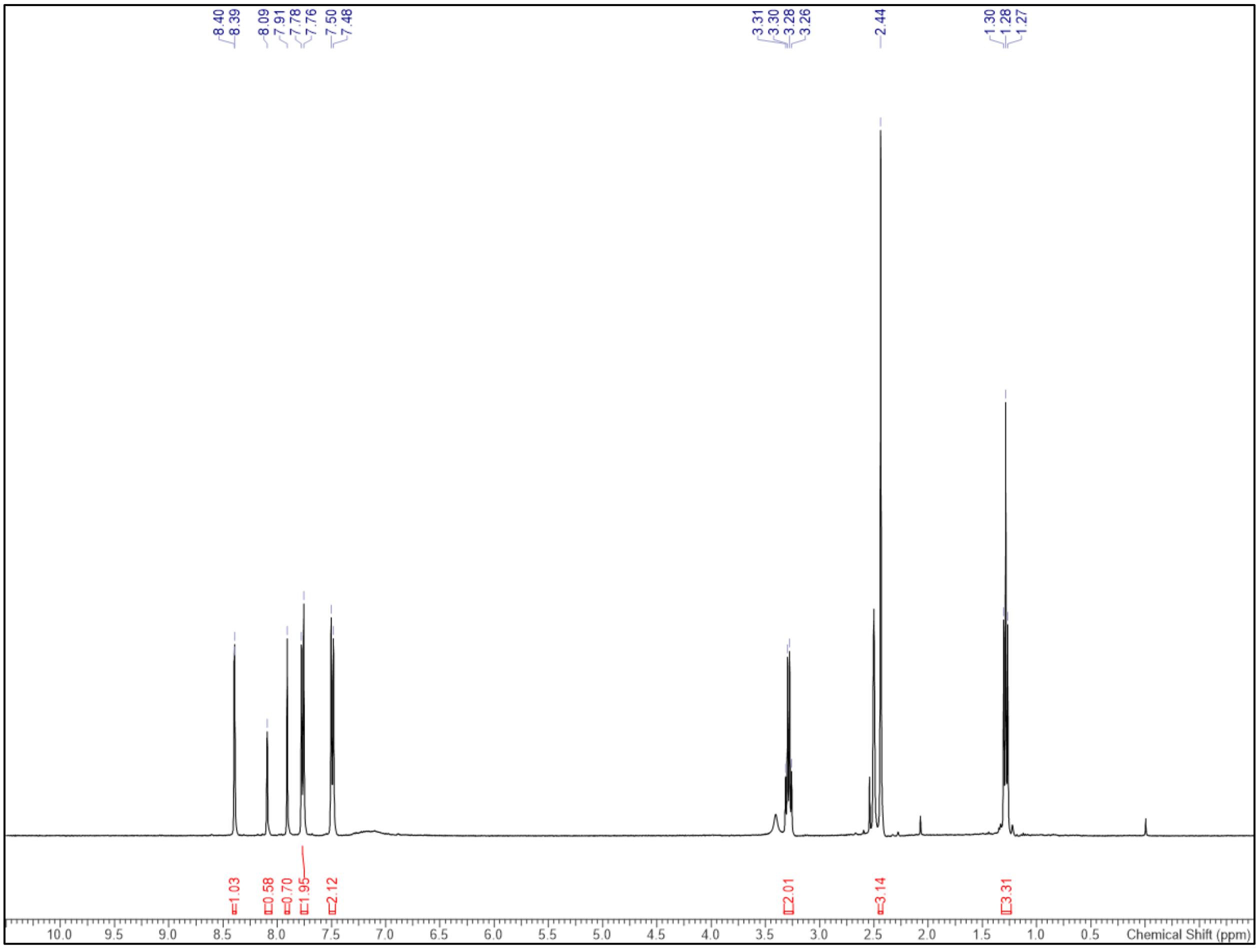

LCMS Data: Retention Time: 1.307 min; **MS ES+** ([M+H]$^+$): 395.9, 398.0

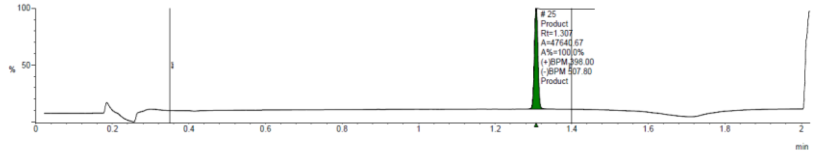

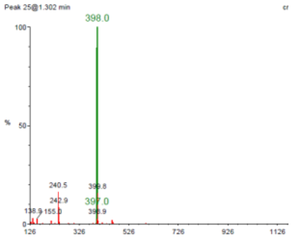

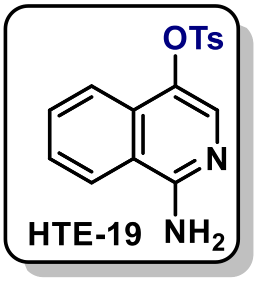

1-Aminoisoquinolin-4-yl 4-methylbenzenesulfonate (HTE-19)

Quantity Obtained: 4.3 mg (12% isolated yield, 42% LCAP)

^{1}H NMR (400 MHz, DMSO-d_6) δ 8.70 (br s, 2H), 8.46 (d, J = 8.3 Hz, 1H), 7.86 (br d, J = 8.3 Hz, 3H), 7.74 (t, J = 7.1 Hz, 1H), 7.69 (d, J = 7.8 Hz, 1H), 7.53 (s, 1H), 7.46 (d, J = 7.8 Hz, 2H), 2.40 (s, 3H)

^{1}H NMR Spectra (without suppression):

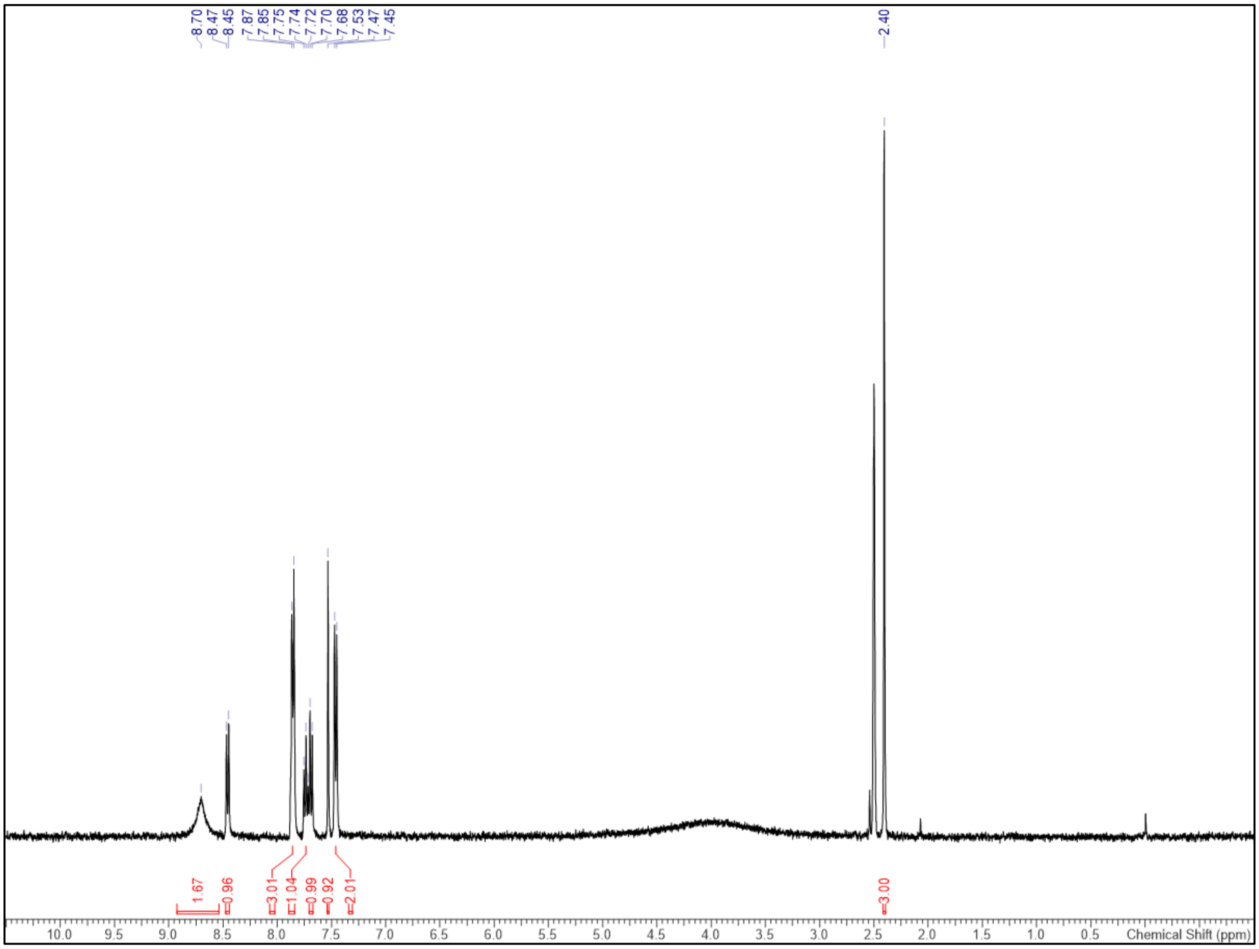

LCMS Data: Retention Time: 0.674 min; **MS ES+** ([M+H]$^+$): 315.1

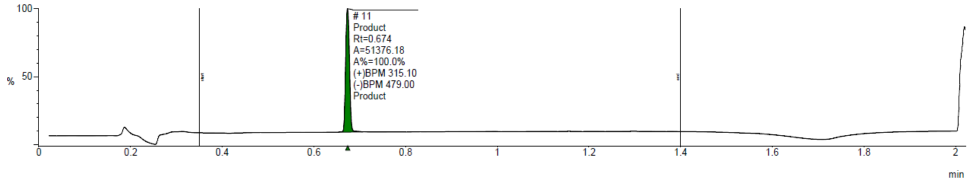

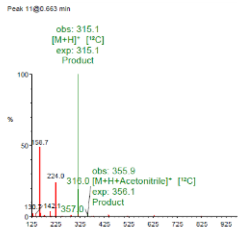

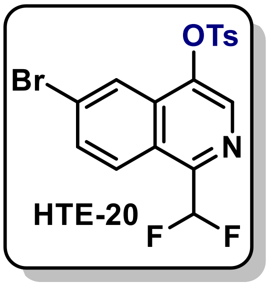

6-Bromo-1-(difluoromethyl)isoquinolin-4-yl 4-methylbenzenesulfonate (HTE-20)

Quantity Obtained: 1.6 mg (4% isolated yield, 15% LCAP)

^{1}H NMR (400 MHz, DMSO-d_6) δ 8.42 (s, 1H), 8.32 (br d, J = 9.0 Hz, 1H), 7.99 (dd, J = 1.7, 9.0 Hz, 1H), 7.83 (d, J = 8.3 Hz, 2H), 7.81 - 7.77 (m, 1H), 7.47 (d, *J* = 8.3 Hz, 2H), 7.45 (t, *J* = 53.3 Hz, 1H), 2.42 (s, 3H)

^{1}H NMR Spectra (with suppression):

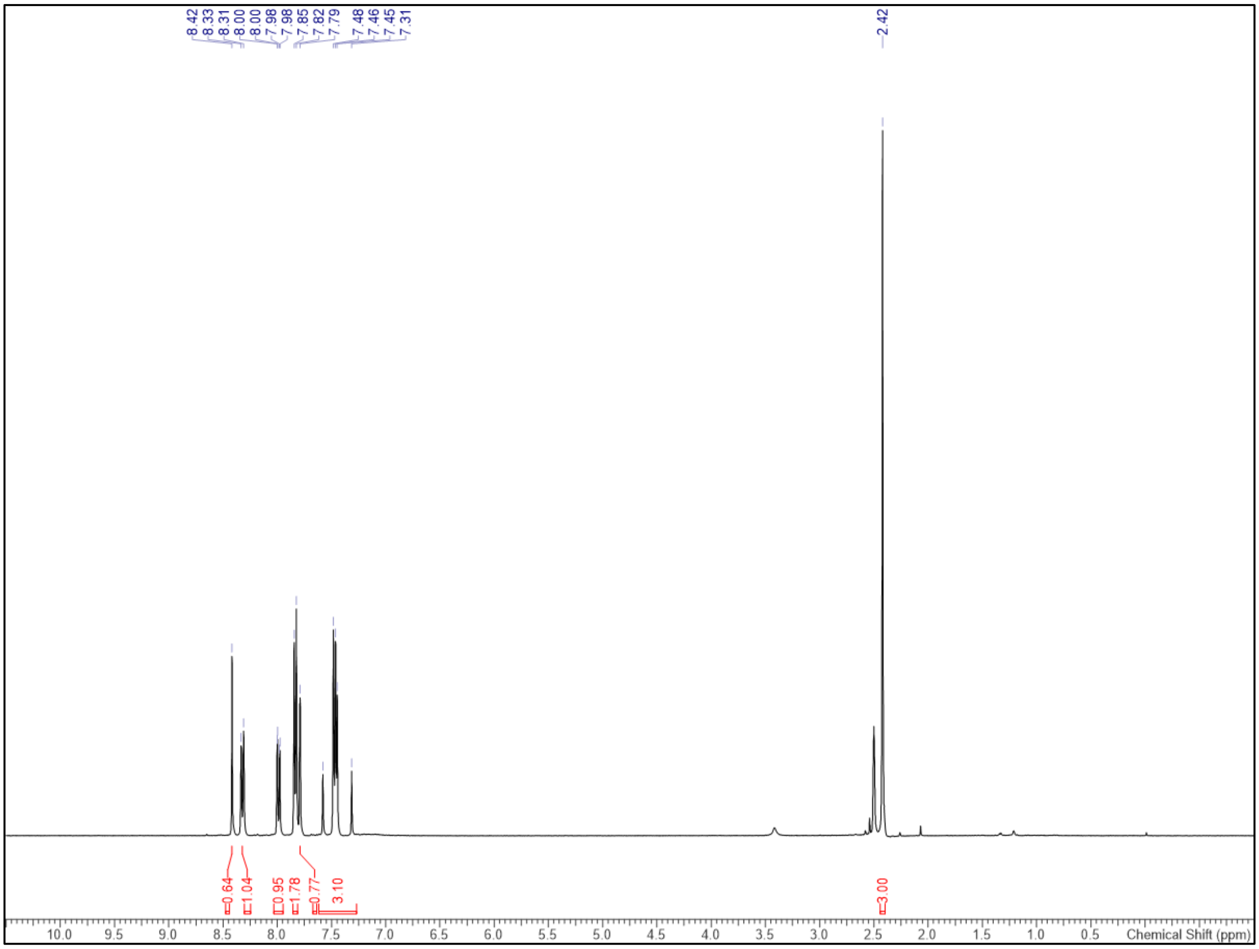

LCMS Data: Retention Time: 1.277 min; **MS ES+** ([M+H]$^+$): 427.8, 429.7

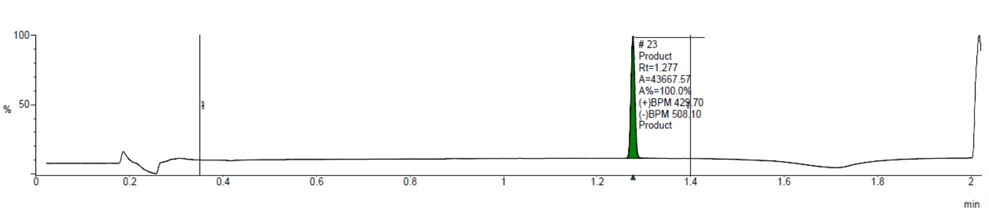

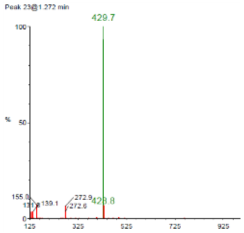

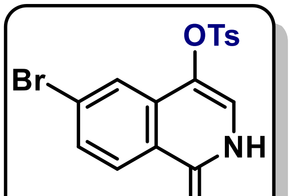

HTE-22

6-Bromo-1-oxo-1,2-dihydroisoquinolin-4-yl 4-methylbenzenesulfonate (HTE-22)

Quantity Obtained: 2.7 mg (7% isolated yield, 9% LCAP)

^{1}H NMR (400 MHz, DMSO-d_6) δ 1H NMR (401 MHz, DMSO-d6) δ = 11.60 - 11.47 (m, 1H), 8.04 (d, J = 8.6 Hz, 1H), 7.82 (d, J = 8.3 Hz, 2H), 7.65 (dd, J = 1.7, 8.6 Hz, 1H), 7.45 (d, J = 8.1 Hz, 2H), 7.29 (d, J = 1.7 Hz, 1H), 7.14 (br d, J = 5.6 Hz, 1H), 2.40 (s, 3H)

^{1}H NMR Spectra (with suppression):

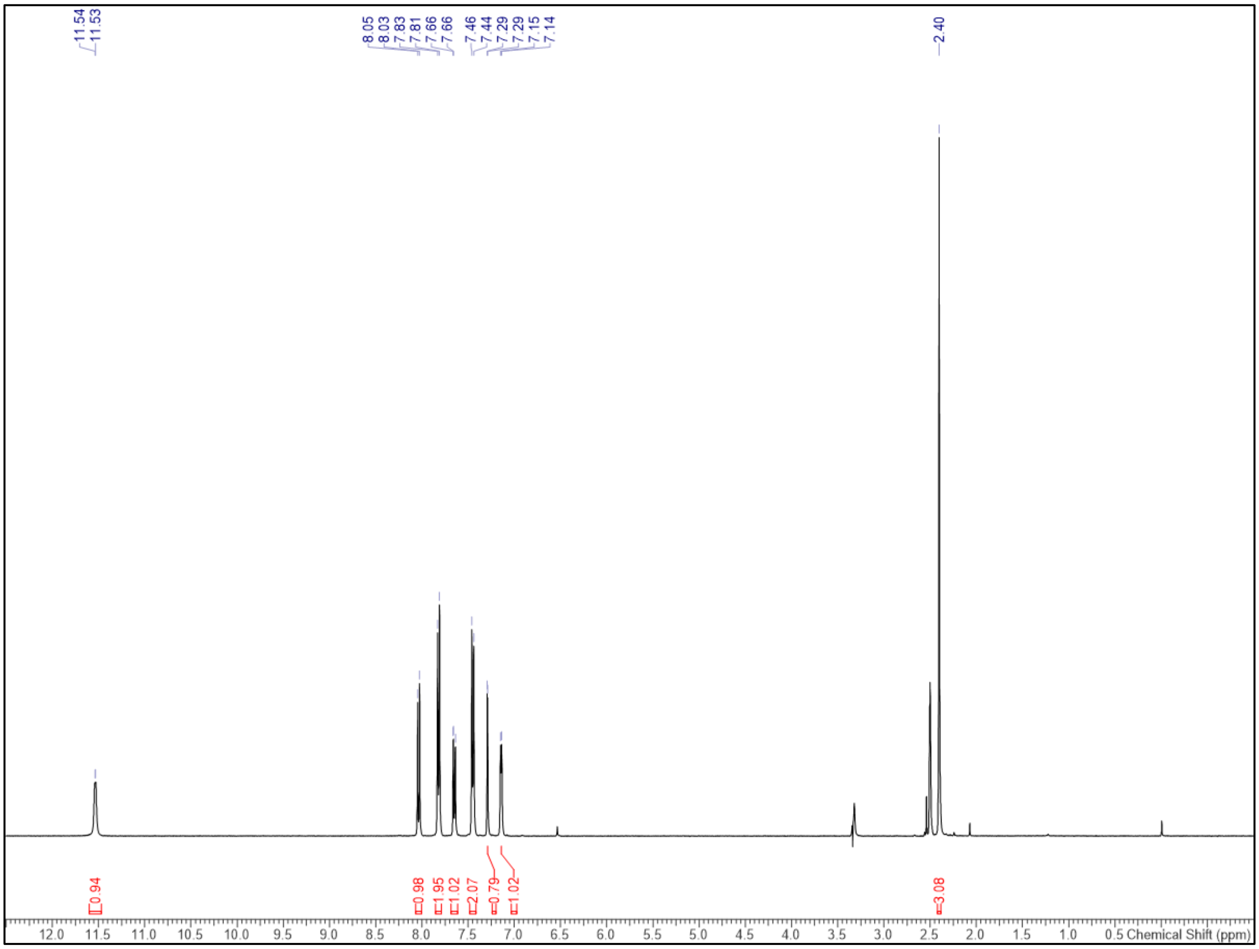

LCMS Data: Retention Time: 1.018 min; **MS ES+** ([M+H]$^+$): 394.0, 395.8

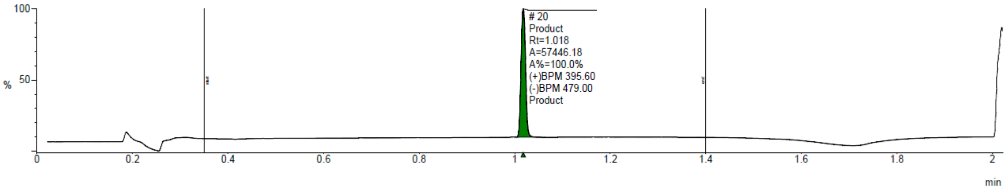

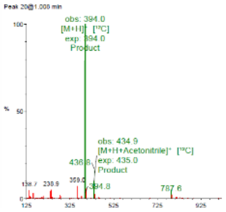

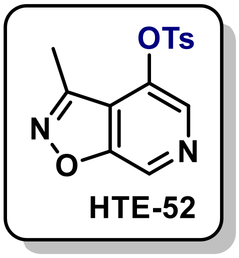

3-methylisoxazolo[5,4-c]pyridin-4-yl 4-methylbenzenesulfonate (HTE-52)

Quantity Obtained: 1.1 mg (4% isolated yield, 25% LCAP)

^{1}H NMR (400 MHz, DMSO-d_6) δ 9.21 (s, 1H), 8.04 (s, 1H), 7.84 (d, J = 8.3 Hz, 2H), 7.54 (d, J = 8.1 Hz, 2H), 2.51 (s, 3H), 2.46 (s, 3H)

^{1}H NMR Spectra (with suppression):

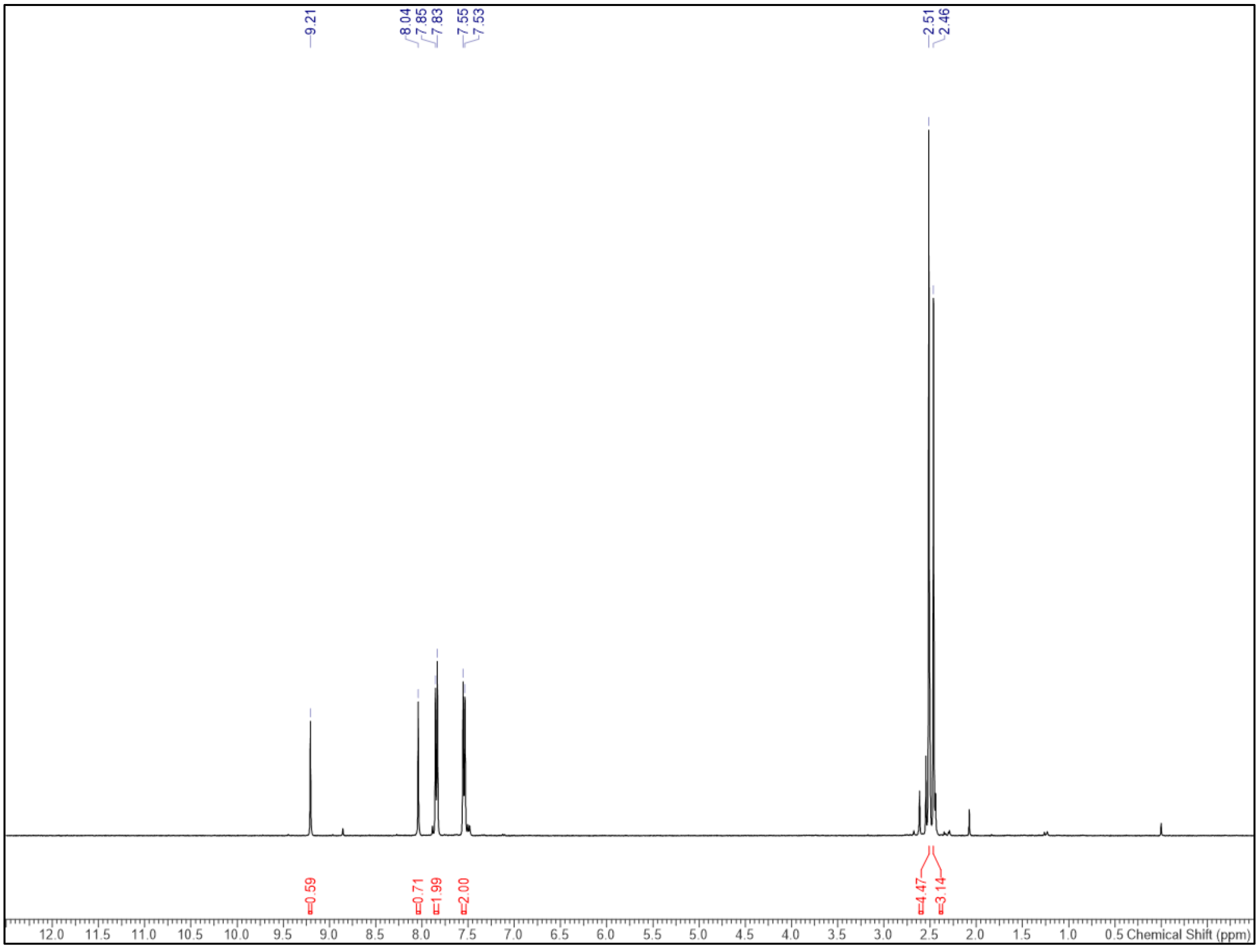

LCMS Data: Retention Time: 1.016 min; **MS ES+** ([M+H]$^+$): 304.9

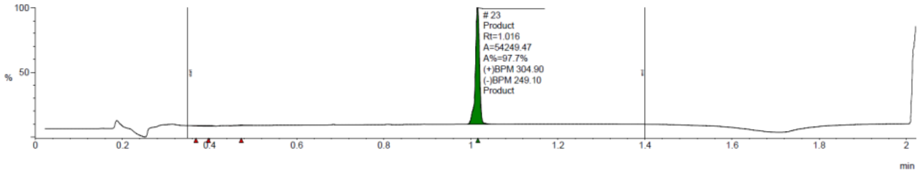

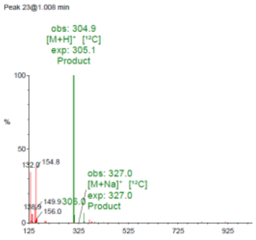

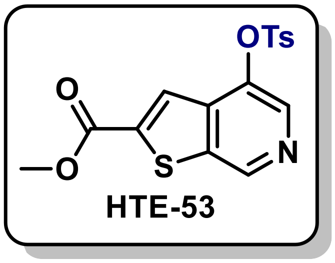

Methyl 4-(tosyloxy)thieno[2,3-c]pyridine-2-carboxylate (HTE-53)

Quantity Obtained: 6.5 mg (18% isolated yield, 12% LCAP)

^{1}H NMR (400 MHz, DMSO-d_6) δ 9.34 (s, 1H), 8.20 (s, 1H), 7.80 (d, J = 8.3 Hz, 2H), 7.62 (s, 1H), 7.48 (br d, J = 8.1 Hz, 2H), 3.93 (s, 3H), 2.42 (s, 3H)

^{1}H NMR Spectra (without suppression):

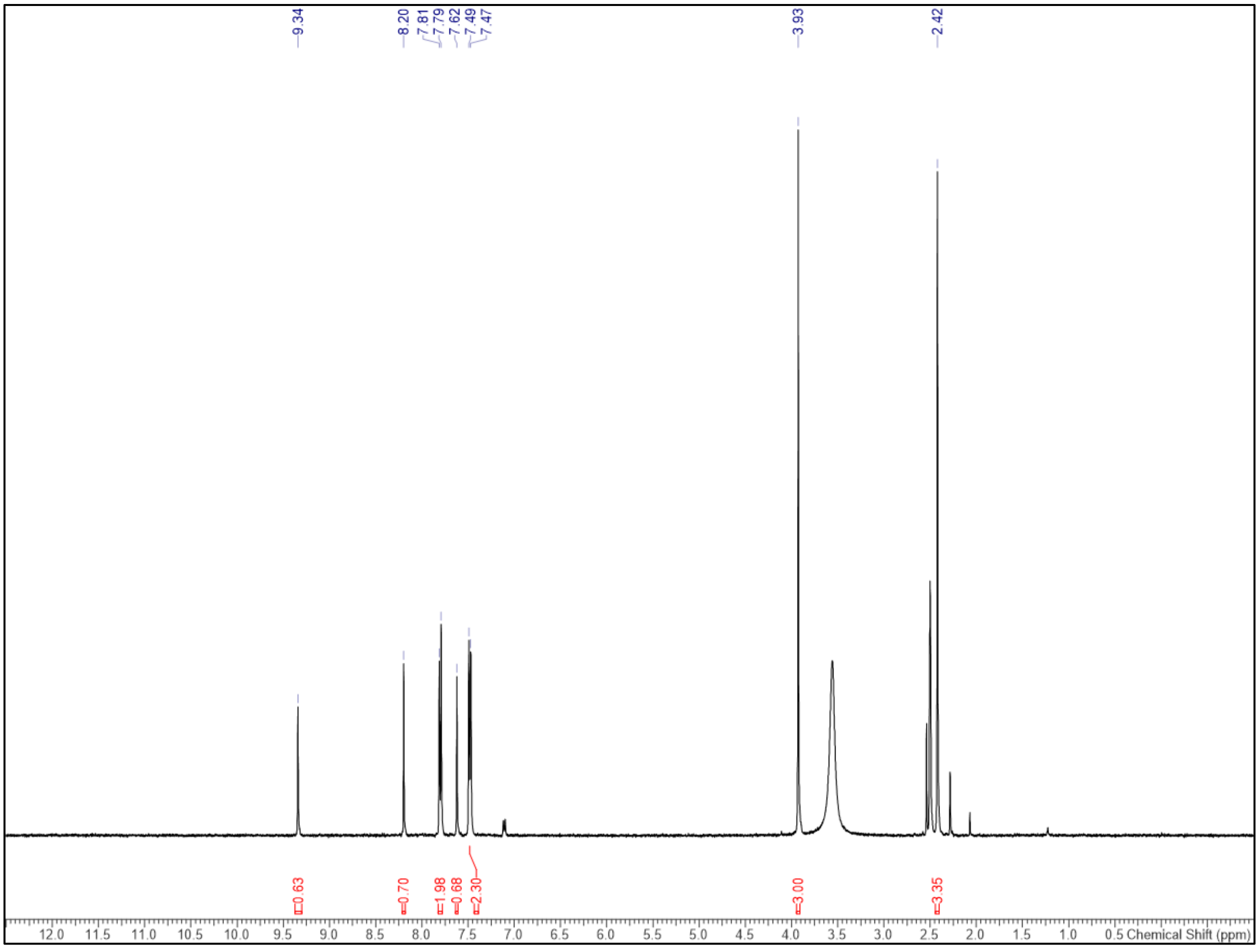

LCMS Data: Retention Time: 1.033 min; **MS ES+** ([M+H]$^+$): 363.9

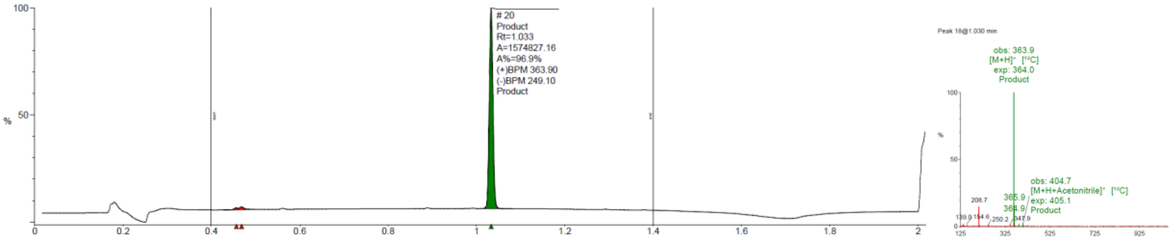

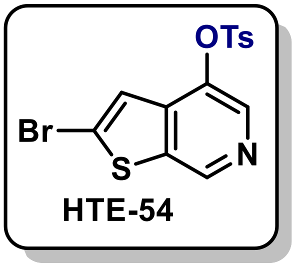

2-Bromothieno[2,3-c]pyridin-4-yl 4-methylbenzenesulfonate (HTE-54)

Quantity Obtained: 1.1 mg, 83% purity (2% isolated yield, 3% LCAP)

^{1}H NMR (400 MHz, DMSO-d_6) δ 9.14 (s, 1H), 8.11 (s, 1H), 7.79 (d, J = 8.3 Hz, 2H), 7.47 (d, J = 8.1 Hz, 2H), 7.26 (s, 1H), 2.43 (s, 3H)

^{1}H NMR Spectra (with suppression):

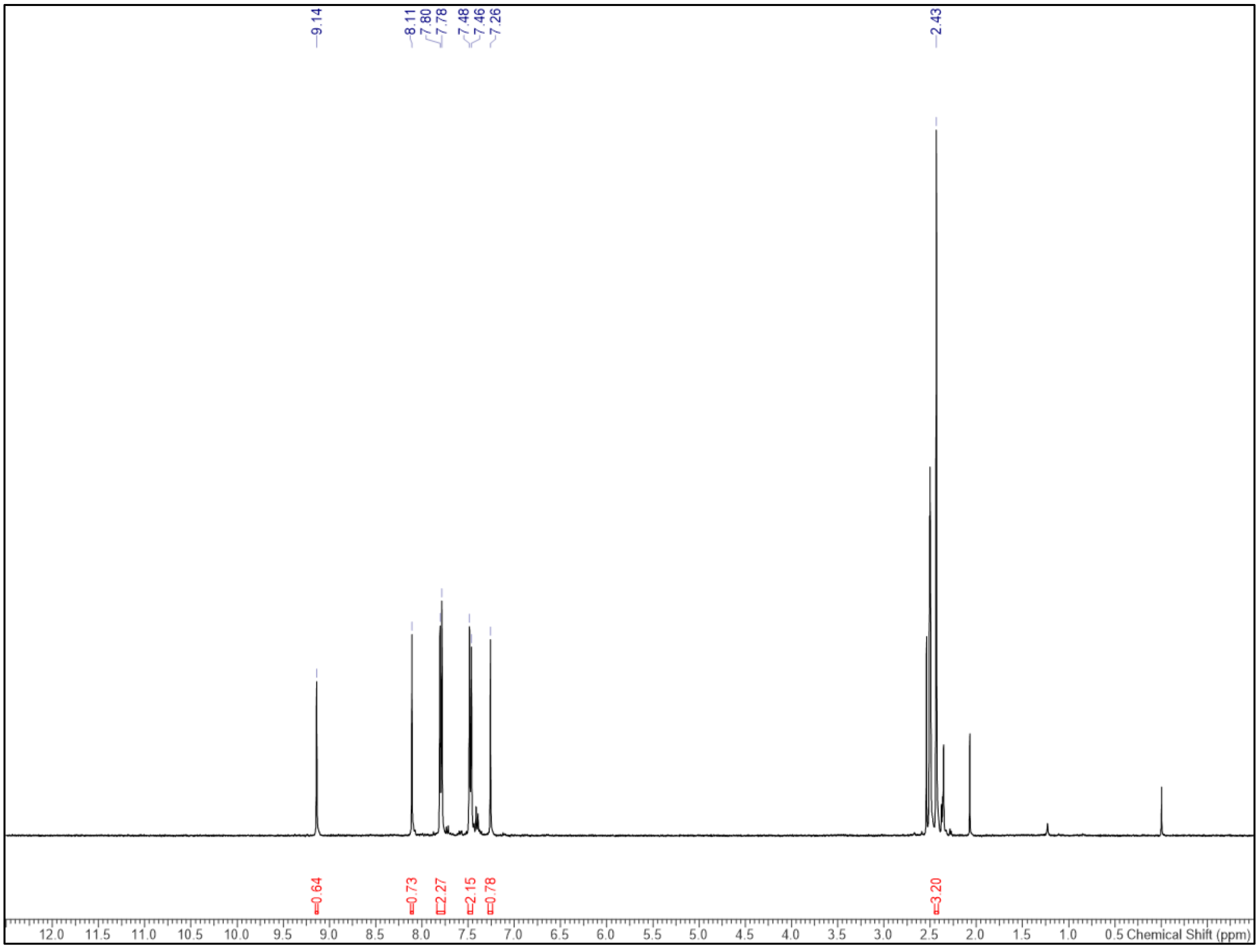

LCMS Data: Retention Time: 1.076 min; **MS ES+** ([M+H]$^+$): 383.7, 385.8

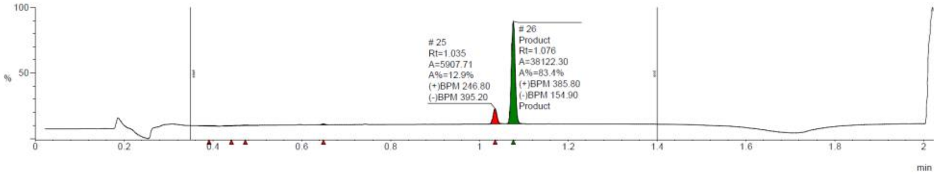

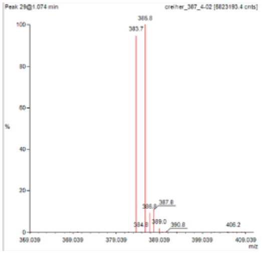

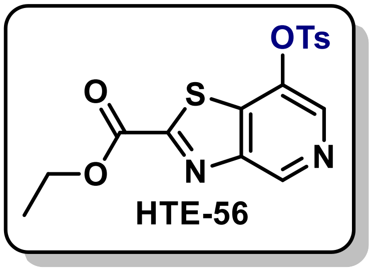

Ethyl 7-(tosyloxy)thiazolo[4,5-c]pyridine-2-carboxylate (HTE-56, 3m)

Quantity Obtained: 0.3 mg (1% isolated yield, 35% LCAP)

^{1}H NMR (400 MHz, DMSO-d_6) δ 9.49 (s, 1H), 8.38 (s, 1H), 7.82 (d, J = 8.3 Hz, 2H), 7.50 (br d, J = 8.1 Hz, 2H), 4.46 (q, J = 7.1 Hz, 2H), 2.43 (s, 3H), 2.36 (br d, J = 4.2 Hz, 1H), 1.37 (t, J = 7.1 Hz, 3H)

^{1}H NMR Spectra (with suppression):

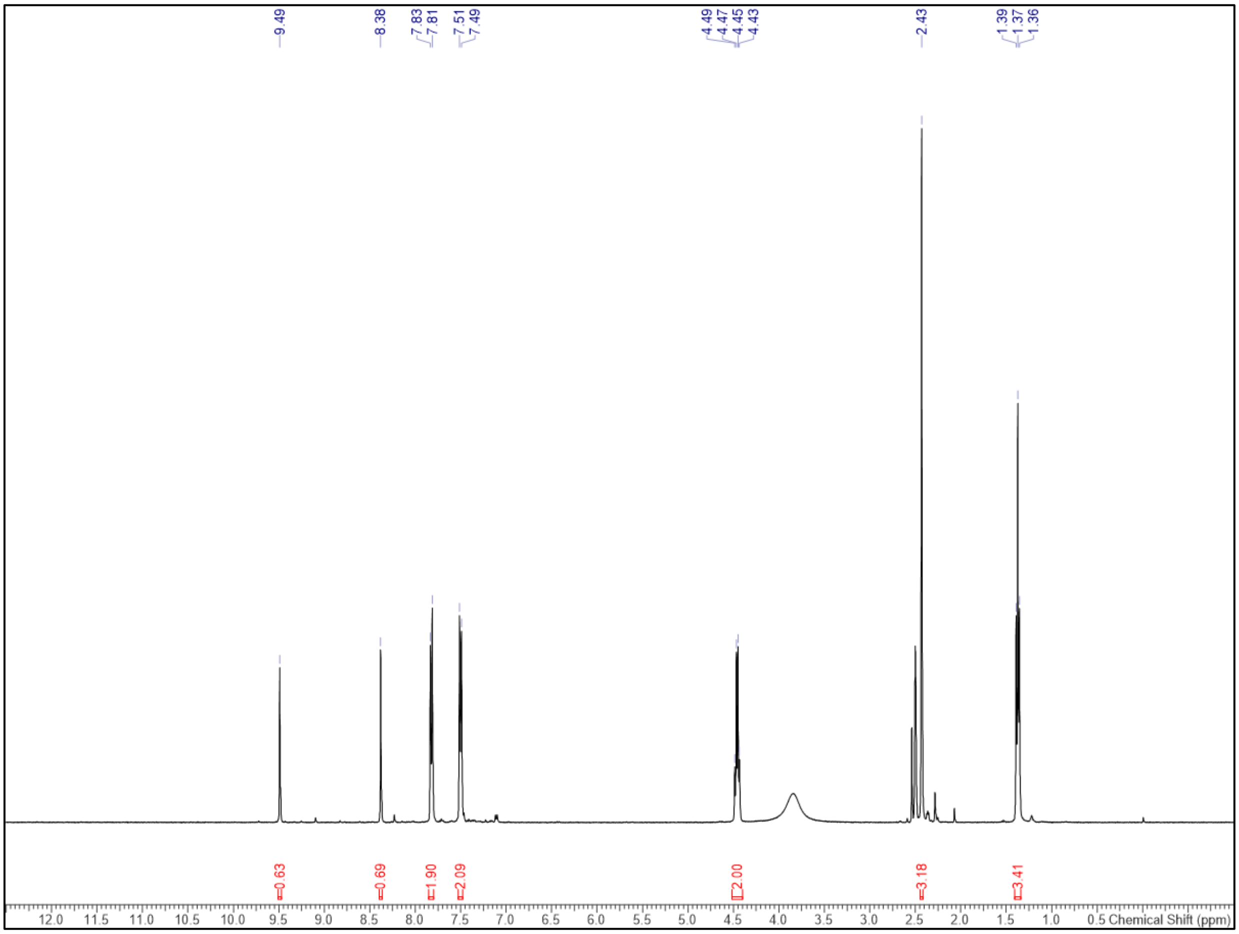

LCMS Data: Retention Time: 1.060 min; **MS ES+** ([M+H]$^+$): 379.0

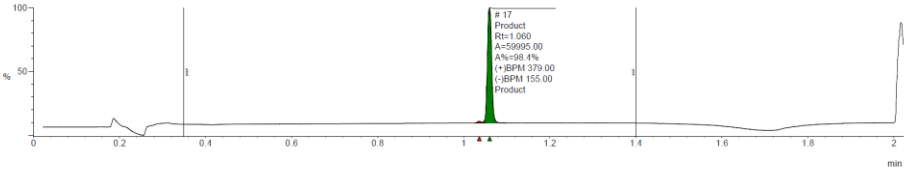

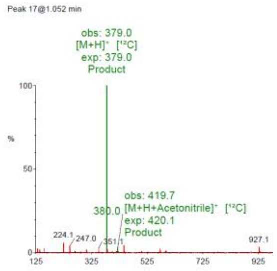

IV. General Procedures and Characterization Data

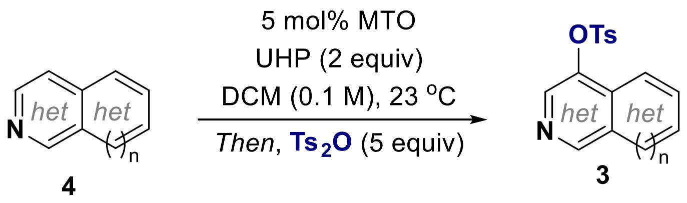

Weigh substrate **4** (0.1 mmol, 1 equiv) in a 8 ml capped vial equipped with a magnetic stirring bar. Dissolve in dry DCM (0.1 M) and add MTO (5 mol%) followed by UHP (2 equiv). Stir at 23 °C for 16 h and analyze the crude by UPLC-MS (5 μl of sample diluted in 195 μl of a 0.02 M solution of 4,4'-di-*tert*-butyl-1,1'-biphenyl in acetonitrile). Add Ts_2O (5 equiv), keep stirring for 2 h and analyze the crude by UPLC-MS again. Reaction at 0.5 mmol scale: dilute the crude with DCM and quench with saturated aqueous $NaHCO_3$. Separate phases, concentrate under reduced pressure and directly purify the desired product by CombiFlash silica gel chromatography (DCM injection).

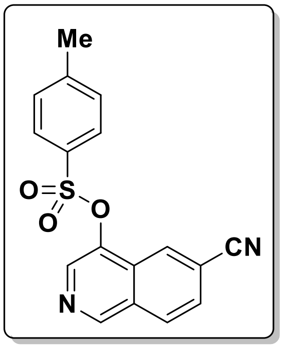

Compound 3b. Separation with heptane/85:15 ethyl acetate and 7N ammonia in methanol (0% to 100%) leads to 70% yield of product **3b** as yellow crystals (113 mg, 0.35 mmol). *Note: add preactivated 4Å MS prior to Ts_2O and keep stirring for 16 h during the addition step*. ^{1}H NMR (400 MHz, chloroform-*d*) δ ppm 2.49 (s, 3 H), 7.38 (d, *J*=8.14 Hz, 2 H), 7.71 - 7.86 (m, 3 H), 8.11 (d, *J*=8.36 Hz, 1 H), 8.16 - 8.19 (m, 1 H), 8.36 (s, 1 H), 9.24 (s, 1 H). ^{13}C NMR (101 MHz, chloroform-*d*) δ ppm 21.73, 114.74, 117.57, 127.39, 128.55, 128.60, 128.69, 129.62, 129.78, 130.26, 131.57, 138.00, 141.20, 146.65, 151.06. ^{1}H COSY, ^{13}C HSQC and ^{13}C HMBC included to confirm regioselectivity. HRMS (ESI$^+$) m/z calculated for $C_{17}H_{12}N_2O_3S$ $[M+H]^+$ 325.0641, found 325.0640.

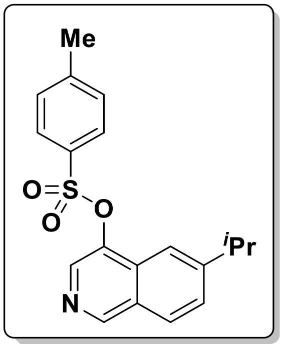

Compound 3c. Separation with heptane/85:15 ethyl acetate and 7N ammonia in methanol (0% to 100%) leads to 64% yield of product **3c** as an orange oil (110 mg, 0.32 mmol). ^{1}H NMR (400 MHz, chloroform-*d*) δ ppm 1.26 (d, *J*=7.04 Hz, 6 H), 2.42 (s, 3 H), 3.00 - 3.07 (m, 1 H), 7.29 (d, *J*=8.14 Hz, 2 H), 7.51 (dd, *J*=8.47, 1.43 Hz, 1 H), 7.62 (s, 1 H), 7.77 (d, *J*=8.36 Hz, 2 H), 7.90 (d, *J*=8.58 Hz, 1 H), 8.18 (s, 1 H), 9.07 (s, 1 H). ^{13}C NMR (101 MHz, chloroform-*d*) δ ppm 21.64, 23.42, 34.66, 117.10, 127.24, 128.06, 128.52, 128.57, 129.94, 130.54, 132.34, 136.11, 141.95, 145.74, 150.59, 152.43. ^{1}H COSY, ^{13}C HSQC and ^{13}C HMBC included to confirm regioselectivity. HRMS (ESI^{+}) m/z calculated for $C_{19}H_{19}NO_3S$ $[M+H]^{+}$ 342.1158, found 342.1156.

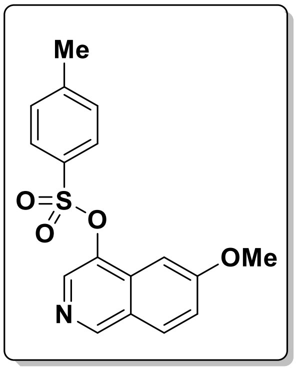

Compound 3d. Separation with heptane/85:15 ethyl acetate and 7N ammonia in methanol (0% to 100%) leads to 23% yield of product **3d** as a white powder (14.5 mg, 0.045 mmol). *Note: reaction at 0.2 mmol scale. Add water (3 equiv) for the addition step and quench after 30 min.* ^{1}H NMR (400 MHz, chloroform-*d*) δ ppm 2.46 (s, 3 H), 3.89 (s, 3 H), 7.16 (d, *J*=2.42 Hz, 1 H), 7.24 (dd, *J*=9.02, 2.42 Hz, 1 H), 7.34 (d, *J*=8.14 Hz, 2 H), 7.78 - 7.82 (m, 2 H), 7.87 (d, *J*=9.02 Hz, 1 H), 8.03 (s, 1 H), 8.98 (s, 1 H). ^{13}C NMR (101 MHz, chloroform-*d*) δ ppm 21.73, 55.62, 98.80, 121.62, 125.61, 128.61, 129.17, 130.05, 132.31, 132.65, 136.31, 141.65, 145.95, 149.98, 161.87. ^{1}H COSY, ^{13}C HSQC and ^{13}C HMBC included to confirm regioselectivity. HRMS (ESI^{+}) m/z calculated for $C_{17}H_{15}NO_4S$ $[M+H]^{+}$ 330.0894, found 330.0789. *Note: significant hydration observed as by-product.*

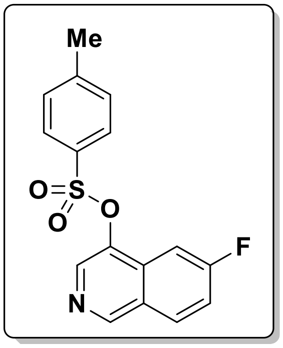

Compound 3e. Separation with heptane/85:15 ethyl acetate and 7N ammonia in methanol (0% to 100%) leads to 65% yield of product **3e** as beige powder (105 mg, 0.33 mmol). ^{1}H NMR (400 MHz, chloroform-*d*) δ ppm 2.46 (s, 3 H), 7.33 - 7.42 (m, 3 H), 7.55 (dd, *J*=9.41, 2.40 Hz, 1 H), 7.78 - 7.83 (m, 2 H), 8.02 (dd, *J*=9.04, 5.28 Hz, 1 H), 8.15 (s, 1 H), 9.11 (s, 1 H). ^{13}C NMR (101 MHz, chloroform-*d*) δ ppm 21.71, 105.43 (d, *J* (^{13}C–^{19}F) =23.65 Hz), 118.88 (d, *J* (^{13}C–^{19}F) =25.18 Hz), 126.94, 128.56, 130.08, 130.50 (d, *J* (^{13}C–^{19}F) =9.92 Hz), 131.95, 132.18 (d, *J* (^{13}C–^{19}F) =10.68 Hz), 136.80, 141.65 (d, *J* (^{13}C–^{19}F) =6.10 Hz), 146.14, 150.66, 163.43 (d, *J* (^{13}C–^{19}F) =235.74 Hz). ^{19}F NMR (376 MHz, chloroform-*d*) δ ppm -103.96 (s). ^{1}H COSY, ^{13}C HSQC and ^{13}C HMBC included to confirm regioselectivity. HRMS (ESI^{+}) m/z calculated for $C_{16}H_{12}FNO_3S$ [M+H]$^{+}$ 318.0595, found 318.0588.

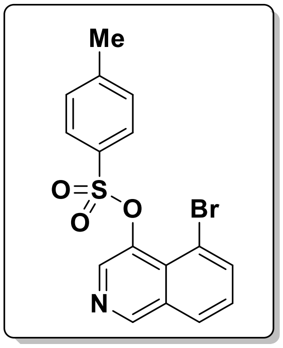

Compound 3f. Separation with heptane/85:15 ethyl acetate and 7N ammonia in methanol (0% to 100%) leads to 63% yield of product **3f** as yellow crystals (47.7 mg, 0.13 mmol). *Note: reaction at 0.2 mmol scale.* ^{1}H NMR (400 MHz, chloroform-*d*) δ ppm 2.46 (s, 3 H), 7.35 (d, *J*=7.92 Hz, 2 H), 7.47 (t, *J*=7.81 Hz, 1 H), 7.79 - 7.84 (m, 2 H), 7.94 - 8.00 (m, 1 H), 8.04 (dd, *J*=7.48, 1.10 Hz, 1 H), 8.14 (s, 1 H), 9.15 (s, 1 H). ^{13}C NMR (101 MHz, chloroform-*d*) δ ppm 21.75, 115.00, 127.68, 128.39, 128.67, 128.96, 129.90, 131.40, 132.43, 138.05, 138.24, 140.54, 145.89, 152.15. ^{1}H COSY, ^{13}C HSQC and ^{13}C HMBC included to confirm regioselectivity. HRMS (ESI^{+}) m/z calculated for $C_{16}H_{12}BrNO_3S$ [M+H]$^{+}$ 377.9794, found 377.9789.

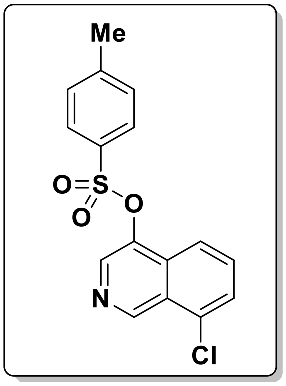

Compound 3g. Separation with heptane/85:15 ethyl acetate and 7N ammonia in methanol (0% to 100%) leads to 44% yield of product **3g** as an orange oil (74 mg, 0.22 mmol). *Note: add preactivated 4Å MS prior to Ts_2O and keep stirring for 16 h during the addition step*. ^{1}H NMR (400 MHz, chloroform-*d*) δ ppm 2.46 (s, 3 H), 7.35 (d, *J*=7.94 Hz, 2 H), 7.57 - 7.64 (m, 1 H) 7.64 - 7.69 (m, 1 H) 7.79 (d, *J*=8.36 Hz, 2 H) 7.93 (d, *J*=8.26 Hz, 1 H) 8.19 (s, 1 H) 9.55 (s, 1 H). ^{13}C NMR (101 MHz, chloroform-*d*) δ ppm 21.72, 120.31, 126.92, 128.45, 128.56, 130.09, 131.02, 131.83, 131.93, 132.29, 136.62, 141.51, 146.14, 148.24. ^{1}H COSY, ^{13}C HSQC and ^{13}C HMBC included to confirm regioselectivity. HRMS (ESI$^+$) m/z calculated for $C_{16}H_{12}ClNO_3S$ $[M+H]^+$ 334.0299, found 334.0299.

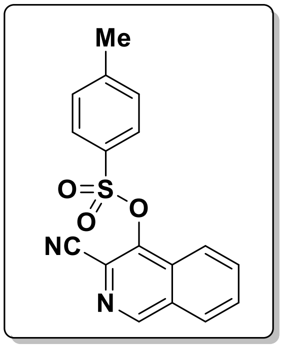

Compound 3h. Separation with heptane/85:15 ethyl acetate and 7N ammonia in methanol (0% to 100%) leads to 52% yield of product **3h** as a beige powder (83 mg, 0.26 mmol). *Note: MTO (10 mol%) and UHP (5 equiv) under reflux for two days at 0.2 M during the oxidation step. Cool down and dilute prior to the addition step*. ^{1}H NMR (400 MHz, chloroform-*d*) δ ppm 2.52 (s, 3 H), 7.46 (d, *J*=8.05 Hz, 2 H), 7.84 - 7.90 (m, 1 H), 7.90 - 7.98 (m, 1 H), 8.01 (d, *J*=8.36 Hz, 2 H), 8.10 (d, *J*=7.94 Hz, 1 H), 8.31 - 8.37 (m, 1 H), 9.18 (s, 1 H). ^{13}C NMR (101 MHz, chloroform-*d*) δ ppm 21.85, 114.82, 121.57, 123.35, 127.52, 128.97, 130.33, 130.83, 131.03, 131.03, 131.58, 132.49, 146.20, 146.91, 151.99. ^{1}H COSY, ^{13}C HSQC and ^{13}C HMBC included to confirm regioselectivity. HRMS (ESI$^+$) m/z calculated for $C_{17}H_{12}N_2O_3S$ $[M+H]^+$ 325.0641, found 325.0640.

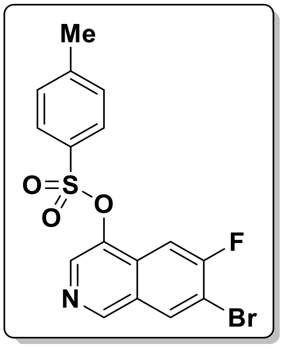

Compound 3i. Separation with heptane/85:15 ethyl acetate and 7N ammonia in methanol (0% to 100%) leads to 72% yield of product **3i** as a pinkish powder (143 mg, 0.36 mmol). ^{1}H NMR (400 MHz, chloroform-*d*) δ ppm 2.48 (s, 3 H), 7.37 (d, *J*=8.14 Hz, 2 H), 7.63 (d, *J*=8.80 Hz, 1 H), 7.80 (d, *J*=8.36 Hz, 2 H), 8.17 (s, 1 H), 8.26 (d, *J*=6.60 Hz, 1 H), 9.05 (s, 1 H). ^{13}C NMR (101 MHz, chloroform-*d*) δ ppm 21.74, 106.94 (d, *J* (^{13}C–^{19}F) =25.43 Hz), 112.48 (d, *J* (^{13}C–^{19}F) =24.66 Hz), 127.47, 128.56, 130.17, 131.10 (d, *J* (^{13}C–^{19}F) =10.02 Hz), 131.77, 132.93 (d, *J* (^{13}C–^{19}F) =2.31 Hz), 136.93, 141.39, 146.36, 149.51 (d, *J* (^{13}C–^{19}F) =1.54 Hz), 159.86 (d, *J* (^{13}C–^{19}F) =255.84 Hz). ^{19}F NMR (377 MHz, chloroform-*d*) δ ppm -99.11. ^{1}H COSY, ^{13}C HSQC and ^{13}C HMBC included to confirm regioselectivity. HRMS (ESI^{+}) m/z calculated for $C_{16}H_{11}BrFNO_3S$ $[M+H]^+$ 395.9700, found 395.9699.

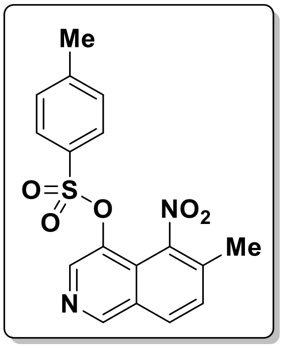

Compound 3j. Separation with heptane/85:15 ethyl acetate and 7N ammonia in methanol (0% to 100%) leads to 66% yield of product **3j** as a pinkish powder (117 mg, 0.33 mmol). ^{1}H NMR (400 MHz, chloroform-*d*) δ ppm 2.49 (s, 3 H), 2.50 (s, 3 H), 7.41 (d, *J*=8.15 Hz, 2 H), 7.59 (d, *J*=8.36 Hz, 1 H), 7.93 (d, *J*=8.36 Hz, 2 H), 8.07 (d, *J*=8.36 Hz, 1 H), 8.67 (s, 1 H), 9.21 (s, 1 H). ^{13}C NMR (101 MHz, chloroform-*d*) δ ppm 17.94, 21.85, 121.20, 128.62, 128.89, 129.51, 129.97, 131.07, 131.60, 134.57, 136.52, 138.75, 143.53, 146.42, 149.93. ^{1}H COSY, ^{13}C HSQC and ^{13}C HMBC included to confirm regioselectivity. HRMS (ESI^{+}) m/z calculated for $C_{17}H_{14}N_2O_5S$ $[M+H]^+$ 359.0696, found 359.0695.

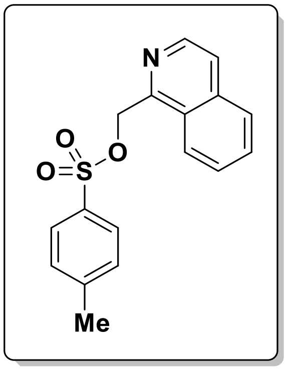

Compound 5. Separation with heptane/85:15 ethyl acetate and 7N ammonia in methanol (0% to 100%) leads to 65% yield of product **5** as a red oil (49 mg, 0.13 mmol, 17% of constitutional isomer). *Note: reaction at 0.2 mmol scale. Dilute to 0.04 M, add preactivated 4Å MS prior to Ts_2O and keep stirring for 16 h during the addition step*. ^{1}H NMR (400 MHz, chloroform-*d*) δ ppm 2.43 (s, 3 H), 5.64 (s, 2 H), 7.25 - 7.30 (m, 2 H), 7.64 - 7.70 (m, 2 H), 7.71 - 7.76 (m, 1 H), 7.78 - 7.83 (m, 2 H), 7.85 (d, *J*=8.58 Hz, 1 H), 8.17 - 8.25 (m, 1 H), 8.44 (d, *J*=5.72 Hz, 1 H). ^{13}C NMR (101 MHz, chloroform-*d*) δ ppm 21.64, 71.43, 122.46, 125.09, 127.31, 128.14, 128.32, 129.78, 130.74. ^{1}H COSY, ^{13}C HSQC and ^{13}C HMBC included to confirm regioselectivity. HRMS (ESI$^+$) m/z calculated for $C_{17}H_{15}NO_3S$ $[M+H]^+$ 314.0845, found 314.0843. The corresponding compound **3** was observed as by-product in 19% LCAP.

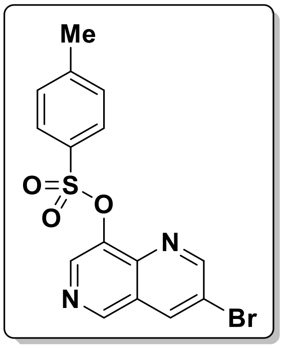

Compound 3k. Separation with heptane/ethyl acetate with 0.5% triethylamine (0% to 100%) leads to 68% yield of product **3k** as a beige powder (51.6 mg, 0.14 mmol). *Note: reaction at 0.2mmol scale and using UHP (1.5 equiv) during the oxidation step*. ^{1}H NMR (400 MHz, chloroform-*d*) δ ppm 2.47 (s, 3 H), 7.34 (d, *J*=8.15 Hz, 2 H), 7.88 (d, *J*=8.36 Hz, 2 H), 8.47 (d, *J*=2.30 Hz, 1 H), 8.57 (s, 1 H), 9.05 (d, *J*=2.19 Hz, 1 H), 9.13 (s, 1 H). ^{13}C NMR (101 MHz, chloroform-*d*) δ ppm 21.74, 119.53, 125.52, 128.74, 129.79, 132.58, 136.41, 140.48, 141.72, 142.42, 145.75, 150.46, 156.42. ^{1}H COSY, ^{13}C HSQC and ^{13}C HMBC included to confirm regioselectivity. HRMS (ESI$^+$) m/z calculated for $C_{15}H_{11}BrN_2O_3S$ $[M+H]^+$ 378.9747, found 378.9747.

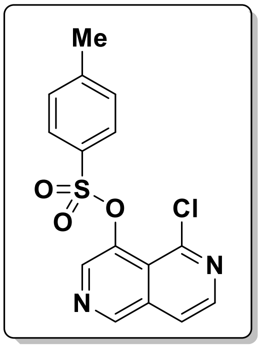

Compound 3l. Separation with heptane/ethyl acetate with 0.5% triethylamine (0% to 100%) leads to 59% yield of product **3l** as yellow crystals (97.8 mg, 0.29 mmol). *Note: quench the reaction after 30 min during the addition step.* ^{1}H NMR (400 MHz, chloroform-*d*) δ ppm 2.47 (s, 3 H), 7.37 (d, *J*=8.26 Hz, 2 H), 7.74 (d, *J*=5.54 Hz, 1 H), 7.83 (d, *J*=8.36 Hz, 2 H), 8.40 (s, 1 H), 8.50 (d, *J*=5.54 Hz, 1 H), 9.22 (s, 1 H). ^{13}C NMR (101 MHz, chloroform-*d*) δ ppm 21.77, 118.81, 123.59, 128.81, 130.05, 132.07, 133.95, 139.42, 140.07, 143.99, 146.29, 146.87, 150.98. ^{1}H COSY, ^{13}C HSQC and ^{13}C HMBC included to confirm regioselectivity. HRMS (ESI$^+$) m/z calculated for $C_{15}H_{11}ClN_2O_3S$ $[M+H]^+$ 335.0252, found 335.0251.

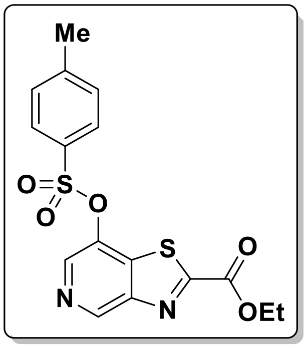

Compound 3m. Separation with heptane/85:15 ethyl acetate and 7N ammonia in methanol (0% to 100%) leads to 52% yield of product **3m** as a pink paste (98 mg, 0.26 mmol). *Note: use UHP (3 equiv) during the oxidation step and stir 30min during the addition step*. ^{1}H NMR (400 MHz, chloroform-*d*) δ ppm 1.48 (t, *J*=7.15 Hz, 3 H), 2.46 (s, 3 H), 4.56 (q, *J*=7.26 Hz, 2 H), 7.32 - 7.39 (m, 2 H), 7.73 - 7.79 (m, 2 H), 8.24 (s, 1 H), 9.39 (s, 1 H). ^{13}C NMR (101 MHz, chloroform-*d*) δ ppm 14.14, 21.74, 63.77, 128.61, 130.27, 131.20, 139.00, 139.16, 140.69, 145.90, 146.68, 151.57, 159.35, 161.40. ^{1}H COSY, ^{13}C HSQC and ^{13}C HMBC included to confirm regioselectivity. HRMS (ESI$^+$) m/z calculated for $C_{16}H_{14}N_2O_5S_2$ $[M+H]^+$ 379.0417, found 379.0417.

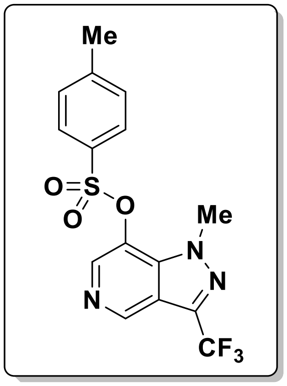

Compound 3n. Separation with heptane/85:15 ethyl acetate and 7N ammonia in methanol (0% to 100%) leads to 76% yield of product **3n** as a white paste (142 mg, 0.38 mmol). *Note: use UHP (1.5 equiv) during the oxidation step*. ^{1}H NMR (400 MHz, chloroform-*d*) δ ppm 2.51 (s, 3 H), 4.34 (s, 3 H), 7.42 (d, *J*=8.14 Hz, 2 H), 7.72 - 7.85 (m, 3 H), 9.03 (s, 1 H). ^{13}C NMR (101 MHz, chloroform-*d*) δ ppm 21.79, 39.28, 115.92 - 125.06 (q, *J* (^{13}C–^{19}F) =269.67 Hz), 120.80, 128.74, 130.37, 131.22, 131.38, 134.23 - 135.47 (q, *J* (^{13}C–^{19}F) =40.40 Hz), 137.16, 137.76, 143.18, 146.92. ^{19}F NMR (377 MHz, chloroform-*d*) δ ppm -60.72. ^{1}H COSY, ^{13}C HSQC and ^{13}C HMBC included to confirm regioselectivity. HRMS (ESI^{+}) m/z calculated for $C_{15}H_{12}F_{3}N_{3}O_{3}S$ $[M+H]^{+}$ 372.0624, found 372.0622.

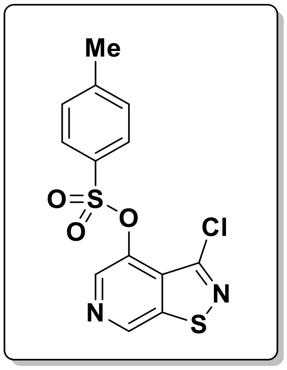

Compound 3o. Separation with heptane/ethyl acetate with 0.5% triethylamine (0% to 100%) leads to 22% yield of product **3o** as a white powder (42 mg, 0.11 mmol). *Note: use UHP (3 equiv) for 3days during the oxidation step and quench after 30 min during the addition step. Filter the crude prior to chromatography, no aqueous work up.* ^{1}H NMR (400 MHz, chloroform-*d*) δ ppm 2.49 (s, 3 H), 7.39 (d, *J*=8.15 Hz, 2 H), 7.84 (d, *J*=8.47 Hz, 2 H), 8.26 (s, 1 H), 9.18 (s, 1 H). ^{13}C NMR (101 MHz, chloroform-*d*) δ ppm 21.79, 128.39, 128.73, 130.15, 131.84, 137.70, 140.72, 141.54, 143.91, 146.49, 150.56. ^{1}H COSY, ^{13}C HSQC and ^{13}C HMBC included to confirm regioselectivity. HRMS (ESI^{+}) m/z calculated for $C_{13}H_{9}ClN_{2}O_{3}S_{2}$ $[M+H]^{+}$ 340.9816, found 340.9815.

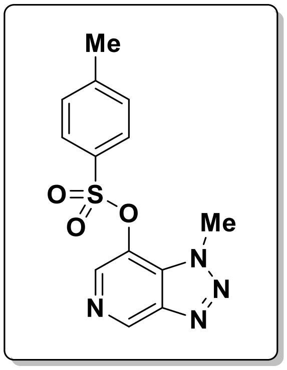

Compound 3p. Separation with heptane/85:15 ethyl acetate and 7N ammonia in methanol (0% to 100%) leads to 48% yield of product **3p** as a white powder (71 mg, 0.24 mmol). *Note: use UHP (1.5 equiv) during the oxidation step and quench after 30 min during the addition step*. ^{1}H NMR (400 MHz, chloroform-*d*) δ ppm 2.49 (s, 3 H), 4.47 (s, 3 H), 7.40 (d, *J*=7.92 Hz, 2 H), 7.72 - 7.78 (m, 3 H), 9.92 (s, 1 H). ^{13}C NMR (101 MHz, chloroform-*d*) δ ppm 21.77, 36.55, 128.71, 130.31, 130.93, 130.95, 131.36, 137.84, 143.23, 145.86, 146.90. ^{1}H COSY, ^{13}C HSQC and ^{13}C HMBC included to confirm regioselectivity. HRMS (ESI^{+}) m/z calculated for $C_{13}H_{12}N_4O_3S$ $[M+H]^+$ 305.0703, found 305.0700.

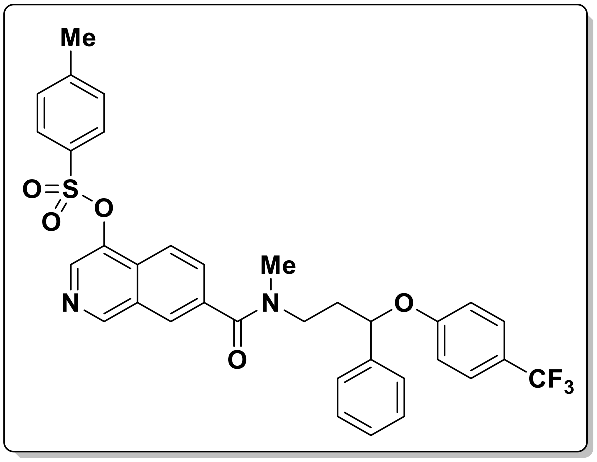

Compound 3q. Separation with heptane/85:15 ethyl acetate and 7N ammonia in methanol (0% to 100%) leads to 60% yield of product **3q** as a white powder (75 mg, 0.12 mmol). *Note: reaction at 0.2 mmol scale. Use UHP (1.5 equiv) during the oxidation step and quench after 30 min during the addition step*. ^{1}H NMR (400 MHz, DMSO-*d*$_6$) δ ppm 2.14-2.33 (m, 2 H), 2.41 (s, 3 H), 2.90-3.06 (m, 3 H), 3.36-3.72 (m, 2 H), 5.27-5.60 (m, 1 H), 6.67-6.69 (m, 1 H), 7.05-7.59 (m, 11 H), 7.65-7.88 (m, 4 H), 8.21 (s, 1 H), 9.13-9.31 (m, 1 H). ^{13}C NMR (101 MHz, DMSO-*d*$_6$) δ ppm 20.86, 21.13, 22.04, 28.65, 28.98, 32.37, 35.21, 36.02, 37.20, 39.79, 44.12, 46.95, 75.86, 77.37, 115.64, 116.17, 120.54, 121.16, 122.93, 125.48, 126.18, 125.72, 126.53, 127.50, 128.48, 127.96, 128.14, 130.04, 129.80, 130.37, 131.03, 136.14, 136.23, 136.31, 136.43, 139.87, 140.70, 140.87, 141.77, 146.39, 151.86, 151.96, 159.74, 160.38, 168.76, 169.13. *Note: signals overlapping.* ^{19}F NMR (376 MHz, DMSO-*d*$_6$) δ ppm -59.99 (s). ^{1}H NMR at 150 °C to distinguish signals from rotamers (400 MHz, DMSO-*d*$_6$) 2.18 - 2.36 (m, 2 H), 2.44 (s, 3 H), 2.98 (s, 3 H), 3.50 - 3.62 (m, 2 H), 5.41-5.44

(m, 1 H), 6.96 (br d, *J*=8.36 Hz, 2 H), 7.17 - 7.24 (m, 1 H), 7.28 (t, *J*=7.47 Hz, 2 H), 7.32 - 7.39 (m, 2 H), 7.48 (d, *J*=8.36 Hz, 4 H), 7.71 (d, *J*=8.69 Hz, 1 H), 7.84 - 7.89 (m, 3 H), 8.11 (s, 1 H), 8.31 (s, 1 H), 9.22 (s, 1 H). HRMS (ESI$^+$) m/z calculated for $C_{34}H_{29}F_3N_2O_5S$ $[M+H]^+$ 635.1822, found 635.1824.

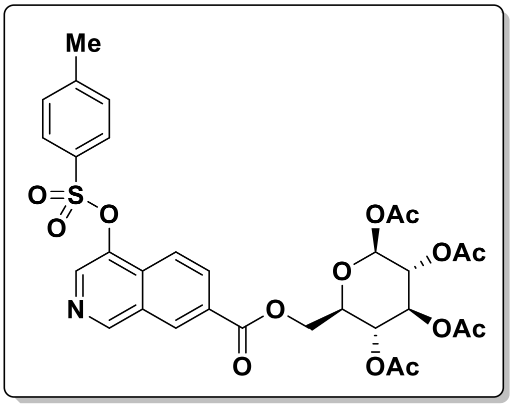

Compound 3r. Separation with heptane/ethyl acetate with 0.5% triethylamine (0% to 100%) leads to 79% yield of product **3r** as white crystals (53 mg, 0.08 mmol). *Note: reaction at 0.1 mmol scale. Quench the reaction after 30 min during the addition step and filter the crude prior to chromatography, no aqueous work up*. ^{1}H NMR (400 MHz, chloroform-*d*) δ ppm 2.02 (s, 3 H), 2.05 (s, 6 H), 2.11 (s, 3 H), 2.45 (s, 3 H), 3.98 - 4.06 (m, 1 H), 4.46 (dd, *J*=12.44, 4.70 Hz, 1 H), 4.49 - 4.62 (m, 1 H), 5.16 - 5.35 (m, 3 H), 5.77 (d, *J*=8.26 Hz, 1 H), 7.34 (d, *J*=8.05 Hz, 2 H), 7.79 (d, *J*=8.36 Hz, 2 H), 8.01 (d, *J*=8.78 Hz, 1 H), 8.23 - 8.29 (m, 2 H), 8.73 - 8.77 (m, 1 H), 9.30 (s, 1 H). ^{13}C NMR (101 MHz, chloroform-*d*) δ ppm 20.50, 20.51, 20.53, 20.75, 21.71, 62.59, 67.92, 70.19, 72.67, 72.74, 91.78, 121.73, 128.53, 128.93, 129.12, 130.09, 130.40, 130.54, 131.82, 132.50, 138.09, 141.68, 146.12, 152.42, 164.85, 168.85, 169.20, 169.29, 169.98. ^{1}H COSY, ^{13}C HSQC and ^{13}C HMBC included to confirm regioselectivity. HRMS (ESI$^+$) m/z calculated for $C_{31}H_{31}NO_{14}S$ $[M+H]^+$ 674.1538, found 674.1541.

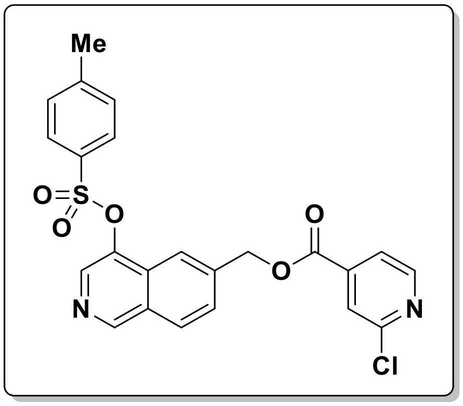

Compound 3s. Separation with heptane/85:15 ethyl acetate and 7N ammonia in methanol (0% to 100%) leads to 68% yield of product **3s** as a white powder (64 mg, 0.14 mmol). *Note: reaction at 0.2 mmol scale. Quench the reaction after 30 min during the addition step and filter the crude*

prior to chromatography, no aqueous work up. ^{1}H NMR (400 MHz, chloroform-*d*) δ ppm 2.46 (s, 3 H), 5.56 (s, 2 H), 7.35 (d, *J*=8.15 Hz, 2 H), 7.69 (dd, *J*=8.47, 1.46 Hz, 1 H), 7.81 (d, *J*=8.36 Hz, 2 H), 7.85 (dd, *J*=5.02, 1.36 Hz, 1 H), 7.95 (s, 1 H), 8.05 (d, *J*=8.57 Hz, 1 H), 8.07 - 8.12 (m, 2 H), 8.58 (d, *J*=5.12 Hz, 1 H), 9.16 (s, 1 H). ^{13}C NMR (101 MHz, chloroform-*d*) δ ppm 21.72, 67.03, 120.36, 121.64, 124.09, 127.61, 128.03, 128.56, 129.40, 130.07, 130.59, 132.00, 136.55, 138.26, 139.76, 141.93, 146.08, 150.68, 150.99, 152.58, 163.50. ^{1}H COSY, ^{13}C HSQC and ^{13}C HMBC included to confirm regioselectivity. HRMS (ESI^{+}) m/z calculated for $C_{23}H_{16}ClN_2O_5S$ $[M+H]^+$ 469.0619, found 469.0619.

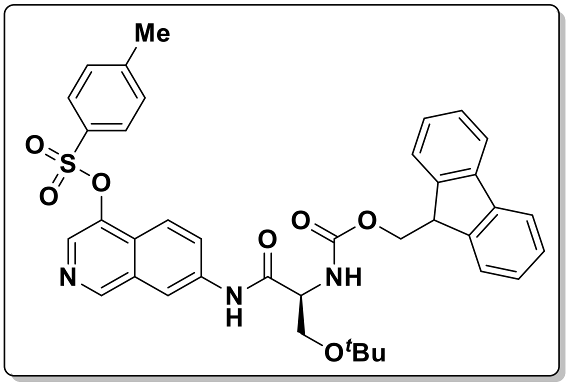

Compound 3t. Separation with heptane/85:15 ethyl acetate and 7N ammonia in methanol (0% to 100%) leads to 53% yield of product **3t** as yellow crystals (36 mg, 0.05 mmol). *Note: reaction at 0.1 mmol scale. Quench the reaction after 30 min during the addition step and filter the crude prior to chromatography, no aqueous work up*. ^{1}H NMR (400 MHz, chloroform-*d*) δ ppm 1.29 (br s, 9 H), 2.46 (s, 3 H), 3.49 - 3.56 (m, 1 H), 3.95 (br s, 1 H), 4.25 – 4.28 (br t, *J*=6.82 Hz, 1 H), 4.41 - 4.46 (m, 1 H), 4.49 (br d, *J*=6.82 Hz, 2 H), 5.82 (br s, 1 H), 7.31 - 7.35 (m, 4 H), 7.39 - 7.43 (m, 2 H), 7.55 (dd, *J*=9.02, 1.98 Hz, 1 H), 7.59 - 7.63 (m, 2 H), 7.77 - 7.81 (m, 4 H), 7.99 - 8.01 (m, 2 H), 8.50 (d, *J*=1.98 Hz, 1 H), 8.95 - 9.08 (m, 1 H). ^{13}C NMR (101 MHz, chloroform-*d*) δ ppm 21.74, 27.51, 47.17, 55.00, 61.67, 67.27, 75.03, 115.18, 120.04, 122.52, 124.63, 124.99, 127.08, 127.47, 127.79, 128.60, 130.05, 130.57, 132.02, 134.93, 136.94, 141.33, 141.88, 143.68, 145.98, 150.86, 156.29, 169.00. ^{1}H COSY, ^{13}C HSQC and ^{13}C HMBC included to confirm regioselectivity. HRMS (ESI^{+}) m/z calculated for $C_{38}H_{37}N_3O_7S$ $[M+H]^+$ 680.2425, found 680.2421.

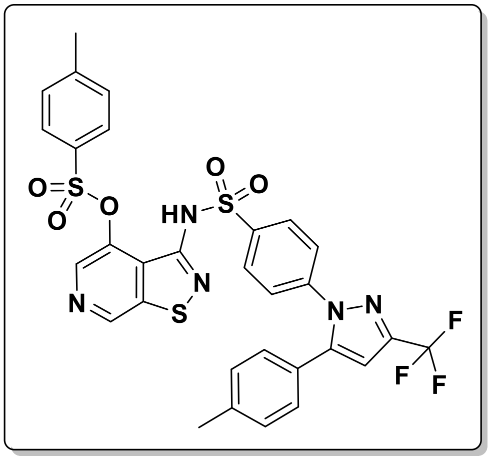

Compound 3u. Separation with heptane/3:1 ethyl acetate and ethanol with 0.5 % triethylamine (0% to 100%) leads to 44% yield of product **3u** as a white powder (30 mg, 0.044 mmol). *Note: reaction at 0.1 mmol scale. Oxidation performed with 1 equiv. of m-CPBA. Quench the reaction after 30 min during the addition step and inject the crude directly for chromatography, no aqueous work up*. ^{1}H NMR (400 MHz, dichloromethane-d_2) δ ppm 2.37 (s, 3 H), 2.50 (s, 3 H), 6.75 (s, 1 H), 7.08 - 7.13 (m, 2 H), 7.13 - 7.20 (m, 2 H), 7.47 (d, *J*=8.67 Hz, 4 H), 7.91 (d, *J*=8.36 Hz, 2 H), 8.11 - 8.17 (m, 2 H), 8.26 (s, 1 H), 8.51 (br s, 1 H), 9.08 (s, 1 H). ^{13}C NMR (101 MHz, dichloromethane-d_2) δ ppm 21.62, 22.22, 106.84 (m), 121.82 (m), 125.30, 125.73, 126.25, 129.21, 129.32, 130.21, 130.28, 131.17, 131.26, 137.71, 138.62, 140.49, 140.62, 142.36, 143.91, 144.36 (m), 146.02, 148.19, 148.37, 150.58. ^{19}F NMR (376 MHz, dichloromethane-d_2) δ ppm -62.86 (s). ^{1}H COSY, ^{13}C HSQC and ^{13}C HMBC included to confirm regioselectivity. HRMS (ESI$^+$) m/z calculated for $C_{30}H_{23}F_3N_5O_5S_3$ $[M+H]^+$ 686.0808, found 686.0808.

Note: Unfortunately, quinolines, quinazolines, pyridines and 4-susbtituted isoquinolines do not undergo tosylation under these conditions.

V. Derivatization of Isoquinolin-4-yl Tosylate

1. Deprotection to the hydroxyl group

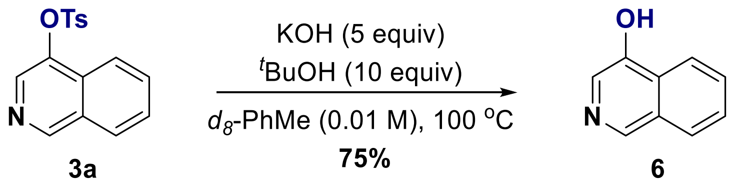

Weigh substrate **3a** (15 mg, 0.05 mmol, 1 equiv) in a 8 ml capped vial equipped with a magnetic stirring bar. Dissolve in deuterated PhMe (0.01 M) and add ^{t}BuOH (10 equiv) followed by KOH (5 equiv). Stir at 100 °C for 30 min and analyze the crude by UPLC-MS (5 μl of sample diluted in 195 μl of a 0.02 M solution of 4,4'-di-*tert*-butyl-1,1'-biphenyl in acetonitrile). Add trimethoxybenzene to measure the NMR yield (75 % of product **6**). Spectroscopic data compared with authentic sample.

2. Nickel-catalyzed Kumada cross-coupling

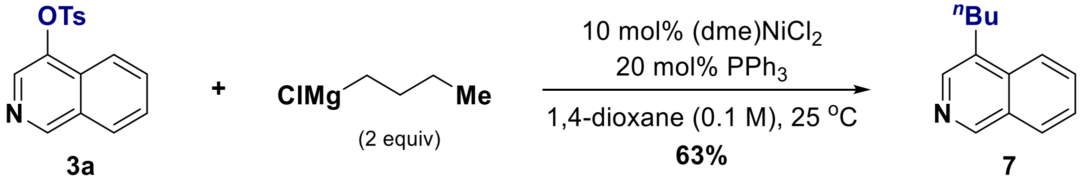

Weigh substrate **3a** (60 mg, 0.2 mmol, 1 equiv) in a 8 ml capped vial equipped with a magnetic stirring bar. Add (dme)$NiCl_2$ (10 mol%), triphenylphosphine (20 mol%) and place them under nitrogen atmosphere. Dissolve in 1,4-dioxane (0.1 M) and add ^{n}BuMgCl 2M in THF (2.0 equiv). Stir at 23 °C for 2 h, quench with water and filter the mixture over celite. Concentrate under reduced pressure and purify the desired product by CombiFlash silica gel chromatography (DCM injection). Separation with heptane/ethyl acetate (0% to 100%) leads to 63% yield of compound **7** as a dark yellow oil (23.4 mg, 0.101 mmol). Spectroscopic data matched that reported in the reported in the literature.[1]

[1]A. J. Day, T. C. Jenkins, M. Kischkewitz, K. E. Christensen, D. L. Poole, T. J. Donohoe, *Org. Lett.* **2023**, *25*, 614.

3. Palladium-catalyzed phosphorylation reaction

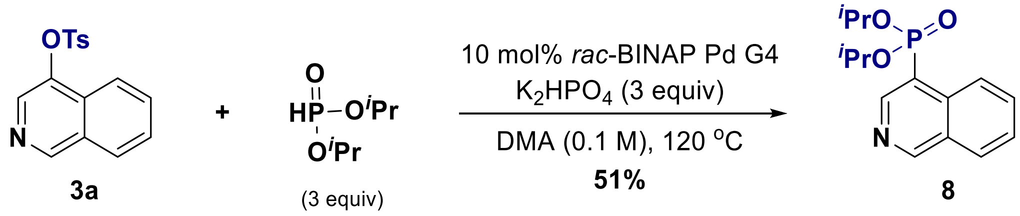

Weigh substrate **3a** (60 mg, 0.2 mmol, 1 equiv) in a 8 ml capped vial equipped with a magnetic stirring bar. Add K_2HPO_4 (3 equiv), *rac*-BINAP Pd G4 (10 mol%) and place them under nitrogen atmosphere. Dissolve in DMA (0.1 M) and add diisopropyl phosphite neat (3 equiv). Stir at 120 °C for 18 h and filter the mixture over celite. Concentrate under reduced pressure and purify the desired product by CombiFlash silica gel chromatography (DCM injection). Separation with heptane/90:10 DCM and methanol (0% to 100%) leads to 51% yield of compound **8** as a yellow oil (29.7 mg, 0.101 mmol). ^{1}H NMR (400 MHz, chloroform-*d*) δ ppm 1.19 (d, *J*=6.16 Hz, 6 H), 1.41 (d, *J*=6.16 Hz, 6 H), 4.79 (dquin, *J*=7.91, 6.22, 6.22, 6.22, 6.22 Hz, 2 H), 7.63 - 7.72 (m, 1 H), 7.81 (td, *J*=7.76, 1.21 Hz, 1 H), 8.02 (br d, *J*=8.14 Hz, 1 H), 8.49 (d, *J*=8.58 Hz, 1 H), 9.09 (d, *J*=9.46 Hz, 1 H), 9.37 (d, *J*=2.42 Hz, 1 H). ^{13}C NMR (101 MHz, chloroform-*d*) δ ppm 23.74 (d, *J* (^{13}C–^{31}P) =4.40 Hz), 24.10 (d, *J* (^{13}C–^{31}P) =3.67 Hz), 71.38 (d, *J* (^{13}C–^{31}P) =5.87 Hz), 120.02 (d, *J* (^{13}C–^{31}P) =184.13 Hz), 126.05 (d, *J* (^{13}C–^{31}P) =4.40 Hz), 127.71 (s), 128.14 (d, *J* (^{13}C–^{31}P) =9.54 Hz), 128.38 (d, *J* (^{13}C–^{31}P) =1.47 Hz), 131.55 (s), 135.12 (d, *J* (^{13}C–^{31}P) =8.80 Hz), 149.12 (d, *J* (^{13}C–^{31}P) =12.47 Hz), 157.04 (d, *J* (^{13}C–^{31}P) =3.67 Hz). ^{31}P NMR (162 MHz, chloroform-*d*) δ ppm 14.27 (s). HRMS (ESI^{+}) m/z calculated for $C_{15}H_{20}NO_3P$ $[M+H]^{+}$ 294.1254, found 294.1253.

4. Palladium-catalyzed Buchwald-Hartwig cross-coupling

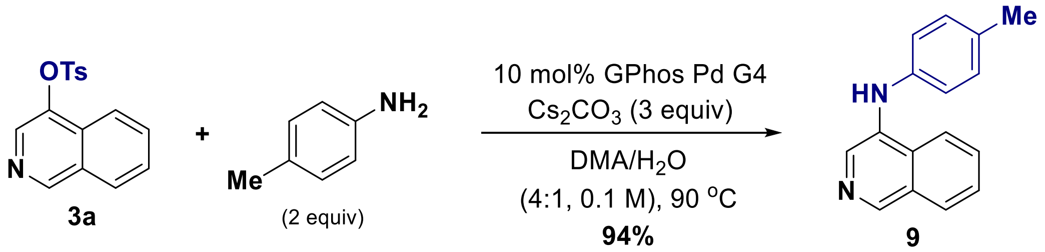

Weigh substrate **3a** (150 mg, 0.5 mmol, 1 equiv) in a 40 ml capped vial equipped with a magnetic stirring bar. Add *p*-toluidine (2 equiv) followed by GPhos Pd G4 (10 mol%) and evacuate/backfill with nitrogen three times. Dissolve in DMA (4 ml) and add a solution of Cs_2CO_3 (3 equiv) in degassed water (1 ml). Stir at 90 °C for 18 h and cool to room temperature. Pour the mixture into ice-cold water and extract with ethyl acetate. Dry the organic phase with $MgSO_4$, concentrate under reduced pressure and purify the desired product by CombiFlash silica gel chromatography (DCM injection). Separation with DCM/90:10 DCM and 7N ammonia in methanol (0% to 50%) leads to 94% yield of compound **9** as a pale brown powder (120 mg, 0.47 mmol). Spectroscopic data matched that reported in the literature.[2]

[2]Q. Shen, T. Ogata, J. F. Hartwig, *J. Am. Chem. Soc.* **2008**, *130*, 6586.

5. Palladium-catalyzed Sonogashira cross-coupling

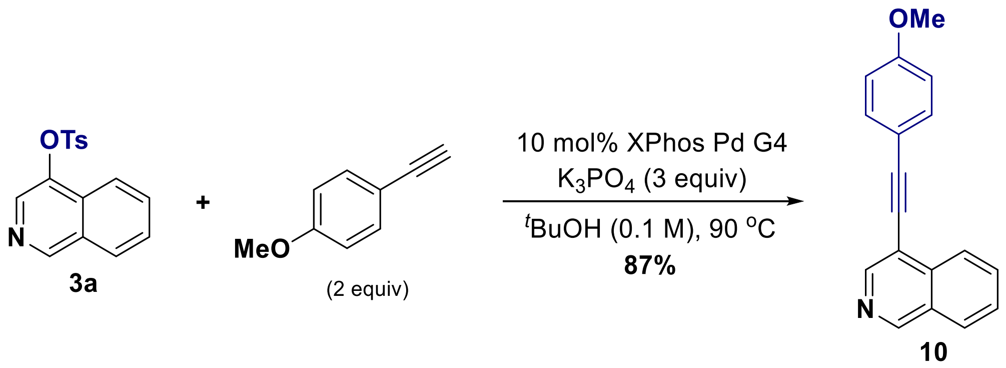

Weigh substrate **3a** (150 mg, 0.5 mmol, 1 equiv) in a 40 ml capped vial equipped with a magnetic stirring bar. Add 4-ethynylanisole (2 equiv) followed by XPhos Pd G4 (10 mol%) and K_3PO_4 (3 equiv). Evacuate/backfill with nitrogen three times and dissolve in ^{t}BuOH (0.1 M). Stir at 90 °C for 18 h and concentrate the mixture under reduced pressure. Dilute the crude with ethyl acetate and wash with brine. Dry the organic phase with $MgSO_4$, concentrate and purify the desired product by CombiFlash silica gel chromatography (DCM injection). Separation with DCM/90:10 DCM and methanol (0% to 100%) leads to 87% yield of compound **10** as a brown oil (144 mg, 0.43 mmol). Spectroscopic data matched that reported in the literature.[3]

6. Palladium-catalyzed Suzuki cross-coupling

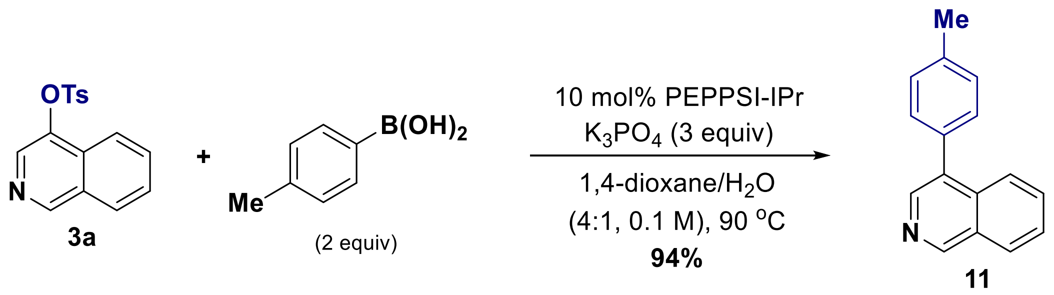

Weigh substrate **3a** (150 mg, 0.5 mmol, 1 equiv) in a 40 ml capped vial equipped with a magnetic stirring bar. Add 4-methylphenylboronic acid (2 equiv) followed by PEPPSI-IPr (10 mol%) and evacuate/backfill with nitrogen three times. Dissolve in 1,4-dioxane (4 ml) and add a solution of K_3PO_4 (3 equiv) in degassed water (1 ml). Stir at 90 °C for 18 h and concentrate the mixture under reduced pressure. Dilute the crude with ethyl acetate and wash with water and brine. Dry the organic phase with $MgSO_4$, concentrate and purify the desired product by CombiFlash silica gel chromatography (DCM injection). Separation with DCM/90:10 DCM and methanol (0% to 70%) leads to 94% yield of compound **11** as a red oil (107 mg, 0.47 mmol). Spectroscopic data matched that reported in the literature.[4]

[3]A. Elangovan, S. W. Yang, J. H. Lin, K. M. Kao, T. I. Ho, *Org. Biomol. Chem*. **2004**, *2*, 1597.
[4]V. K. Tiwari, G. G. Pawar, H. S. Jena, M. Kapur, *Chem. Commun*. **2014**, *50*, 7322.

VI. Preparation of Starting Materials

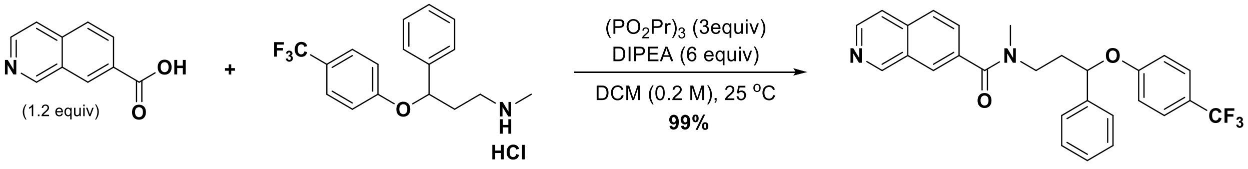

Weigh fluoxetine hydrochloride (346 mg, 1 mmol, 1 equiv) and isoquinoline-7-carboxylic acid (1.2 equiv) in a 20 ml capped vial equipped with a magnetic stirring bar. Dissolve in DCM (0.2 M) and add DIPEA (6 equiv) followed by $(PO_2Pr)_3$ 1.6 M in ethyl acetate (3 equiv). Stir at 25 °C for 18 h and analyze the crude by UPLC-MS (5 μl of sample diluted in 195 μl of a 0.02 M solution of 4,4'-di-*tert*-butyl-1,1'-biphenyl in acetonitrile). Dilute the crude with DCM and wash the mixture with brine, separate the organic phase and dry it with $MgSO_4$. Concentrate under reduced pressure and purify the desired product by CombiFlash silica gel chromatography (DCM injection). Separation with heptane/ethyl acetate with 25% ethanol (0% to 100%) leads to quantitative yield of precursor of **3q** as white crystals (480 mg, 1 mmol). ^{1}H NMR (400 MHz, DMSO-*d6*) δ ppm 2.09-2.38 (m, 2 H), 2.95-3.06 (m, 3 H), 3.37-3.49 (m, 1 H), 3.57-3.79 (m, 1 H), 5.28-5.602 (m, 1 H), 6.69-6.71 (m, 1 H), 7.07-8.12 (m, 12 H), 8.55-8.56 (m, 1 H), 9.21-9.34 (m, 1 H). ^{13}C NMR (101 MHz, DMSO-*d6*) δ ppm 32.40, 35.22, 36.07, 37.30, 44.10, 47.12, 76.07, 76.07, 77.40, 115.71, 116.19, 117.98, 120.13, 120.65, 120.97, 121.29, 121.60, 123.01, 125.41, 125.76, 126.02, 126.60, 126.77, 127.37, 127.57, 127.77, 128.38, 128.60, 128.98, 135.08, 135.01, 135.32, 139.92, 140.73, 143.57, 143.60, 143.63, 152.67, 159.84, 160.40, 169.32, 169.72. *Note: signals overlapping.* ^{19}F NMR (376 MHz, methanol-*d4*) δ ppm -62.98 (s). ^{1}H NMR at 150 °C to distinguish signals from rotamers (400 MHz, DMSO-*d*6) 2.19 - 2.38 (m, 2 H), 3.01 (s, 3 H), 3.53 - 3.64 (m, 2 H), 5.43 (dd, *J*=7.37, 5.07 Hz, 1 H), 6.97 (br d, *J*=8.57 Hz, 2 H), 7.18 - 7.25 (m, 1 H), 7.28 (t, *J*=7.42 Hz, 2 H), 7.33 - 7.40 (m, 2 H), 7.48 (d, *J*=8.78 Hz, 2 H), 7.66 (dd, *J*=8.41, 1.62 Hz, 1 H), 7.79 (d, *J*=5.64 Hz, 1 H), 7.92 (d, *J*=8.47 Hz, 1 H), 8.03 (s, 1 H), 8.55 (d, *J*=5.75 Hz, 1 H), 9.28 (s, 1 H). HRMS (ESI$^+$) m/z calculated for $C_{27}H_{23}N_2F_3O_2$ $[M+H]^+$ 465.1784, found 465.1782.

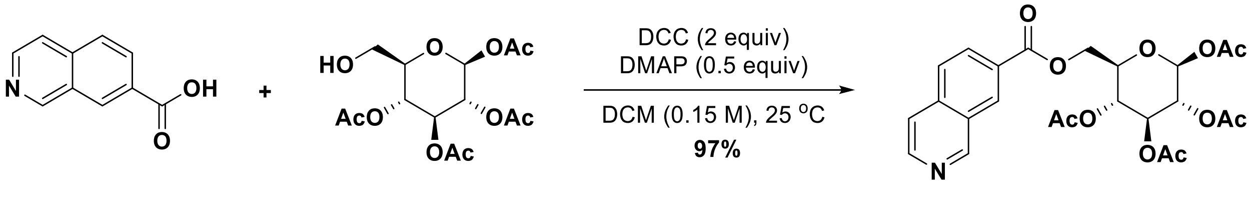

Weigh isoquinoline-7-carboxylic acid (270 mg, 1.6 mmol, 1 equiv), 1,2,3,4-*tetra*-O-acetyl-*beta*-D-glucopyranose (1 equiv), DMAP (0.5 equiv) and DCC (2 equiv) in a 40 ml capped vial equipped with a magnetic stirring bar. Dissolve in DCM (0.15 M), stir at 25 °C for 18 h and analyze the crude by UPLC-MS (5 μl of sample diluted in 195 μl of a 0.02 M solution of 4,4'-di-*tert*-butyl-1,1'-biphenyl in acetonitrile). Dilute the crude with DCM and wash the mixture with water, separate the organic phase and dry it with $MgSO_4$. Concentrate under reduced pressure and purify the desired product by CombiFlash silica gel chromatography (DCM injection). Separation with heptane/ethyl acetate

(0% to 100%) leads to 97% yield of precursor of **3r** as a white powder (762 mg, 1.5 mmol). ^{1}H NMR (400 MHz, chloroform-*d*) δ ppm 2.04 (s, 3 H), 2.06 (s, 6 H), 2.13 (s, 3 H), 4.01 - 4.07 (m, 1 H), 4.46 - 4.53 (m, 1 H), 4.54 - 4.61 (m, 1 H), 5.19 - 5.24 (m, 1 H), 5.26 - 5.36 (m, 2 H), 5.79 (d, *J*=8.26 Hz, 1 H), 7.72 (d, *J*=5.75 Hz, 1 H), 7.91 (d, *J*=8.57 Hz, 1 H), 8.29 (dd, *J*=8.62, 1.62 Hz, 1 H), 8.65 (d, *J*=5.75 Hz 1 H), 8.78 (s, 1 H), 9.43 (s, 1 H). ^{13}C NMR (101 MHz, chloroform-*d*) δ ppm 20.58, 20.58, 20.81, 62.52, 68.05, 70.26, 72.79, 72.83, 91.83, 120.27, 127.00, 127.82, 128.29, 129.70, 131.18, 137.99, 145.26, 153.99, 165.36, 168.92, 169.26, 169.35, 170.06. *Note: signals overlapping.* HRMS (ESI$^+$) m/z calculated for $C_{24}H_{25}NO_{11}$ [M+H]$^+$ 504.1500, found 504.1498.

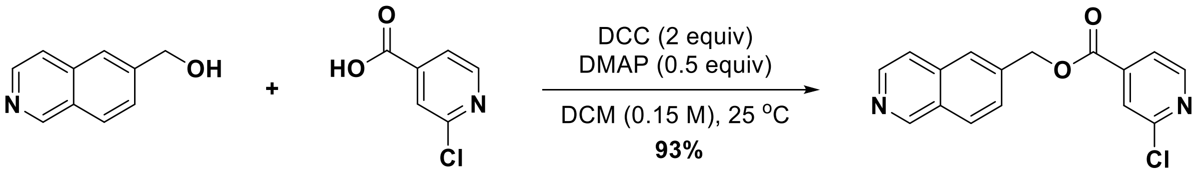

Weigh isoquinolin-6-yl methanol (255 mg, 1.6 mmol, 1 equiv), 2-chloroisonicotinic acid (1 equiv), DMAP (0.5 equiv) and DCC (2 equiv) in a 40 ml capped vial equipped with a magnetic stirring bar. Dissolve in DCM (0.15 M), stir at 25 °C for 18 h and analyze the crude by UPLC-MS (5 μl of sample diluted in 195 μl of a 0.02 M solution of 4,4'-di-*tert*-butyl-1,1'-biphenyl in acetonitrile). Dilute the crude with DCM and wash the mixture with water, separate the organic phase and dry it with $MgSO_4$. Concentrate under reduced pressure and purify the desired product by CombiFlash silica gel chromatography (DCM injection). Separation with heptane/ethyl acetate (0% to 100%) leads to 93% yield of precursor of **3s** as a pale yellow powder (446 mg, 1.5 mmol). ^{1}H NMR (400 MHz, chloroform-*d*) δ ppm 5.59 (s, 2 H), 7.64 - 7.70 (m, 2 H), 7.82 (dd, *J*=5.12, 1.36 Hz, 1 H), 7.89 (s, 1 H), 7.92 - 7.94 (m, 1 H), 8.04 (d, *J*=8.47 Hz, 1 H), 8.56 – 8.59 (m, 2 H), 9.29 (s, 1 H). ^{13}C NMR (101 MHz, chloroform-*d*) δ ppm 67.40, 120.44, 121.63, 124.09, 125.88, 126.93, 128.34, 128.44, 135.67, 137.11, 139.89, 143.77, 150.63, 152.37, 152.57, 163.60. HRMS (ESI$^+$) m/z calculated for $C_{16}H_{11}ClN_2O_2$ [M+H]$^+$ 299.0582, found 299.0581.

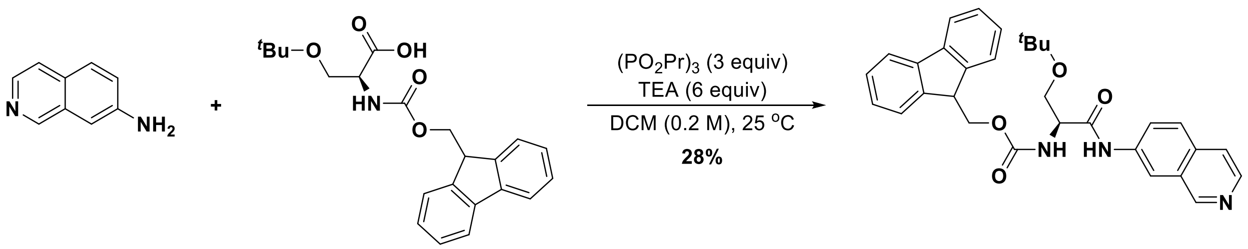

Weigh 7-aminoisoquinoline (144 mg, 1 mmol, 1 equiv) and Fmoc-Ser(^{t}Bu)-OH (1 equiv) in a 20 ml capped vial equipped with a magnetic stirring bar. Dissolve in DCM (0.2 M) and add TEA (6 equiv) followed by $(PO_2Pr)_3$ 1.6 M in ethyl acetate (3 equiv). Stir at 25 °C for 18 h and analyze the crude by UPLC-MS (5 μl of sample diluted in 195 μl of a 0.02 M solution of 4,4'-di-*tert*-butyl-1,1'-biphenyl in acetonitrile). Dilute the crude with DCM and wash the mixture with water, separate the organic phase and dry it with $MgSO_4$. Concentrate under reduced pressure and purify the desired product by CombiFlash silica gel chromatography (DCM injection). Separation with heptane/ethyl acetate with 25% ethanol (0% to 100%) leads to 28% yield of precursor of product **3t** as yellow

crystals (142 mg, 0.3 mmol). ^{1}H NMR (400 MHz, chloroform-*d*) δ ppm 1.29 (br s, 9 H), 3.53 (t, *J*=8.57 Hz, 1 H), 3.96 (br s, 1 H), 4.26 (br t, *J*=6.85 Hz, 1 H), 4.39 (br d, *J*=7.32 Hz, 1 H), 4.48 (br d, *J*=6.90 Hz, 2 H), 5.91 (br s, 1 H), 7.32 (br s, 2 H), 7.36 - 7.46 (m, 2 H), 7.59 - 7.68 (m, 4 H), 7.74 - 7.83 (m, 3 H), 8.38 (s, 1 H), 8.48 (d, *J*=5.75 Hz, 1 H), 8.95 - 9.20 (m, 1 H), 9.23 (s, 1 H). ^{13}C NMR (101 MHz, chloroform-*d*) δ ppm 27.49, 47.13, 54.81, 61.73, 67.23, 74.91, 115.86, 119.87, 120.01, 120.28, 124.31, 124.99, 125.19, 127.01, 127.05, 127.53, 127.59, 127.75, 129.09, 132.98, 136.26, 141.22, 141.30, 141.64, 143.60, 143.69, 151.77, 156.18, 168.87. HRMS (ESI$^+$) m/z calculated for $C_{31}H_{31}N_3O_4$ [M+H]$^+$ 510.2387, found 510.2390.

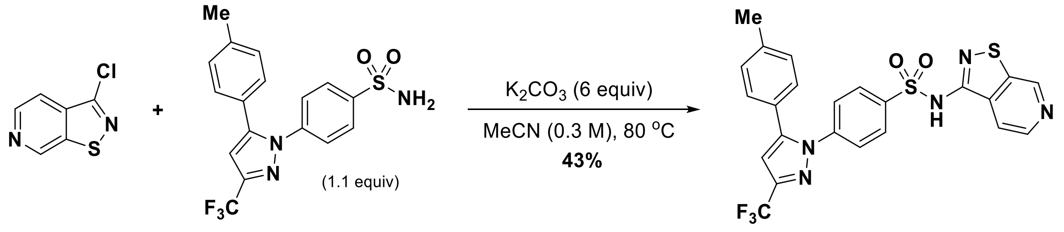

Weigh 3-chloro-[1,2]thiazolo[5,4-C]pyridine (150 mg, 0.88 mmol, 1 equiv) and Celecoxib (1.1 equiv) in a 20 ml capped vial equipped with a magnetic stirring bar. Dissolve in MeCN (0.3 M) and add K_2CO_3 (4 equiv). Stir at 80 °C for 6 h and analyze the crude by UPLC-MS (5 μl of sample diluted in 195 μl of a 0.02 M solution of 4,4'-di-*tert*-butyl-1,1'-biphenyl in acetonitrile). Dilute the crude with DCM and filter the precipitate to then redissolve it in MeOH. Concentrate under reduced pressure and dissolve in ethyl acetate. Wash the mixture with saturated $NaHCO_3$, separate the organic phase and age overnight. Drying the solution with $MgSO_4$ and concentrating under reduced pressure leads to 46% yield of precursor of **3u** as yellow crystals (280 mg, 0.4 mmol). ^{1}H NMR (400 MHz, DMSO-*d$_6$*) δ ppm 2.29 (s, 3 H), 7.14 (m, 4 H), 7.18 (s, 1 H), 7.53 - 7.59 (m, 2 H), 8.04 - 8.09 (m, 2 H), 8.20 (d, *J*=5.64 Hz, 1 H), 8.60 (d, *J*=5.54 Hz, 1 H), 9.47 (s, 1 H), 12.31 (br s, 1 H). ^{13}C NMR (101 MHz, DMSO-*d$_6$*) δ ppm 20.76, 106.09, 116.50, 121.20 (m), 125.14, 126.09, 128.65, 128.82, 129.31, 131.93, 139.05, 139.80, 142.08, 142.26 (m), 143.36, 144.12, 145.29, 146.61, 151.04. ^{19}F NMR (376 MHz, DMSO-*d$_6$*) δ ppm -60.96 (s). HRMS (ESI$^+$) m/z calculated for $C_{23}H_{17}F_3N_5O_2S_2$ [M+H]$^+$ 516.0770, found 516.0778.

VII. Mechanistic Insights

1. Competitive deuterium incorporation

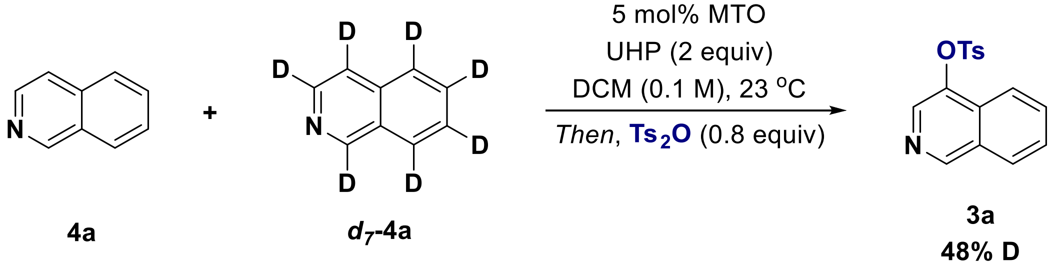

Weigh isoquinoline (65 mg, 0.5 mmol, 0.5 equiv) and d_7-isoquinoline (68 mg, 0.5 mmol, 0.5 equiv) in a 40 ml capped vial equipped with a magnetic stirring bar. Dissolve in dry DCM (0.1 M) and add MTO (5 mol%) followed by UHP (2 equiv). Stir at 23 °C for 16 h and analyze the crude by UPLC-MS (5 μl of sample diluted in 195 μl of a 0.02 M solution of 4,4'-di-*tert*-butyl-1,1'-biphenyl in acetonitrile). Add Ts_2O (0.8 equiv), keep stirring for 2 h and analyze the crude by UPLC-MS again (54% conversion). Dilute the crude with DCM and quench with saturated aqueous $NaHCO_3$. Separate phases, concentrate under reduced pressure and directly purify the desired product by CombiFlash silica gel chromatography (DCM injection). Separation with heptane/85:15 ethyl acetate and 7N ammonia in methanol (0% to 100%) leads to 15% yield of product **3a** over the two steps with 48% deuterium incorporation (44 mg, 1 mmol). Repurify further with preparative-TLC using DCM/MeOH 1%.

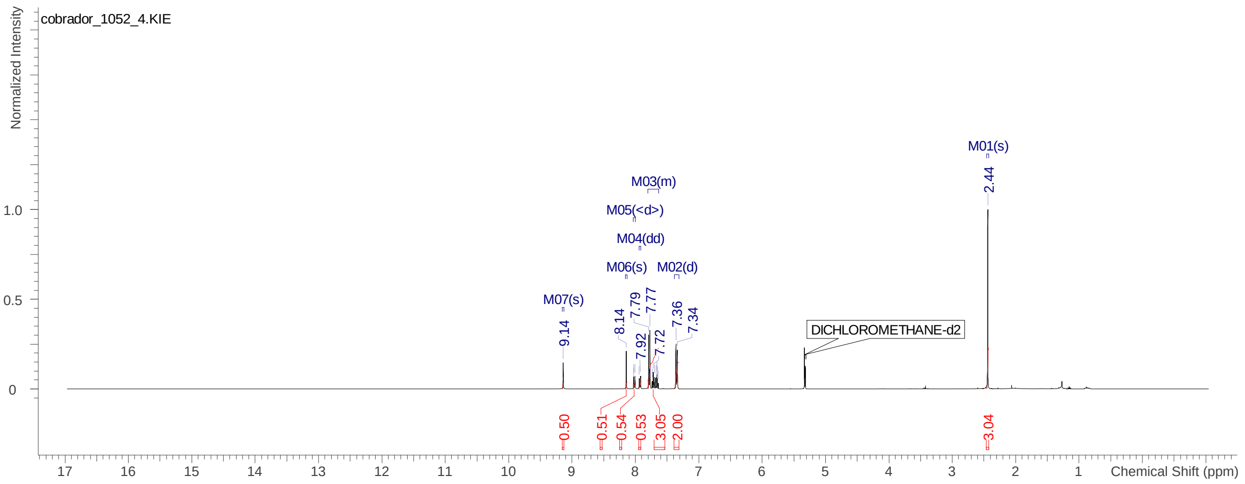

2. Kinetic analysis

Monitoring of the reaction *via* sampling over time followed by analysis of the crude by UPLC-MS: conditions as pictured (bold blue line); using TsCl (grey line); generation of the *N*-oxide *in situ* (yellow line); generation of the *N*-oxide *in situ* and usage of 5 equiv. of Ts_2O (bold green line); addition of 3 equiv. of H_2O to **1a** (red line).

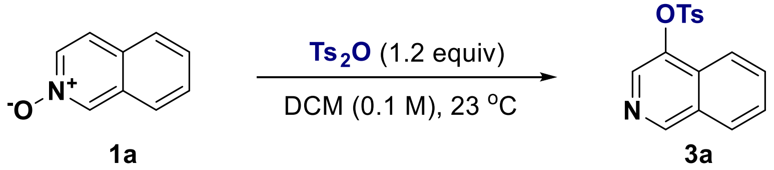

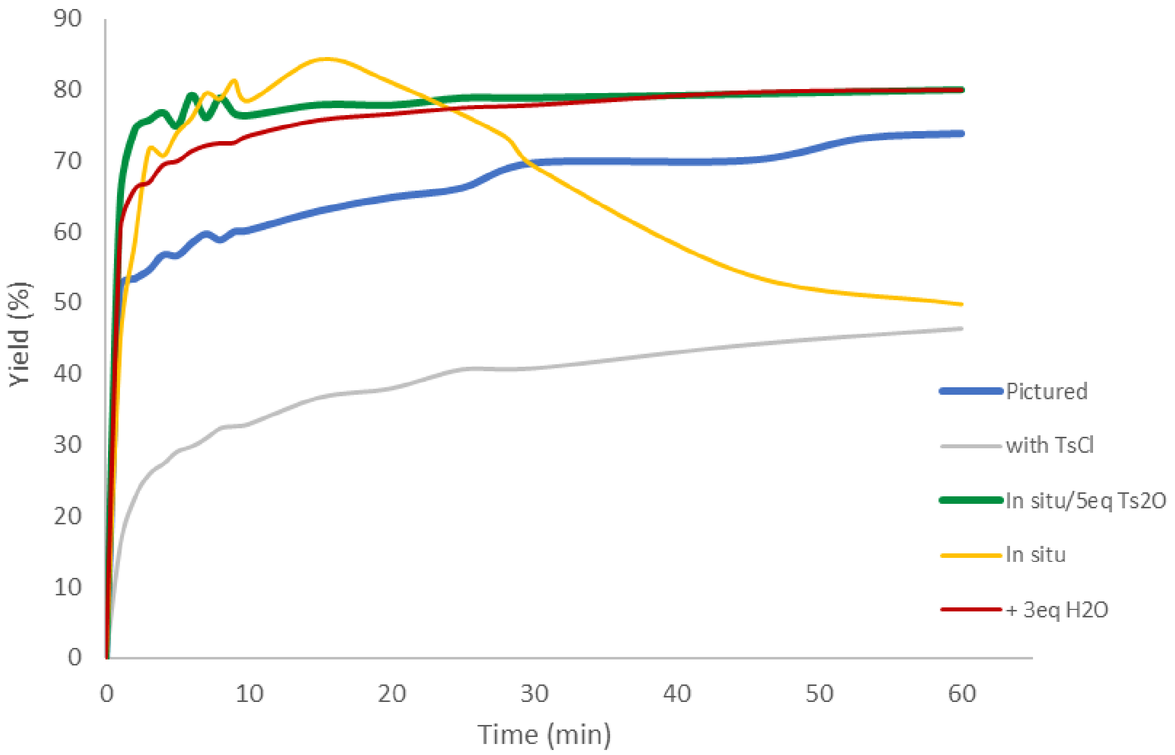

Standard conditions (bold blue line):

Time (min)	IS	Product	SM	Hydrolysis	Byproduct	Yield	Time (min)	IS	Product	SM	Hydrolysis	Byproduct	Yield	Time	Average
0	-	0	-	-	-	0	0	-	0	-	-	-	0	0	0
1	3.71E+1	6.73E+1	5.34E+1	8.23E+0	0	5.22E+1	1	4.36E+1	7.31E+1	5.80E+1	8.20E+0	0	5.25E+1	1	5.24E+1
2	3.81E+1	7.39E+1	5.31E+1	8.94E+0	0	5.43E+1	2	4.00E+1	7.48E+1	5.89E+1	8.79E+0	0	5.25E+1	2	5.34E+1
3	3.89E+1	7.68E+1	5.21E+1	9.48E+0	0	5.55E+1	3	3.75E+1	7.70E+1	5.69E+1	9.21E+0	0	5.38E+1	3	5.46E+1
4	4.12E+1	7.27E+1	4.33E+1	8.52E+0	0	5.84E+1	4	4.04E+1	7.82E+1	5.42E+1	9.28E+0	0	5.52E+1	4	5.68E+1
5	3.77E+1	7.75E+1	4.77E+1	9.19E+0	0	5.77E+1	5	3.89E+1	7.86E+1	5.31E+1	9.46E+0	0	5.57E+1	5	5.67E+1
6	4.09E+1	7.58E+1	4.15E+1	8.71E+0	0	6.01E+1	6	3.87E+1	7.51E+1	4.85E+1	8.87E+0	0	5.67E+1	6	5.84E+1
7	3.80E+1	7.81E+1	4.43E+1	9.35E+0	0	5.93E+1	7	3.80E+1	5.14E+1	2.86E+1	5.40E+0	0	6.02E+1	7	5.97E+1
8	3.87E+1	7.83E+1	4.26E+1	9.41E+0	0	6.01E+1	8	4.02E+1	9.09E+1	5.55E+1	1.13E+1	0	5.76E+1	8	5.89E+1
9	3.89E+1	7.48E+1	3.81E+1	8.71E+0	0	6.15E+1	9	3.88E+1	8.33E+1	4.84E+1	1.03E+1	0	5.87E+1	9	6.01E+1
10	3.84E+1	8.51E+1	4.38E+1	1.05E+1	0	6.11E+1	10	3.92E+1	7.99E+1	4.51E+1	9.42E+0	0	5.94E+1	10	6.03E+1
15	3.92E+1	8.22E+1	3.64E+1	1.01E+1	0	6.39E+1	15	3.90E+1	8.49E+1	4.13E+1	1.05E+1	0	6.21E+1	15	6.30E+1
20	4.36E+1	9.42E+1	3.70E+1	1.18E+1	0	6.58E+1	20	4.00E+1	8.83E+1	3.91E+1	1.07E+1	0	6.39E+1	20	6.49E+1
25	4.07E+1	9.15E+1	3.23E+1	1.15E+1	0	6.76E+1	25	3.98E+1	1.01E+2	4.18E+1	1.29E+1	0	6.48E+1	25	6.62E+1
30	4.21E+1	6.11E+1	1.66E+1	6.52E+0	0	7.26E+1	30	3.92E+1	9.20E+1	3.40E+1	1.14E+1	0	6.69E+1	30	6.97E+1
53	4.37E+1	1.03E+2	2.40E+1	1.38E+1	0	7.32E+1	45	4.03E+1	9.43E+1	2.80E+1	1.22E+1	0	7.01E+1	45	7.01E+1
60	4.14E+1	9.26E+1	1.93E+1	1.22E+1	0	7.46E+1	60	4.19E+1	9.25E+1	2.18E+1	1.21E+1	0	7.32E+1	53	7.32E+1
90	4.28E+1	1.05E+2	1.62E+1	1.49E+1	0	7.71E+1	90	4.16E+1	9.88E+1	1.72E+1	1.36E+1	0	7.62E+1	60	7.39E+1
120	4.33E+1	1.03E+2	1.22E+1	1.50E+1	0	7.92E+1	120	4.40E+1	9.87E+1	1.26E+1	1.44E+1	0	7.85E+1	90	7.67E+1
														120	7.88E+1

Use of TsCl (grey line):

Time (min)	IS	Product	SM	Hydrolysis	Byproduct	Yield	Time (min)	IS	Product	SM	Hydrolysis	Byproduct	Yield	Time (min)	Average
0	-	0	-	-	-	0	0	-	0	-	-	-	0	0	0
1	4.53E+1	3.52E+1	1.71E+2	7.81E+0	0	1.64E+1	1	3.84E+1	2.59E+1	1.37E+2	4.92E+0	0	1.54E+1	1	1.59E+1
2	5.32E+1	5.88E+1	1.78E+2	1.48E+1	0	2.33E+1	2	3.75E+1	4.02E+1	1.40E+2	6.69E+0	0	2.16E+1	2	2.25E+1
3	3.96E+1	4.90E+1	1.32E+2	8.93E+0	0	2.58E+1	3	3.96E+1	4.86E+1	1.32E+2	8.14E+0	0	2.58E+1	3	2.58E+1
4	3.92E+1	5.04E+1	1.29E+2	8.06E+0	0	2.68E+1	4	3.86E+1	4.88E+1	1.20E+2	7.76E+0	0	2.77E+1	4	2.73E+1
5	6.06E+1	7.53E+1	1.63E+2	1.73E+1	0	2.94E+1	5	3.73E+1	5.26E+1	1.24E+2	7.48E+0	0	2.86E+1	5	2.90E+1
6	4.04E+1	5.67E+1	1.27E+2	8.53E+0	0	2.95E+1	6	3.82E+1	5.29E+1	1.16E+2	7.80E+0	0	2.99E+1	6	2.97E+1
7	4.31E+1	6.41E+1	1.37E+2	9.62E+0	0	3.04E+1	7	3.85E+1	4.99E+1	1.03E+2	6.85E+0	0	3.13E+1	7	3.09E+1
8	4.00E+1	6.16E+1	1.22E+2	9.48E+0	0	3.19E+1	8	3.85E+1	5.82E+1	1.11E+2	8.53E+0	0	3.27E+1	8	3.23E+1
9	5.93E+1	9.07E+1	1.71E+2	2.12E+1	0	3.21E+1	9	3.90E+1	6.66E+1	1.25E+2	1.01E+1	0	3.31E+1	9	3.26E+1
10	6.34E+1	9.64E+1	1.78E+2	2.21E+1	0	3.25E+1	10	3.79E+1	6.34E+1	1.19E+2	8.66E+0	0	3.33E+1	10	3.29E+1
15	3.83E+1	6.28E+1	1.01E+2	8.11E+0	0	3.64E+1	15	3.95E+1	6.18E+1	9.75E+1	8.28E+0	0	3.69E+1	15	3.67E+1
20	3.97E+1	7.12E+1	1.05E+2	9.30E+0	0	3.84E+1	20	4.25E+1	1.12E+2	1.69E+2	1.81E+1	0	3.74E+1	20	3.79E+1
25	3.95E+1	4.02E+1	5.24E+1	4.27E+0	0	4.15E+1	25	3.98E+1	7.39E+1	1.03E+2	9.83E+0	0	3.96E+1	25	4.06E+1
30	3.90E+1	7.74E+1	1.06E+2	1.05E+1	0	3.99E+1	30	3.84E+1	6.85E+1	8.78E+1	8.53E+0	0	4.16E+1	30	4.07E+1
45	3.97E+1	7.09E+1	8.10E+1	9.24E+0	0	4.40E+1	45	3.93E+1	7.19E+1	8.15E+1	9.58E+0	0	4.41E+1	45	4.41E+1
60	3.99E+1	7.18E+1	7.49E+1	9.64E+0	0	4.59E+1	60	4.20E+1	7.78E+1	7.82E+1	1.04E+1	0	4.68E+1	60	4.63E+1
90	4.13E+1	8.84E+1	8.48E+1	1.30E+1	0	4.75E+1	90	4.52E+1	1.36E+2	1.36E+2	2.64E+1	0	4.56E+1	90	4.65E+1
120	4.25E+1	8.53E+1	7.27E+1	1.32E+1	0	4.98E+1	120	4.30E+1	9.16E+1	7.78E+1	1.38E+1	0	5.00E+1	120	4.99E+1

Generation of the N-oxide in situ (yellow line):

Time (min)	IS	Product	SM	Hydrolysis	Byproduct	Yield	Time (min)	IS	Product	SM	Hydrolysis	Byproduct	Yield	Time (min)	Average
0	-	0	-	-	-	0	0	-	0	-	-	-	0	0	0
1	5.62E+5	8.72E+5	1.93E+6	1.46E+5		2.96E+1	1	8.73E+1	1.35E+2	6.62E+1	2.61E+1		5.94E+1	1	44.50965
2	5.63E+5	9.92E+5	7.83E+5	1.52E+5		5.15E+1	2	8.85E+1	1.49E+2	4.92E+1	2.96E+1		6.54E+1	2	58.46386
3	5.62E+5	1.27E+6	2.64E+5	1.95E+5		7.35E+1	3	8.68E+1	1.57E+2	3.81E+1	3.02E+1		6.97E+1	3	71.61902
4	5.67E+5	1.18E+6	3.60E+5	1.77E+5		6.86E+1	4	8.61E+1	1.60E+2	3.01E+1	2.98E+1		7.27E+1	4	70.68984
5	5.84E+5	1.27E+6	2.69E+5	1.86E+5		7.36E+1	5	8.70E+1	1.57E+2	2.53E+1	2.85E+1		7.44E+1	5	74.03866
6	3.67E+5	7.12E+5	1.78E+5	1.12E+5		7.11E+1	6	8.73E+1	1.21E+2	1.05E+1	1.78E+1		8.10E+1	6	76.06626
7	5.07E+5	1.10E+6	1.04E+5	1.61E+5	2.93E+4	7.89E+1	7	8.63E+1	1.58E+2	1.27E+1	2.65E+1		8.02E+1	7	79.52824
8	3.35E+5	8.02E+5	8.85E+4	1.22E+5	2.19E+4	7.75E+1	8	8.66E+1	1.66E+2	1.35E+1	2.80E+1		8.00E+1	8	78.75669
9	1.12E+5	7.56E+5	7.68E+4	1.21E+5	2.10E+4	7.75E+1	9	8.66E+1	1.36E+2	3.93E+0	1.98E+1		8.51E+1	9	81.33368
10	4.39E+5	9.63E+5	1.74E+5	1.36E+5	2.72E+4	7.40E+1	10	8.71E+1	1.75E+2	6.91E+0	2.89E+1		8.30E+1	10	78.50703
15	4.73E+5	9.89E+5	5.08E+4	1.21E+5	2.69E+4	8.33E+1	16	8.77E+1	1.60E+2	3.90E+0	2.36E+1		8.53E+1	15	84.32779
20	1.89E+5	7.53E+5	4.54E+4	1.00E+5	2.21E+4	8.18E+1	20	8.71E+1	1.65E+2	4.08E+0	2.46E+1	1.18E+1	8.04E+1	20	81.0722
28	1.99E+5	5.77E+5	5.43E+4	7.58E+4	8.16E+4	7.32E+1	25	8.95E+1	1.58E+2	5.39E+0	2.22E+1	2.87E+1	7.37E+1	28	73.44209
30	1.22E+5	6.42E+5	6.51E+4	8.78E+4	1.34E+5	6.91E+1	30	8.92E+1	1.34E+2	4.31E+0	1.69E+1	3.75E+1	6.95E+1	30	69.27679
45	6.96E+4	3.51E+5	5.73E+4	5.25E+4	2.23E+5	5.13E+1	46	8.98E+1	1.60E+2	1.08E+1	2.22E+1	8.94E+1	5.67E+1	45	54.00119
60	2.51E+5	5.42E+5	9.81E+4	7.06E+4	4.98E+5	4.48E+1	60	9.21E+1	1.39E+2	8.58E+0	1.75E+1	8.87E+1	5.48E+1	60	49.80009
90	9.38E+4	3.47E+5	7.31E+4	4.56E+4	4.85E+5	3.65E+1	90	9.69E+1	1.51E+2	9.25E+0	2.00E+1	1.16E+2	5.09E+1	90	43.70789
120	8.56E+4	1.63E+5	4.07E+4	2.40E+4	2.85E+5	3.18E+1	120	9.92E+1	1.30E+2	7.86E+0	1.59E+1	9.69E+1	5.18E+1	120	41.82501

Generation of the N-oxide in situ and usage of 5 equiv. of Ts_2O (bold green line):

Time (min)	IS	Product	SM	Hydrolysis	Byproduct	Yield	Time (min)	IS	Product	SM	Hydrolysis	Byproduct	Yield	Time	Average
0	-	0	-	-	-	0	0	-	0	-	-	-	0	0	0
1	3.69E+2	9.69E+1	8.79E+0	2.34E+1		7.51E+1	1	8.45E+5	1.25E+6	6.80E+5	3.57E+5		5.47E+1	1	6.49E+1
2	3.73E+2	1.10E+2	4.85E+0	2.37E+1		7.94E+1	2	8.48E+5	1.21E+6	2.83E+5	2.57E+5		6.91E+1	2	7.43E+1
3	3.82E+2	8.27E+1		1.52E+1		8.45E+1	3	8.57E+5	1.63E+6	3.46E+5	4.15E+5	4.80E+4	6.68E+1	3	7.56E+1
4	3.75E+2	1.16E+2		2.46E+1		8.26E+1	4	8.52E+5	1.56E+6	2.13E+5	3.75E+5	5.40E+4	7.08E+1	4	7.67E+1
5	3.74E+2	1.25E+2	3.70E+0	2.76E+1		8.00E+1	5	8.70E+5	1.67E+6	2.17E+5	4.39E+5	7.12E+4	6.96E+1	5	7.48E+1
6	3.72E+2	1.05E+2	3.85E+0	2.09E+1		8.09E+1	6	8.48E+5	1.10E+6	6.22E+4	2.15E+5	4.33E+4	7.74E+1	6	7.92E+1
7	3.84E+2	1.15E+2		2.40E+1	3.93E+0	8.04E+1	7	8.58E+5	1.69E+6	1.58E+5	4.23E+5	9.25E+4	7.15E+1	7	7.60E+1
8	3.67E+2	9.01E+1		1.67E+1		8.44E+1	8	8.62E+5	1.41E+6	1.14E+5	3.25E+5	7.76E+4	7.32E+1	8	7.88E+1
9	3.71E+2	1.12E+2		2.29E+1	4.27E+0	8.04E+1	9	8.63E+5	1.50E+6	1.21E+5	3.52E+5	9.18E+4	7.27E+1	9	7.65E+1
10	3.77E+2	1.19E+2		2.51E+1	4.90E+0	7.99E+1	10	8.65E+5	1.60E+6	1.19E+5	3.76E+5	1.06E+5	7.27E+1	10	7.63E+1
15	3.68E+2	1.06E+2		2.12E+1	4.91E+0	8.03E+1	15	8.61E+5	1.41E+6	8.44E+4	2.87E+5	9.02E+4	7.53E+1	15	7.78E+1
20	3.76E+2	1.21E+2		2.46E+1	6.68E+0	7.94E+1	20	8.74E+5	1.55E+6	9.61E+4	2.96E+5	9.59E+4	7.61E+1	20	7.77E+1
25	3.74E+2	1.07E+2		2.11E+1	5.61E+0	8.00E+1	25	8.83E+5	1.47E+6	9.05E+4	2.53E+5	8.23E+4	7.75E+1	25	7.88E+1
30	3.75E+2	1.00E+2		1.86E+1	5.13E+0	8.09E+1	30	8.86E+5	1.75E+6	1.23E+5	3.07E+5	1.03E+5	7.67E+1	30	7.88E+1
45	3.75E+2	1.02E+2		2.10E+1	5.78E+0	7.92E+1	45	9.22E+5	1.57E+6	1.09E+5	2.20E+5	7.67E+4	7.94E+1	45	7.93E+1
60	3.80E+2	1.01E+2		2.03E+1	5.58E+0	7.96E+1	60	9.42E+5	1.67E+6	1.16E+5	2.17E+5	7.92E+4	8.02E+1	60	7.99E+1
90	3.93E+2	9.07E+1		1.52E+1	4.13E+0	8.24E+1	90	1.01E+6	1.80E+6	7.11E+4	2.21E+5	8.42E+4	8.27E+1	90	8.26E+1

*Addition of 3 equiv. of H_2O to **1a** (red line):*

Time (min)	IS	Product	SM	Hydrolysis	Byproduct	Impurity	Yield	Time (min)	IS	Product	SM	Hydrolysis	Byproduct	Impurity	Yield	Time (min)	Average
0	-	0	-	-	-	-	0	0	-	0	-	-	-	-	0	0	0
1	3.42E+2	8.81E+1	3.58E+1	1.11E+1	0	6.53E+0	6.22E+1	1	3.67E+2	9.52E+1	4.12E+1	1.47E+1	0	1.29E+1	5.80E+1	1	60.12929
2	3.44E+2	6.48E+1	1.58E+1	7.28E+0	0	6.47E+0	6.87E+1	2	3.76E+2	1.07E+2	3.19E+1	1.58E+1	0	1.42E+1	6.33E+1	2	66.00161
3	3.46E+2	9.72E+1	2.43E+1	1.26E+1	0	7.23E+0	6.88E+1	3	3.76E+2	9.51E+1	2.20E+1	1.30E+1	0	1.57E+1	6.52E+1	3	66.97729
4	3.51E+2	8.97E+1	1.83E+1	1.11E+1	0	7.31E+0	7.10E+1	4	3.70E+2	9.69E+1	1.80E+1	1.29E+1	0	1.49E+1	6.79E+1	4	69.44324
5	3.45E+2	1.02E+2	1.86E+1	1.36E+1	0	8.04E+0	7.16E+1	5	3.82E+2	9.95E+1	1.62E+1	1.35E+1	0	1.67E+1	6.83E+1	5	69.95075
6	3.49E+2	9.31E+1	1.47E+1	1.17E+1	0	7.70E+0	7.32E+1	6	3.68E+2	9.56E+1	1.40E+1	1.26E+1	0	1.53E+1	6.95E+1	6	71.36871
7	3.45E+2	9.39E+1	1.40E+1	1.19E+1	0	7.54E+0	7.38E+1	7	3.68E+2	1.02E+2	1.47E+1	1.33E+1	0	1.47E+1	7.05E+1	7	72.14178
8	3.44E+2	1.01E+2	1.50E+1	1.31E+1	0	7.83E+0	7.37E+1	8	3.77E+2	9.87E+1	1.23E+1	1.27E+1	0	1.49E+1	7.12E+1	8	72.46815
9	3.53E+2	1.01E+2	1.37E+1	1.29E+1	0	8.09E+0	7.45E+1	9	3.68E+2	6.72E+1	6.64E+0	7.55E+0	0	1.38E+1	7.06E+1	9	72.55166
10	3.36E+2	9.79E+1	9.41E+0	1.32E+1	0	8.19E+0	7.61E+1	10	3.82E+2	1.03E+2	9.66E+0	1.38E+1	0	1.86E+1	7.10E+1	10	73.5391
15	3.47E+2	9.23E+1	7.74E+0	1.15E+1	0	8.01E+0	7.72E+1	15	3.74E+2	9.99E+1	6.09E+0	1.29E+1	0	1.55E+1	7.43E+1	15	75.76051
20	3.59E+2	1.09E+2	6.94E+0	1.47E+1	0	9.17E+0	7.80E+1	20	3.75E+2	1.07E+2	5.25E+0	1.40E+1	0	1.59E+1	7.52E+1	20	76.59433
25	3.40E+2	1.04E+2	5.08E+0	1.38E+1	0	8.55E+0	7.91E+1	25	3.75E+2	1.09E+2	4.62E+0	1.43E+1	0	1.57E+1	7.58E+1	25	77.48037
30	3.55E+2	1.08E+2	4.52E+0	1.43E+1	0	8.49E+0	7.99E+1	30	3.81E+2	9.88E+1	3.10E+0	1.26E+1	0	1.56E+1	7.59E+1	30	77.88488
45	3.63E+2	7.65E+1		8.75E+0	0	8.44E+0	8.16E+1	45	3.81E+2	1.12E+2		1.51E+1	0	1.70E+1	7.77E+1	45	79.66318
60	3.53E+2	9.95E+1		1.27E+1	0	8.34E+0	8.25E+1	60	4.03E+2	1.04E+2		1.36E+1	0	1.67E+1	7.75E+1	60	80.00393
90	3.70E+2	8.15E+1		9.95E+0	0	9.76E+0	8.05E+1	90	3.79E+2	1.06E+2		1.41E+1	0	1.36E+1	7.92E+1	90	79.87789
120	3.76E+2	1.22E+2		1.79E+1	0	8.82E+0	8.20E+1	120	3.91E+2	1.17E+2		1.62E+1	0	1.22E+1	8.05E+1	120	81.26209

3. ^{1}H NMR monitoring

Downfield shift observed when mixing *N*-oxide **1a** and TsOH in CD_2Cl_2 (8.7 ppm to 9.68 ppm for H1). A similar intermediate is detected upon addition of Ts_2O (9.75 ppm) while product **3a·TsOH** is formed (9.79 ppm).

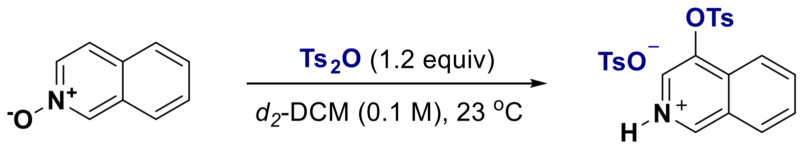

1a **3a·TsOH**

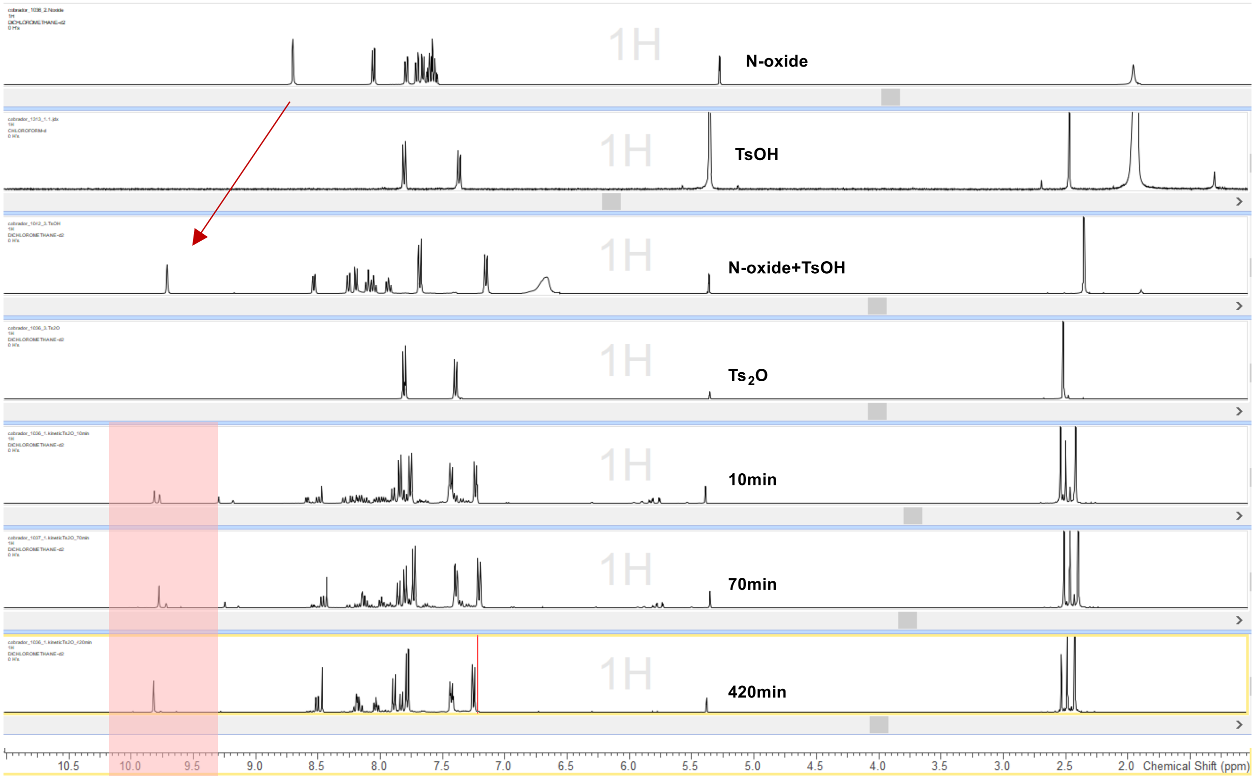

4. Computational analysis

Methods

Quantum chemical calculations were performed using Turbomole versions 7.6.1,[5] xTB 6.4,[6] CREST 2.11[7] and CENSO 1.0.8[8] program packages. Solvation free energies were calculated using COSMO-RS[9] theory as implemented in COSMOTherm version 2020.[10]

Conformers for all complexes were searched for at GFN2-xTB[11] level in dichloromethane for using the default settings of the CREST code unless otherwise noted. Free energies for the so-obtained low-energy ensembles were further refined at density functional theory level using the CENSO code together with Turbomole and COSMOTherm (R2SCAN-3c/def2-mTZVPP[12] in dichloromethane). In case of transition state, coordinates defining the forming and breaking bonds were kept frozen during the conformer sampling according to their initial guess structures identified using the single-ended growing string method.[13] The so-obtained lowest energy structures were optimized using TPSS-D3[14] functional with def2-TZVP[15] basis sets and solvation effects for dichloromethane were accounted for in the optimizations using conductor like screening model (COSMO) with dielectric constant of 8.93.[16]

[5]S. G. Balasubramani, G. P. Chen, S. Coriani, M. Diedenhofen, M. S. Frank, Y. J. Franzke, F. Furche, R. Grotjahn, M. E. Harding, C. Hättig, *J. Chem. Phys.* **2020**, *152*, 184107.

[6]C. Bannwarth, E. Caldeweyher, S. Ehlert, A. Hansen, P. Pracht, J. Seibert, S. Spicher, S. Grimme, *WIREs Computational Molecular Science* **2021**, *11*, e1493.

[7] a) S. J. Grimme, *Chem. Theory Comput.* **2019**, *15*, 2847; b) P. Pracht, F. Bohle, S. Grimme, *Phys. Chem. Chem. Phys.* **2020**, *22*, 7169.

[8]Grimme, S.; Bohle, F.; Hansen, A.; Pracht, P.; Spicher, S.; Stahn, M. *J. Phys. Chem. A* **2021**, *125*, 4039.

[9] a) A. Klamt, *J. Phys. Chem.* ***1995**, 99, 2224*; b) *A.* Klamt, V. Jonas, T. Bürger, J. C. Lohrenz, *J. Phys. Chem. A*, **1998**, 102, 5074; c) F. Eckert, A. Klamt, *AIChE Journal*, **2002**, *48*, 369.

[10]BIOVIA COSMOtherm, Dassault Systèmes, **2020**.

[11]C. Bannwarth, S. Ehlert, S. Grimme, *J. Chem. Theory Comput.* **2019**, *15*, 1652.

[12]S. Grimme, A. Hansen, S. Ehlert, J. Mewes, *J. Chem. Phys.* **2021,** *154*, 064103.

[13] a) P. Zimmerman, *J. Chem. Theory Comput.* **2013**, *9*, 3043; b) P. M. Zimmerman, *J. Chem. Phys.* **2013**, *138*, 184102; c) P. M. Zimmerman, *J. Comput. Chem.* **2015**, *36*, 601.

[14] a) J. Tao, J. Perdew, V. Staroverov, G. Scuseria, *Phys. Rev. Lett.* **2003**, *91*, 146401; b) S. Grimme, J. Antony, S. Ehrlich, H. Krieg, *J. Chem. Phys.* **2010**, *132*, 154104.

[15] a) F. Weigend, F. Furche, R. Ahlrichs, *J. Chem. Phys.* **2003**, *119*, 12753; b) F. Weigend, R. Ahlrichs, *Phys. Chem. Chem. Phys.* **2005**, *7*, 3297.

[16] a) A. Schäfer, A. Klamt, D. Sattel, J. W. C. Lohrenz, F. Eckert, *Phys. Chem. Chem. Phys.* **2000**, *2*, 2187; b) A. Klamt, G. Schüürmann, *J. Chem. Soc., Perkin Trans. 2*, **1993**, 799.

We further calculated the final energies for each optimized structure with higher level method RIRPA[17]/def2-QZVPP[18] using gas phase TPSS orbitals and keeping the core orbitals frozen in the RPA correlation energy calculation. All Turbomole calculations were sped-up using multipole-accelerated resolution-of-identity approximation for the Coulomb term (MARI-J[19]) and used together with the corresponding auxiliary basis sets.[20] Default settings of Turbomole were used throughout except finer integration grids of *m4* and *m5* were used in all optimizations and in RPA calculations, respectively. Higher threshold for energy convergence (scfconv 7) was also used throughout.

The solvation free energies were calculated in dichloromethane using COSMO-RS theory as implemented in COSMOTherm 20 using parameter file BP_TZVPD_20.ctd based on BP86[21]/def2-TZVPD[22] level. After, the free energies for each species were calculated according to G = E + c.p. + Gsolv unless otherwise noted, where E is energy calculated at RIRPA level, c.p. is the chemical potential calculated using standard RRHO approach at 25 °C, and Gsolv is solvation free energy obtained from COSMOTherm. The thermodynamic reference state of the free energy was further corrected to 1 mol/L for reactions with different number of educts and products from the hypothetic mole fraction of 1 used in COSMOtherm using solvent molarity of 15.6 mol/L for dichloromethane by adding term RTln(c) to the free energy of all species in the reaction equation.

[17]H. Eshuis, J. Yarkony, F. Furche, *J. Chem. Phys.* **2010**, *132*, 234114.
[18] a) F. Weigend, M. Häser, H. Patzelt, R. Ahlrichs, *Phys. Lett.* **1998**, *294*, 143; b) C. Hättig, *Phys. Chem. Chem. Phys.* **2005**, *7*, 59.
[19]M. Sierka, A. Hogekamp, R. Ahlrichs, *J. Chem..* **2003**, *118*, 9136.
[20]F. Weigend, *Phys. Chem. Chem. Phys.* **2006**, *8*, 1057.
[21]A. D. Becke, *Phys. Rev. A* **1988**, *38*, 3098.
[22]D. Rappoport, F. Furche, *J. Chem. Phys.* **2010**, 133, 134105.

Mechanism of the nucleophilic addition-tosyl migration step

We studied further the tosyl migration computationally to understand the nature of the predictable regioselectivity towards C4 observed, see below the reaction profile and the visualization of the optimized transition state **TS2-3**.

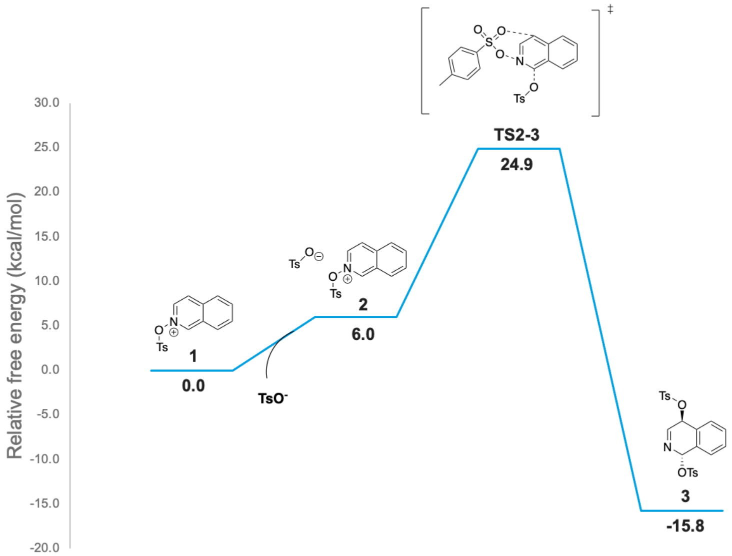

Computed free energies for the investigated reaction step

The activation free energy for the migration was calculated from the cationic tosyl activated intermediate **1** based on the chemical shifts of the major species observed by NMR monitoring of the reaction mixture. The transition state for the rearrangement starts from ion pair **2** to intermediate **3**. Both the nucleophilic addition of the tosylate anion to C1 and the migration happen in a concerted fashion *via* an asynchronous [3,3]-sigmatropic rearrangement and with a calculated activation free energy of 24.9 kcal/mol in dichloromethane. The nature of the transition was further investigated using Dynamic Reaction Coordinate calculations, which suggests that the nucleophilic addition to C1 initiates the rearrangement by elongating the N–O bond while approaching the sulfonate to C4 but does not form a stable intermediate. *Note: based on experimental evidence, the presence of electron donating groups may also trigger the migration to a lesser extent.*

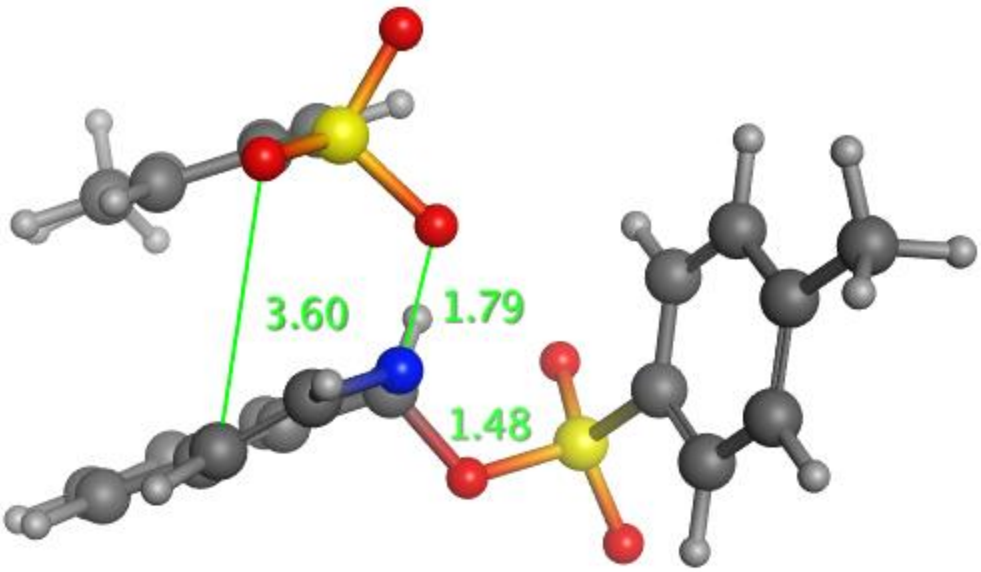

3D presentation of the optimized transition state ***TS2-3***

Cartesian coordinates of the optimized structures

Cartesian coordinates of all optimized complexes and raw data for reproducing the computational results is provided below.

1 (cationic heterocycle)
E(RPA) = -1296.43687126797
c.p. = 0.21121219109
Gsolv = -0.07420272041

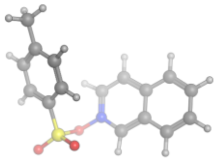

35
Charge = +1

C	-0.5968954	1.2421040	0.7076093
N	-0.8787238	-0.0471212	1.0951217
C	-1.4613430	1.8683434	-0.1432252
C	-1.9512556	-0.7404937	0.7044980
C	-2.8671674	-0.1425753	-0.1711685
C	-2.6212085	1.2016336	-0.6175299
H	0.3027751	1.6694151	1.1276327
H	-1.2469465	2.8864603	-0.4490476
H	-2.0506516	-1.7426415	1.1011452
C	-3.5449224	1.8034087	-1.5001446
C	-4.0187449	-0.8450117	-0.6126229
C	-4.8956055	-0.2259786	-1.4738040
C	-4.6568941	1.0987947	-1.9164555
H	-5.3647060	1.5648371	-2.5951203
H	-3.3696166	2.8186458	-1.8422566
H	-4.1903760	-1.8595051	-0.2667605

Tosylate anion
E(RPA) = -0.07420272041
c.p. = 0.08557275062
Gsolv = -0.10138295762

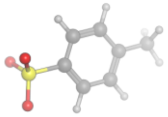

18
Charge = -1

C	0.0748504	-0.0210550	-0.0104096
S	1.8740015	-0.0059671	0.0059575
O	2.2409228	0.6007795	1.3033878
O	2.2897850	-1.4170079	-0.1190976
O	2.2666879	0.8281751	-1.1490915
C	-0.6203186	1.1945771	-0.0294291
C	-0.6336377	-1.2213897	0.0101812
C	-2.0319490	-1.2054463	0.0138817
C	-2.7432669	-0.0005452	-0.0018235
C	-2.0121280	1.1996403	-0.0250730
H	-2.5429026	2.1496245	-0.0418282
H	-0.0688632	2.1303562	-0.0519902
H	-2.5759318	-2.1478105	0.0278084
C	-4.2537580	0.0192080	0.0102795
H	-0.0894292	-2.1606808	0.0198123
H	-4.6632763	-0.9937569	-0.0454561
H	-4.6472189	0.5961615	-0.8350138
H	-4.6318693	0.4891846	0.9266364

H	-5.7792240	-0.7526337	-1.8195837
S	1.1852363	-1.6806010	1.1856547
O	0.4164250	-2.5290997	0.3077759
O	1.8723509	-2.1635629	2.3519970
C	2.1251669	-0.5274297	0.2711783
O	0.0085018	-0.6452070	1.9856458
C	1.8475979	-0.3506963	-1.0895010
C	3.0940499	0.2361088	0.9364614
C	3.7942514	1.1900397	0.2106525
H	3.2899550	0.0820951	1.9922239
C	3.5432466	1.3945197	-1.1584140
C	2.5647161	0.6122639	-1.7913705
H	2.3600403	0.7580625	-2.8481053
H	1.0942634	-0.9556550	-1.5821602
H	4.5498392	1.7875823	0.7127557
C	4.3320159	2.4163136	-1.9320018
H	4.5265337	3.3057302	-1.3251724
H	3.8119111	2.7129410	-2.8464915
H	5.3045791	1.9963178	-2.2189445

2
E(RPA) = -2191.34694630729
c.p. = 0.322603145968
Gsolv = -0.0539902171479552

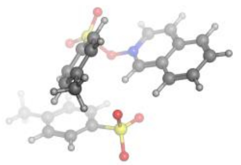

53
Charge = 0

C	0.3285274	0.8195326	-3.8515097
N	0.2765597	0.4199552	-2.5345614
C	1.2108845	1.8000888	-4.1974208
C	1.0383330	0.9131453	-1.5567814
C	1.9630336	1.9227029	-1.8679372
C	2.0579062	2.3906045	-3.2198926
H	-0.3485075	0.3085464	-4.5205926
H	1.2571786	2.1191746	-5.2328766
H	0.9127304	0.5266329	-0.5493624
C	2.9890342	3.4094966	-3.5191966
C	2.7918441	2.4667714	-0.8535713
C	3.6875782	3.4597803	-1.1830401
C	3.7828709	3.9305403	-2.5155344
H	4.4979756	4.7134902	-2.7502666
H	3.0723457	3.7728361	-4.5390369
H	2.7080769	2.0713738	0.1544563
H	4.3319615	3.8849570	-0.4200045
C	0.9044998	-2.2724352	1.1619772
S	2.2991101	-1.1544881	0.9968827
O	1.7450690	0.1767287	1.3500615
O	3.3230802	-1.6212796	1.9471835
O	2.7123733	-1.2319457	-0.4209629
S	-2.1984916	-0.0475738	-1.7826540
O	-2.8600106	-1.3177361	-1.6488752
O	-2.5556028	0.9265656	-2.7872357
O	-0.6047210	-0.6160174	-2.2208237
C	-1.6509669	2.1002313	-0.2168600
C	-1.7757939	-0.0780719	0.8978653
C	-1.4062813	0.5290041	2.0875589
H	-1.9496545	-1.1475610	0.8463714
C	-1.1299895	1.9048765	2.1507377
C	-1.2731387	2.6790067	0.9902908

TS2-3
E(RPA) = -2191.33188437697
c.p. = 0.32231371646
Gsolv = -0.0385525411729163
Imaginary frequency = -263.3 cm^{-1}

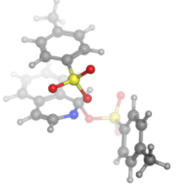

53
Charge = 0

C	-0.2105734	-0.0373992	-2.5318007
N	0.0480700	-0.6156064	-1.3308607
C	0.4758904	1.0674758	-2.9636894
C	1.0166029	-0.0273882	-0.4244651
C	1.5620837	1.3234757	-0.7886962
C	1.3345215	1.8160142	-2.0892162
H	-0.9021639	-0.5757779	-3.1698102
H	0.3861974	1.3613836	-4.0056639
H	0.5823673	-0.0057754	0.5765654
C	1.8778184	3.0631564	-2.4544998
C	2.2714448	2.0771642	0.1409329
C	2.8051197	3.3120806	-0.2334387
C	2.6075773	3.8019749	-1.5298727
H	3.0238802	4.7639192	-1.8139418
H	1.7017668	3.4489214	-3.4549022
H	2.4328407	1.6895453	1.1429219
H	3.3845972	3.8876676	0.4819840
C	1.0200400	-3.2128556	0.5305355
S	2.2593907	-2.0015315	0.8506875
O	1.9173851	-1.2757705	2.0607152
O	3.5767602	-2.5733558	0.6809898
O	2.1613683	-0.9659708	-0.4044785
S	-2.5132387	0.3504086	-0.3718173
O	-3.5391429	-0.1371082	0.5400567
O	-2.8909964	0.6889505	-1.7337841
O	-1.4313589	-0.7978336	-0.3456008
C	-1.4685705	2.8902684	-0.4252427
C	-1.2670686	1.6787063	1.6746904
C	-0.5846297	2.7583126	2.2219027
H	-1.4585625	0.7882489	2.2660183
C	-0.3327917	3.9167308	1.4661281
C	-0.7911186	3.9672054	0.1448279

H	-1.2977117	-0.0819647	2.9779711	H	-0.2343634	2.7005433	3.2493061
C	-0.6552511	2.5170519	3.4400304	C	0.4235675	5.0714450	2.0709834
H	-1.0729514	3.7460653	1.0262624	H	-0.5979625	4.8510265	-0.4567233
H	-1.7531600	2.6974537	-1.1164941	H	-1.8032990	2.9244739	-1.4561462
H	0.2997817	2.0645899	3.7325294	H	1.3531815	4.7247762	2.5358314
H	-1.3686313	2.3249305	4.2493858	H	-0.1708851	5.5557186	2.8551780
H	-0.5145639	3.5965686	3.3447665	H	0.6712284	5.8221783	1.3159695
C	0.3271776	-2.8603404	0.0352014	C	1.2437446	-4.1656724	-0.4694352
C	0.3494974	-2.4957796	2.4248284	C	-0.1725469	-3.1850345	1.2542130
C	-0.8137692	-3.6509763	0.1712660	C	0.2508183	-5.0995640	-0.7379604
C	-1.3961219	-3.8730684	1.4271626	C	-0.9603868	-5.0991279	-0.0246624
C	-0.7903226	-3.2886457	2.5502148	C	-1.1532388	-4.1319607	0.9701869
H	0.7649108	-2.6836643	-0.9410231	H	2.1767945	-4.1719768	-1.0235166
H	-1.2658947	-4.0898903	-0.7151449	H	0.4155083	-5.8418185	-1.5148226
C	-2.6411227	-4.7170354	1.5603072	C	-2.0176491	-6.1348337	-0.3121468
H	-1.2219869	-3.4514226	3.5353471	H	-2.0865065	-4.1109102	1.5263831
H	0.8004744	-2.0412782	3.3022701	H	-0.3312637	-2.4333847	2.0189523
H	-3.4524002	-4.3161020	0.9412297	H	-2.0685963	-6.3602340	-1.3819879
H	-2.9866696	-4.7537279	2.5973780	H	-3.0025739	-5.8010949	0.0261148
H	-2.4556066	-5.7444478	1.2249238	H	-1.7834071	-7.0715497	0.2098101
C	-1.8898526	0.7205608	-0.2476714	C	-1.6995495	1.7508658	0.3445027

4
E(RPA) = -2191.4004240179
c.p. = 0.327014802472
Gsolv = -0.0395462470732243

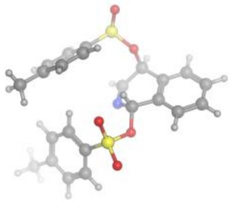

53
Charge = 0

C	-0.5446376	-0.2447862	1.9424548
N	0.4434924	-0.1250140	1.1473339
C	-1.3044572	-1.5406378	2.1479782
C	0.8252142	-1.3007387	0.3870654
C	0.7016883	-2.6021565	1.1453587
C	-0.3725198	-2.7065971	2.0392018
H	-0.8561356	0.6288850	2.5146451
H	-1.8753895	-1.5346411	3.0765685
H	0.2067485	-1.3252253	-0.5202533
C	-0.5594472	-3.8747807	2.7820514
C	1.5821627	-3.6722663	0.9852355
C	1.3902876	-4.8392897	1.7278071
C	0.3245508	-4.9419554	2.6262647
H	0.1836352	-5.8512263	3.2031193
H	-1.3943835	-3.9446656	3.4740213
H	2.4159553	-3.5891999	0.2963952
H	2.0804182	-5.6695286	1.6081053
C	2.6746815	1.3597304	-0.8415142
S	2.5553500	-0.3176373	-1.3759168
O	1.4360321	-0.4516595	-2.2898471
O	3.8693804	-0.7998819	-1.7387062
O	2.2158763	-1.1566866	-0.0247123
S	-3.4987710	-0.6733907	0.8508376
O	-4.5083198	-1.4303756	0.1425246
O	-2.2928784	-1.7389617	1.0307766
C	-2.8222595	0.5481849	-0.2354989
O	-3.7957981	-0.0667669	2.1348271
C	-2.7763379	1.8825410	0.1658159
C	-2.3769211	0.1542411	-1.5032401
C	-1.8875014	1.1196351	-2.3734389

C -1.8471836 2.4765924 -2.0055695<br>C -2.2914471 2.8377074 -0.7274692<br>H -3.1185061 2.1679281 1.1548247<br>H -2.4150492 -0.8888141 -1.8012993<br>H -1.5284371 0.8174690 -3.3533566<br>C -1.3264126 3.5103883 -2.9715240<br>H -2.2600225 3.8803091 -0.4224138<br>H -1.2977723 4.5032692 -2.5152550<br>H -1.9624440 3.5587779 -3.8630788<br>H -0.3159217 3.2533509 -3.3092195<br>C 3.8955220 1.8498771 -0.3724211<br>C 1.5340472 2.1634442 -0.8734257<br>C 1.6239728 3.4793257 -0.4346874<br>H 0.5937809 1.7567308 -1.2249564<br>C 2.8361165 4.0048494 0.0388411<br>C 3.9643398 3.1705226 0.0643023<br>H 4.9109452 3.5595034 0.4303239<br>H 4.7725470 1.2114514 -0.3539736<br>H 0.7375358 4.1081442 -0.4545151<br>C 2.9292952 5.4422196 0.4843612<br>H 3.1596036 6.0899389 -0.3713439<br>H 3.7226879 5.5775272 1.2251747<br>H 1.9827796 5.7859803 0.9123292	

VIII. NMR Spectra

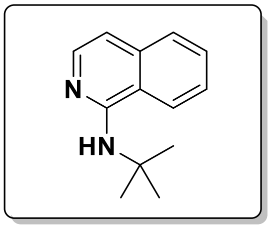

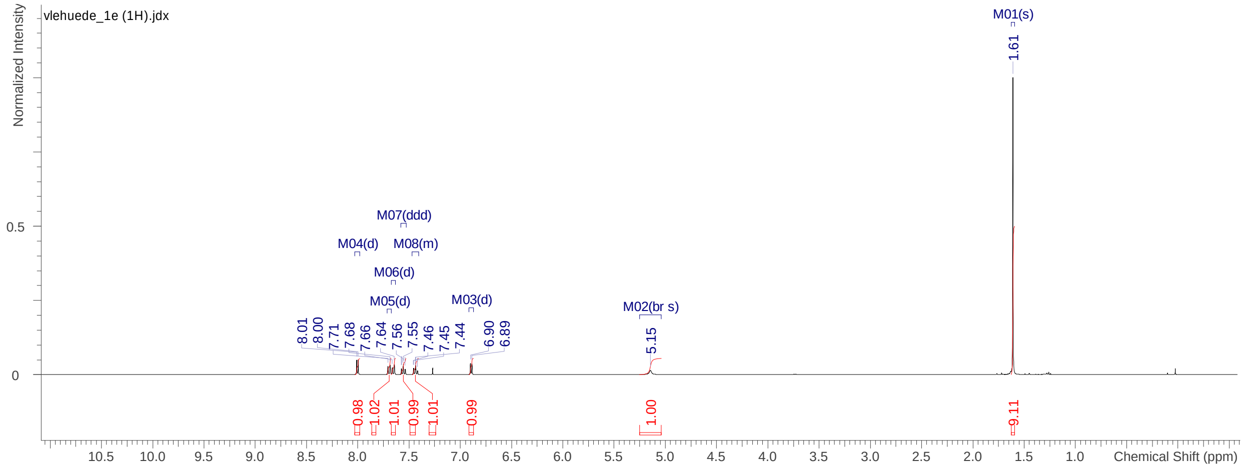

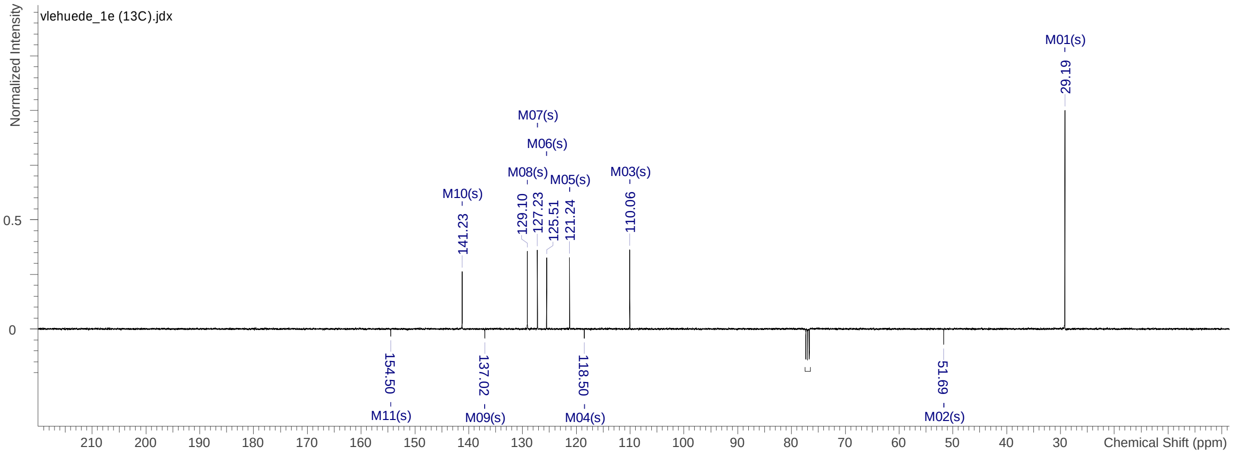

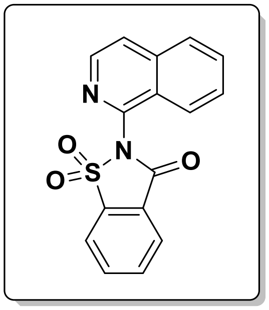
N
O
O
S
N
O

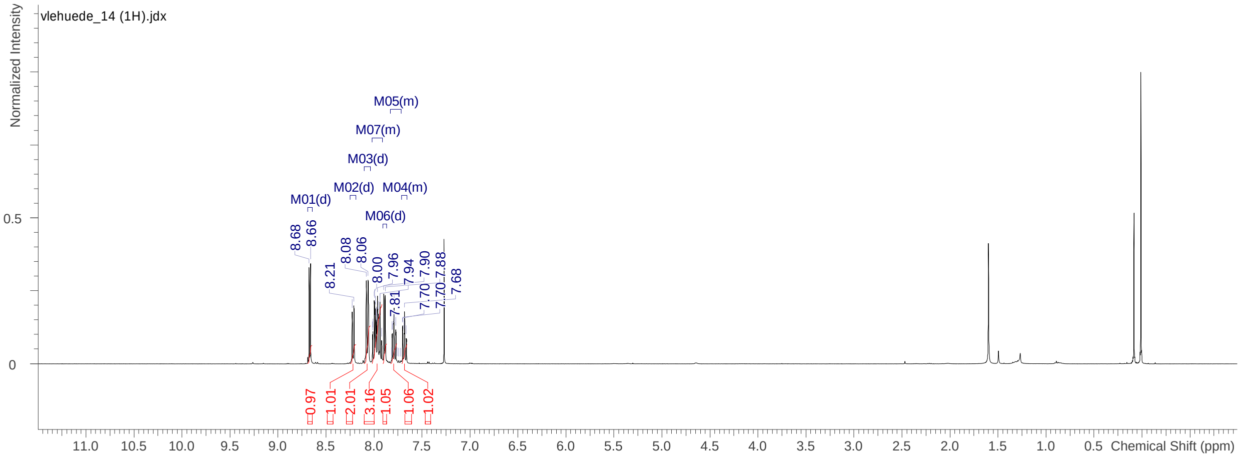
vlehuede_14 (1H).jdx
Normalized Intensity
Chemical Shift (ppm)
M01(d)
M02(d)
M03(d)
M04(m)
M05(m)
M06(d)
M07(m)
8.68
8.66
8.21
8.08
8.06
8.00
7.96
7.94
7.90
7.88
7.81
7.70
7.70
7.68
0.97
1.01
2.01
3.16
1.05
1.06
1.02

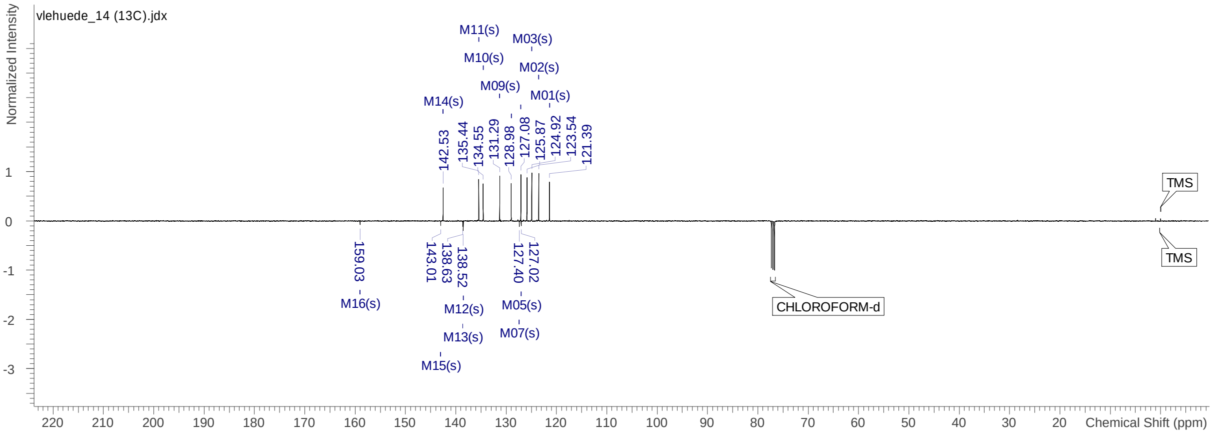
vlehuede_14 (13C).jdx
Normalized Intensity
Chemical Shift (ppm)
M01(s)
M02(s)
M03(s)
M05(s)
M07(s)
M09(s)
M10(s)
M11(s)
M12(s)
M13(s)
M14(s)
M15(s)
M16(s)
142.53
135.44
134.55
131.29
128.98
127.08
125.87
124.92
123.54
121.39
159.03
143.01
138.63
138.52
127.40
127.02
TMS
TMS
CHLOROFORM-d

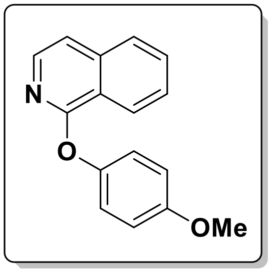
N
O
OMe

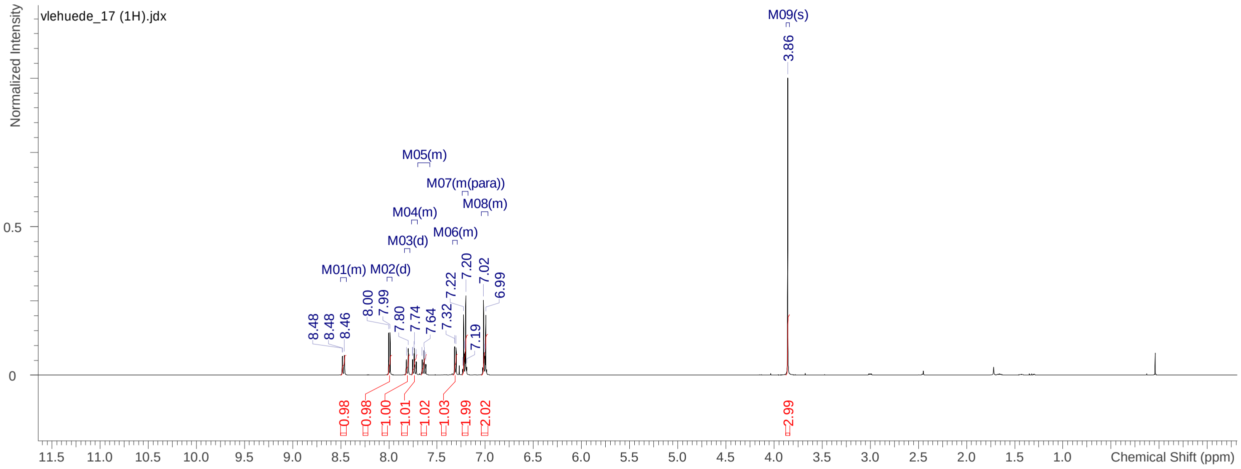
vlehuede_17 (1H).jdx
Normalized Intensity
M09(s)
3.86
M05(m)
M07(m(para))
M08(m)
M04(m)
M06(m)
M03(d)
M01(m) M02(d)
8.48
8.48
8.46
8.00
7.99
7.80
7.74
7.64
7.32
7.22
7.20
7.19
7.02
6.99
0.98
0.98
1.00
1.01
1.02
1.03
1.99
2.02
2.99
Chemical Shift (ppm)

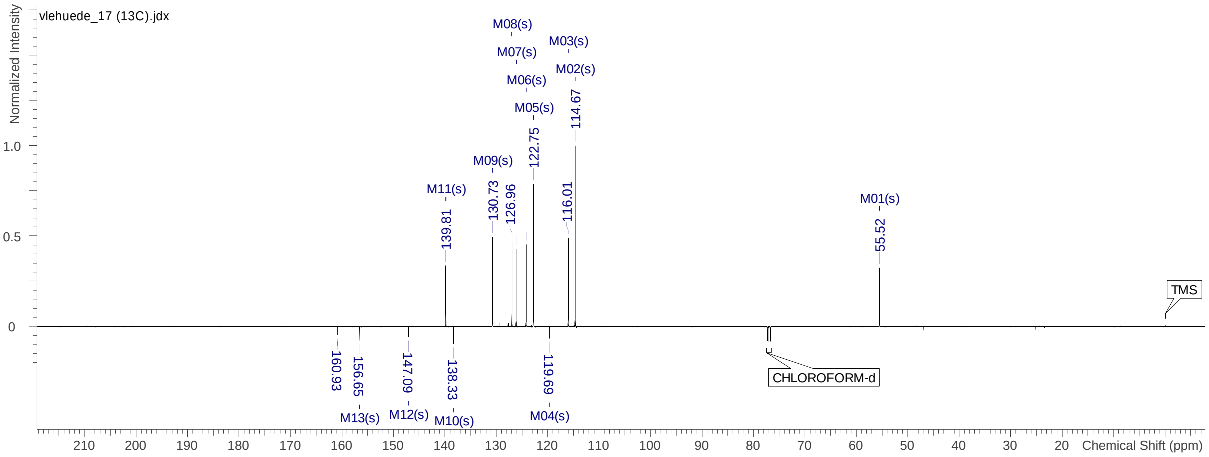
vlehuede_17 (13C).jdx
Normalized Intensity
M08(s)
M07(s)
M03(s)
M02(s)
M06(s)
M05(s)
M09(s)
M11(s)
M01(s)
139.81
130.73
126.96
122.75
116.01
114.67
55.52
TMS
160.93
156.65
147.09
138.33
119.69
M13(s)
M12(s)
M10(s)
M04(s)
CHLOROFORM-d
Chemical Shift (ppm)

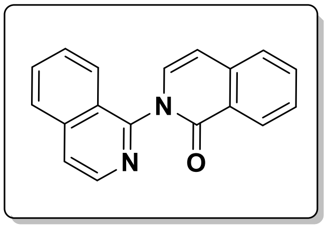
N
N
O

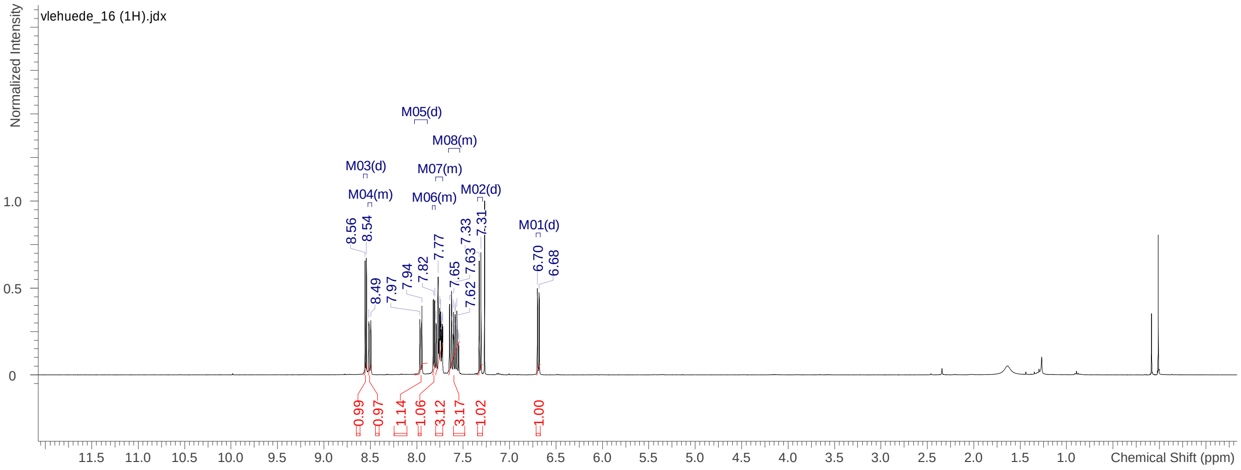
vlehuede_16 (1H).jdx
Normalized Intensity
M05(d)
M08(m)
M03(d)
M07(m)
M04(m)
M06(m)
M02(d)
M01(d)
8.56
8.54
8.49
7.97
7.94
7.82
7.77
7.65
7.62
7.63
7.33
7.31
6.70
6.68
0.99
0.97
1.14
1.06
3.12
3.17
1.02
1.00
Chemical Shift (ppm)

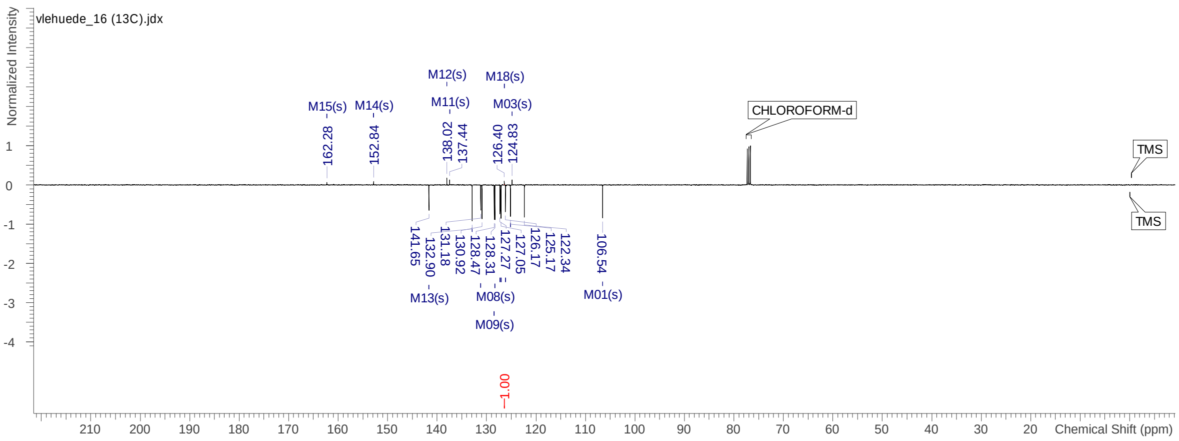
vlehuede_16 (13C).jdx
Normalized Intensity
M12(s)
M18(s)
M11(s)
M03(s)
M15(s)
M14(s)
162.28
152.84
138.02
137.44
126.40
124.83
CHLOROFORM-d
TMS
141.65
132.90
131.18
130.92
128.47
128.31
127.27
127.05
126.17
125.17
122.34
106.54
M13(s)
M08(s)
M09(s)
M01(s)
TMS
-1.00
Chemical Shift (ppm)

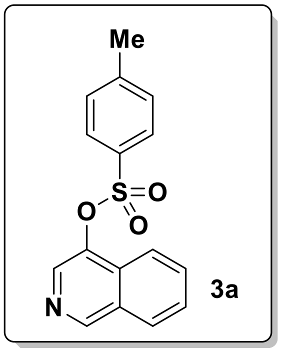
Me
S
O
O
O
N

3a

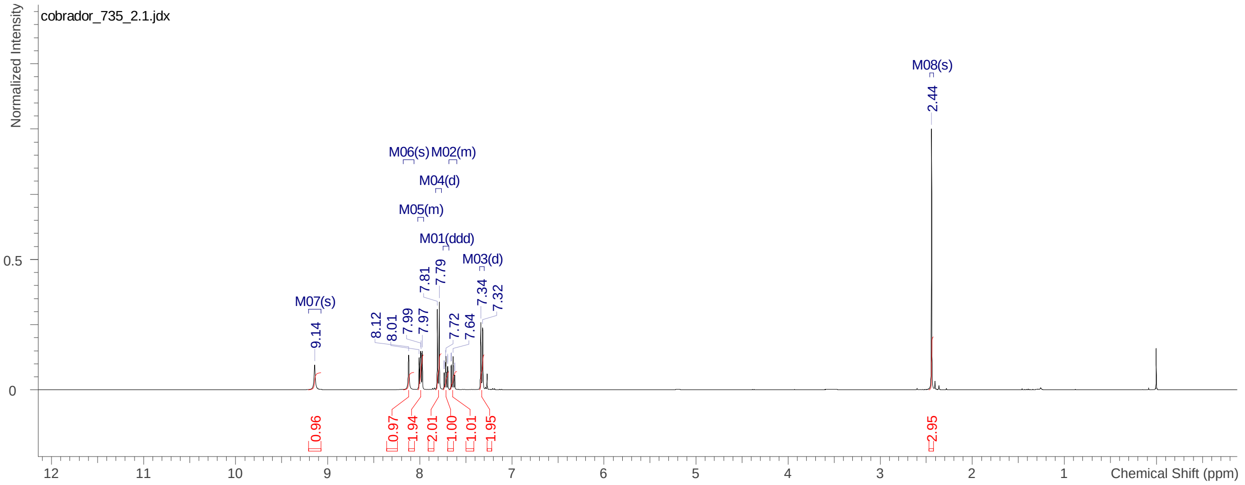
cobrador_735_2.1.jdx
Normalized Intensity
M08(s)
2.44
M06(s) M02(m)
M04(d)
M05(m)
M01(ddd)
M03(d)
M07(s)
9.14
8.12
8.01
7.99
7.97
7.81
7.79
7.72
7.64
7.34
7.32
0.96
0.97
1.94
2.01
1.00
1.01
1.95
2.95
0.5
0
12
11
10
9
8
7
6
5
4
3
2
1
Chemical Shift (ppm)

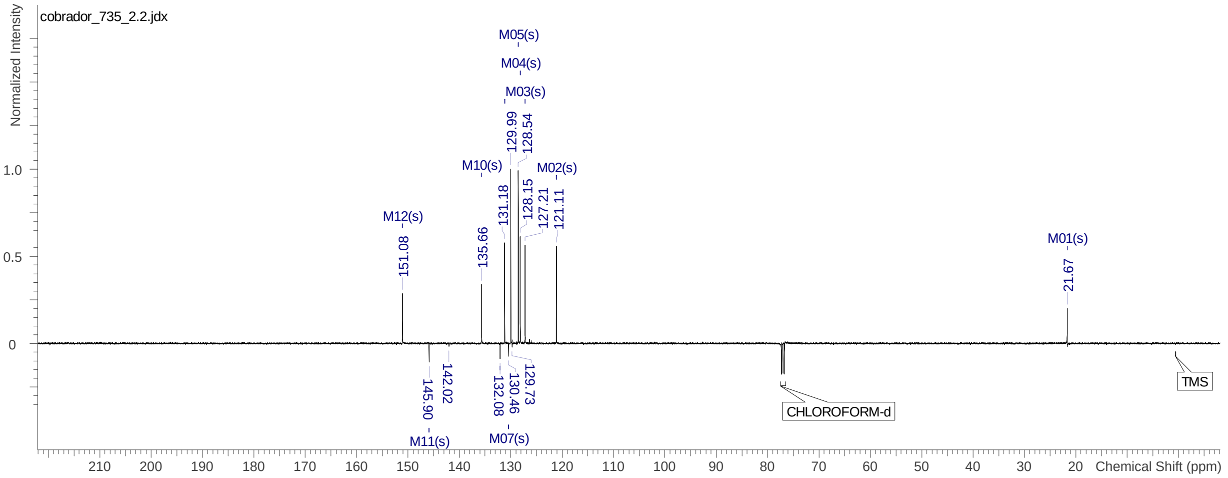
cobrador_735_2.2.jdx
Normalized Intensity
M05(s)
M04(s)
M03(s)
129.99
128.54
M10(s)
M02(s)
131.18
128.15
127.21
121.11
M12(s)
151.08
135.66
M01(s)
21.67
145.90
142.02
132.08
130.46
129.73
M11(s)
M07(s)
CHLOROFORM-d
TMS
1.0
0.5
0
210
200
190
180
170
160
150
140
130
120
110
100
90
80
70
60
50
40
30
20
Chemical Shift (ppm)

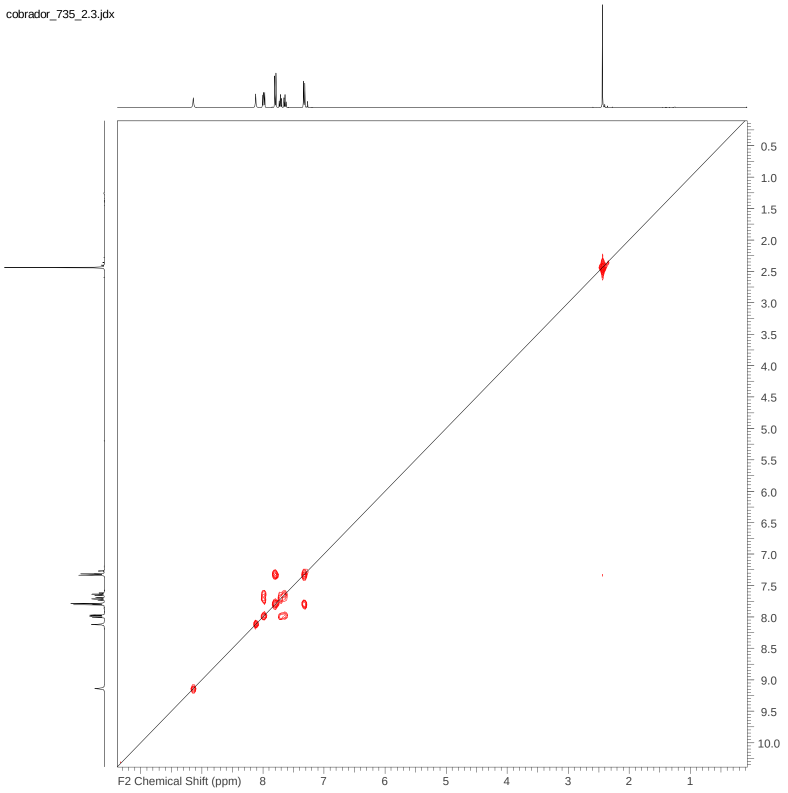
cobrador_735_2.3.jdx
F2 Chemical Shift (ppm)

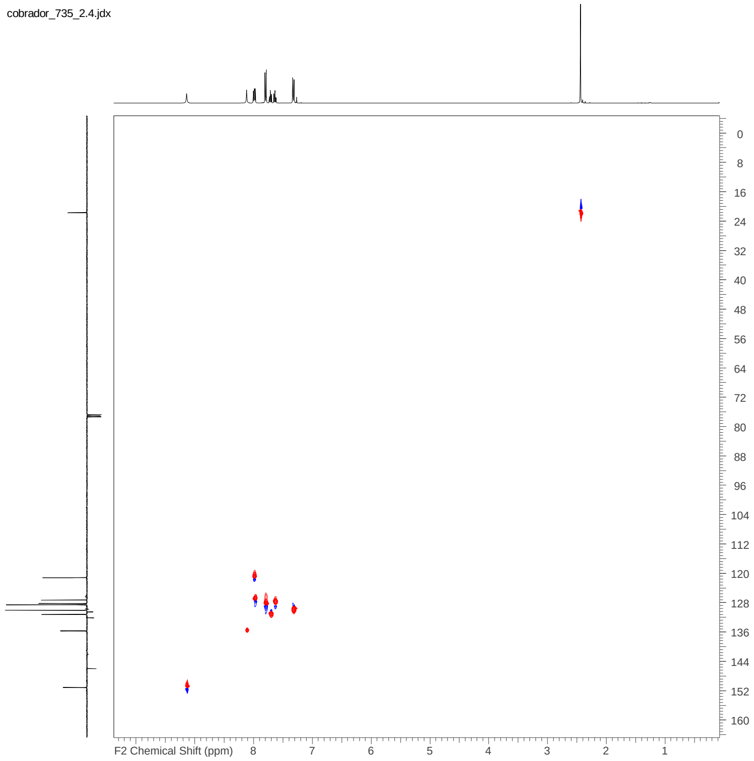
cobrador_735_2.4.jdx
F2 Chemical Shift (ppm)

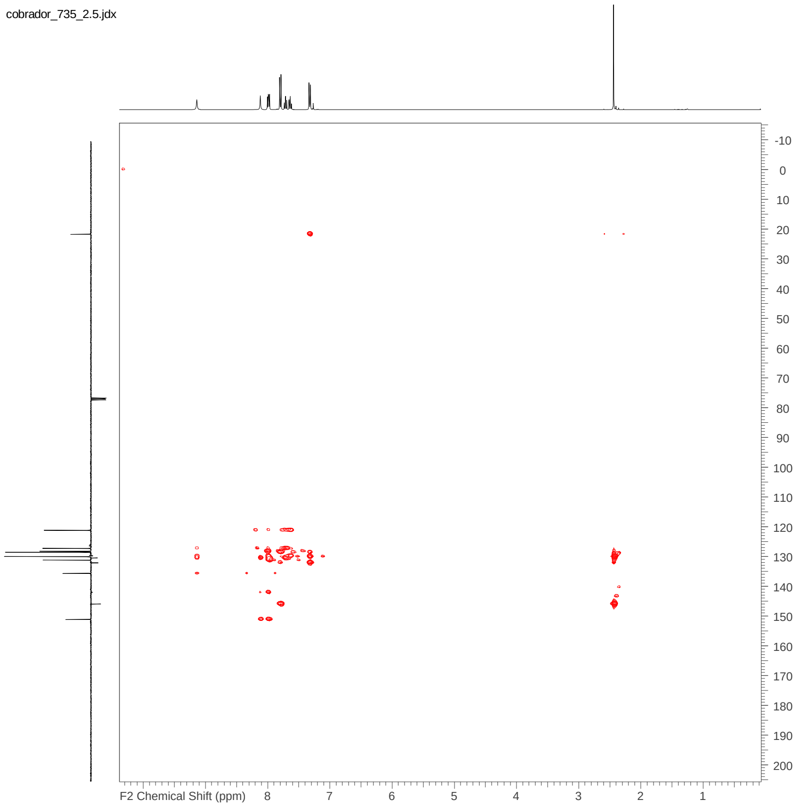
cobrador_735_2.5.jdx
F2 Chemical Shift (ppm)
8
7
6
5
4
3
2
1
-10
0
10
20
30
40
50
60
70
80
90
100
110
120
130
140
150
160
170
180
190
200

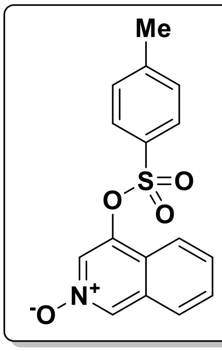
Me
S
O
O
O
N+
-O

12

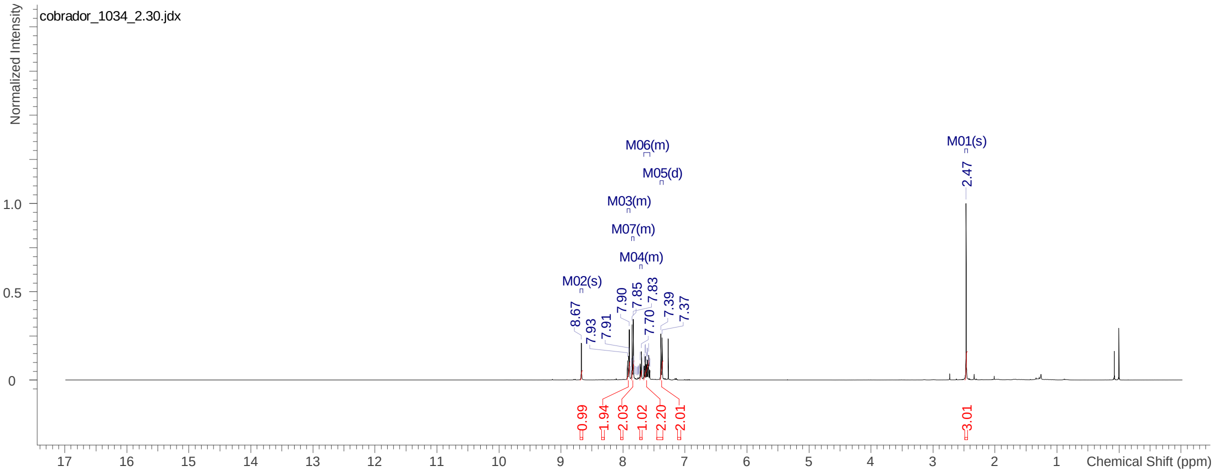
cobrador_1034_2.30.jdx
Normalized Intensity
M06(m)
M05(d)
M03(m)
M07(m)
M04(m)
M02(s)
M01(s)
2.47
8.67
7.93
7.91
7.90
7.85
7.70
7.83
7.39
7.37
0.99
1.94
2.03
1.02
2.20
2.01
3.01
Chemical Shift (ppm)

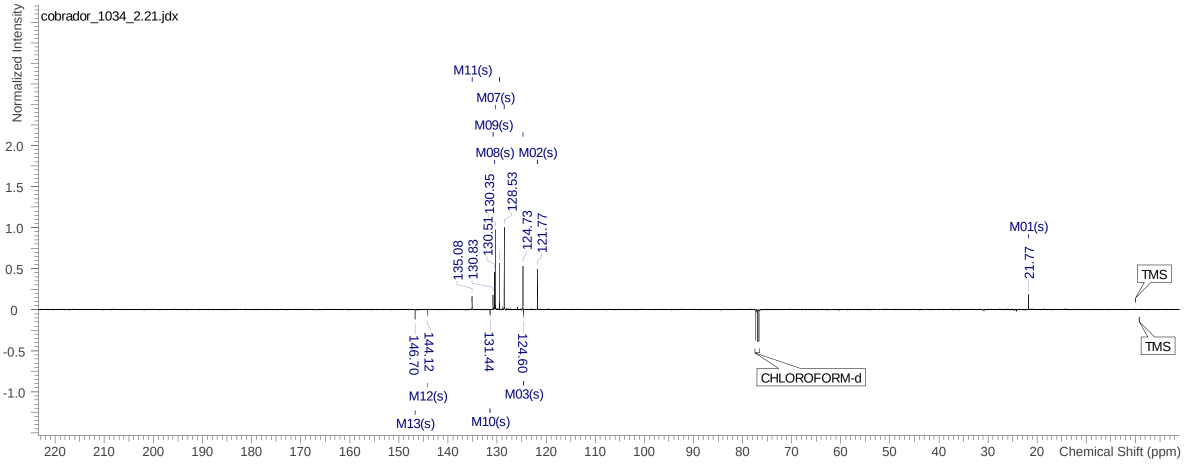
cobrador_1034_2.21.jdx
Normalized Intensity
M11(s)
M07(s)
M09(s)
M08(s)
M02(s)
M01(s)
135.08
130.83
130.51
130.35
128.53
124.73
121.77
21.77
146.70
144.12
131.44
124.60
M12(s)
M13(s)
M10(s)
M03(s)
TMS
CHLOROFORM-d
Chemical Shift (ppm)

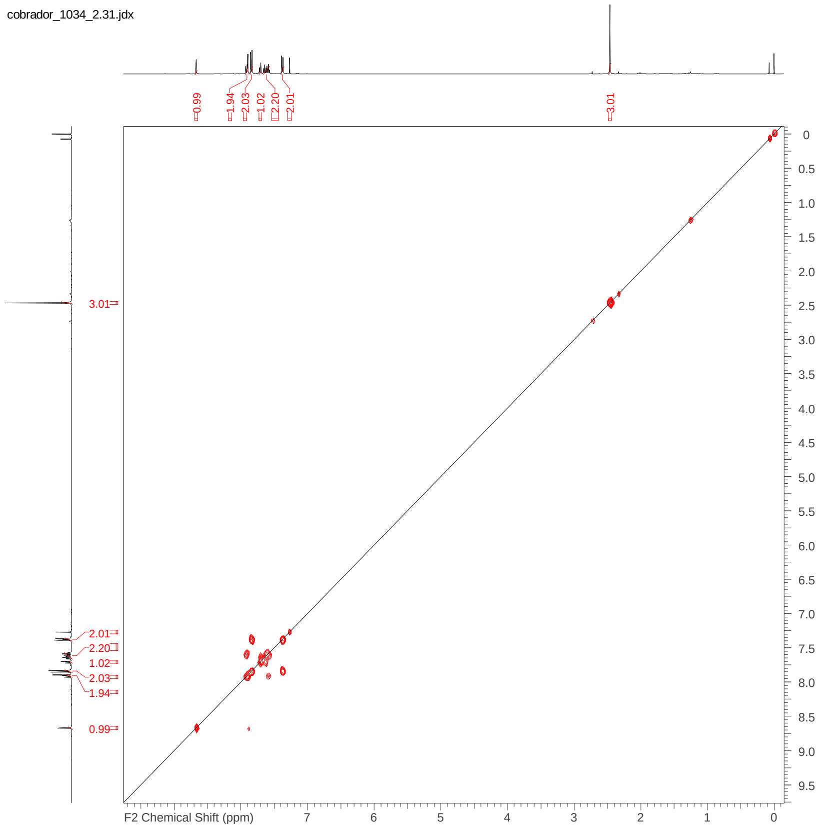
cobrador_1034_2.31.jdx
0.99
1.94
2.03
1.02
2.20
2.01
3.01
F2 Chemical Shift (ppm)

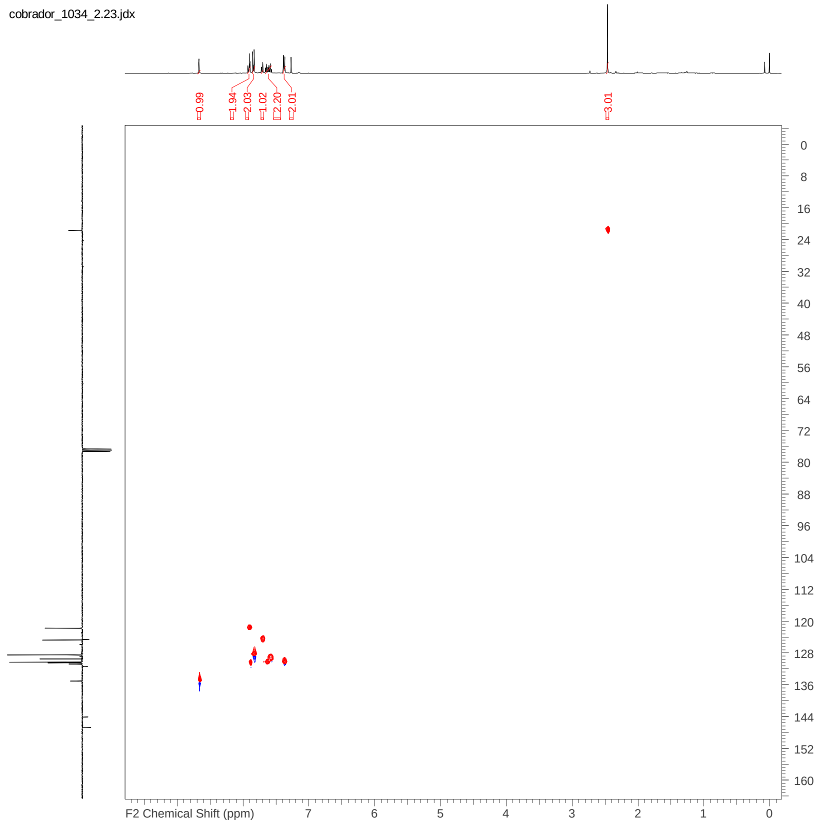
cobrador_1034_2.23.jdx
0.99
1.94
2.03
1.02
2.20
2.01
3.01
F2 Chemical Shift (ppm)

cobrador_1034_2.32.jdx

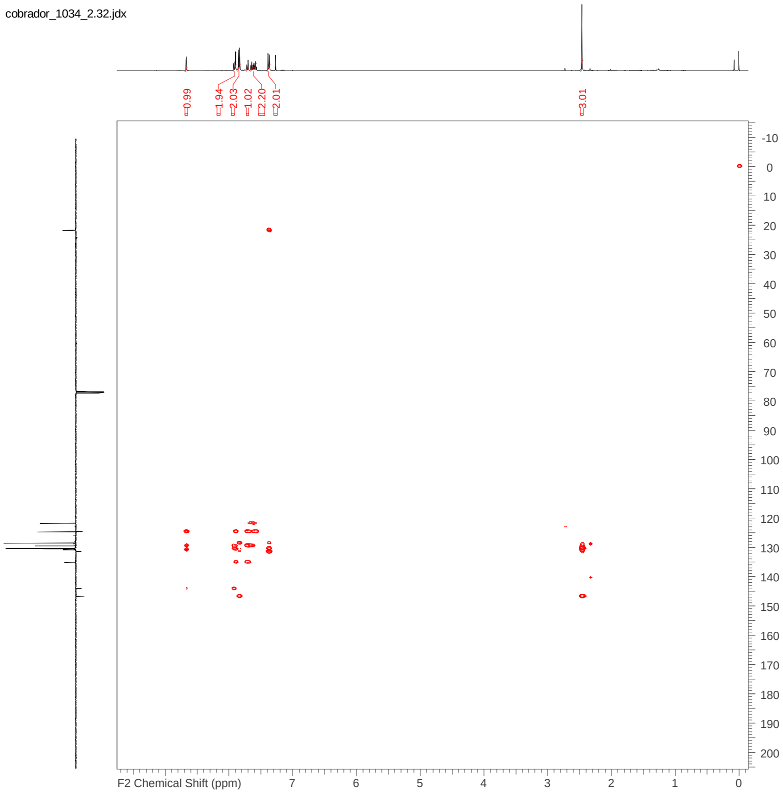

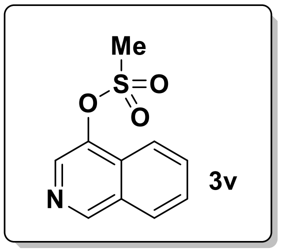
Me
S
O
O
O
N
3v

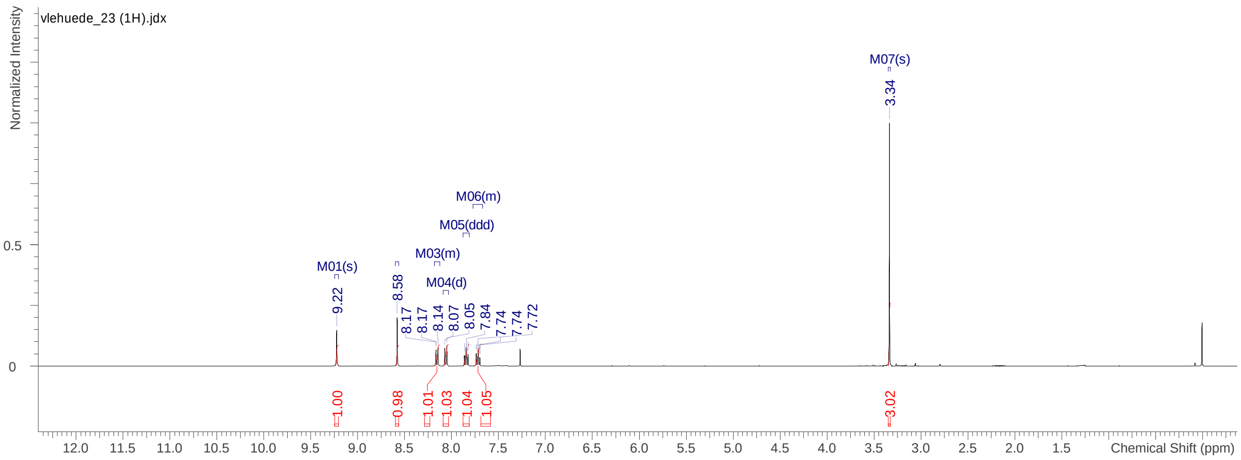
vlehuede_23 (1H).jdx
Normalized Intensity
M07(s)
3.34
M06(m)
M05(ddd)
M03(m)
M04(d)
M01(s)
9.22
8.58
8.17
8.17
8.14
8.07
8.05
7.84
7.74
7.74
7.72
1.00
0.98
1.01
1.03
1.04
1.05
3.02
Chemical Shift (ppm)

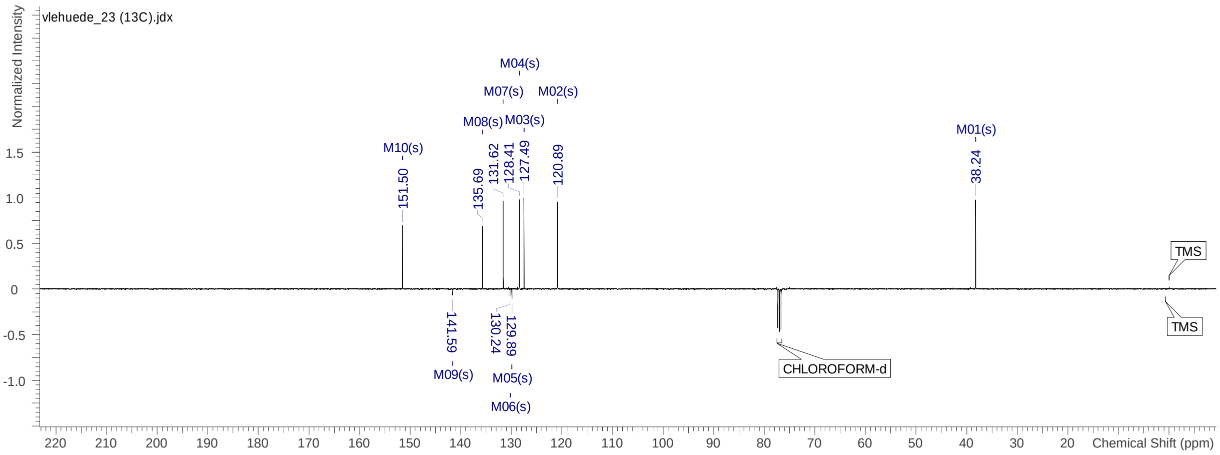
vlehuede_23 (13C).jdx
Normalized Intensity
M04(s)
M07(s)
M02(s)
M08(s)
M03(s)
M10(s)
M01(s)
151.50
135.69
131.62
128.41
127.49
120.89
38.24
141.59
130.24
129.89
M09(s)
M05(s)
M06(s)
CHLOROFORM-d
TMS
TMS
Chemical Shift (ppm)

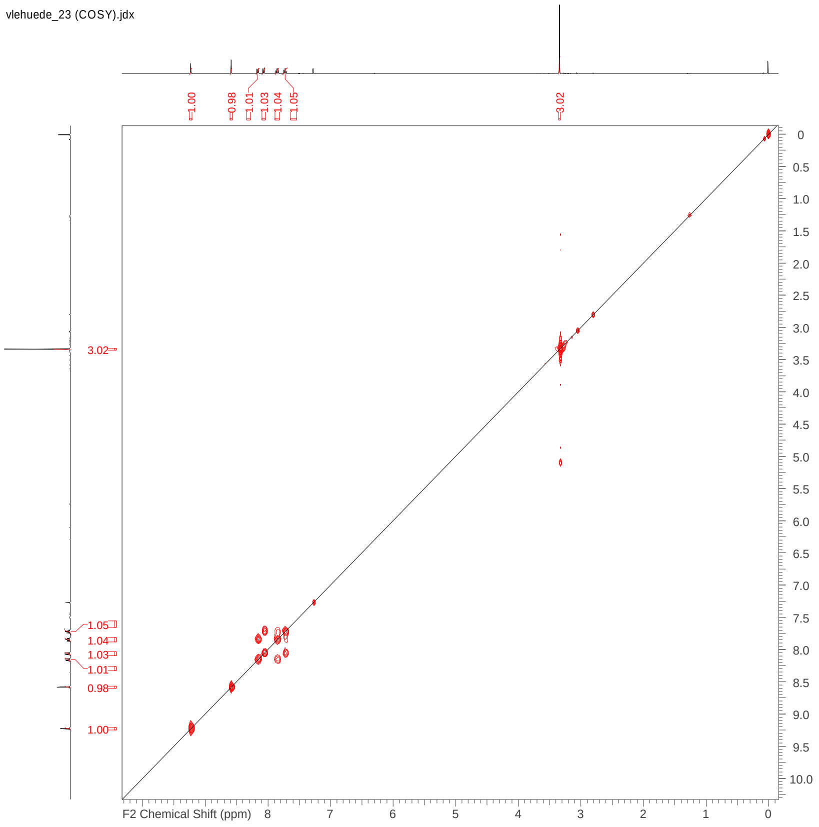
vlehuede_23 (COSY).jdx
F2 Chemical Shift (ppm)

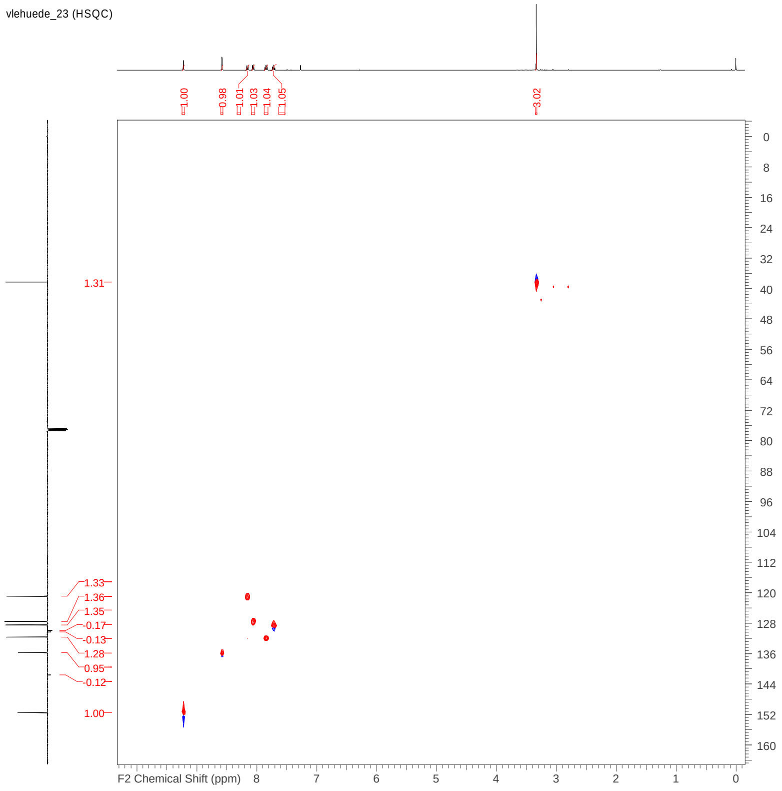
vlehuede_23 (HSQC)
F2 Chemical Shift (ppm)

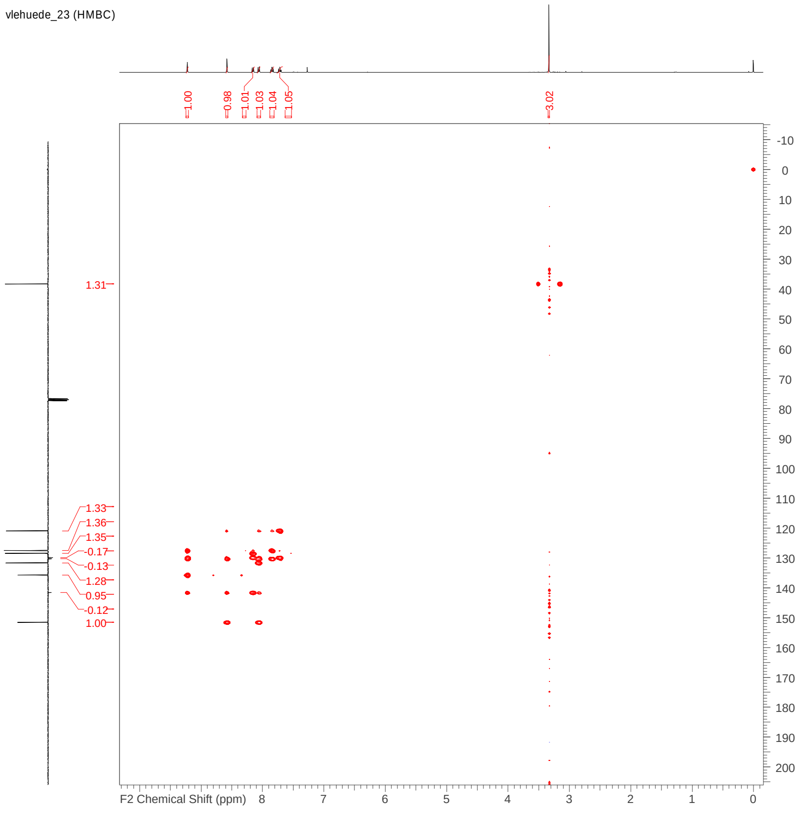

vlehuede_23 (HMBC)
1.00
0.98
1.01
1.03
1.04
1.05
3.02
1.31
1.33
1.36
1.35
-0.17
-0.13
1.28
0.95
-0.12
1.00
F2 Chemical Shift (ppm)

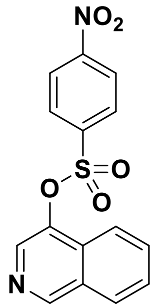

3w

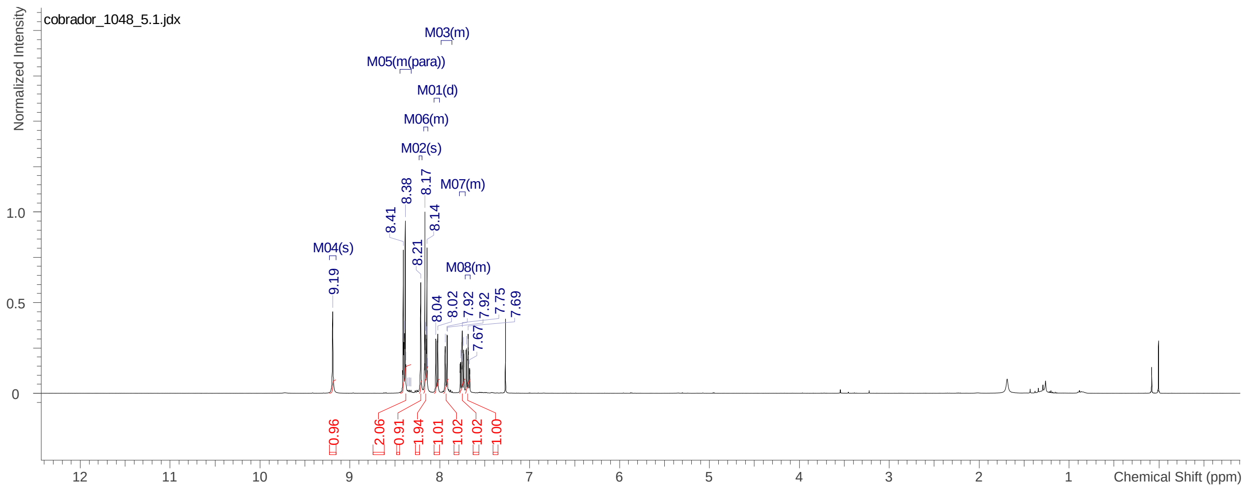
cobrador_1048_5.1.jdx
Normalized Intensity
Chemical Shift (ppm)
M04(s)
M05(m(para))
M03(m)
M01(d)
M06(m)
M02(s)
M07(m)
M08(m)
9.19
8.41
8.38
8.21
8.17
8.14
8.04
8.02
7.92
7.92
7.75
7.69
7.67
0.96
2.06
0.91
1.94
1.01
1.02
1.02
1.00

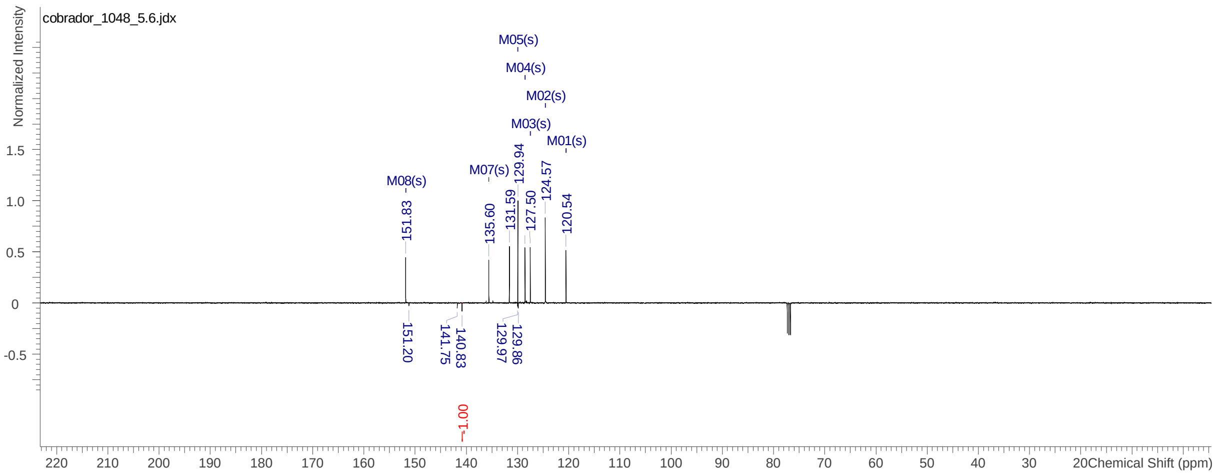
cobrador_1048_5.6.jdx
Normalized Intensity
Chemical Shift (ppm)
M05(s)
M04(s)
M02(s)
M03(s)
M01(s)
M07(s)
M08(s)
151.83
135.60
131.59
129.94
127.50
124.57
120.54
151.20
141.75
140.83
129.97
129.86
-1.00

cobrador_1048_5.2.jdx

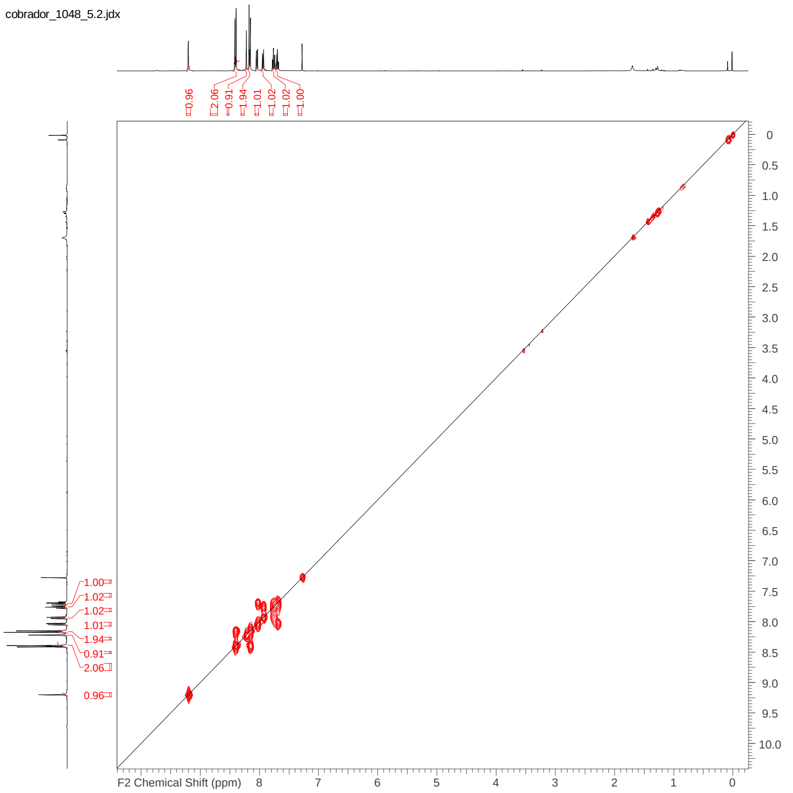

cobrador_1048_5.4.jdx

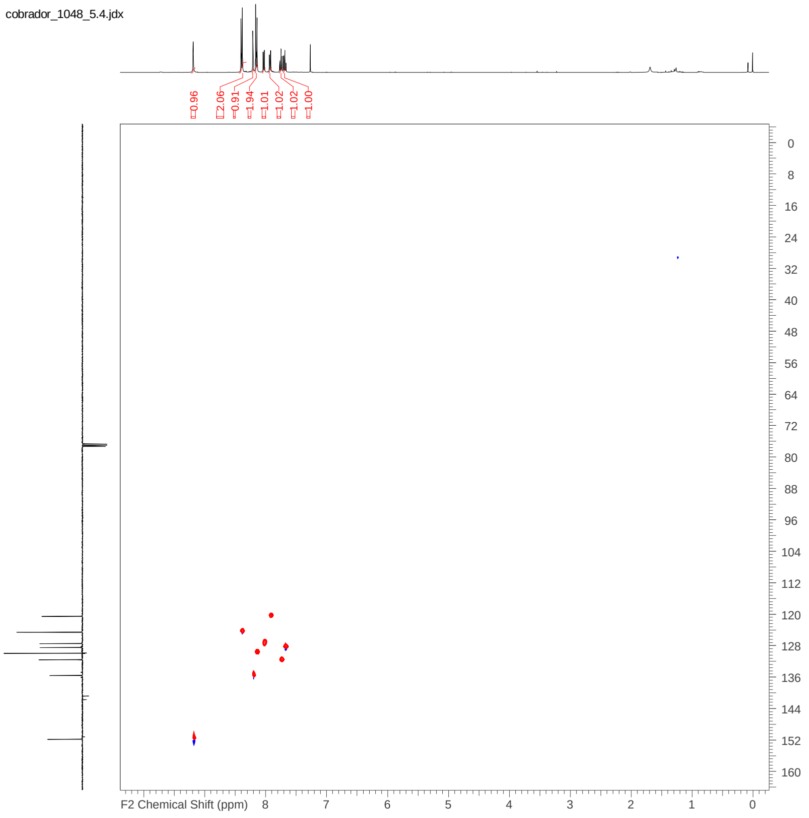

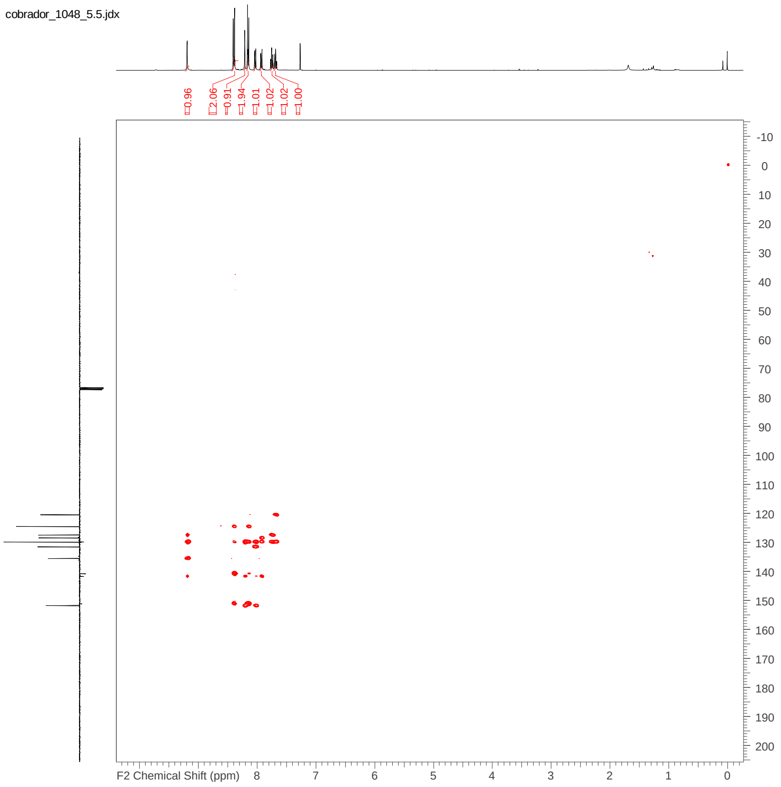
cobrador_1048_5.5.jdx
0.96
2.06
0.91
1.94
1.01
1.02
1.02
1.00
F2 Chemical Shift (ppm)

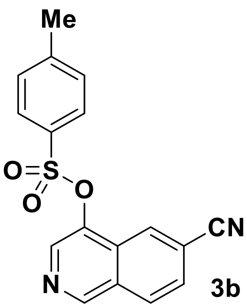

3b

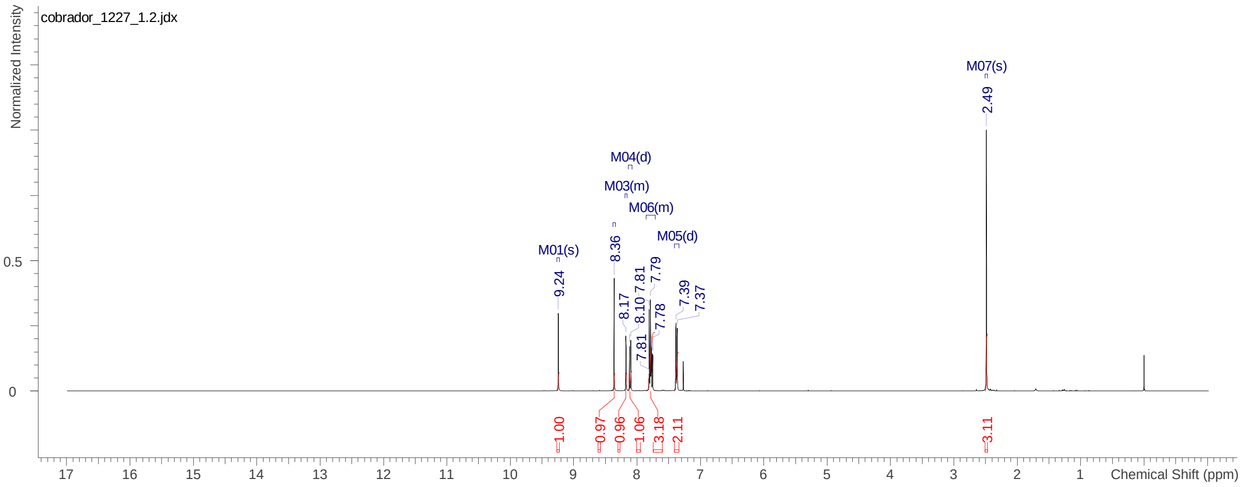

cobrador_1227_1.2.jdx
Normalized Intensity
Chemical Shift (ppm)
M01(s)
M03(m)
M04(d)
M05(d)
M06(m)
M07(s)
9.24
8.36
8.17
8.10
7.81
7.81
7.79
7.78
7.39
7.37
2.49
1.00
0.97
0.96
1.06
3.18
2.11
3.11

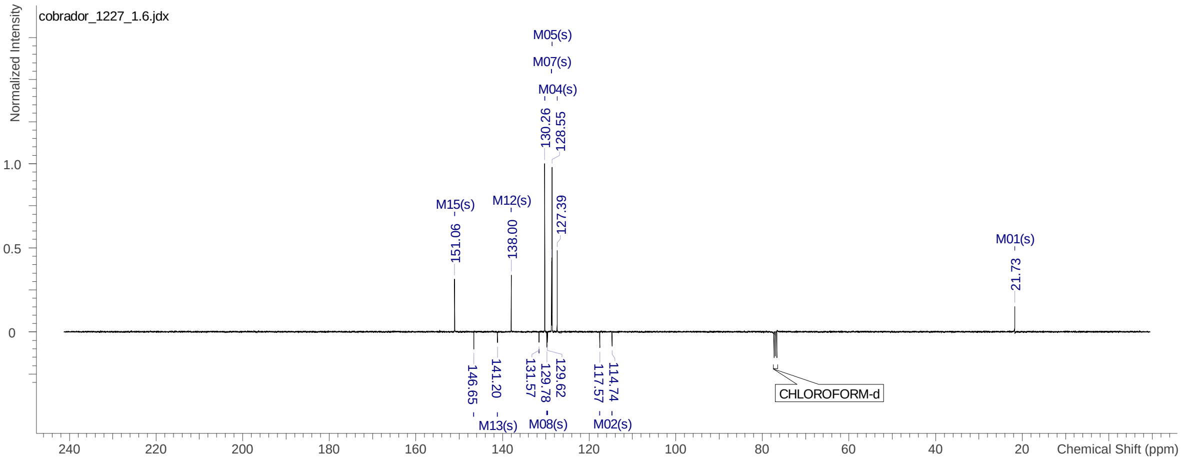

cobrador_1227_1.6.jdx
Normalized Intensity
Chemical Shift (ppm)
M15(s)
M12(s)
M05(s)
M07(s)
M04(s)
M01(s)
151.06
138.00
130.26
128.55
127.39
21.73
146.65
141.20
131.57
129.78
129.62
117.57
114.74
M13(s)
M08(s)
M02(s)
CHLOROFORM-d

cobrador_1227_1.3.jdx

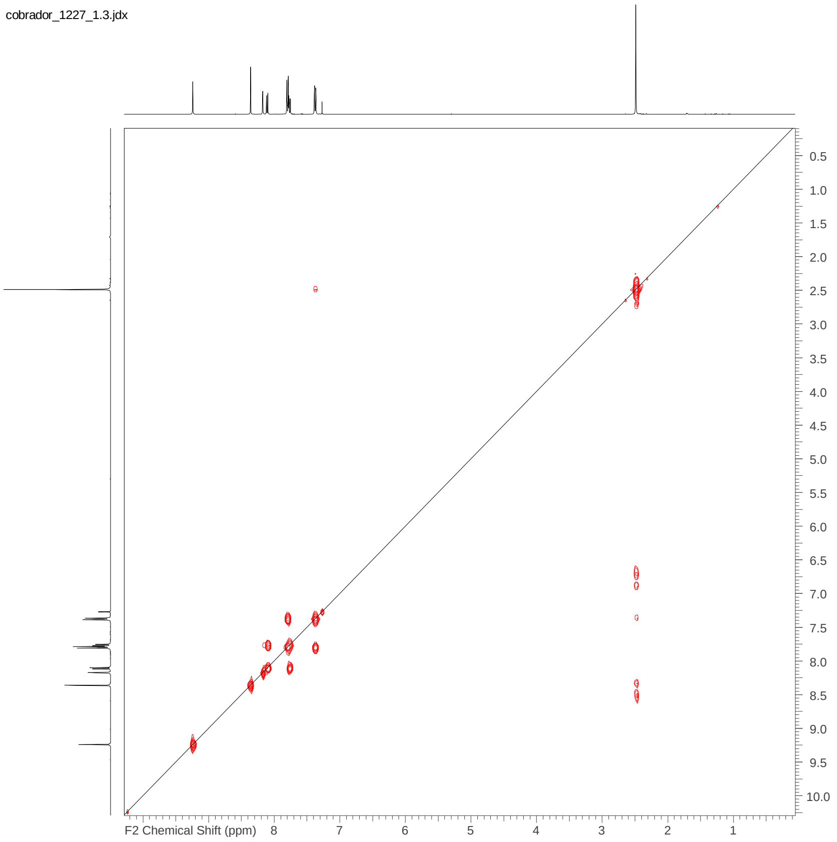

cobrador_1227_1.4.jdx

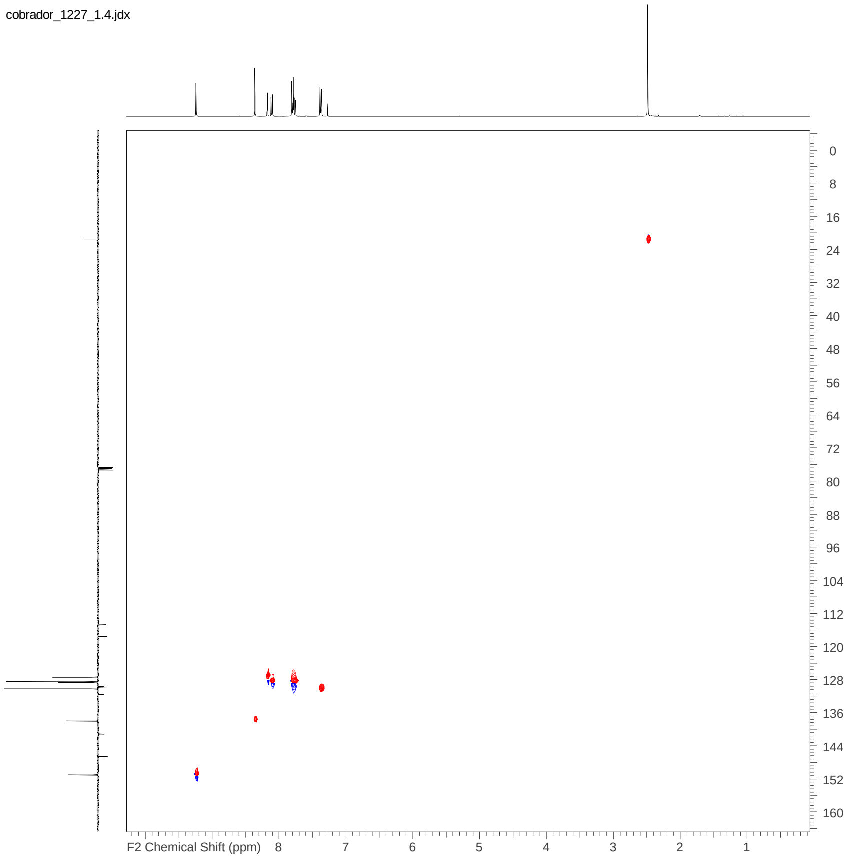

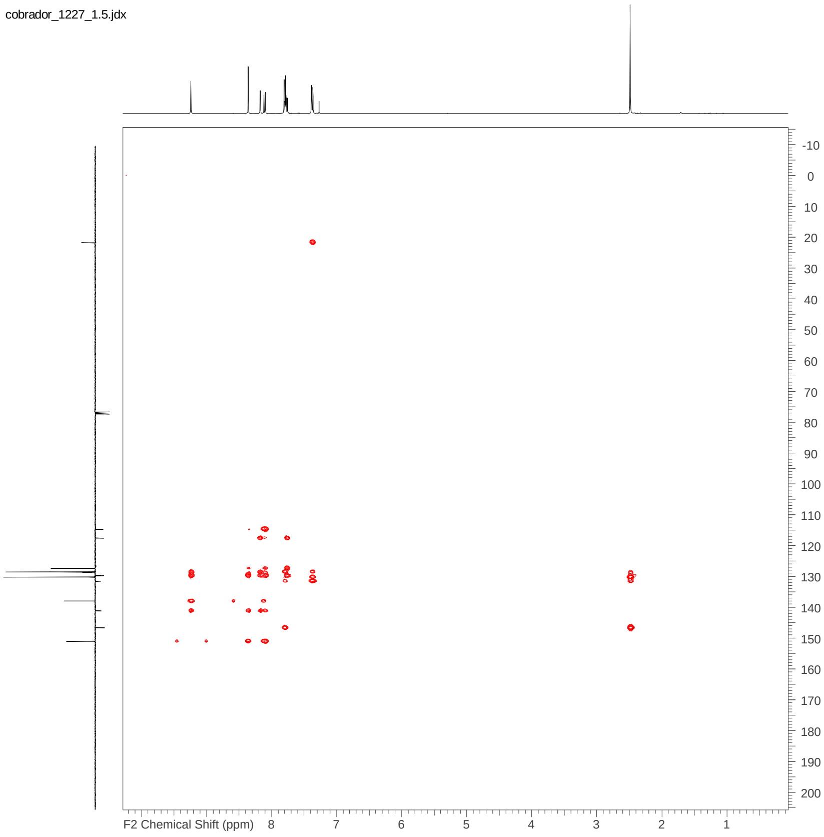
cobrador_1227_1.5.jdx
F2 Chemical Shift (ppm)
8
7
6
5
4
3
2
1
-10
0
10
20
30
40
50
60
70
80
90
100
110
120
130
140
150
160
170
180
190
200

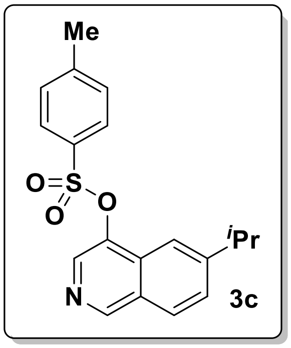
Me
O
S
O
O
N
iPr
3c

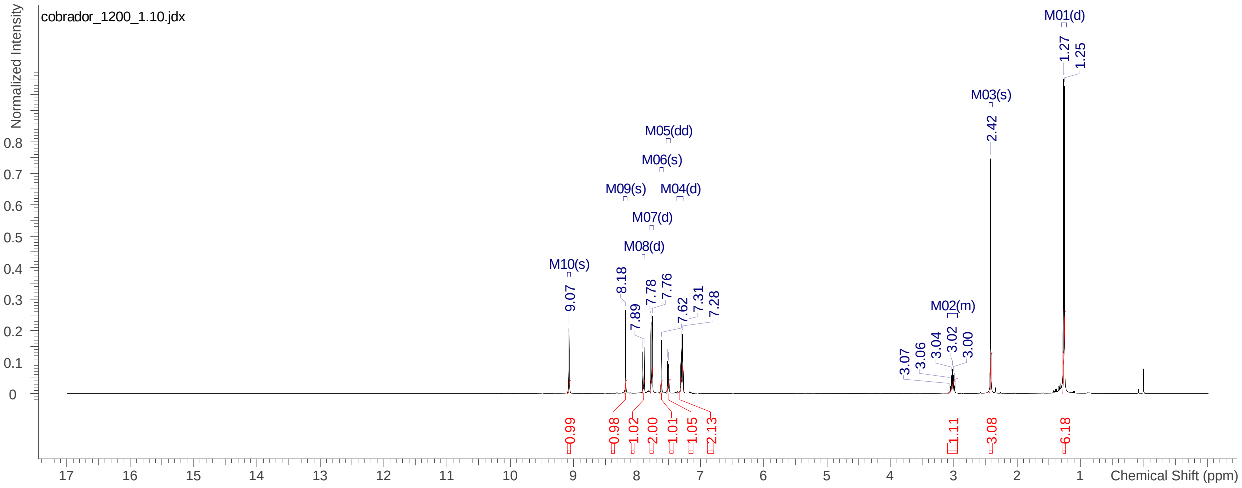
cobrador_1200_1.10.jdx
Normalized Intensity
M10(s)
9.07
M09(s)
8.18
M08(d)
7.89
M07(d)
7.78
M06(s)
7.76
M05(dd)
M04(d)
7.62
7.31
7.28
M02(m)
3.07
3.06
3.04
3.02
3.00
M03(s)
2.42
M01(d)
1.27
1.25
0.99
0.98
1.02
2.00
1.01
1.05
2.13
1.11
3.08
6.18
Chemical Shift (ppm)

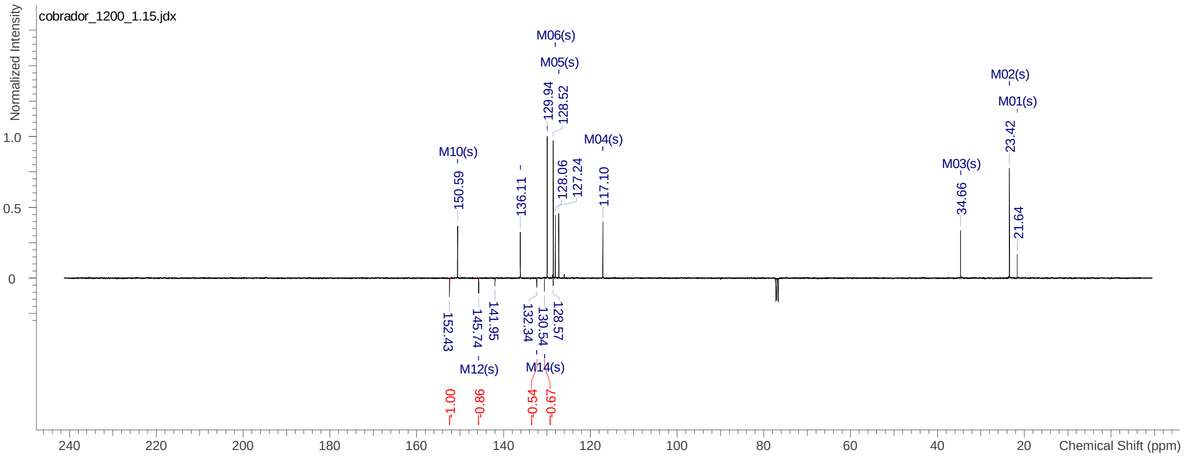
cobrador_1200_1.15.jdx
Normalized Intensity
M10(s)
150.59
136.11
M06(s)
129.94
M05(s)
128.52
128.06
127.24
M04(s)
117.10
M03(s)
34.66
M02(s)
M01(s)
23.42
21.64
152.43
145.74
141.95
132.34
130.54
128.57
M12(s)
M14(s)
1.00
0.86
0.54
0.67
Chemical Shift (ppm)

cobrador_1200_1.11.jdx

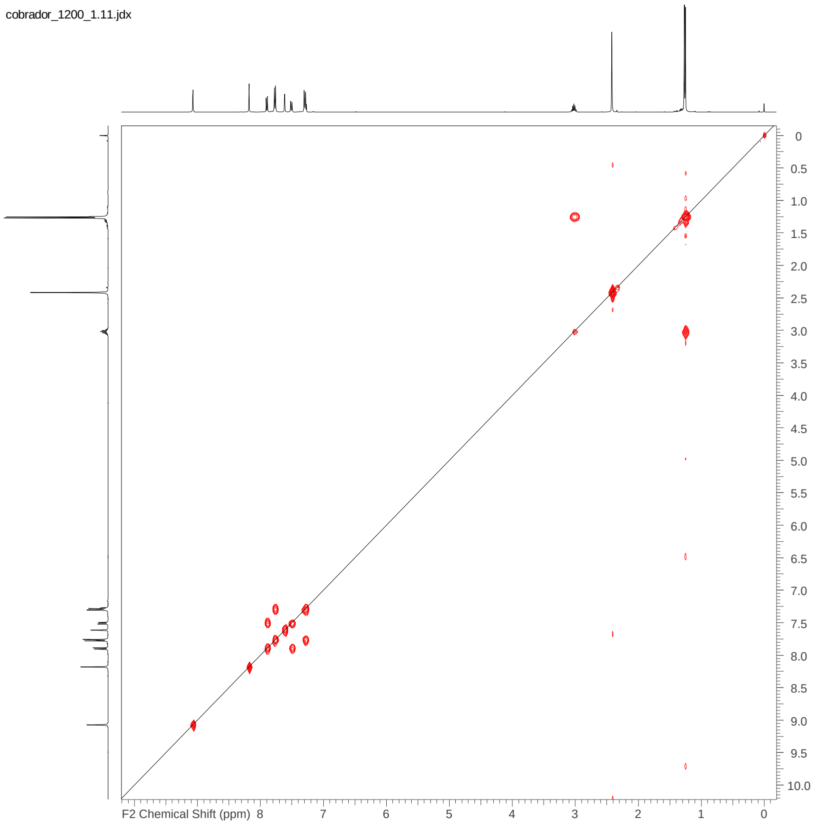

cobrador_1200_1.12.jdx

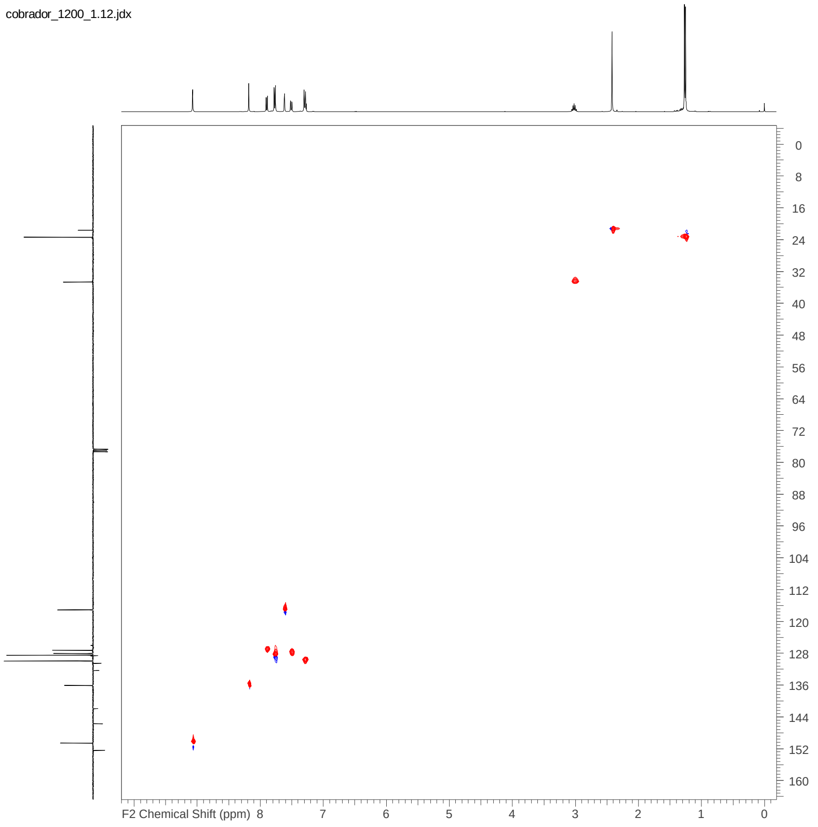

cobrador_1200_1.14.jdx

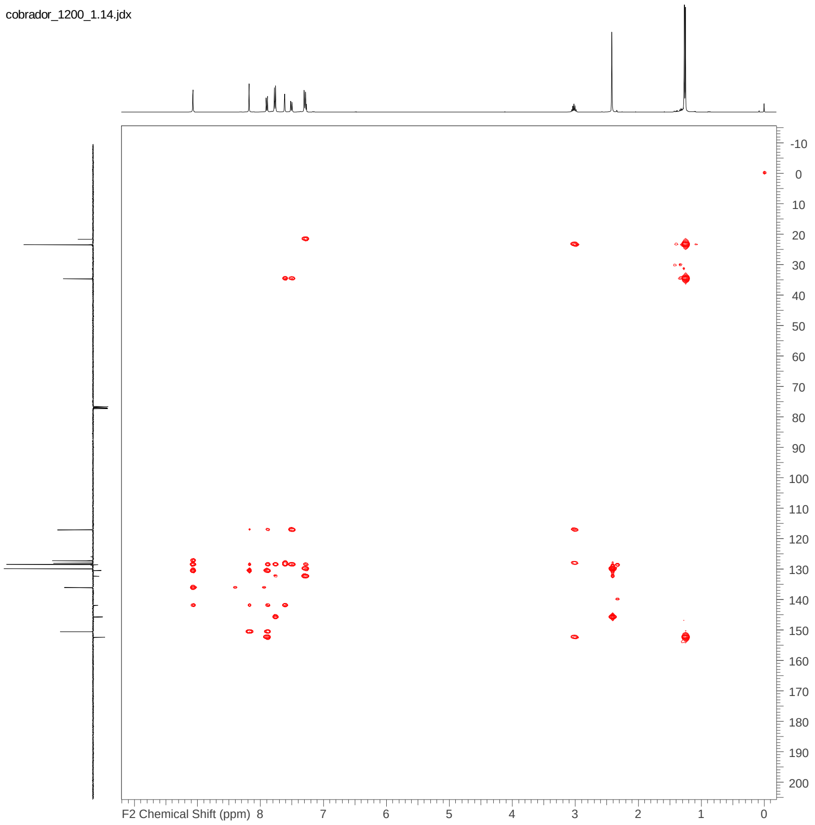

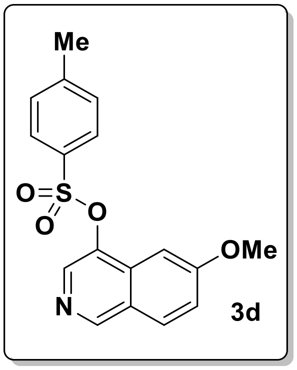
Me
O=S
O
O
OMe
N
3d

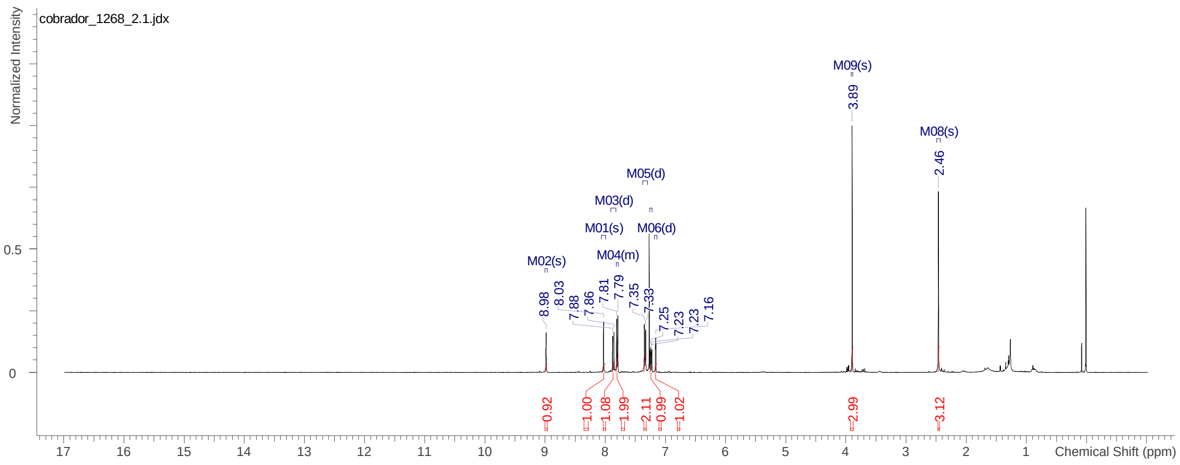
cobrador_1268_2.1.jdx
Normalized Intensity
M09(s)
3.89
M08(s)
2.46
M05(d)
M03(d)
M01(s)
M06(d)
M04(m)
M02(s)
8.98
8.03
7.88
7.86
7.81
7.79
7.35
7.33
7.25
7.23
7.23
7.16
0.92
1.00
1.08
1.99
2.11
0.99
1.02
2.99
3.12
0.5
0
17 16 15 14 13 12 11 10 9 8 7 6 5 4 3 2 1
Chemical Shift (ppm)

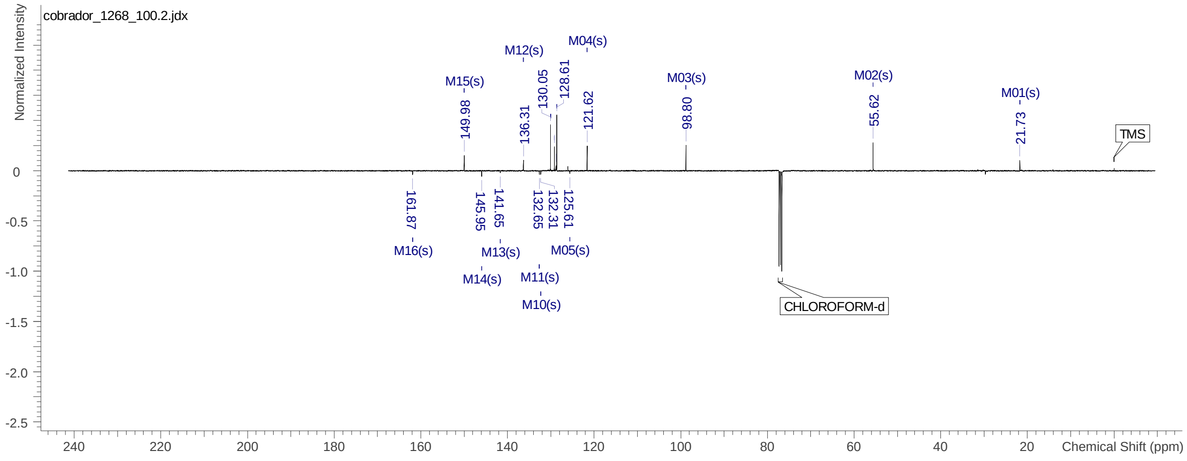
cobrador_1268_100.2.jdx
Normalized Intensity
M12(s)
M04(s)
M15(s)
130.05
128.61
149.98
136.31
121.62
M03(s)
98.80
M02(s)
55.62
M01(s)
21.73
TMS
161.87
145.95
141.65
132.65
132.31
125.61
M16(s)
M13(s)
M05(s)
M14(s)
M11(s)
M10(s)
CHLOROFORM-d
0
-0.5
-1.0
-1.5
-2.0
-2.5
240 220 200 180 160 140 120 100 80 60 40 20
Chemical Shift (ppm)

cobrador_1268_2.2.jdx

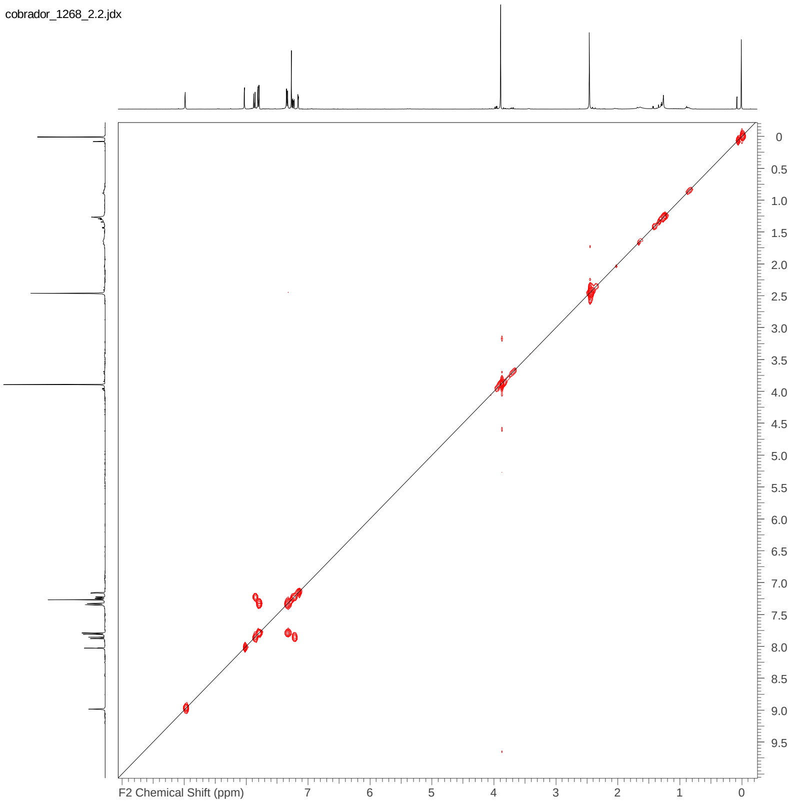

cobrador_1268_2.3.jdx

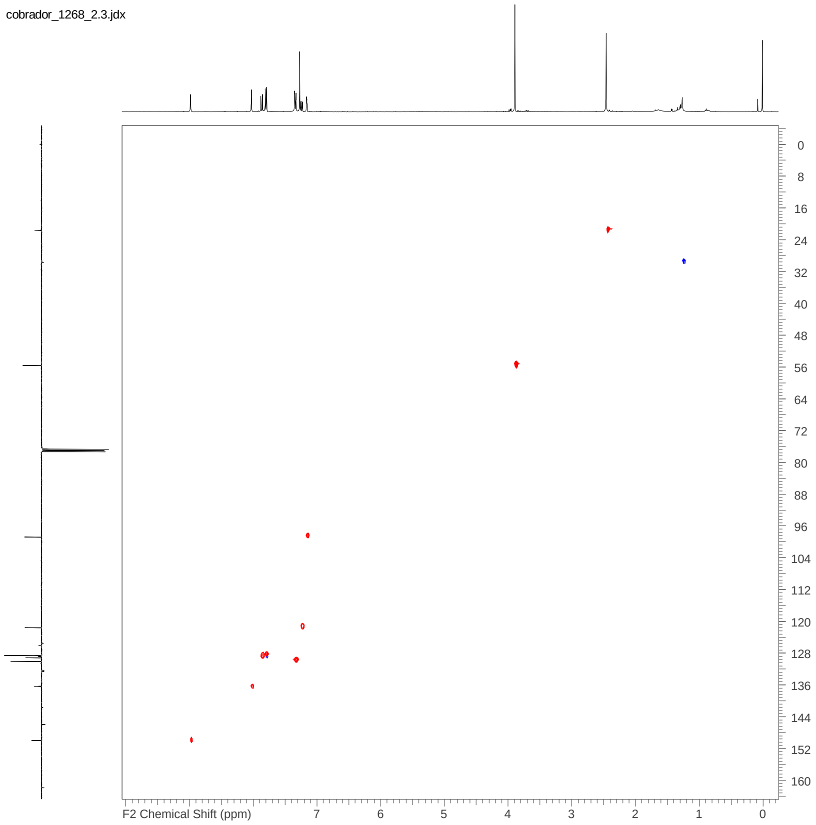

cobrador_1268_2.4.jdx

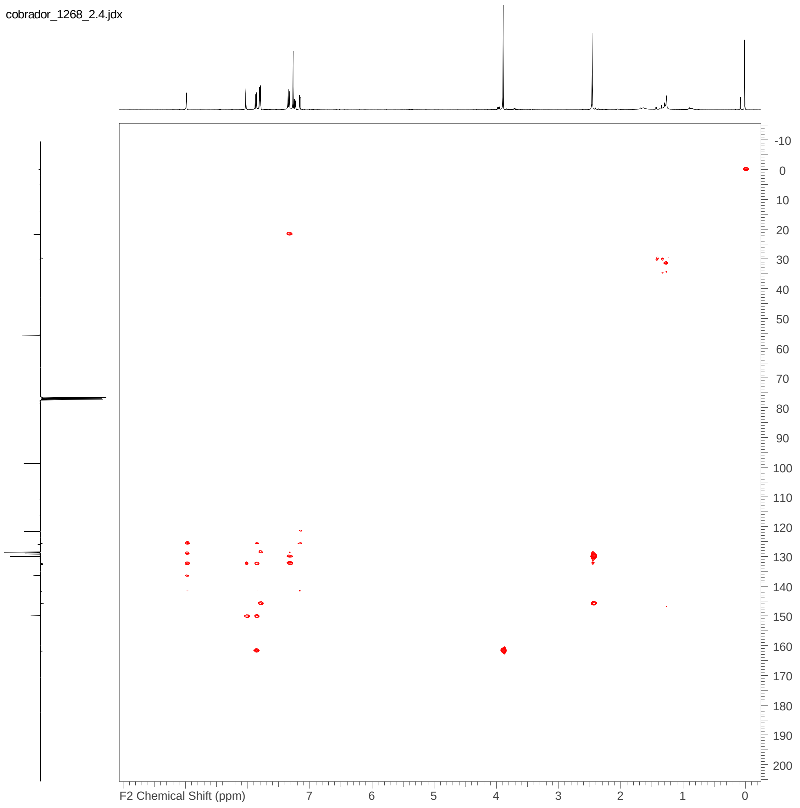

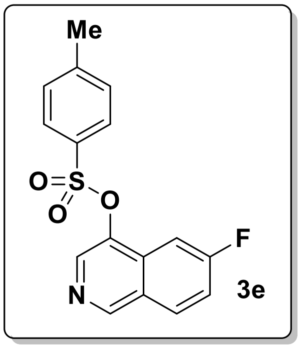
Me
O
S
O
O
F
N
3e

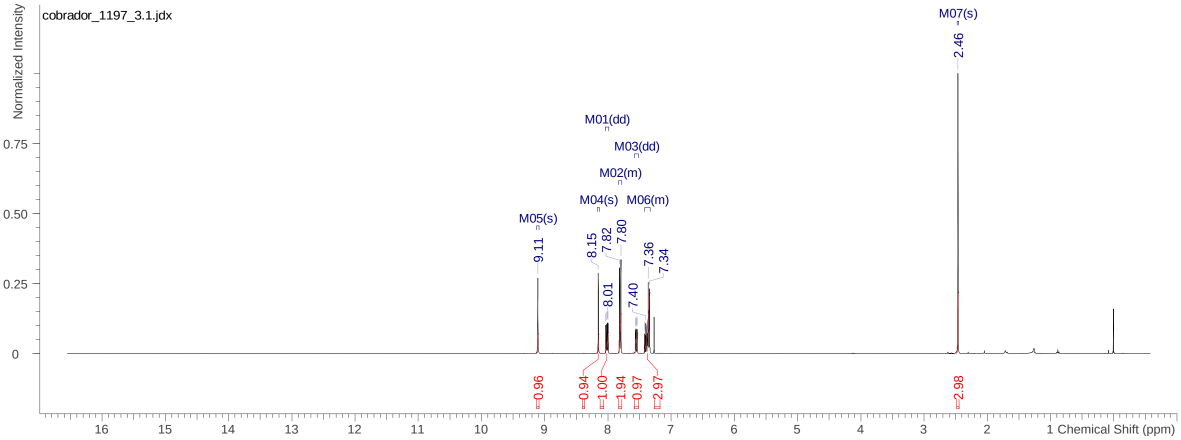
cobrador_1197_3.1.jdx
Normalized Intensity
M07(s)
2.46
M01(dd)
M03(dd)
M02(m)
M04(s)
M06(m)
M05(s)
9.11
8.15
7.82
7.80
8.01
7.40
7.36
7.34
0.96
0.94
1.00
1.94
0.97
2.97
2.98
0.75
0.50
0.25
0
16 15 14 13 12 11 10 9 8 7 6 5 4 3 2 1 Chemical Shift (ppm)

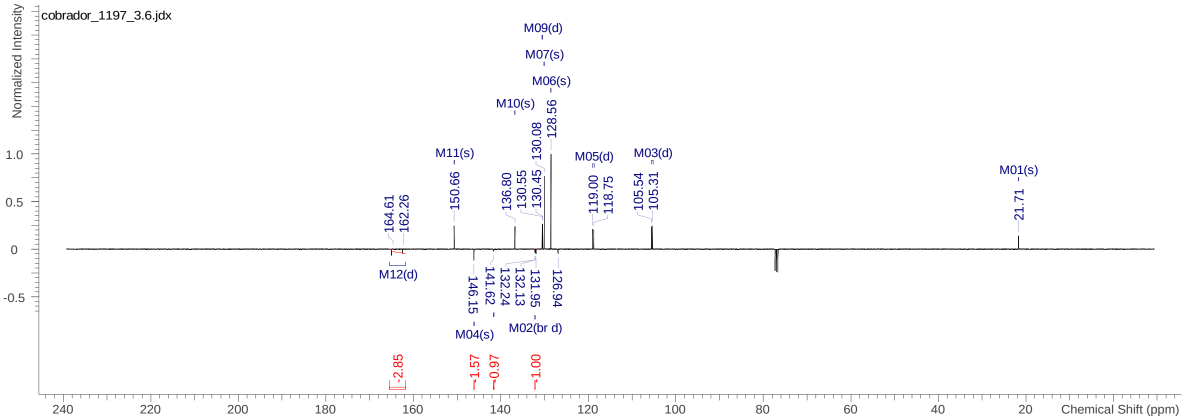
cobrador_1197_3.6.jdx
Normalized Intensity
M09(d)
M07(s)
M06(s)
M10(s)
M11(s)
M05(d)
M03(d)
M01(s)
128.56
130.08
150.66
136.80
130.55
130.45
119.00
118.75
105.54
105.31
21.71
164.61
162.26
M12(d)
146.15
141.62
132.24
132.13
131.95
126.94
M04(s)
M02(br d)
-2.85
-1.57
-0.97
-1.00
1.0
0.5
0
-0.5
240 220 200 180 160 140 120 100 80 60 40 20 Chemical Shift (ppm)

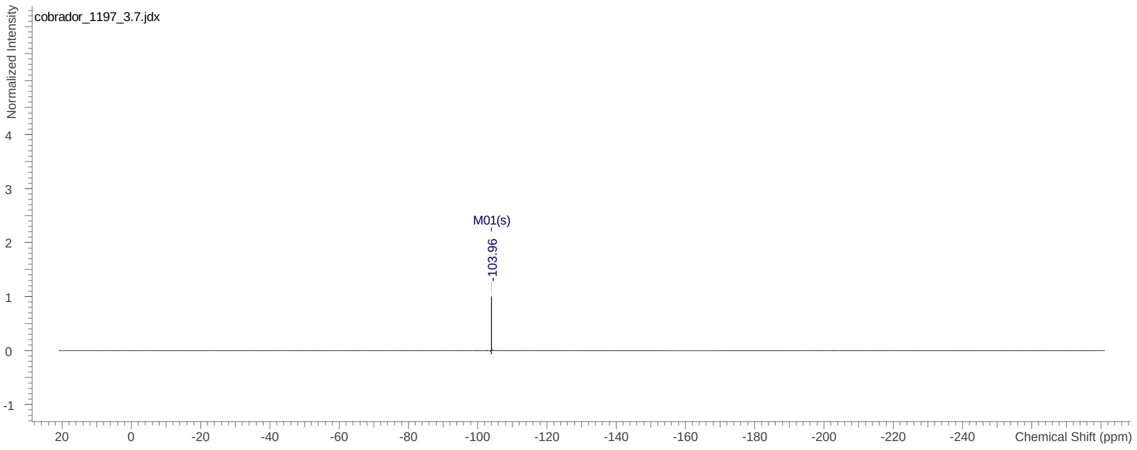
cobrador_1197_3.7.jdx
Normalized Intensity
M01(s)
-103.96
Chemical Shift (ppm)

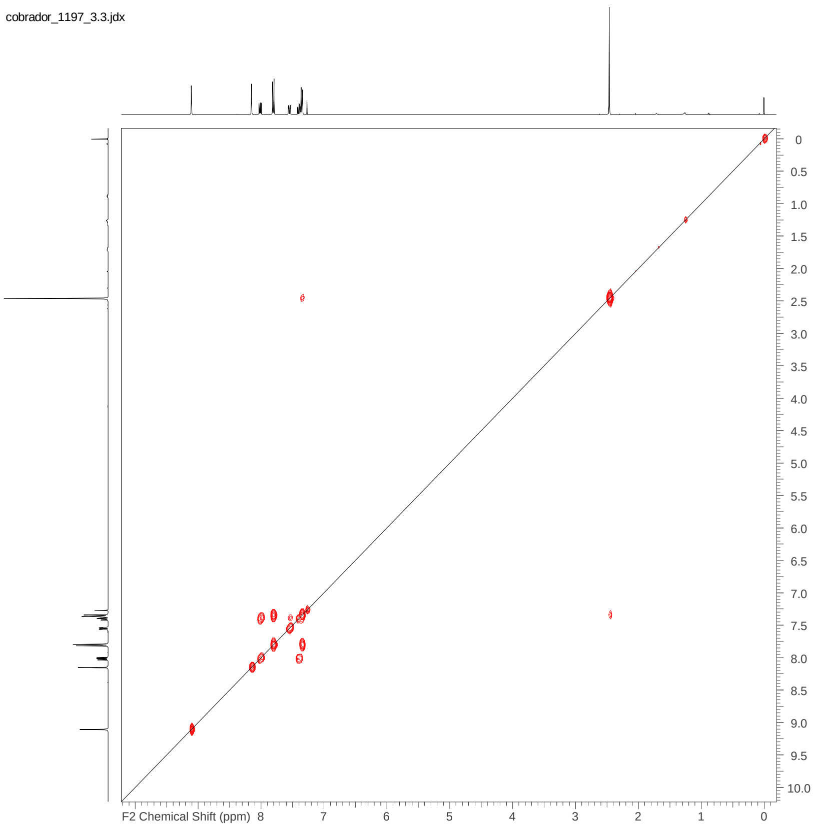
cobrador_1197_3.3.jdx
F2 Chemical Shift (ppm)

cobrador_1197_3.4.jdx

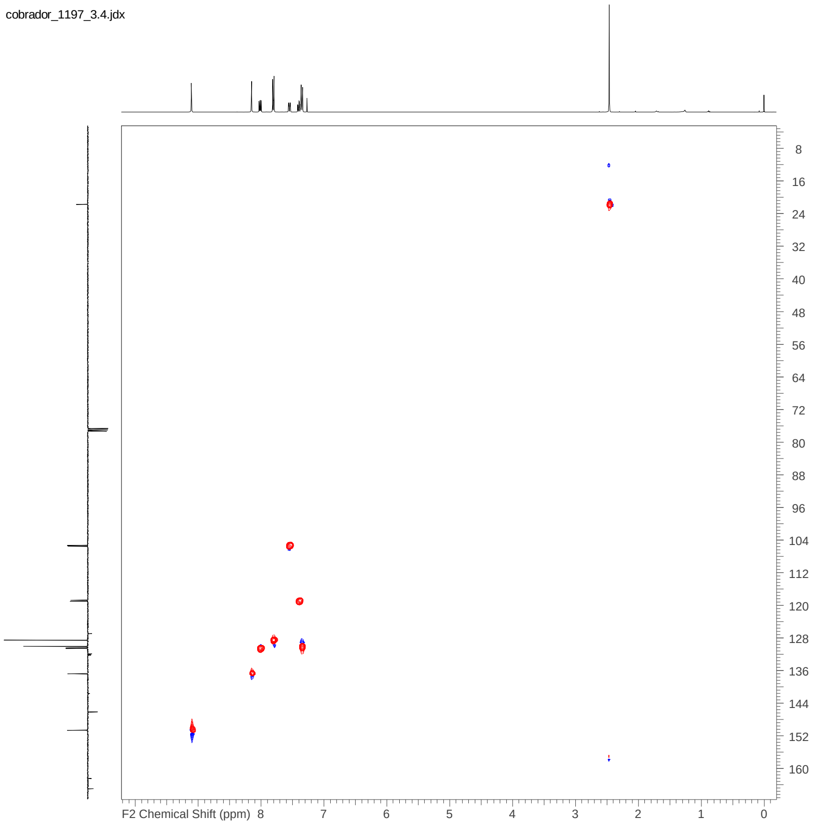

cobrador_1197_3.11.jdx

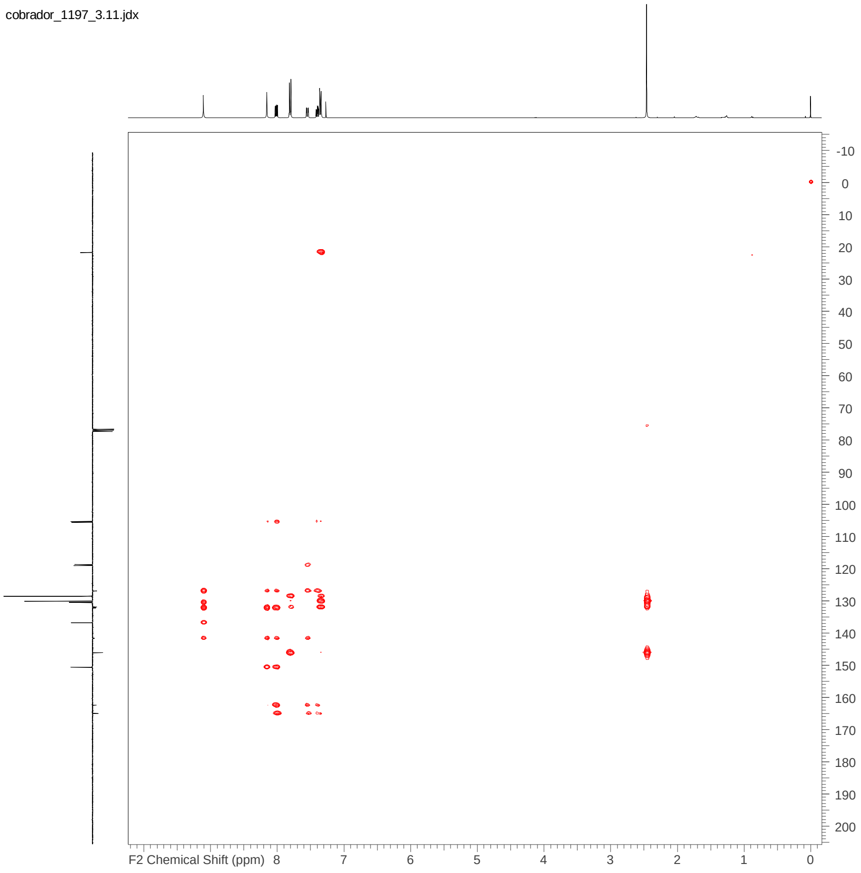

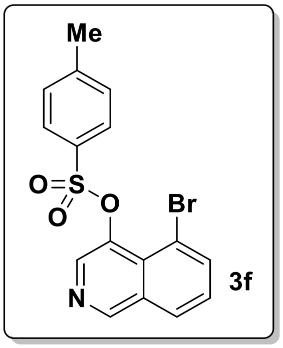

Me
O
S
O
O
Br
N

3f

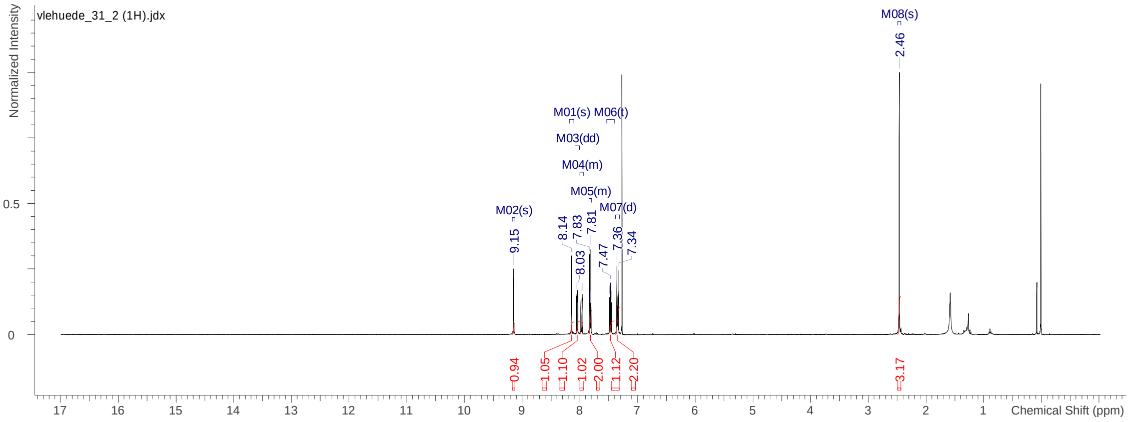

vlehuede_31_2 (1H).jdx
Normalized Intensity
Chemical Shift (ppm)
M01(s)
M02(s)
M03(dd)
M04(m)
M05(m)
M06(t)
M07(d)
M08(s)
9.15
8.14
8.03
7.83
7.81
7.47
7.36
7.34
2.46
0.94
1.05
1.10
1.02
2.00
1.12
2.20
3.17

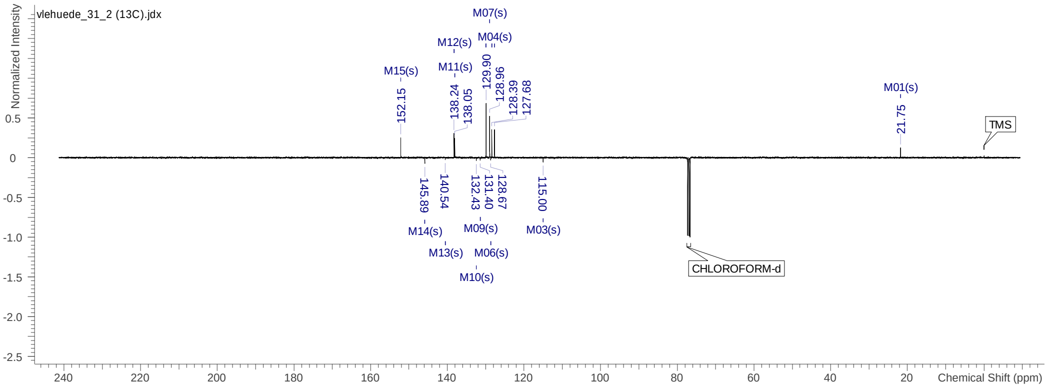

vlehuede_31_2 (13C).jdx
Normalized Intensity
Chemical Shift (ppm)
M15(s)
M12(s)
M11(s)
M07(s)
M04(s)
M01(s)
152.15
138.24
138.05
129.90
128.96
128.39
127.68
21.75
TMS
145.89
140.54
132.43
131.40
128.67
115.00
M14(s)
M13(s)
M09(s)
M06(s)
M10(s)
M03(s)
CHLOROFORM-d

cobrador_vleuhende31.2.jdx

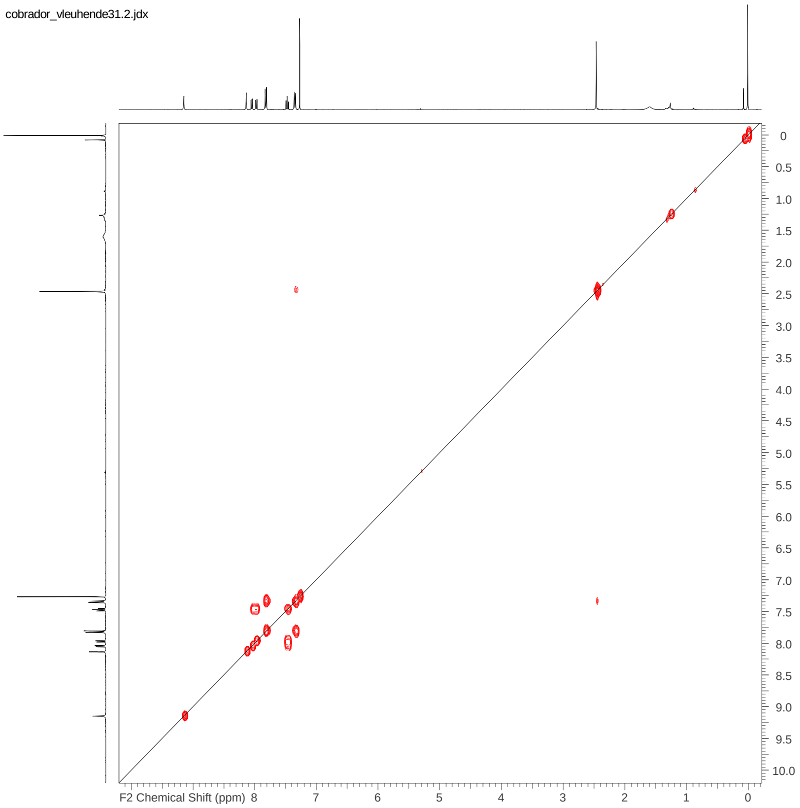

vlehuede_31_2 (HSQC).jdx

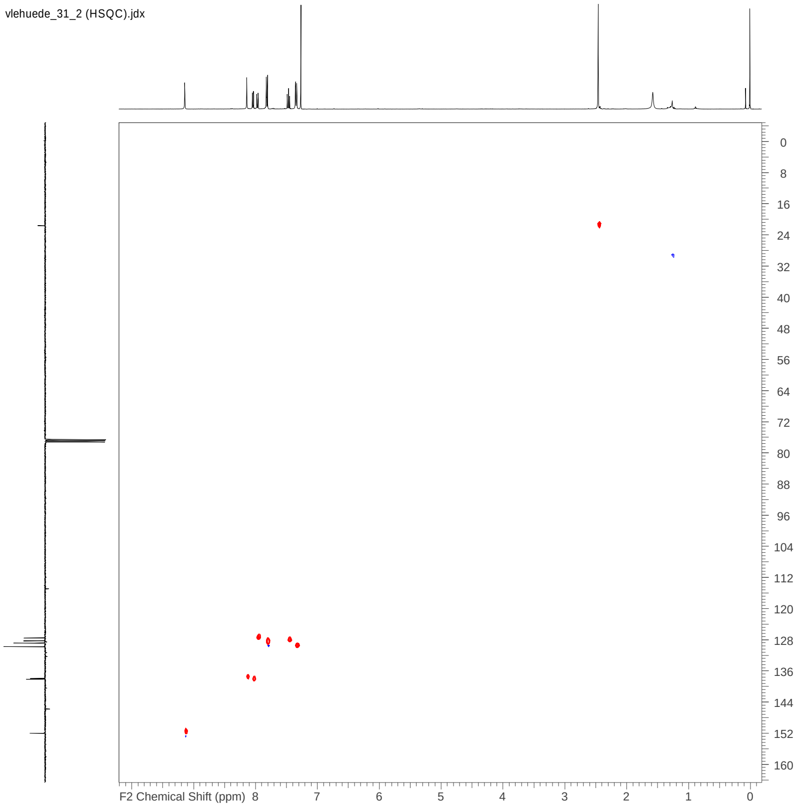

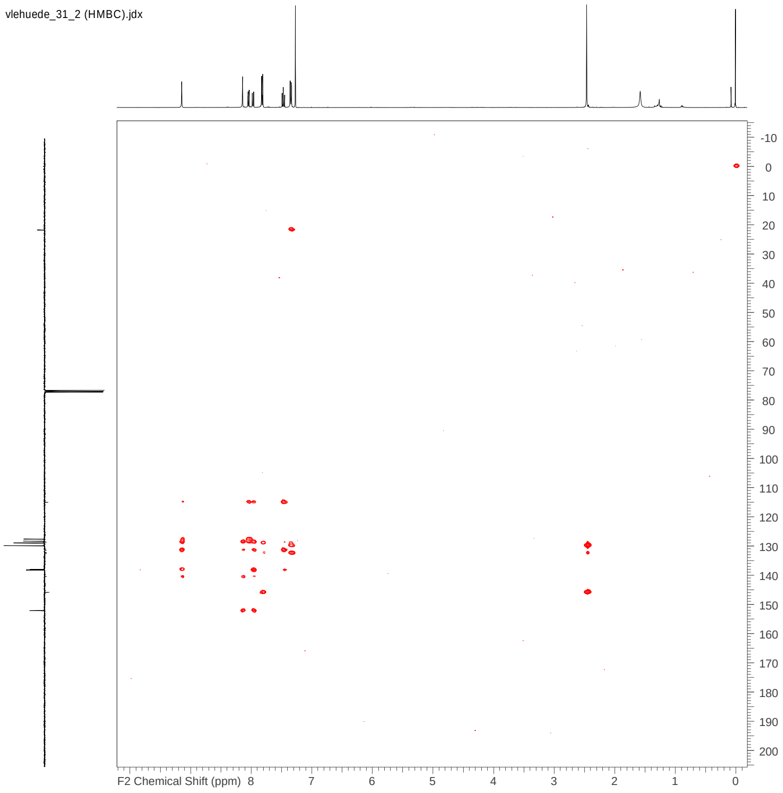
vlehuede_31_2 (HMBC).jdx
F2 Chemical Shift (ppm)

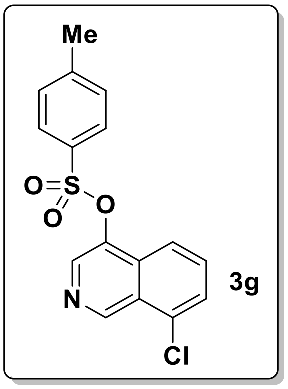
Me
O=S
O
O
N
Cl

3g

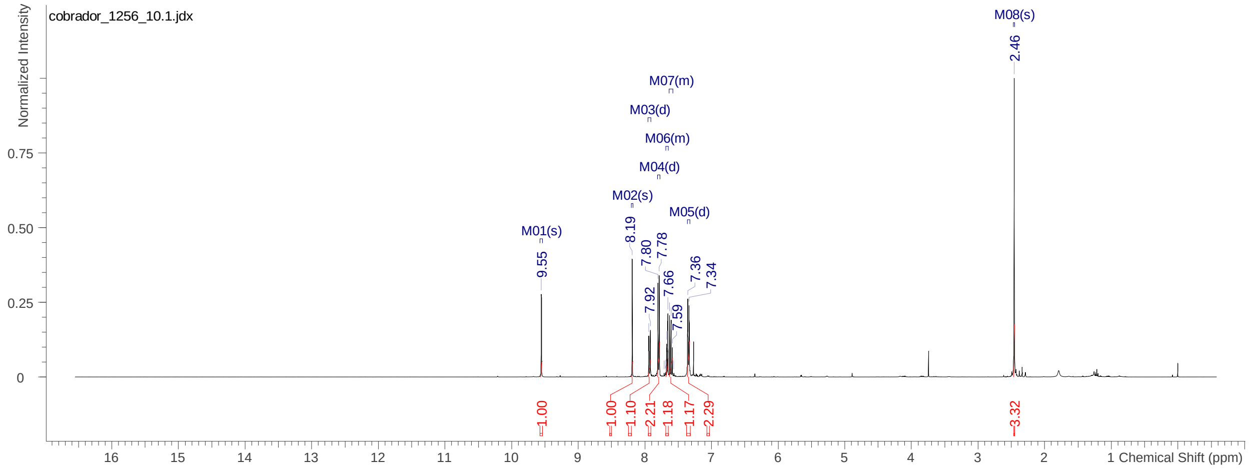
cobrador_1256_10.1.jdx
Normalized Intensity
Chemical Shift (ppm)
M01(s)
9.55
M02(s)
8.19
M03(d)
M04(d)
M05(d)
M06(m)
M07(m)
M08(s)
2.46
7.92
7.80
7.78
7.66
7.59
7.36
7.34
1.00
1.00
1.10
2.21
1.18
1.17
2.29
3.32

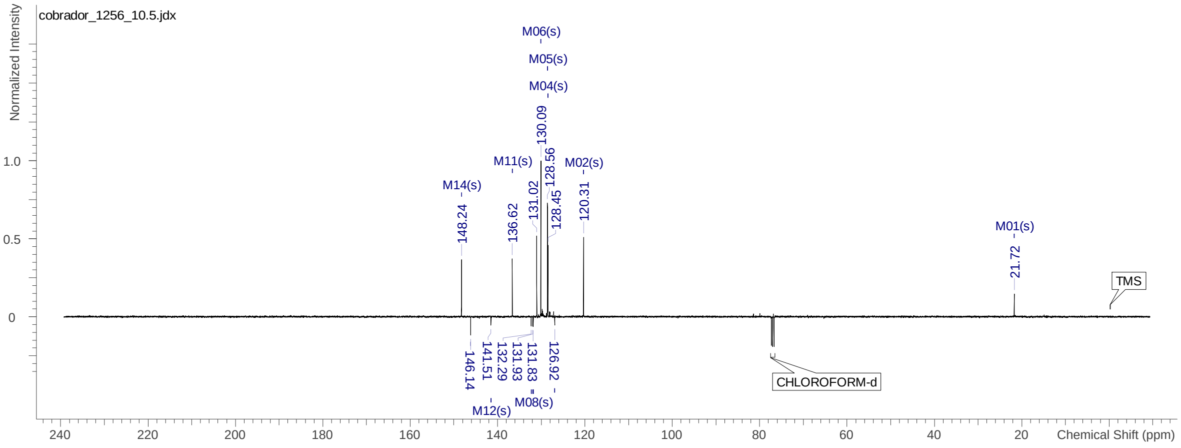
cobrador_1256_10.5.jdx
Normalized Intensity
Chemical Shift (ppm)
M14(s)
148.24
M11(s)
136.62
M06(s)
M05(s)
M04(s)
130.09
131.02
128.56
128.45
M02(s)
120.31
M01(s)
21.72
TMS
146.14
141.51
132.29
131.93
131.83
126.92
M12(s)
M08(s)
CHLOROFORM-d

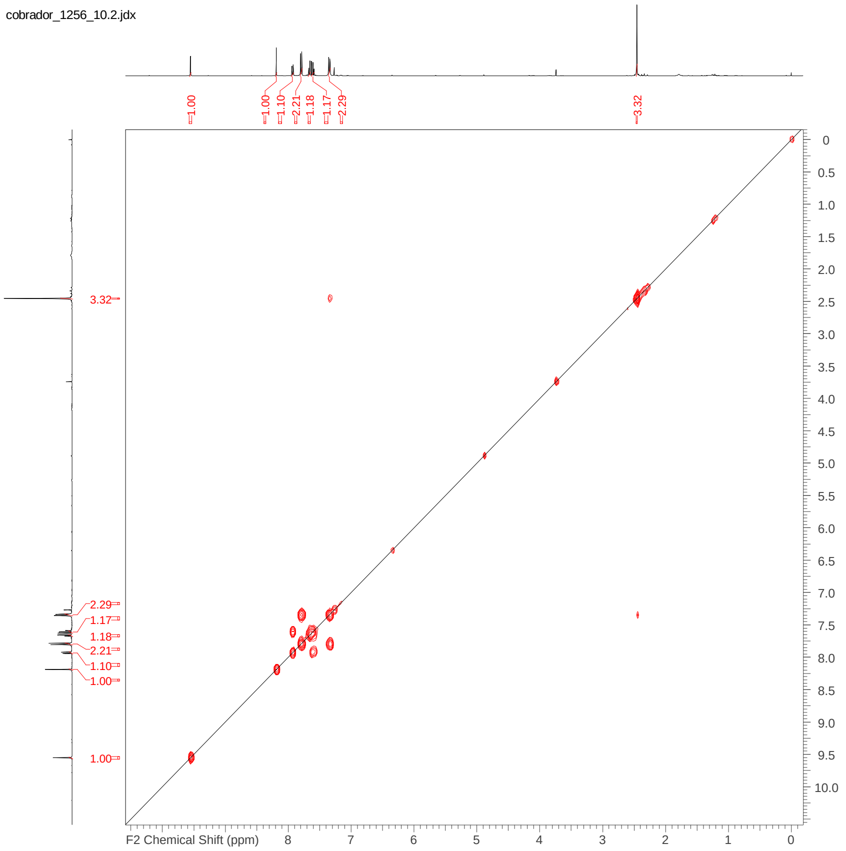
cobrador_1256_10.2.jdx
F2 Chemical Shift (ppm)

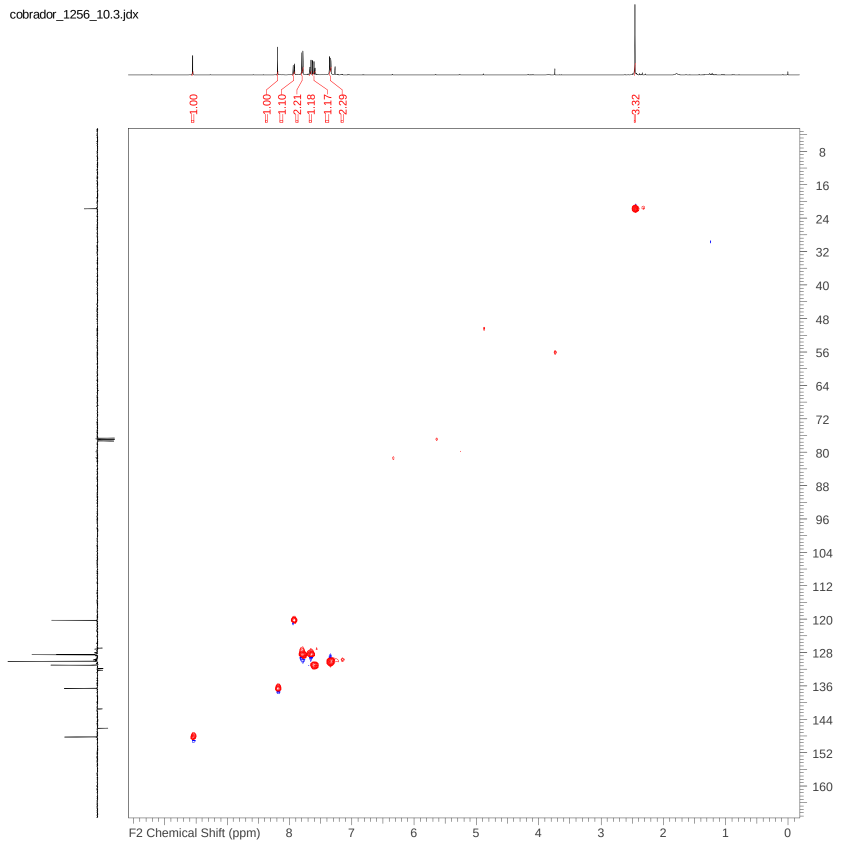
cobrador_1256_10.3.jdx
F2 Chemical Shift (ppm)

cobrador_1256_10.4.jdx

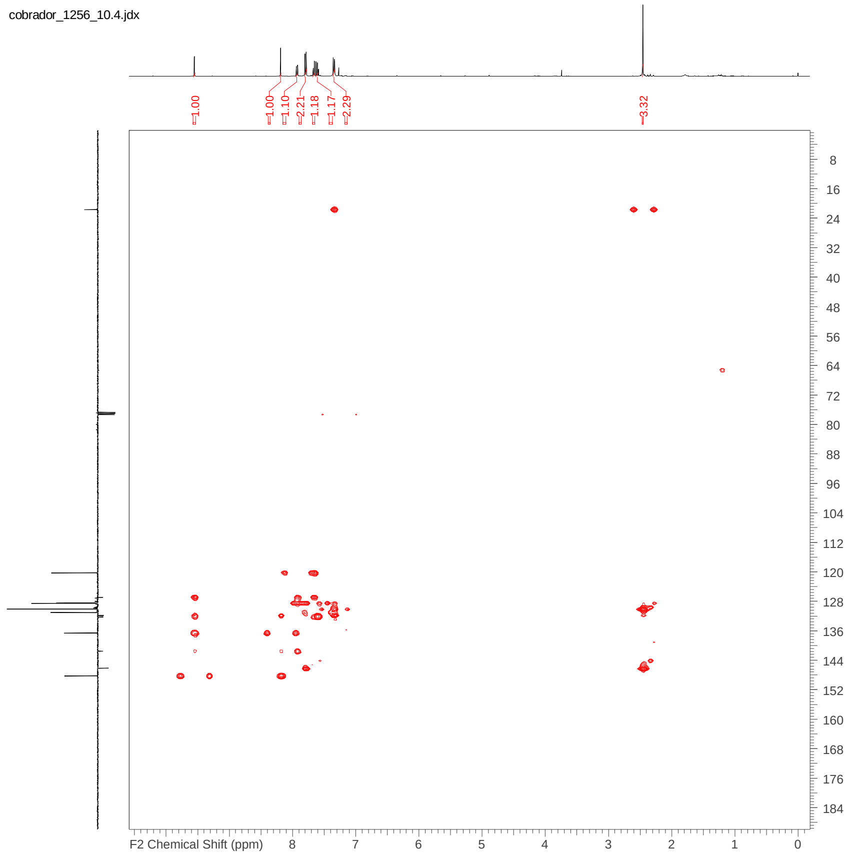

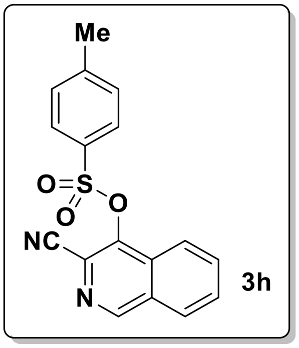
Me
O
S
O
O
NC
N

3h

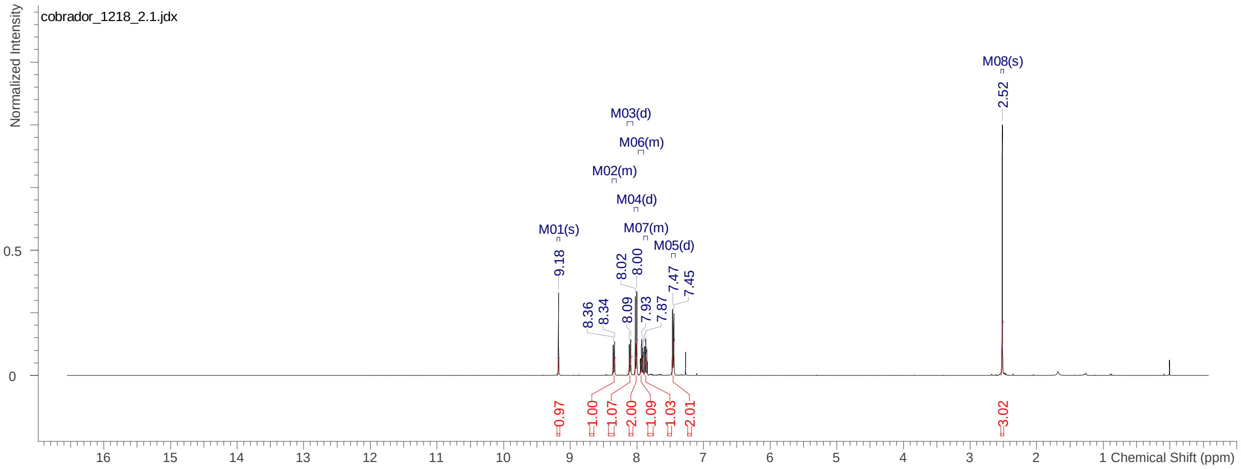
cobrador_1218_2.1.jdx
Normalized Intensity
M08(s)
2.52
M03(d)
M06(m)
M02(m)
M04(d)
M01(s)
M07(m)
M05(d)
9.18
8.36
8.34
8.09
8.02
8.00
7.93
7.87
7.47
7.45
0.97
1.00
1.07
2.00
1.09
1.03
2.01
3.02
Chemical Shift (ppm)

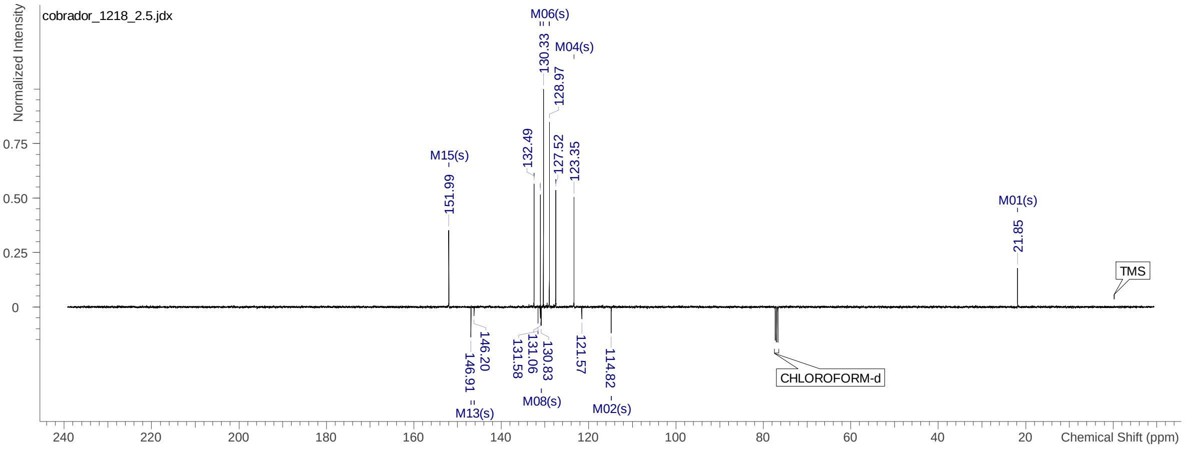
cobrador_1218_2.5.jdx
Normalized Intensity
M06(s)
130.33
M04(s)
128.97
132.49
127.52
123.35
M15(s)
151.99
M01(s)
21.85
TMS
146.20
146.91
131.58
131.06
130.83
121.57
114.82
M13(s)
M08(s)
M02(s)
CHLOROFORM-d
Chemical Shift (ppm)

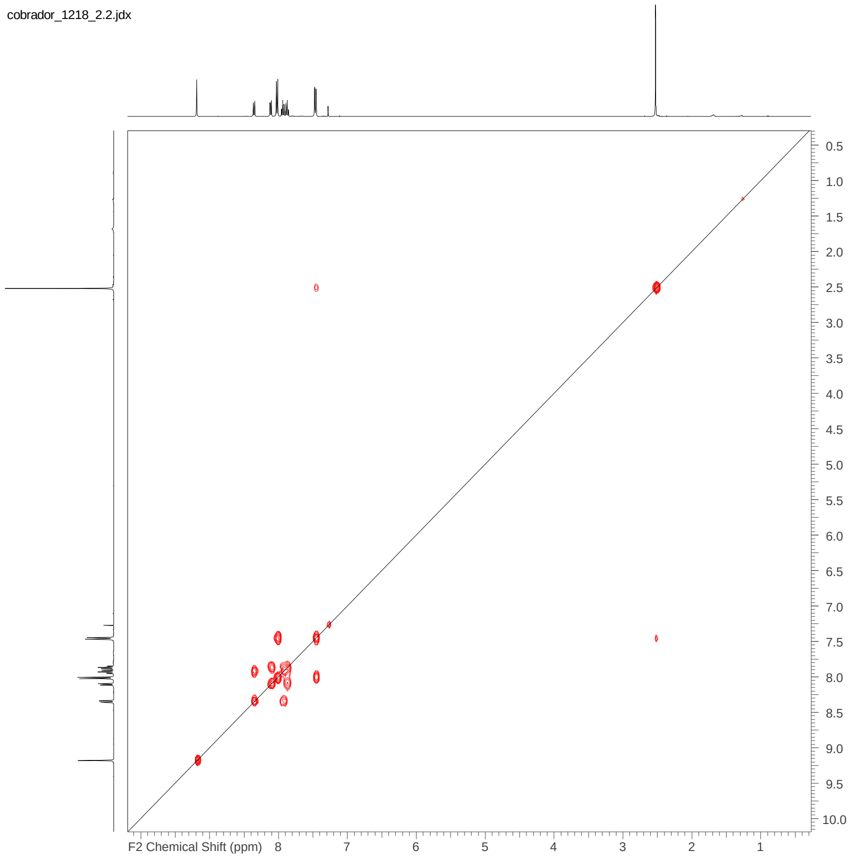
cobrador_1218_2.2.jdx
F2 Chemical Shift (ppm)

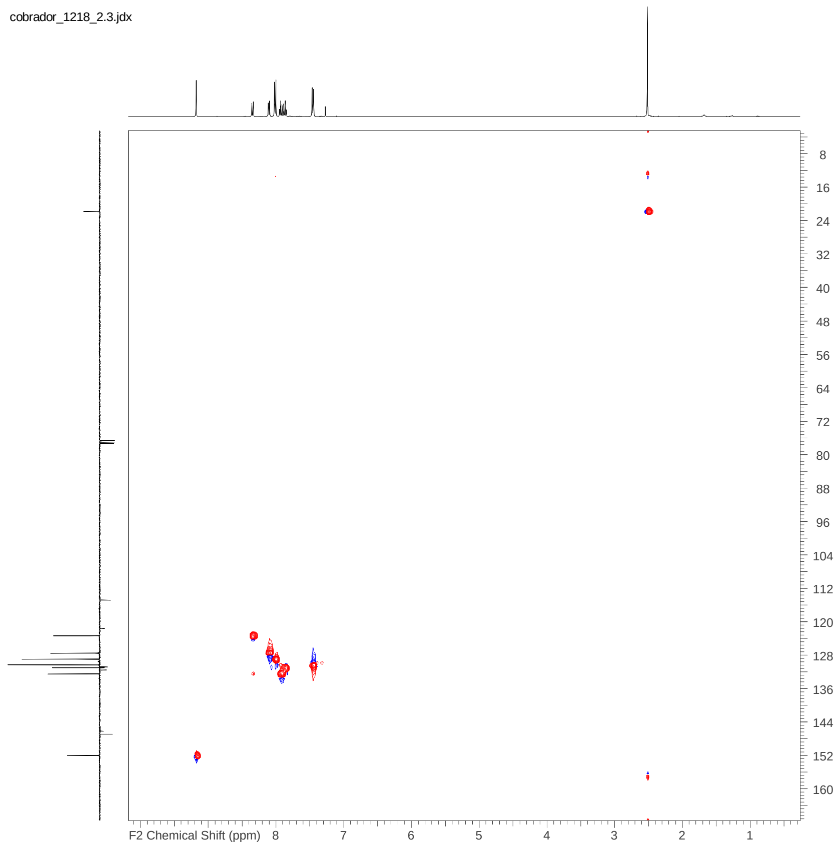
cobrador_1218_2.3.jdx
F2 Chemical Shift (ppm)

cobrador_1218_2.4.jdx

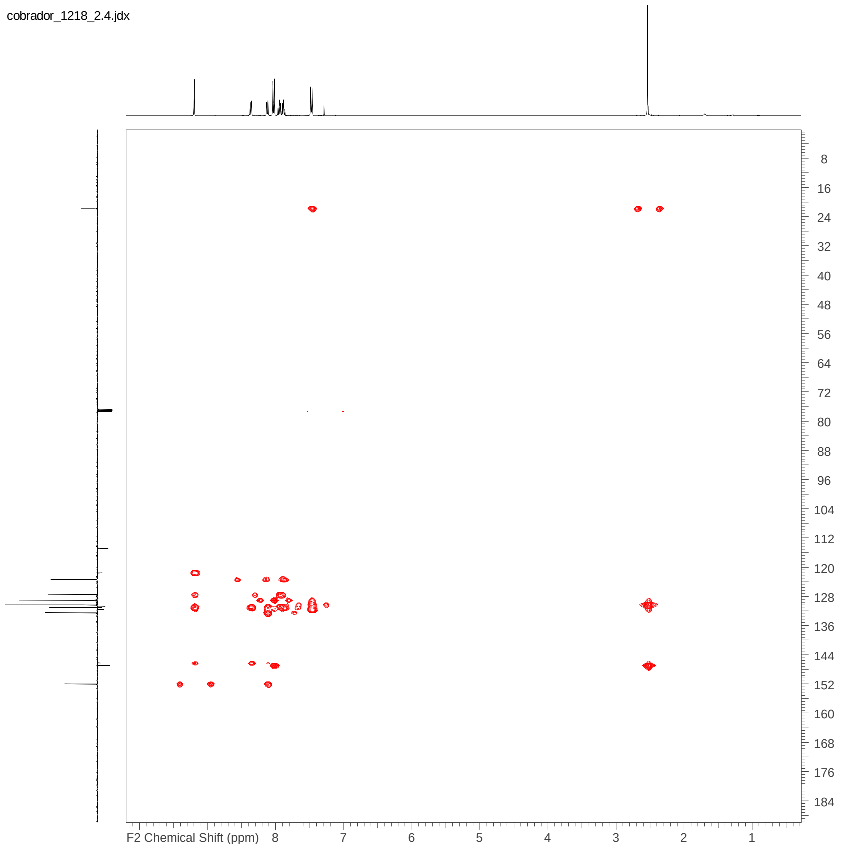

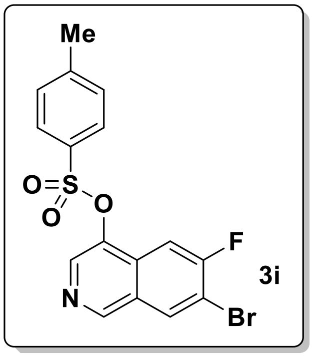
Me
O=S
O
O
F
N
Br
3i

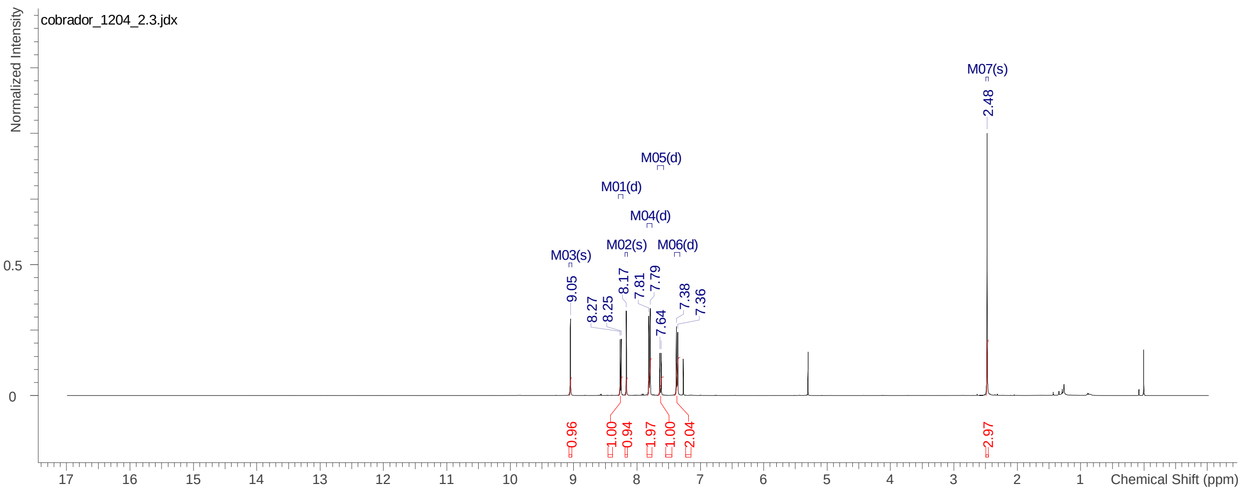
cobrador_1204_2.3.jdx
Normalized Intensity
M07(s)
2.48
M05(d)
M01(d)
M04(d)
M03(s)
M02(s)
M06(d)
9.05
8.27
8.25
8.17
7.81
7.79
7.64
7.38
7.36
0.96
1.00
0.94
1.97
1.00
2.04
2.97
Chemical Shift (ppm)

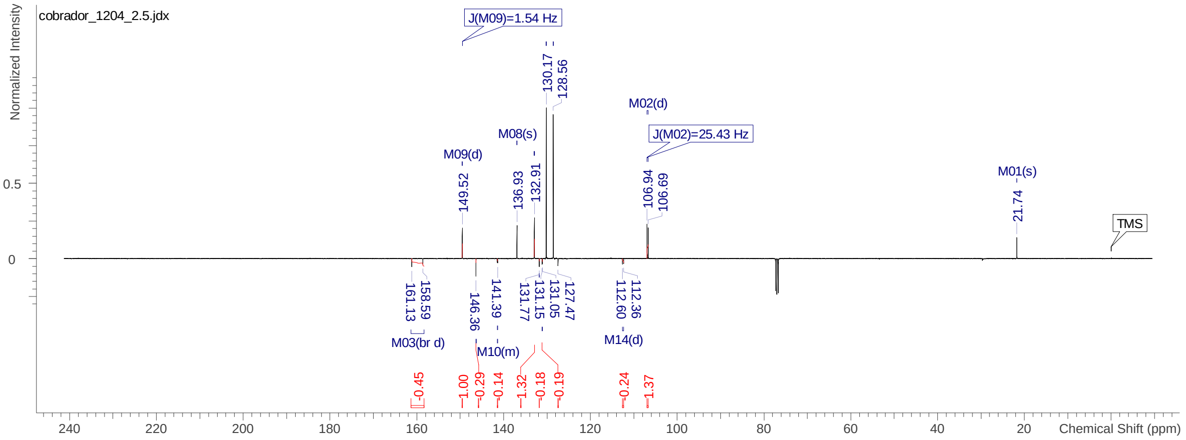
cobrador_1204_2.5.jdx
Normalized Intensity
J(M09)=1.54 Hz
130.17
128.56
M02(d)
J(M02)=25.43 Hz
M08(s)
M09(d)
149.52
136.93
132.91
106.94
106.69
M01(s)
21.74
TMS
161.13
158.59
146.36
141.39
131.77
131.15
131.05
127.47
112.60
112.36
M03(br d)
M10(m)
M14(d)
0.45
1.00
0.29
0.14
1.32
0.18
0.19
0.24
1.37
Chemical Shift (ppm)

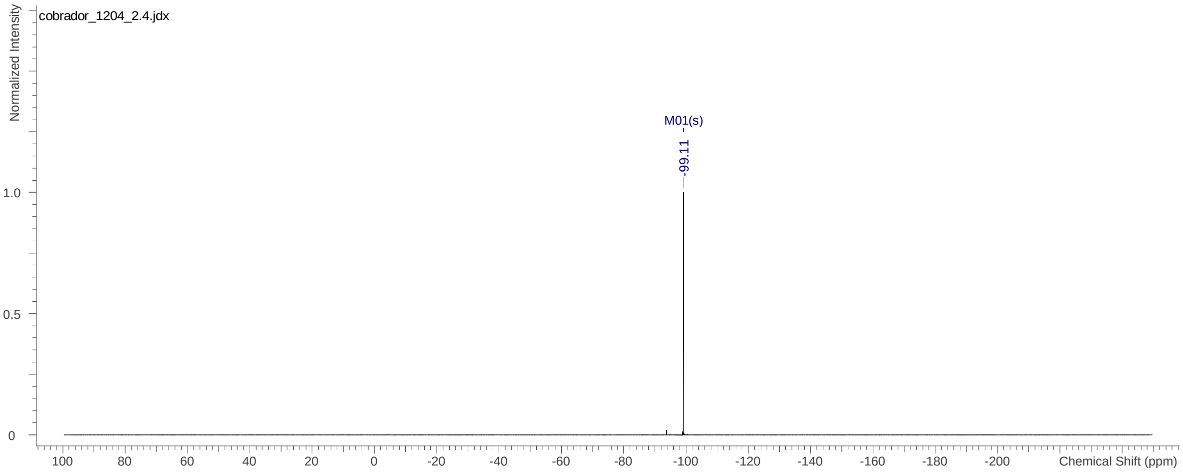
cobrador_1204_2.4.jdx
Normalized Intensity
M01(s)
-99.11
1.0
0.5
0
100
80
60
40
20
0
-20
-40
-60
-80
-100
-120
-140
-160
-180
-200
Chemical Shift (ppm)

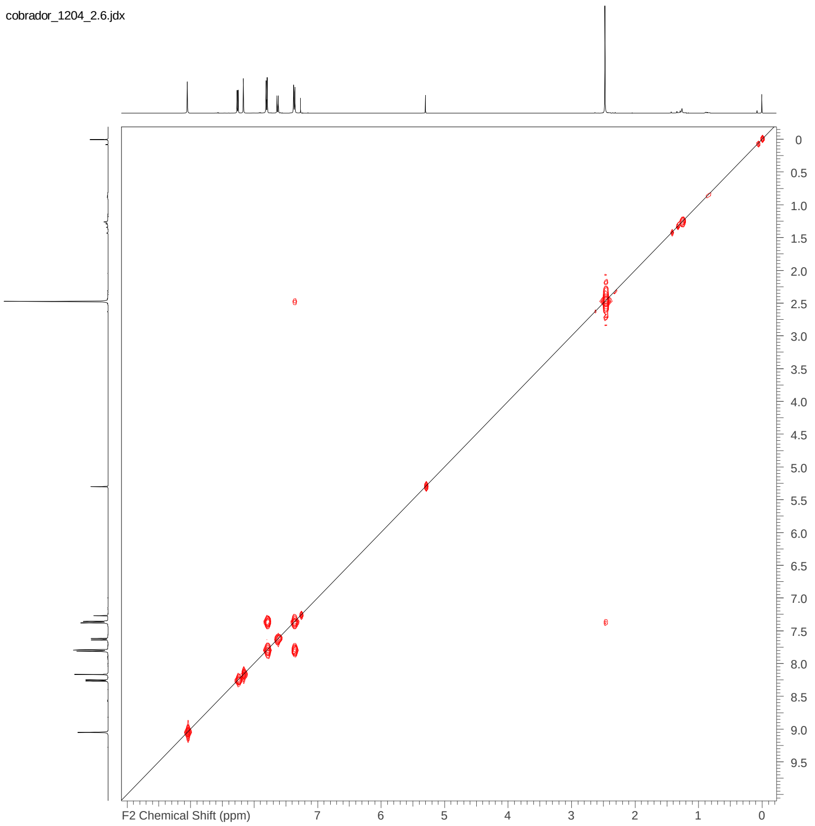
cobrador_1204_2.6.jdx
F2 Chemical Shift (ppm)
7
6
5
4
3
2
1
0
0.5
1.0
1.5
2.0
2.5
3.0
3.5
4.0
4.5
5.0
5.5
6.0
6.5
7.0
7.5
8.0
8.5
9.0
9.5

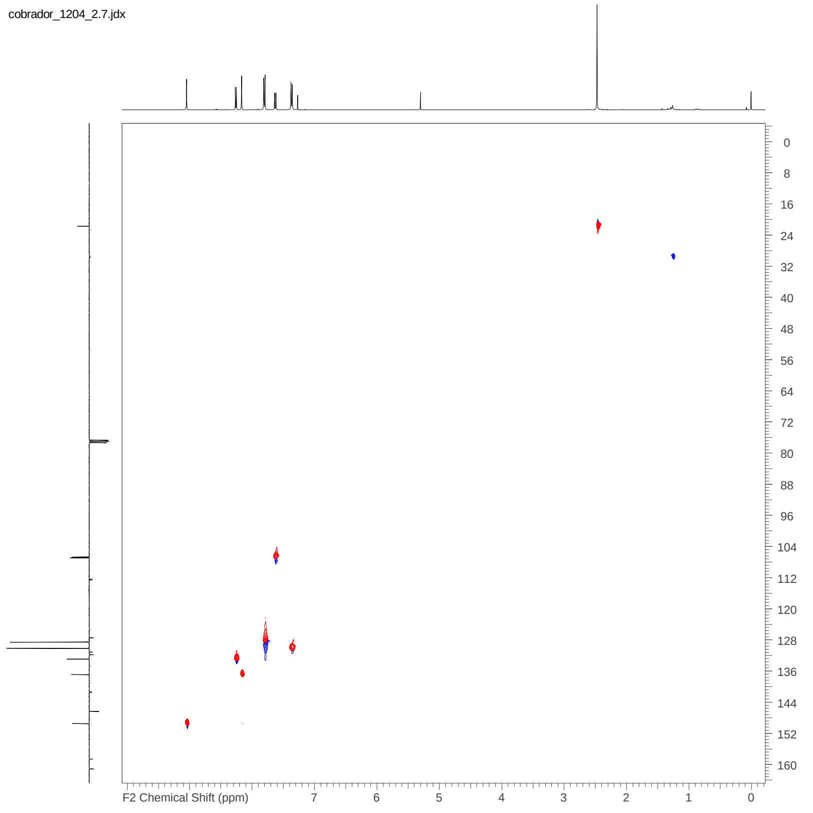
cobrador_1204_2.7.jdx
F2 Chemical Shift (ppm)
7
6
5
4
3
2
1
0
8
16
24
32
40
48
56
64
72
80
88
96
104
112
120
128
136
144
152
160

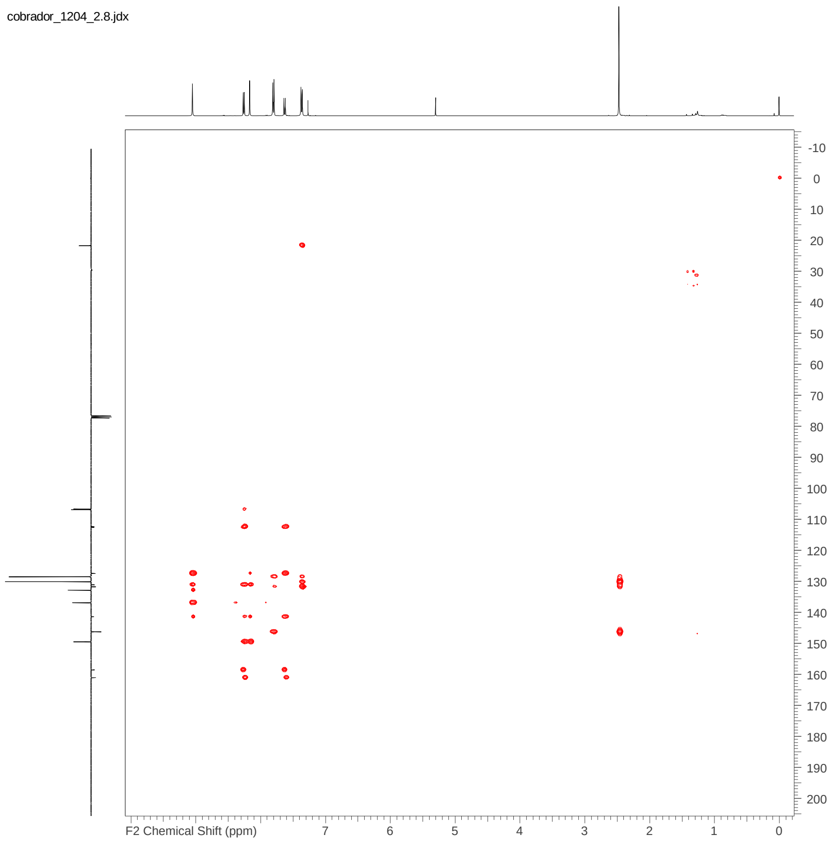
cobrador_1204_2.8.jdx
F2 Chemical Shift (ppm)
7
6
5
4
3
2
1
0
-10
10
20
30
40
50
60
70
80
90
100
110
120
130
140
150
160
170
180
190
200

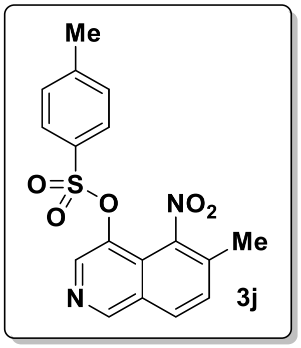
Me
O
S
O
O
NO2
Me
N
3j

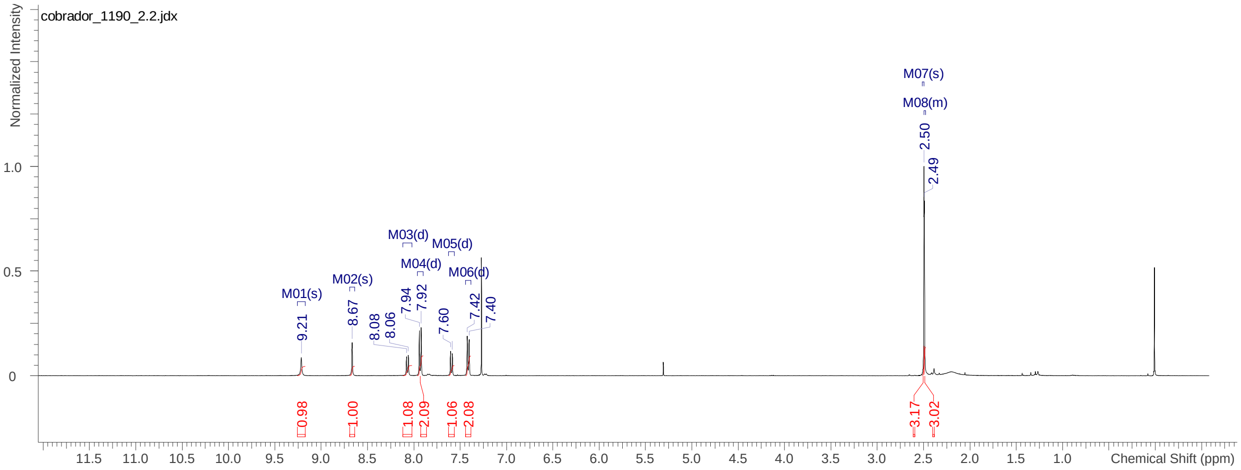
cobrador_1190_2.2.jdx
Normalized Intensity
Chemical Shift (ppm)
M01(s)
M02(s)
M03(d)
M04(d)
M05(d)
M06(d)
M07(s)
M08(m)
9.21
8.67
8.08
8.06
7.94
7.92
7.60
7.42
7.40
2.50
2.49
0.98
1.00
1.08
2.09
1.06
2.08
3.17
3.02

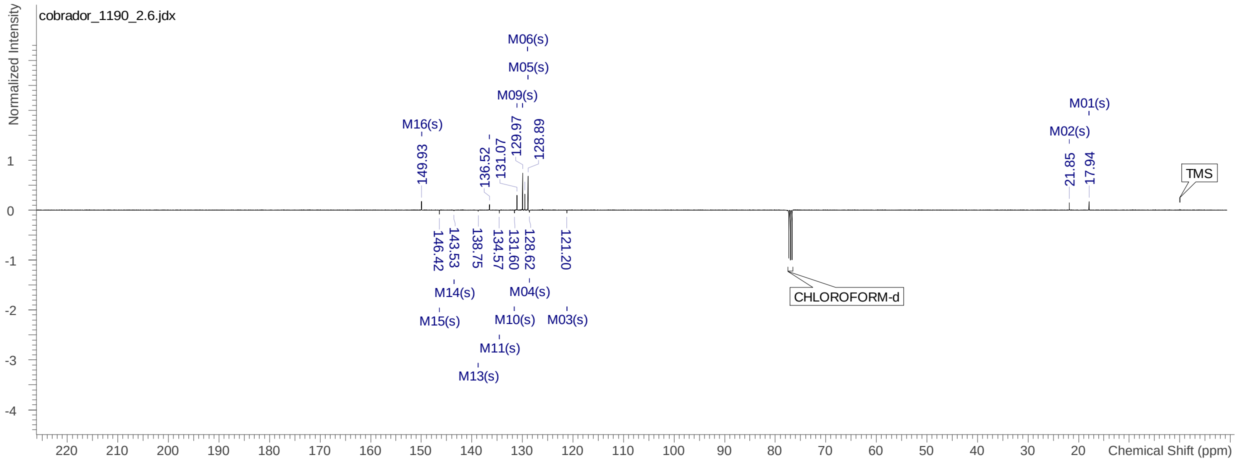
cobrador_1190_2.6.jdx
Normalized Intensity
Chemical Shift (ppm)
M16(s)
M09(s)
M05(s)
M06(s)
M01(s)
M02(s)
149.93
136.52
131.07
129.97
128.89
21.85
17.94
TMS
146.42
143.53
138.75
134.57
131.60
128.62
121.20
M14(s)
M15(s)
M04(s)
M10(s)
M03(s)
M11(s)
M13(s)
CHLOROFORM-d

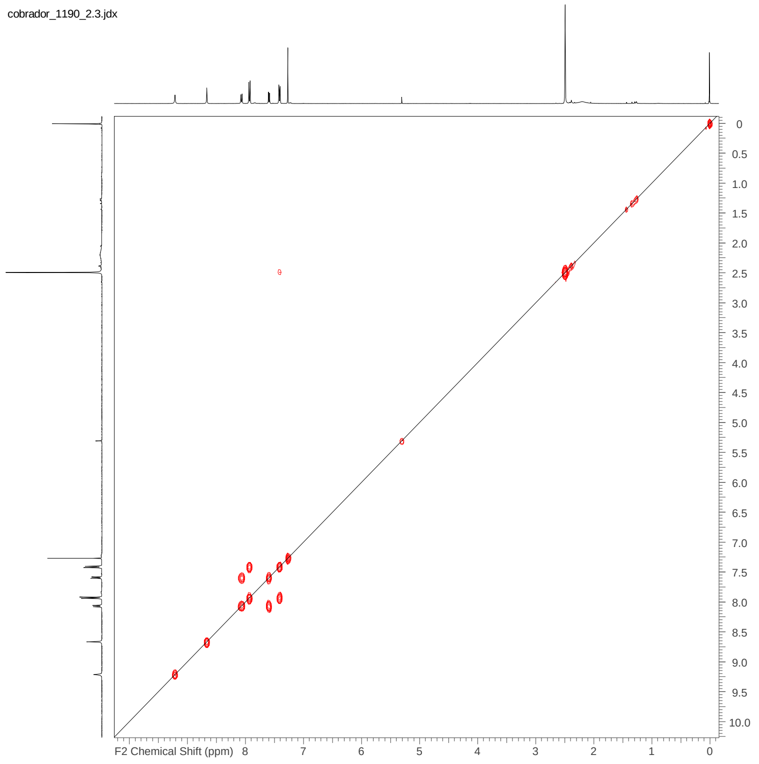
cobrador_1190_2.3.jdx
F2 Chemical Shift (ppm)

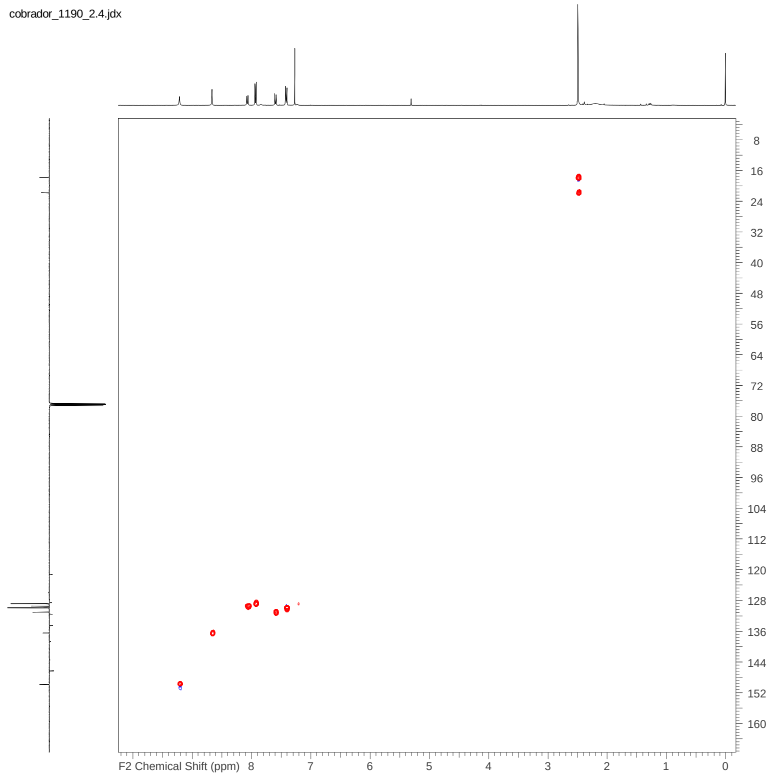
cobrador_1190_2.4.jdx
F2 Chemical Shift (ppm)

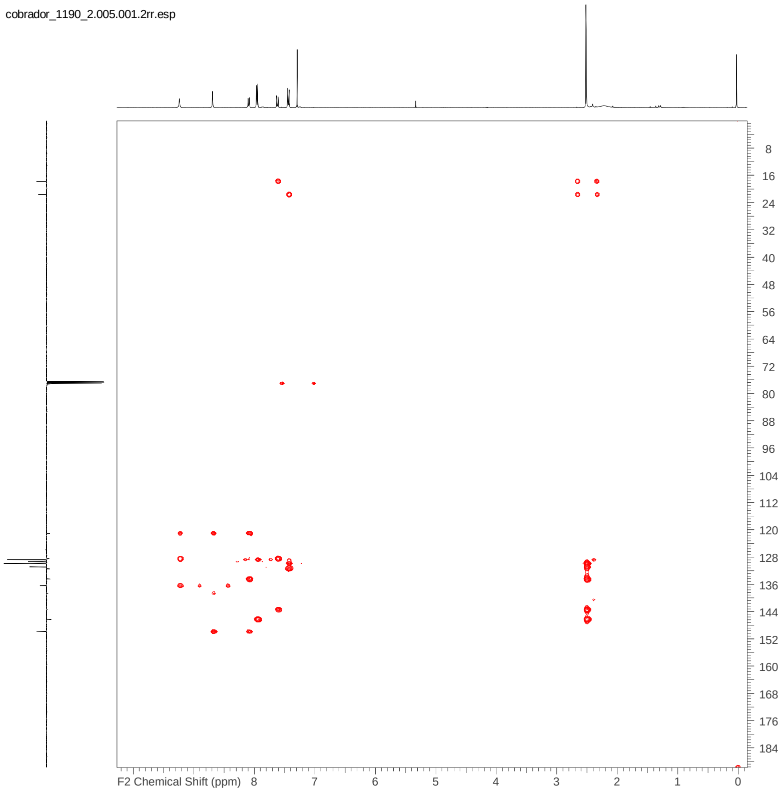
cobrador_1190_2.005.001.2rr.esp
F2 Chemical Shift (ppm)
8
7
6
5
4
3
2
1
0
8
16
24
32
40
48
56
64
72
80
88
96
104
112
120
128
136
144
152
160
168
176
184

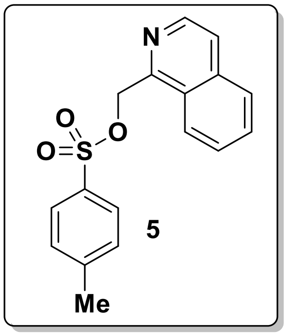
N
O
O
S
O
Me
5

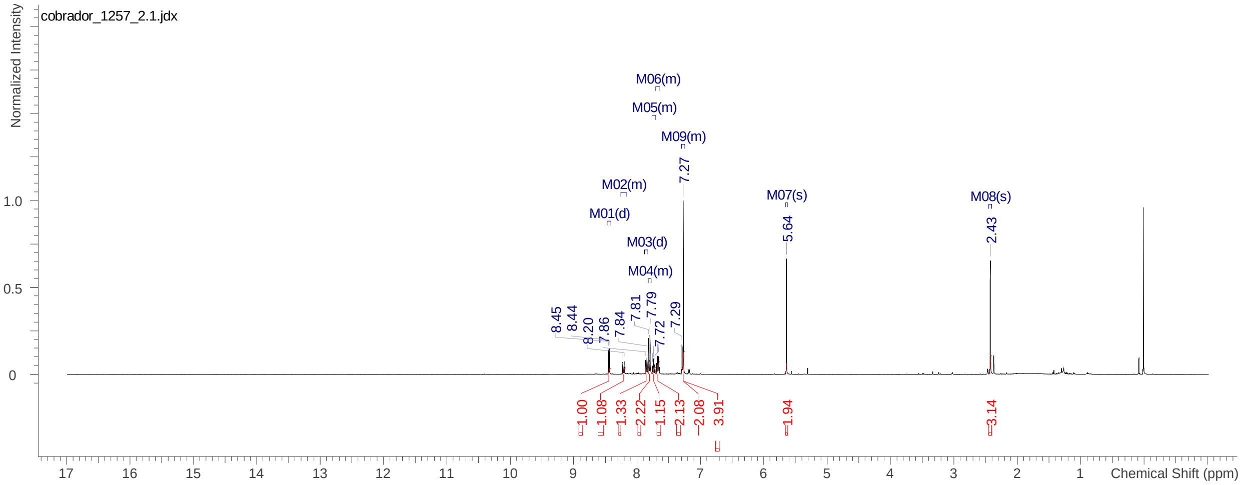
cobrador_1257_2.1.jdx
Normalized Intensity
M06(m)
M05(m)
M09(m)
M02(m)
M01(d)
M03(d)
M04(m)
M07(s)
M08(s)
7.27
5.64
2.43
8.45
8.44
8.20
7.86
7.84
7.81
7.79
7.72
7.29
1.00
1.08
1.33
2.22
1.15
2.13
2.08
3.91
1.94
3.14
Chemical Shift (ppm)

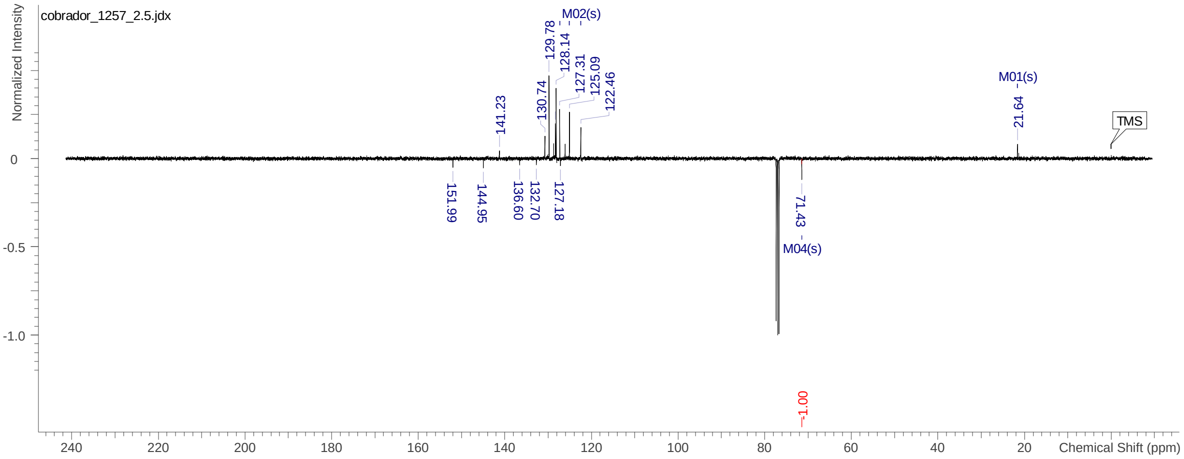
cobrador_1257_2.5.jdx
Normalized Intensity
M02(s)
129.78
128.14
127.31
125.09
122.46
130.74
141.23
M01(s)
21.64
TMS
151.99
144.95
136.60
132.70
127.18
71.43
M04(s)
1.00
Chemical Shift (ppm)

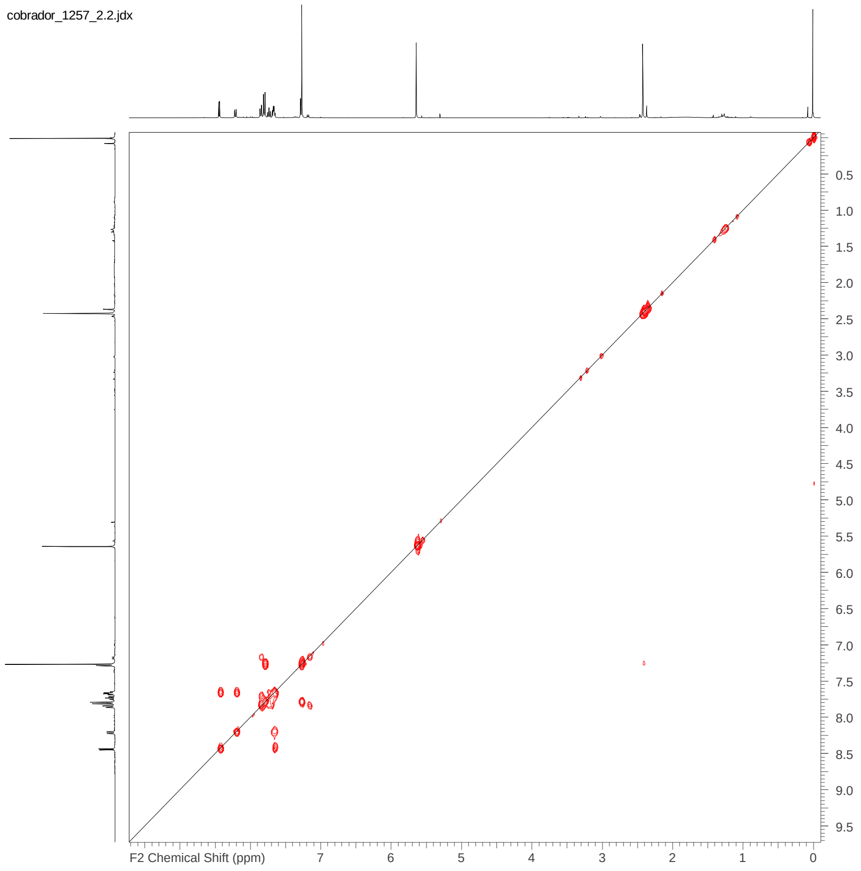
cobrador_1257_2.2.jdx
F2 Chemical Shift (ppm)
7
6
5
4
3
2
1
0
0.5
1.0
1.5
2.0
2.5
3.0
3.5
4.0
4.5
5.0
5.5
6.0
6.5
7.0
7.5
8.0
8.5
9.0
9.5

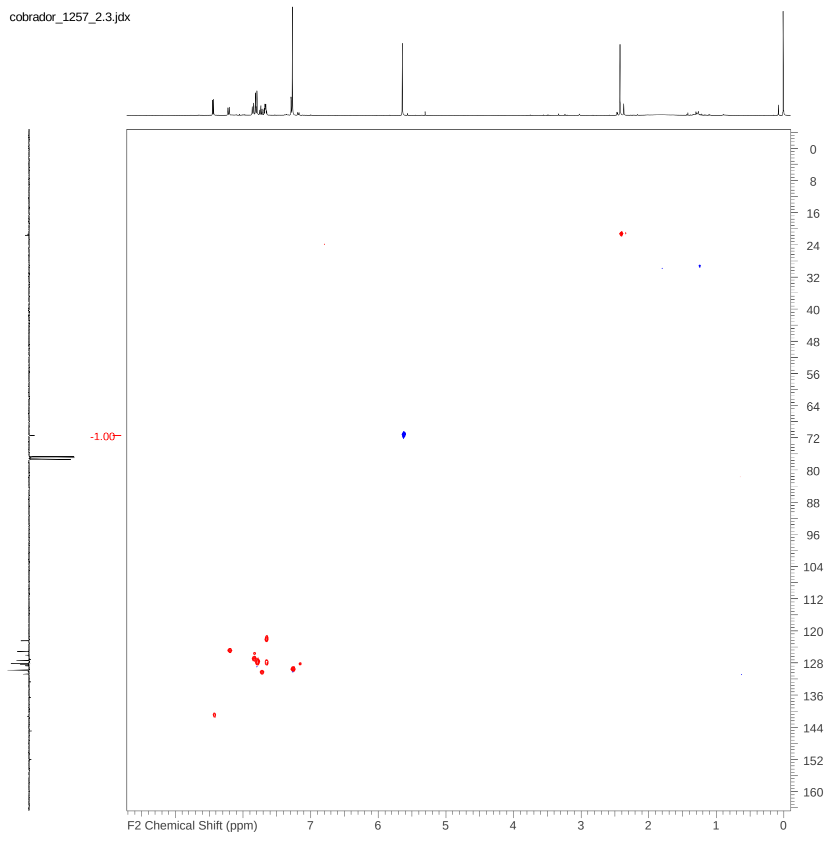
cobrador_1257_2.3.jdx
-1.00
F2 Chemical Shift (ppm)
7
6
5
4
3
2
1
0
0
8
16
24
32
40
48
56
64
72
80
88
96
104
112
120
128
136
144
152
160

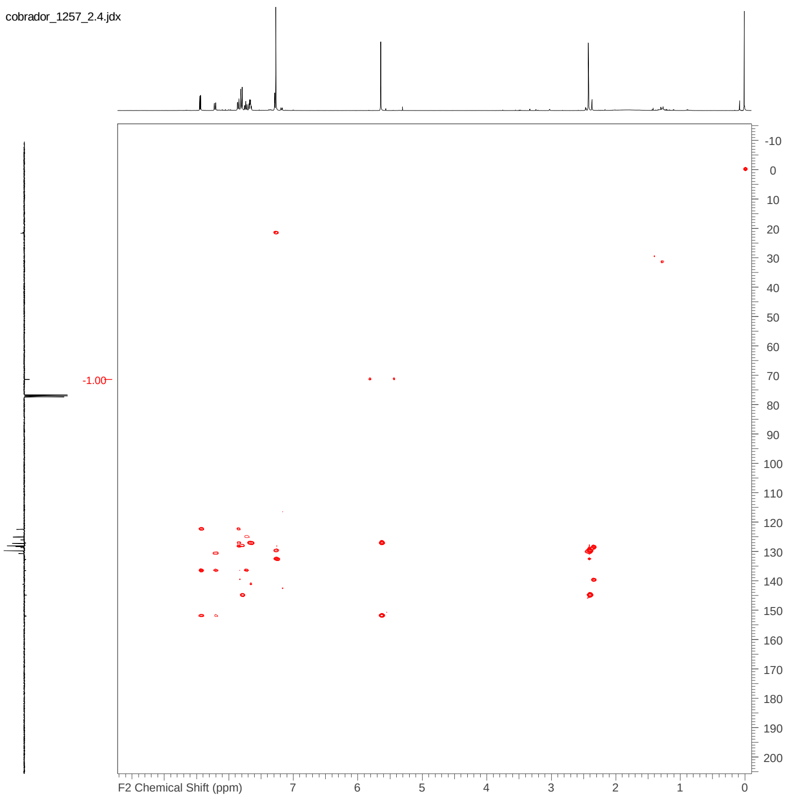
cobrador_1257_2.4.jdx
-1.00
-10
0
10
20
30
40
50
60
70
80
90
100
110
120
130
140
150
160
170
180
190
200
F2 Chemical Shift (ppm)
7
6
5
4
3
2
1
0

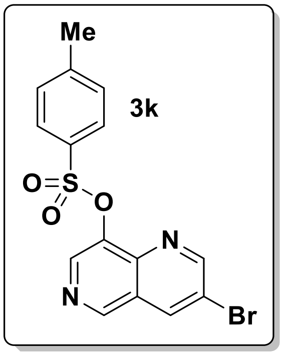
Me
O=S
O
O
N
N
Br

3k

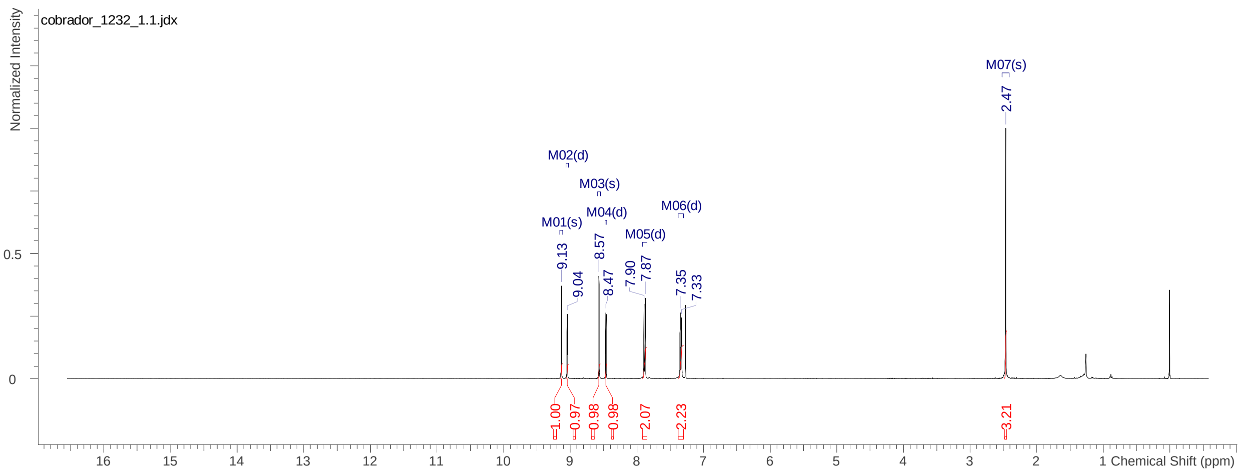
cobrador_1232_1.1.jdx
Normalized Intensity
M01(s)
M02(d)
M03(s)
M04(d)
M05(d)
M06(d)
M07(s)
9.13
9.04
8.57
8.47
7.90
7.87
7.35
7.33
2.47
1.00
0.97
0.98
0.98
2.07
2.23
3.21
Chemical Shift (ppm)

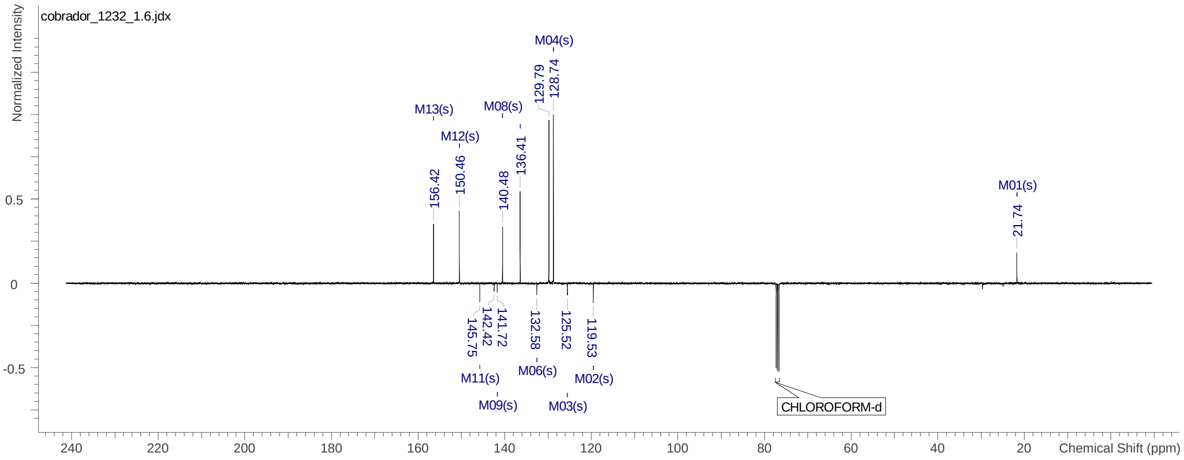
cobrador_1232_1.6.jdx
Normalized Intensity
M13(s)
M12(s)
M08(s)
M04(s)
M01(s)
156.42
150.46
140.48
136.41
129.79
128.74
21.74
145.75
142.42
141.72
132.58
125.52
119.53
M11(s)
M09(s)
M06(s)
M03(s)
M02(s)
CHLOROFORM-d
Chemical Shift (ppm)

cobrador_1232_1.3.jdx

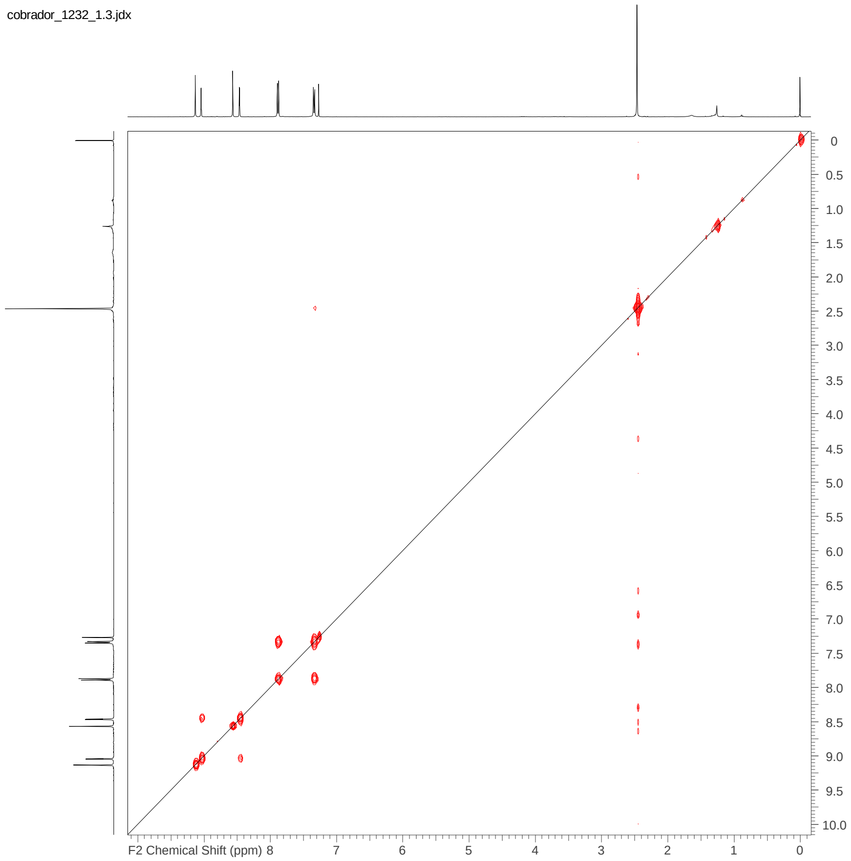

cobrador_1232_1.4.jdx

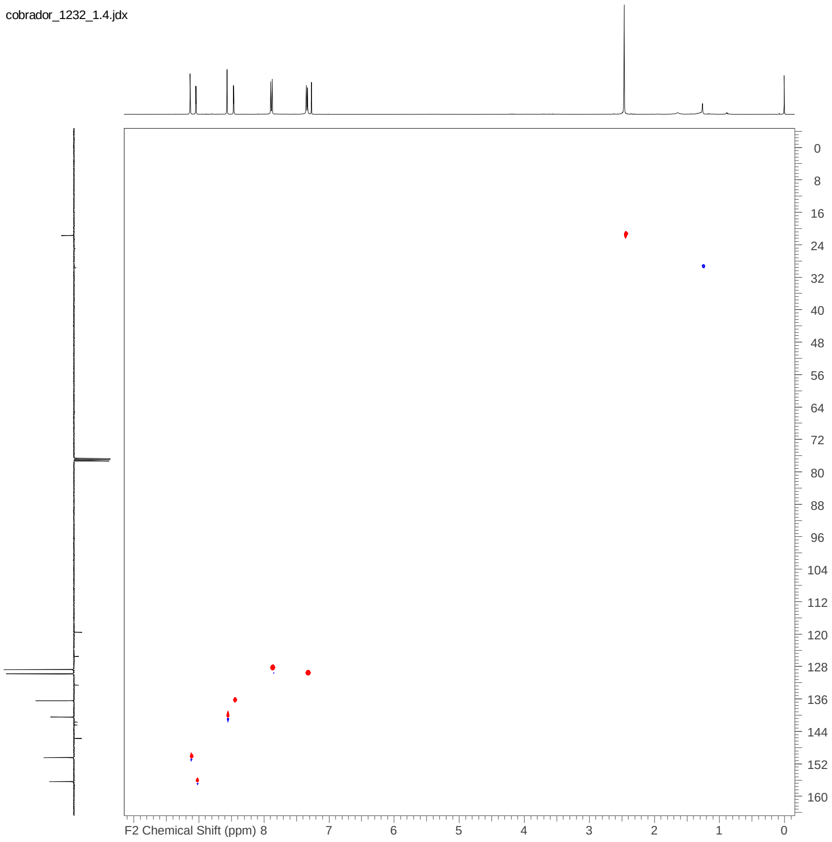

cobrador_1232_1.5.jdx

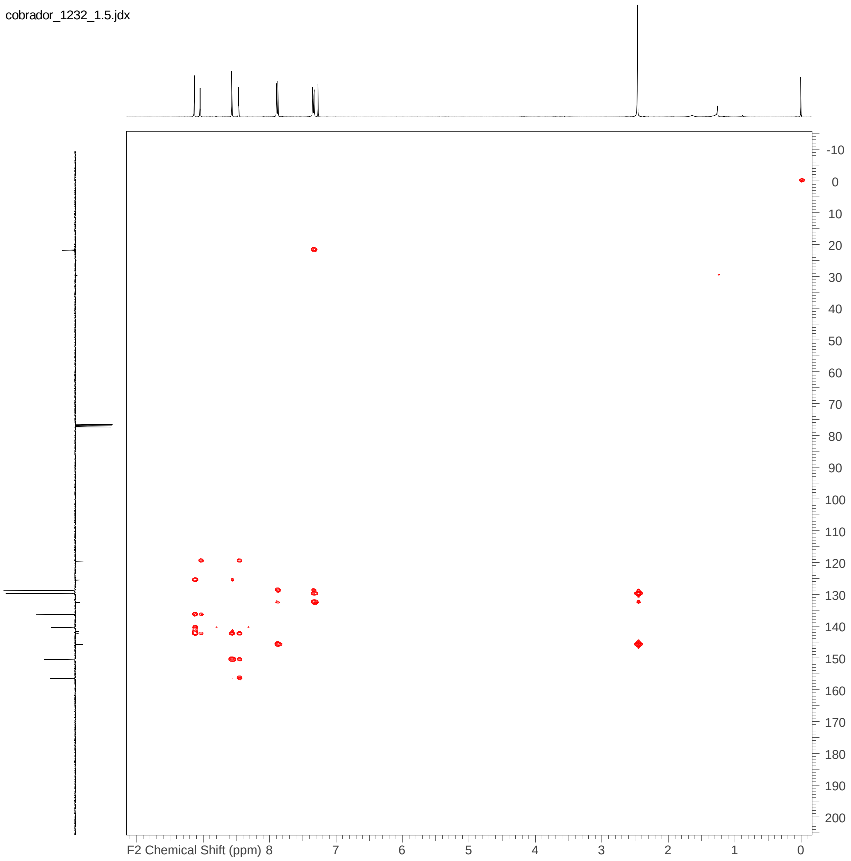

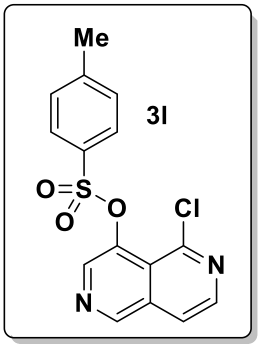

Me
O
S
O
O
Cl
N
N

3l

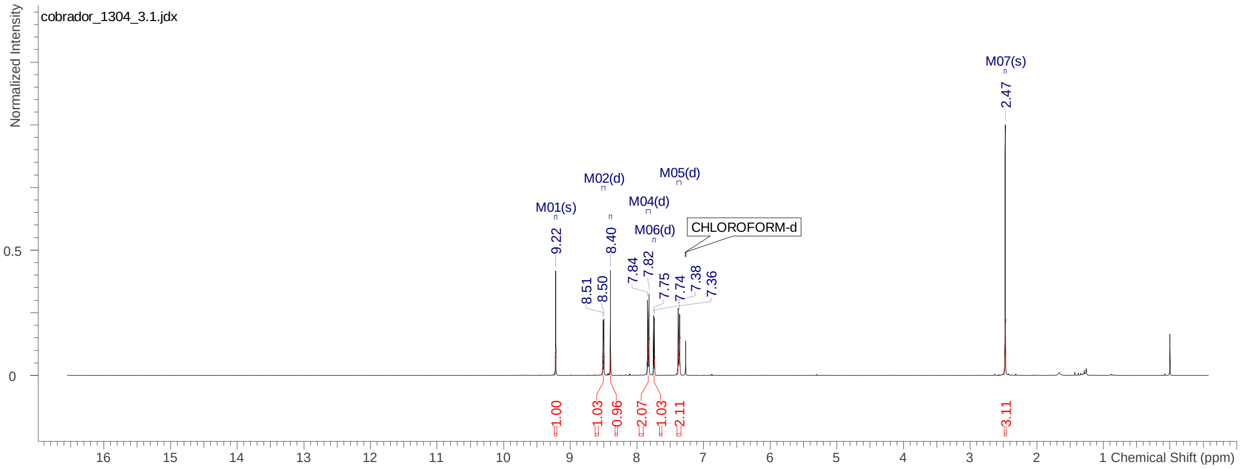

cobrador_1304_3.1.jdx
Normalized Intensity
M07(s)
2.47
M02(d)
M05(d)
M01(s)
M04(d)
M06(d)
CHLOROFORM-d
9.22
8.40
8.51
8.50
7.84
7.82
7.75
7.74
7.38
7.36
0.5
0
1.00
1.03
0.96
2.07
1.03
2.11
3.11
16
15
14
13
12
11
10
9
8
7
6
5
4
3
2
1 Chemical Shift (ppm)

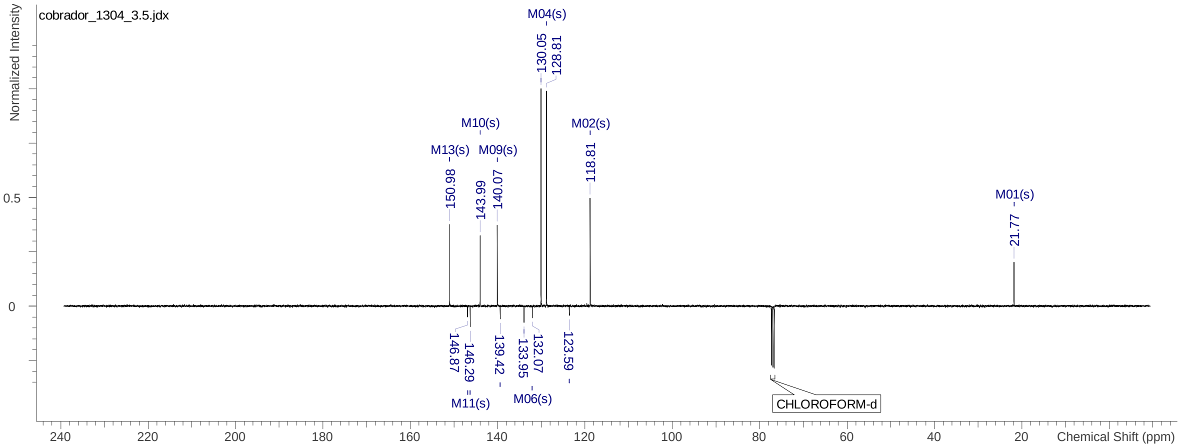

cobrador_1304_3.5.jdx
Normalized Intensity
M04(s)
130.05
128.81
M10(s)
M02(s)
M13(s)
M09(s)
150.98
143.99
140.07
118.81
M01(s)
21.77
0.5
0
146.87
146.29
139.42
133.95
132.07
123.59
M11(s)
M06(s)
CHLOROFORM-d
240
220
200
180
160
140
120
100
80
60
40
20
Chemical Shift (ppm)

cobrador_1304_3.2.jdx

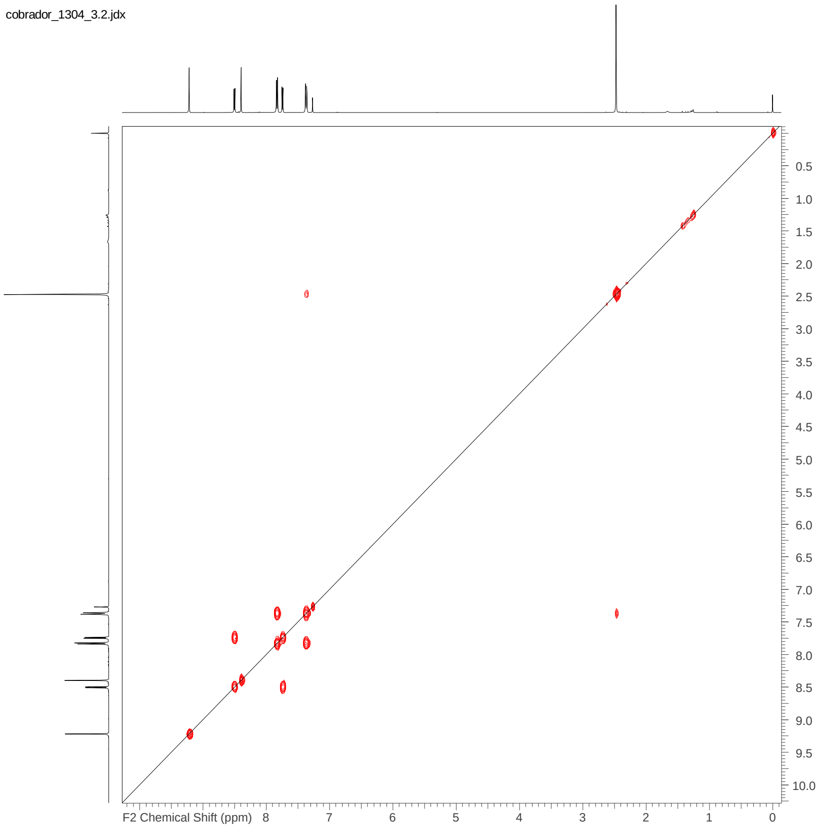

cobrador_1304_3.3.jdx

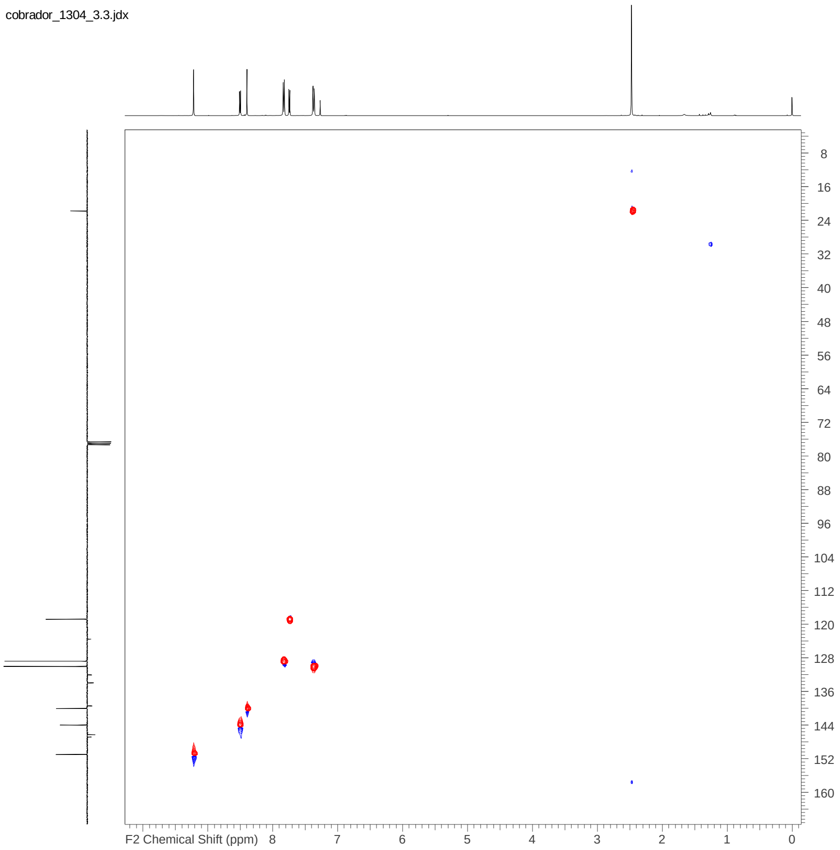

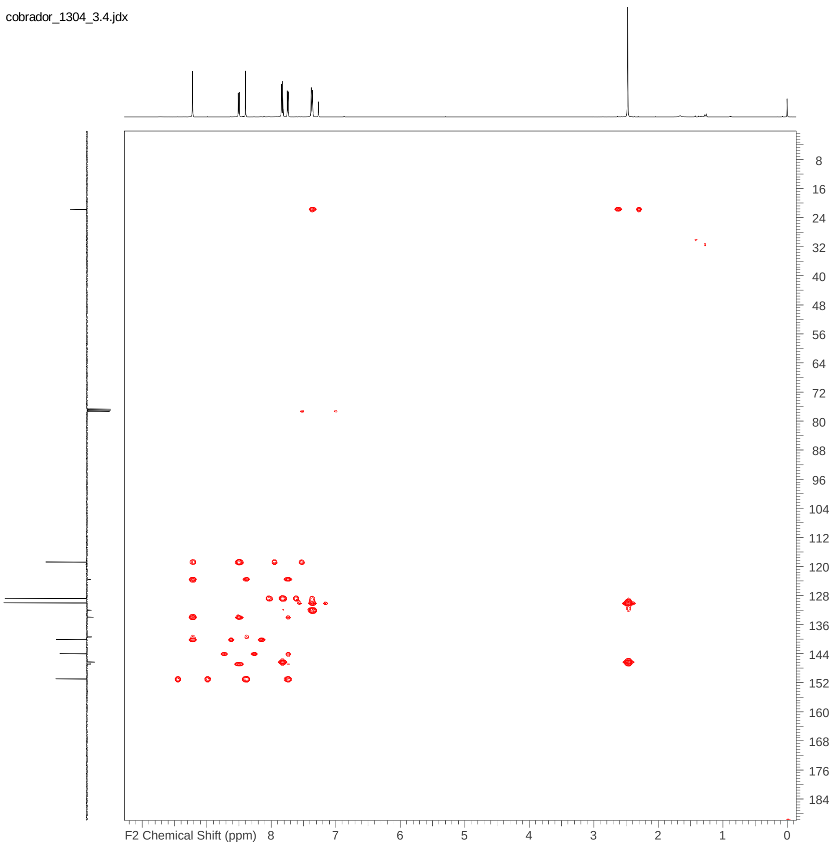

cobrador_1304_3.4.jdx
F2 Chemical Shift (ppm)

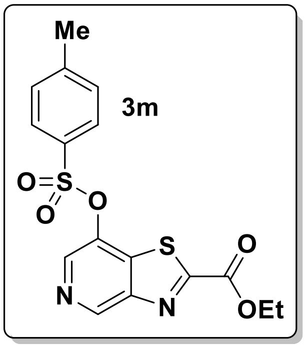
Me
3m
O
S
O
O
S
O
N
N
OEt

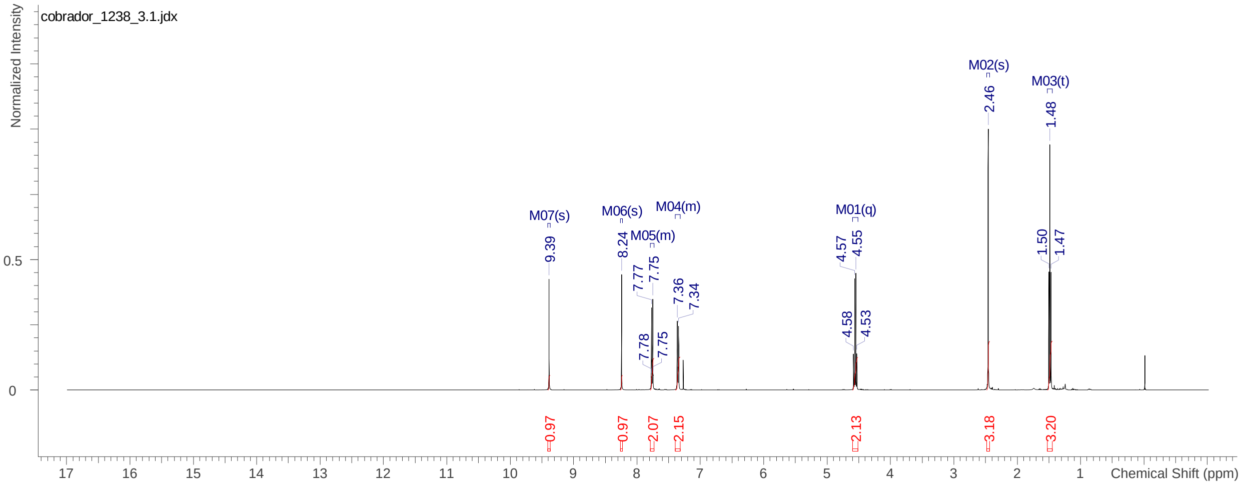
cobrador_1238_3.1.jdx
Normalized Intensity
Chemical Shift (ppm)

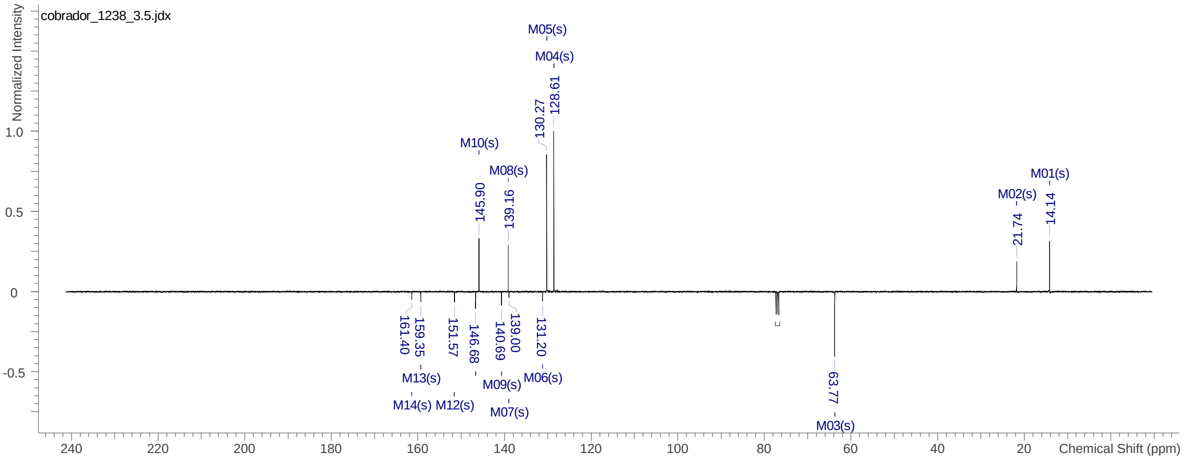
cobrador_1238_3.5.jdx
Normalized Intensity
Chemical Shift (ppm)

cobrador_1238_3.2.jdx

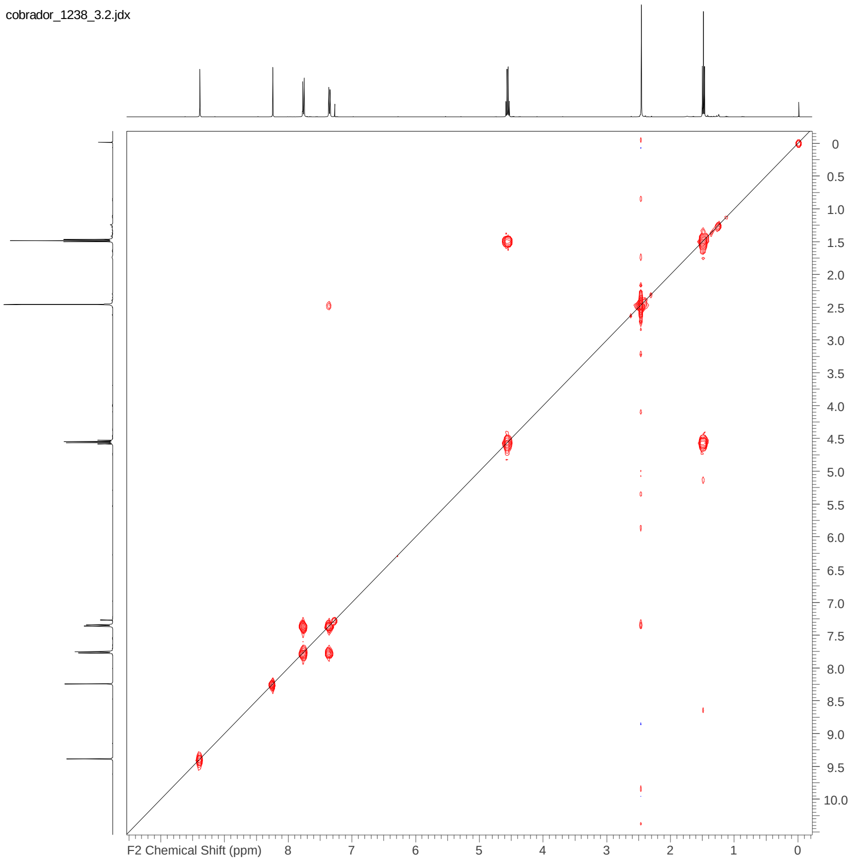

cobrador_1238_3.3.jdx

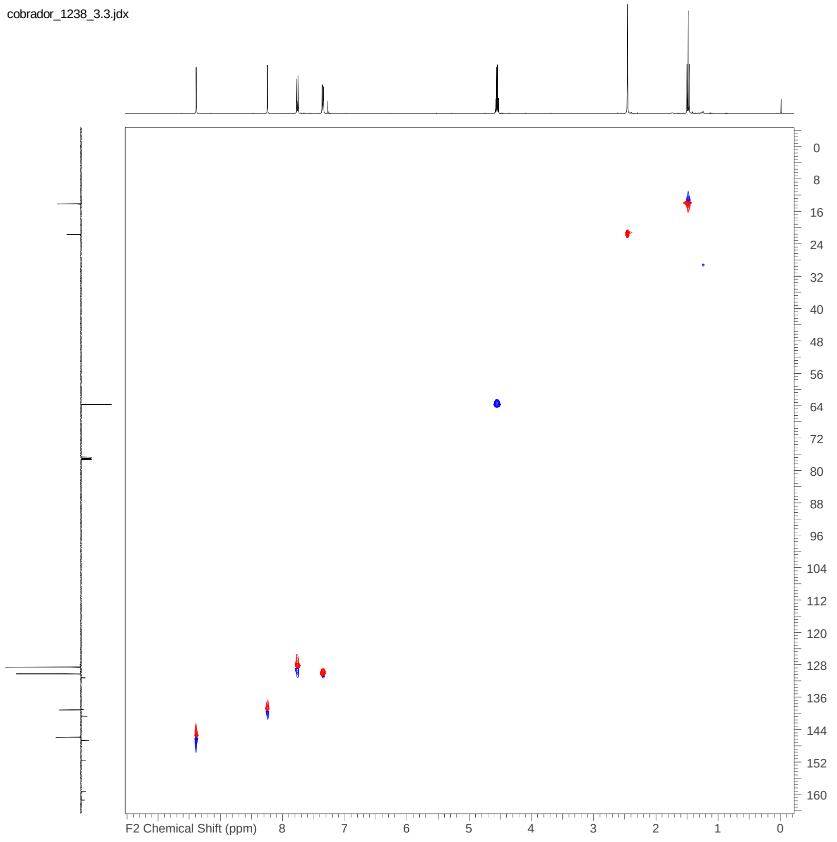

cobrador_1238_3.4.jdx

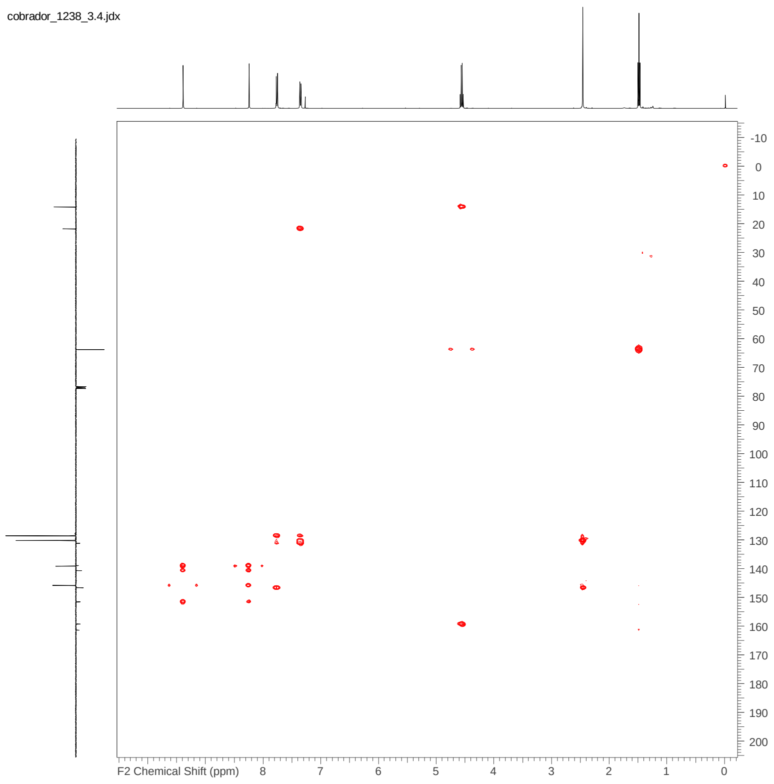

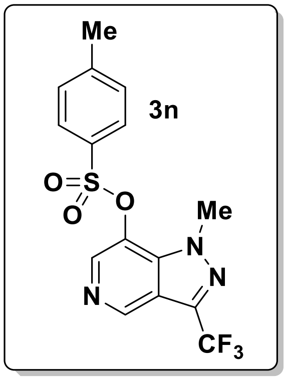

Me
3n
O
S
O
O
Me
N
N
N
CF3

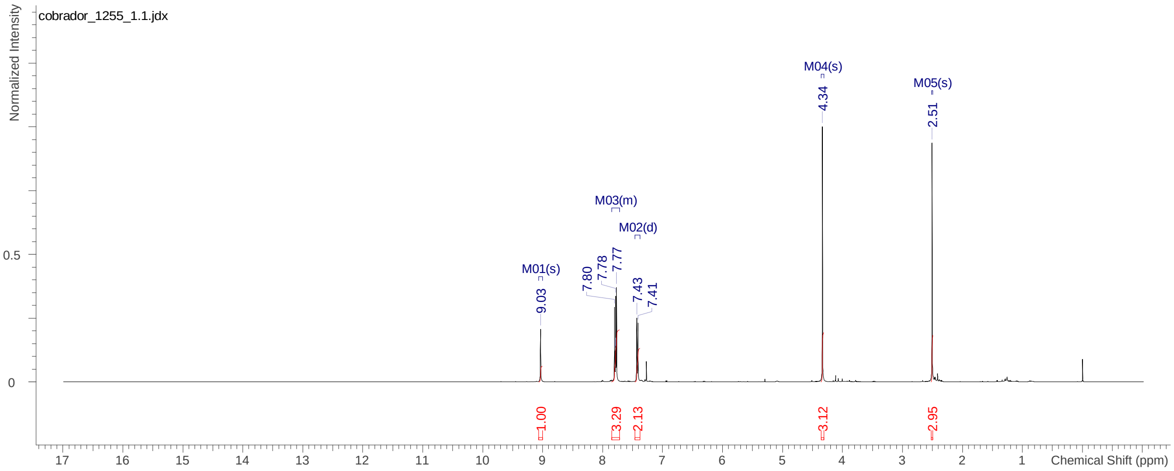

cobrador_1255_1.1.jdx
Normalized Intensity
M04(s)
4.34
M05(s)
2.51
M03(m)
M02(d)
M01(s)
7.80
7.78
7.77
7.43
7.41
9.03
1.00
3.29
2.13
3.12
2.95
Chemical Shift (ppm)

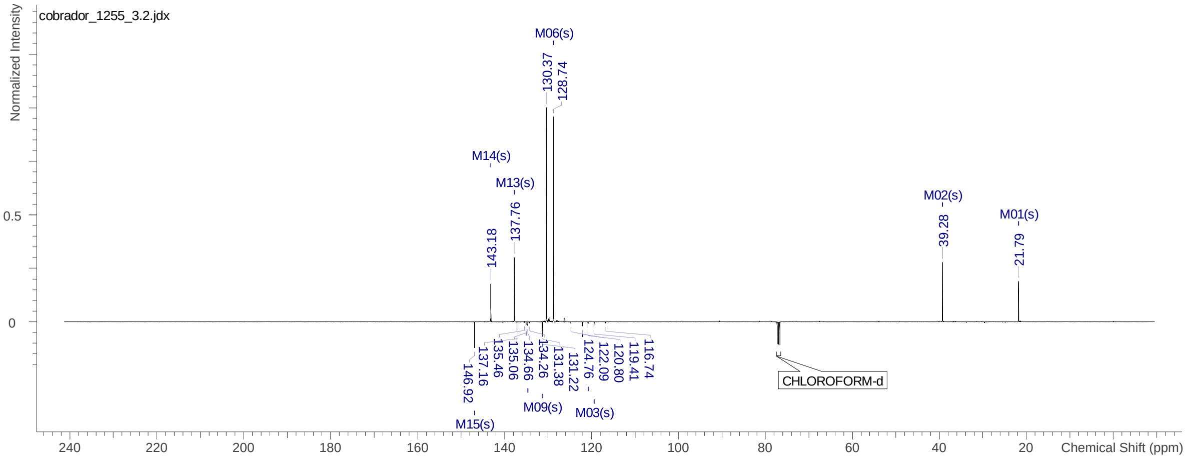

cobrador_1255_3.2.jdx
Normalized Intensity
M06(s)
130.37
128.74
M14(s)
M13(s)
137.76
143.18
M02(s)
39.28
M01(s)
21.79
146.92
137.16
135.46
135.06
134.66
134.26
131.38
131.22
124.76
122.09
120.80
119.41
116.74
M15(s)
M09(s)
M03(s)
CHLOROFORM-d
Chemical Shift (ppm)

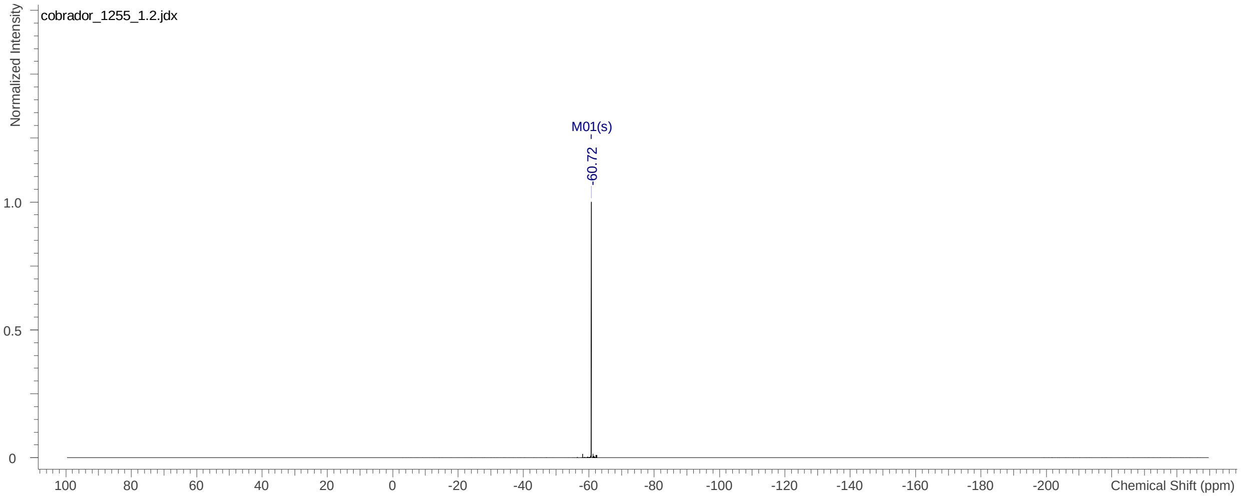
cobrador_1255_1.2.jdx
Normalized Intensity
M01(s)
-60.72
Chemical Shift (ppm)

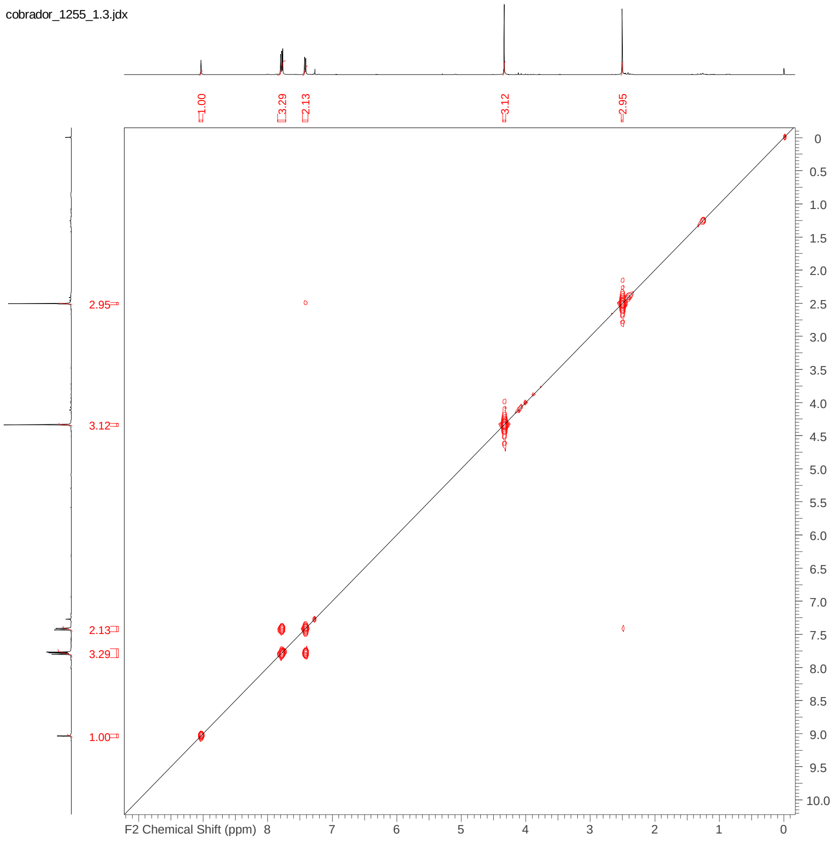
cobrador_1255_1.3.jdx
1.00
3.29
2.13
3.12
2.95
F2 Chemical Shift (ppm)

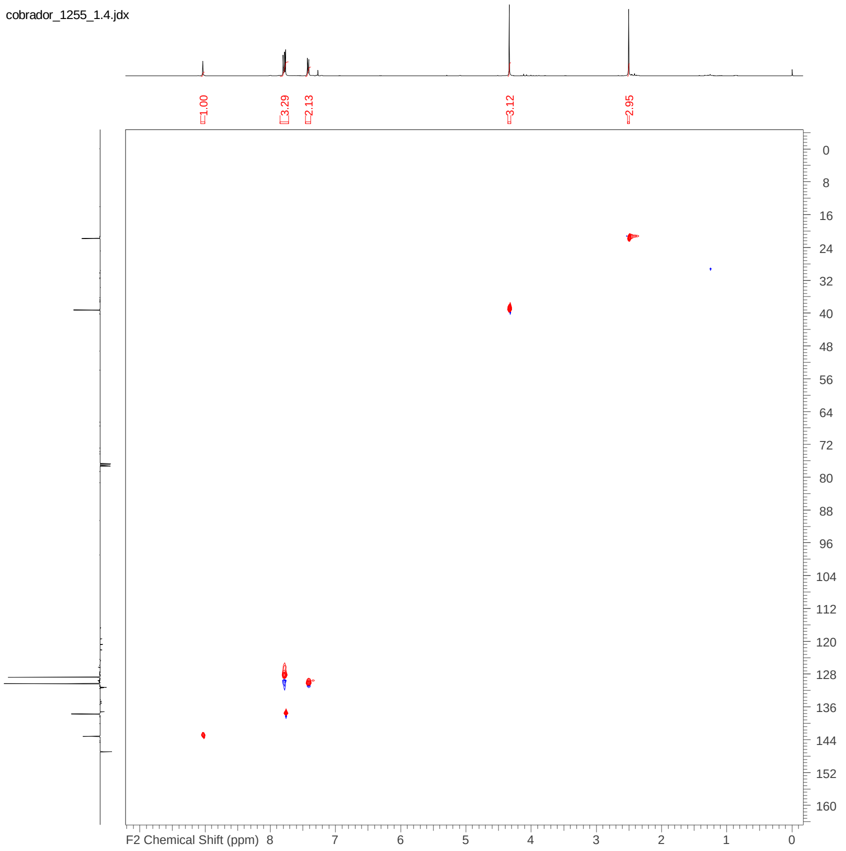
cobrador_1255_1.4.jdx
1.00
3.29
2.13
3.12
2.95
F2 Chemical Shift (ppm)

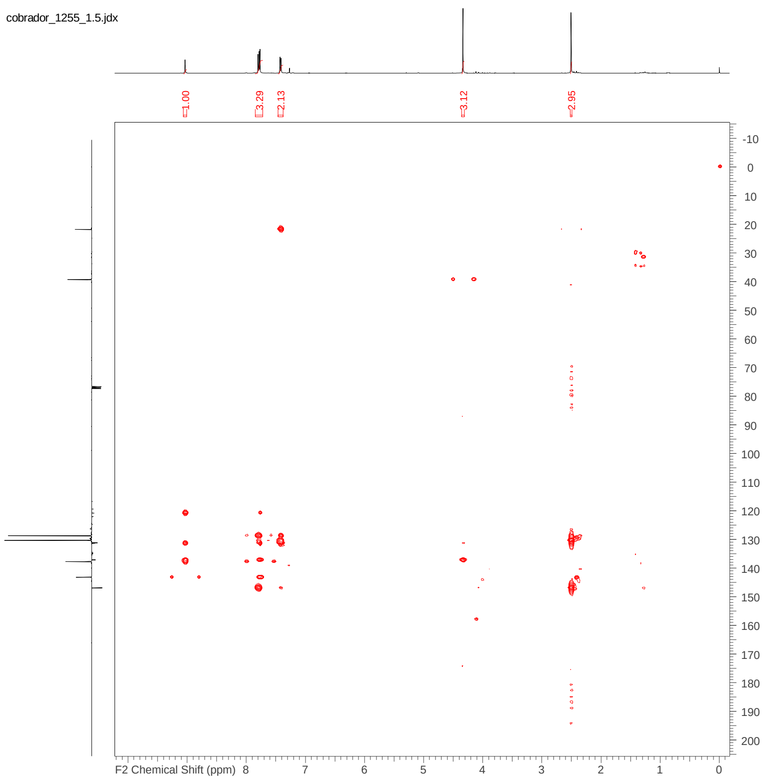
cobrador_1255_1.5.jdx
1.00
3.29
2.13
3.12
2.95
F2 Chemical Shift (ppm)

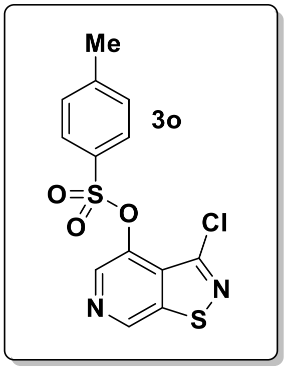
Me
3o
O
S
O
O
Cl
N
N
S

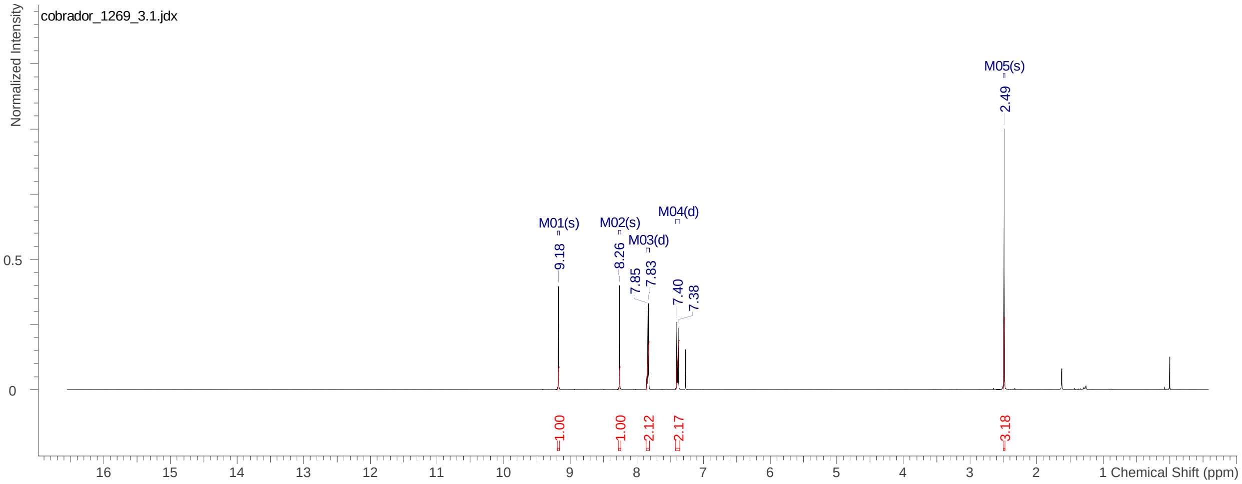
cobrador_1269_3.1.jdx
Normalized Intensity
M01(s)
9.18
M02(s)
8.26
M03(d)
7.85
7.83
M04(d)
7.40
7.38
M05(s)
2.49
1.00
1.00
2.12
2.17
3.18
0.5
0
16 15 14 13 12 11 10 9 8 7 6 5 4 3 2 1
Chemical Shift (ppm)

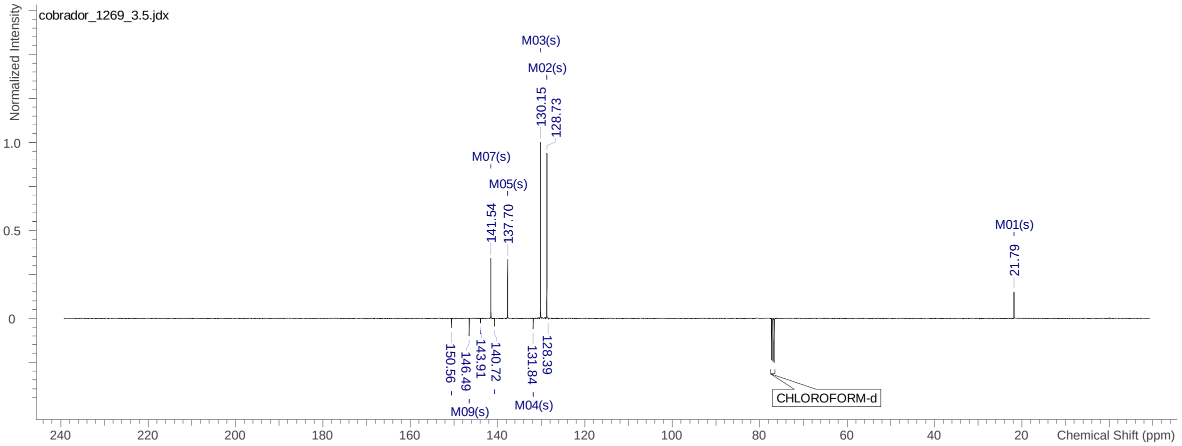
cobrador_1269_3.5.jdx
Normalized Intensity
M03(s)
M02(s)
130.15
128.73
M07(s)
M05(s)
141.54
137.70
M01(s)
21.79
150.56
146.49
143.91
140.72
131.84
128.39
M09(s)
M04(s)
CHLOROFORM-d
1.0
0.5
0
240 220 200 180 160 140 120 100 80 60 40 20
Chemical Shift (ppm)

cobrador_1269_3.2.jdx

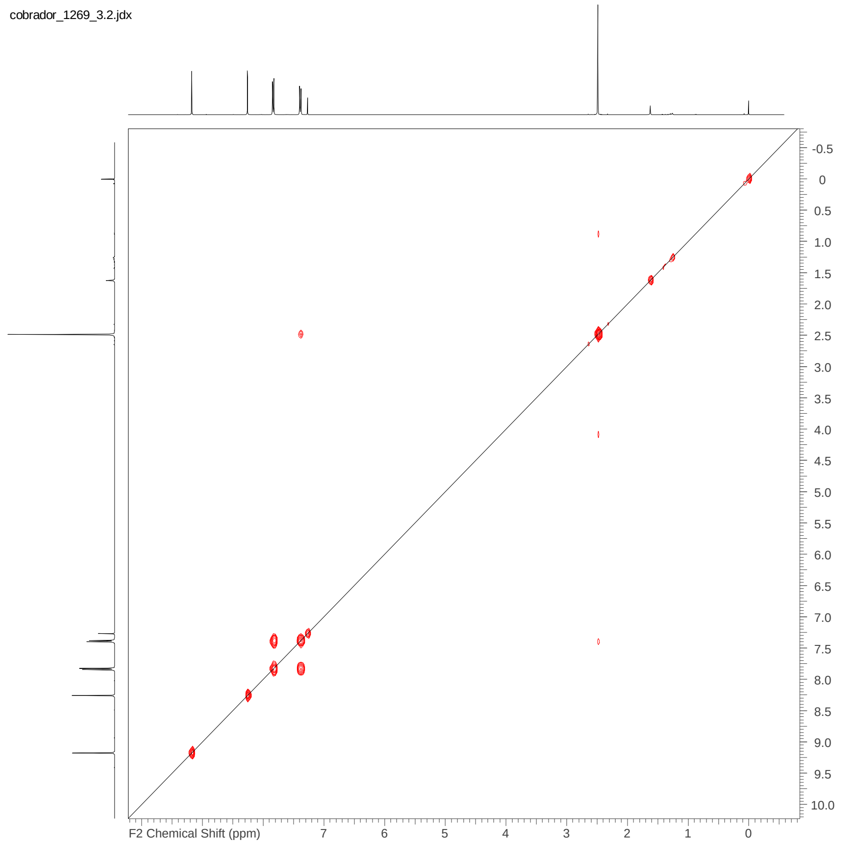

cobrador_1269_3.3.jdx

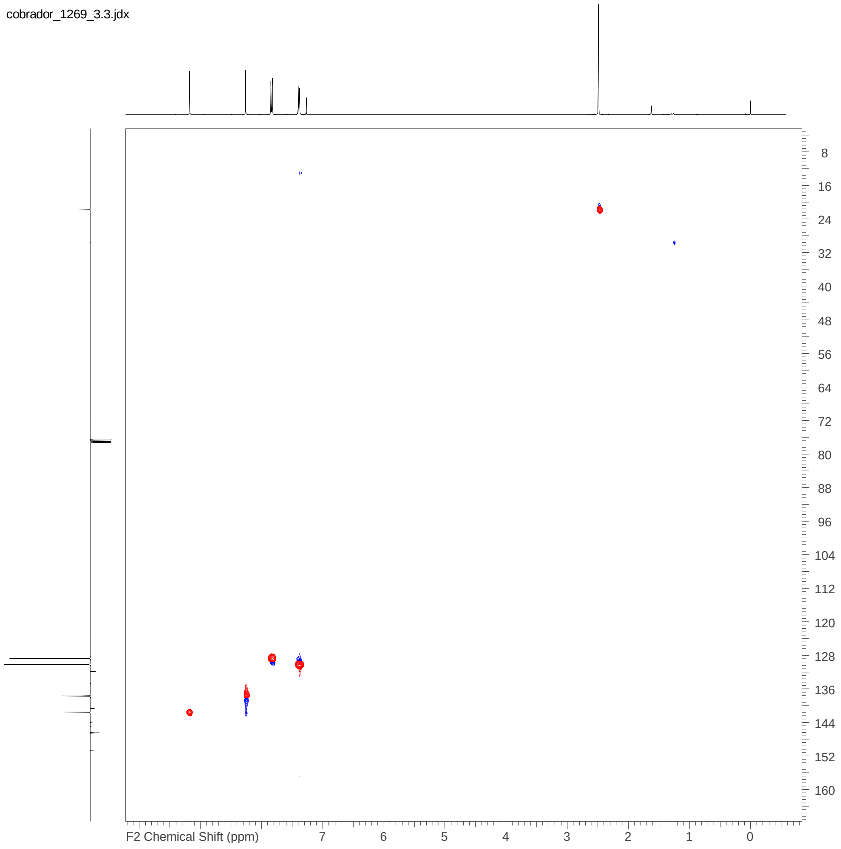

cobrador_1269_3.4.jdx

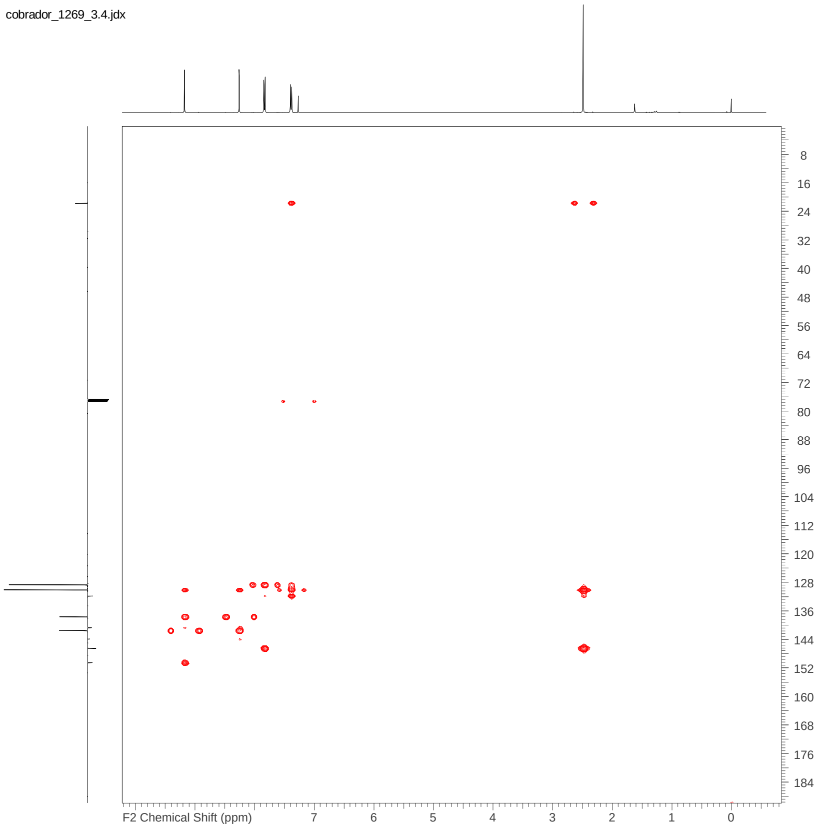

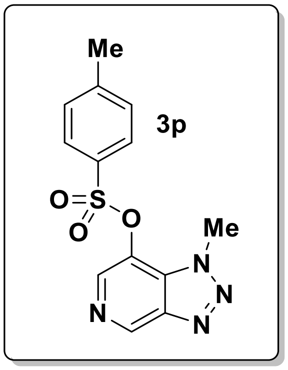

Me
3p
O
S
O
O
Me
N
N
N
N

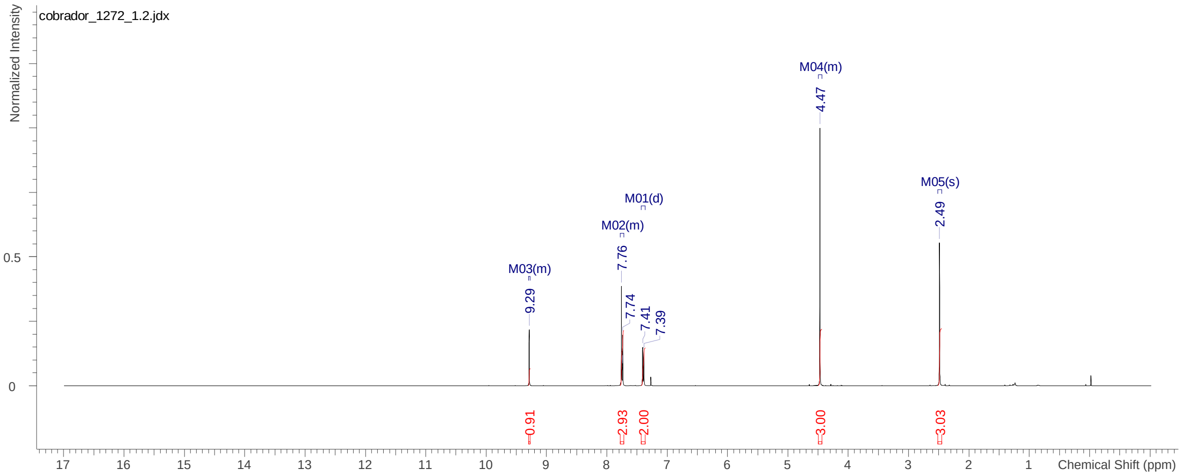

cobrador_1272_1.2.jdx
Normalized Intensity
M04(m)
4.47
M05(s)
2.49
M01(d)
M02(m)
7.76
7.74
7.41
7.39
M03(m)
9.29
0.91
2.93
2.00
3.00
3.03
Chemical Shift (ppm)

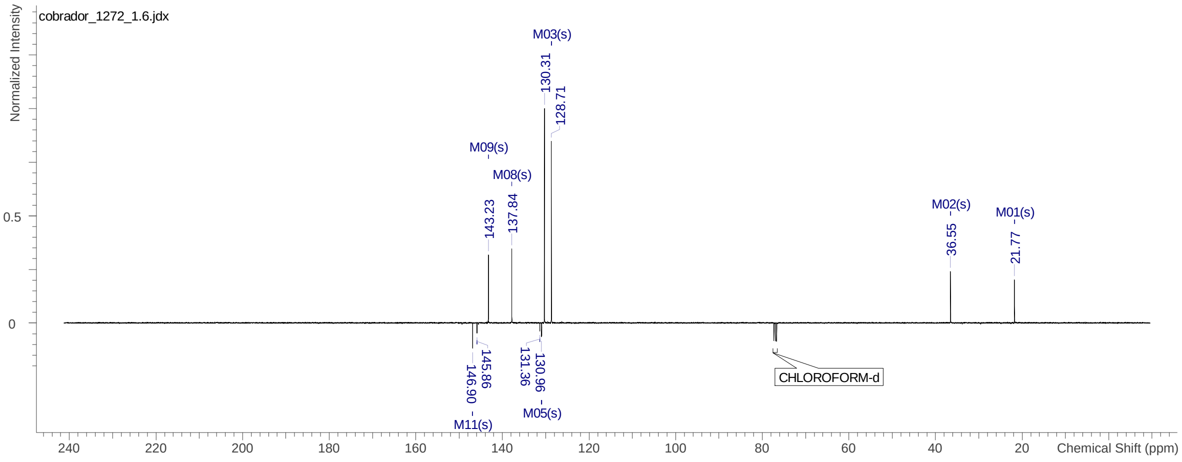

cobrador_1272_1.6.jdx
Normalized Intensity
M03(s)
130.31
128.71
M09(s)
M08(s)
143.23
137.84
M02(s)
36.55
M01(s)
21.77
145.86
146.90
131.36
130.96
M05(s)
M11(s)
CHLOROFORM-d
Chemical Shift (ppm)

cobrador_1272_1.3.jdx

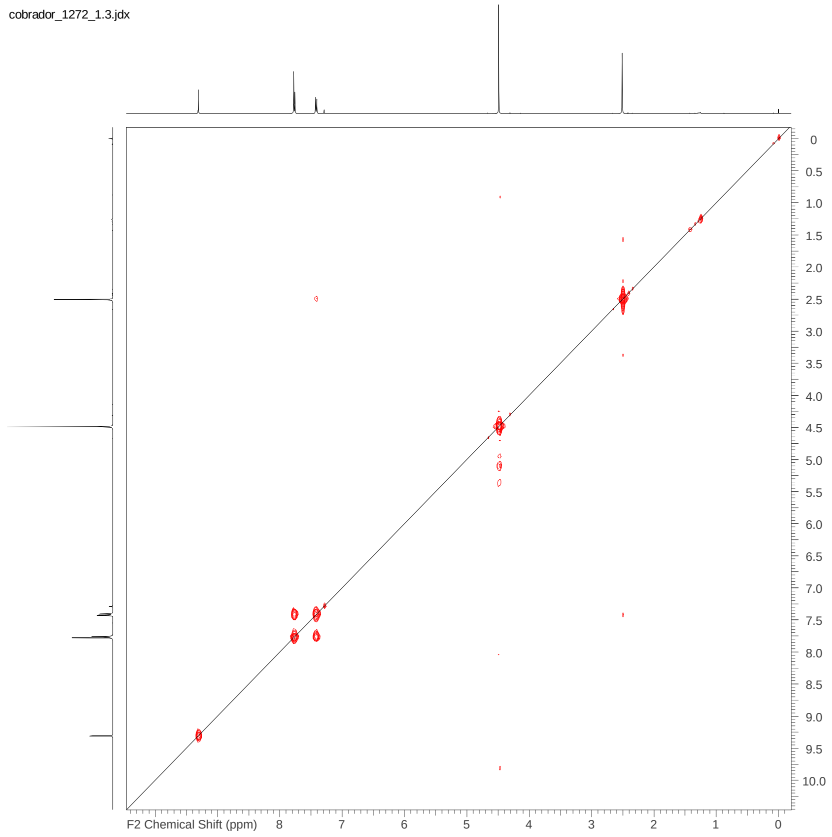

cobrador_1272_1.4.jdx

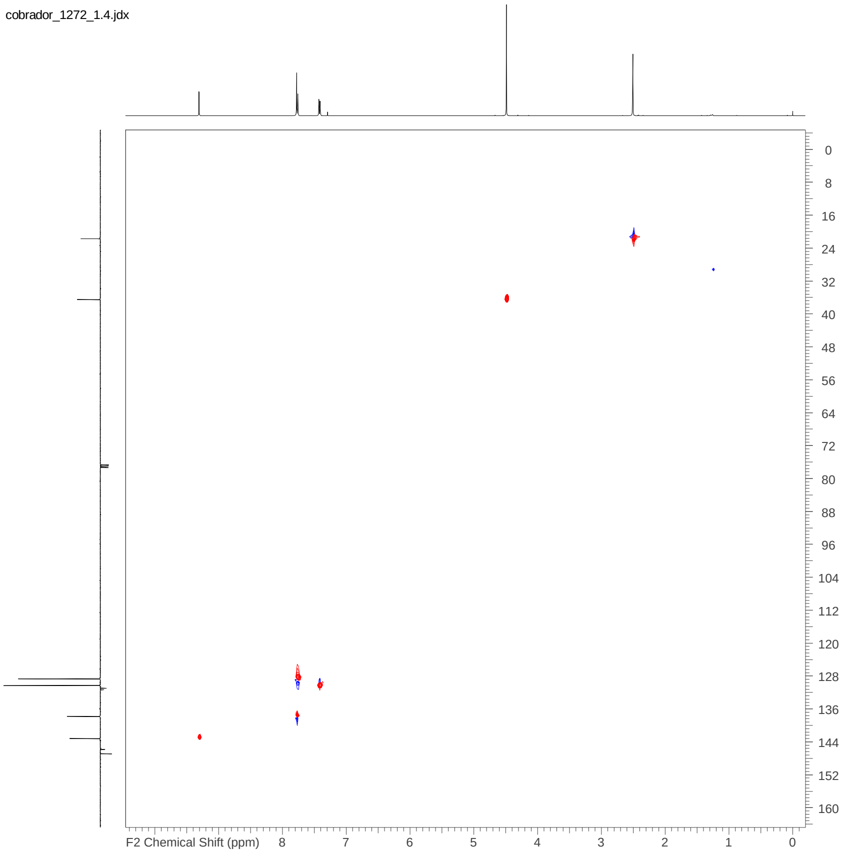

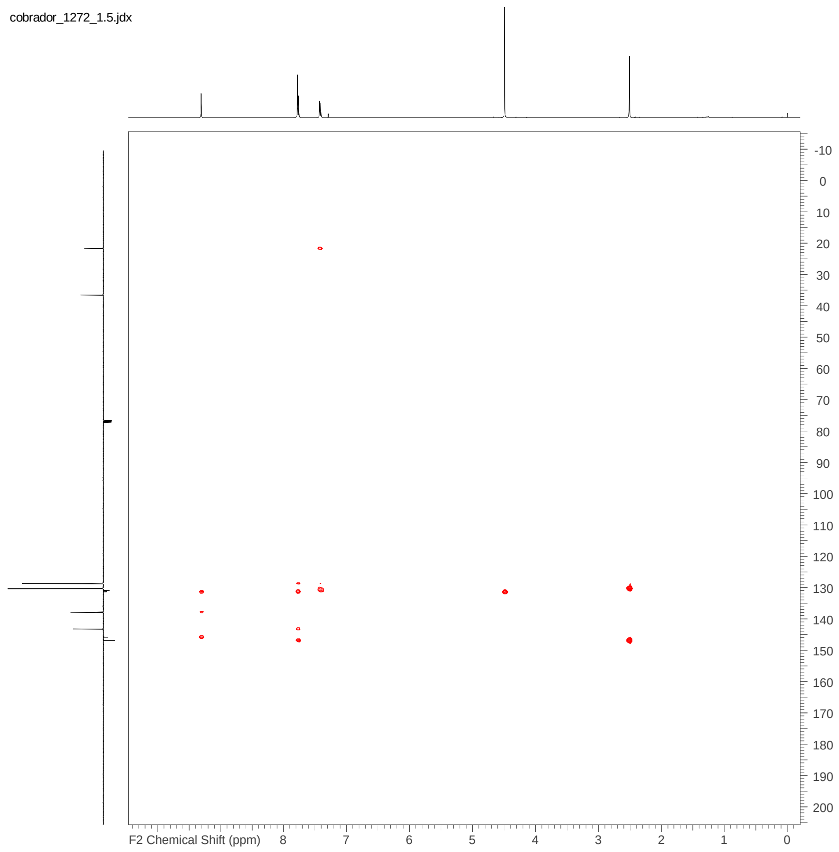
cobrador_1272_1.5.jdx
F2 Chemical Shift (ppm)
8
7
6
5
4
3
2
1
0
-10
0
10
20
30
40
50
60
70
80
90
100
110
120
130
140
150
160
170
180
190
200

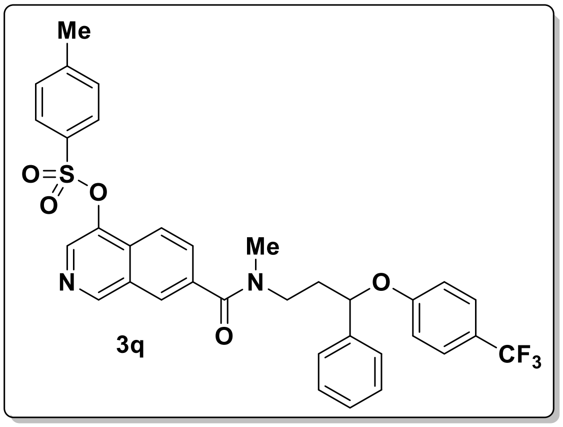

3q

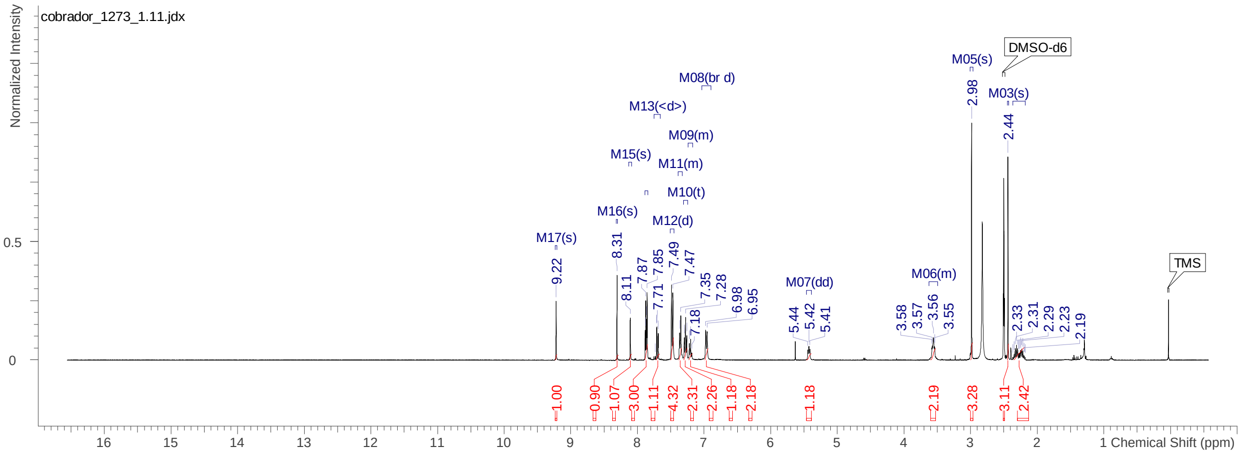
cobrador_1273_1.11.jdx
Normalized Intensity
Chemical Shift (ppm)
DMSO-d6
TMS
M17(s)
M16(s)
M15(s)
M13(<d>)
M12(d)
M11(m)
M10(t)
M09(m)
M08(br d)
M07(dd)
M06(m)
M05(s)
M03(s)
9.22
8.31
8.11
7.87
7.85
7.71
7.49
7.47
7.35
7.28
7.18
6.98
6.95
5.44
5.42
5.41
3.58
3.57
3.56
3.55
2.98
2.44
2.33
2.31
2.29
2.23
2.19
1.00
0.90
1.07
3.00
1.11
4.32
2.31
2.26
1.18
2.18
1.18
2.19
3.28
3.11
2.42

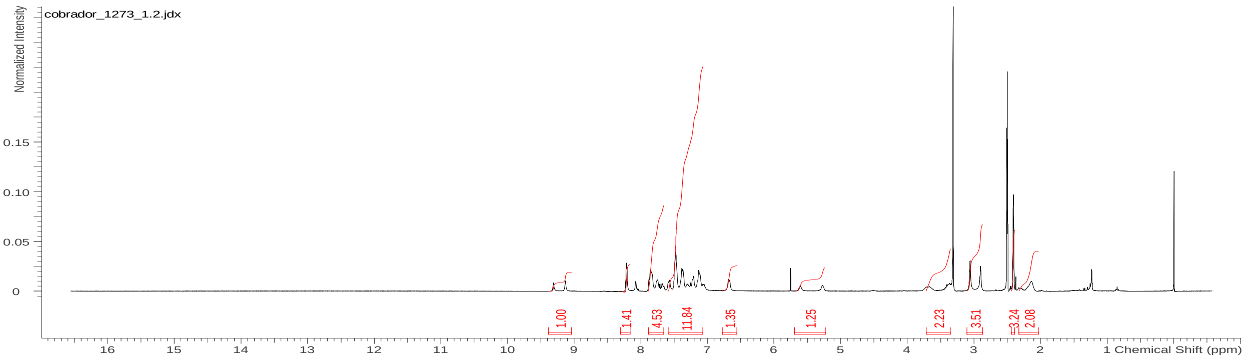
cobrador_1273_1.2.jdx
Normalized Intensity
Chemical Shift (ppm)
1.00
1.41
4.53
11.84
1.35
1.25
2.23
3.51
3.24
2.08

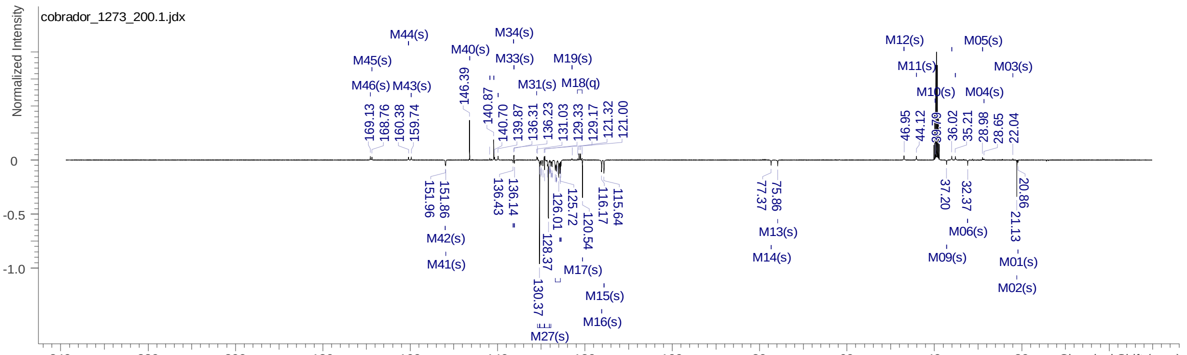

cobrador_1273_200.1.jdx
Normalized Intensity
Chemical Shift (ppm)

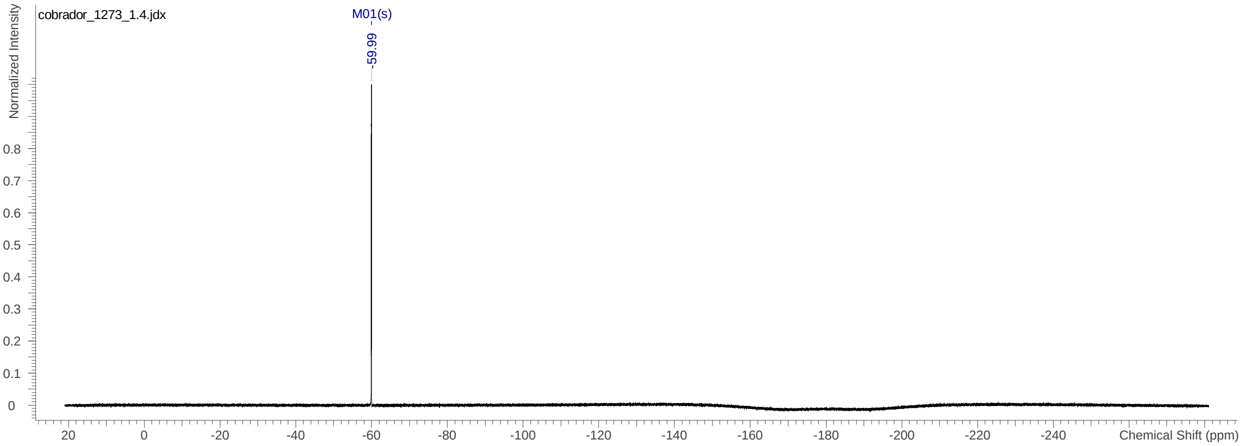

cobrador_1273_1.4.jdx
Normalized Intensity
M01(s)
-59.99
Chemical Shift (ppm)

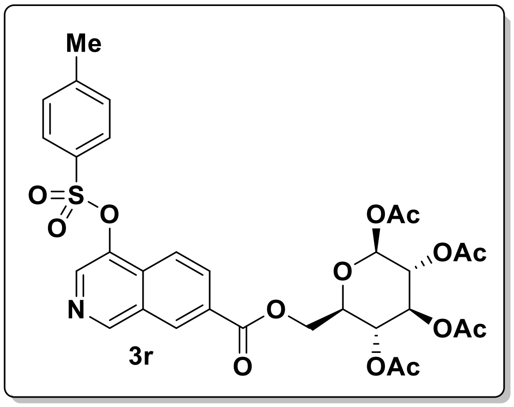
Me
O
N
3r
O
O
OAc
OAc
OAc
OAc

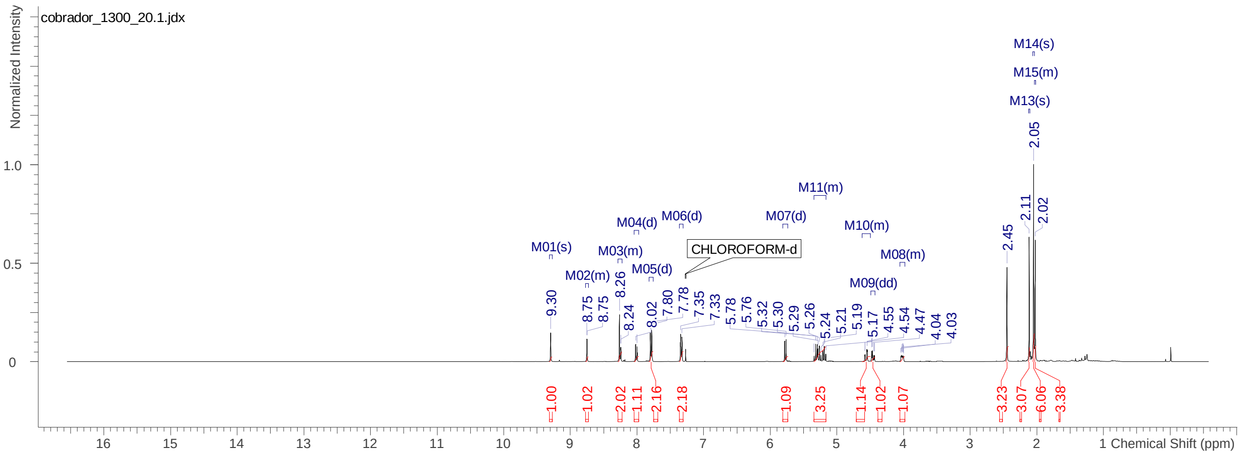
cobrador_1300_20.1.jdx
Normalized Intensity
Chemical Shift (ppm)
CHLOROFORM-d
M01(s)
M02(m)
M03(m)
M04(d)
M05(d)
M06(d)
M07(d)
M08(m)
M09(dd)
M10(m)
M11(m)
M13(s)
M14(s)
M15(m)

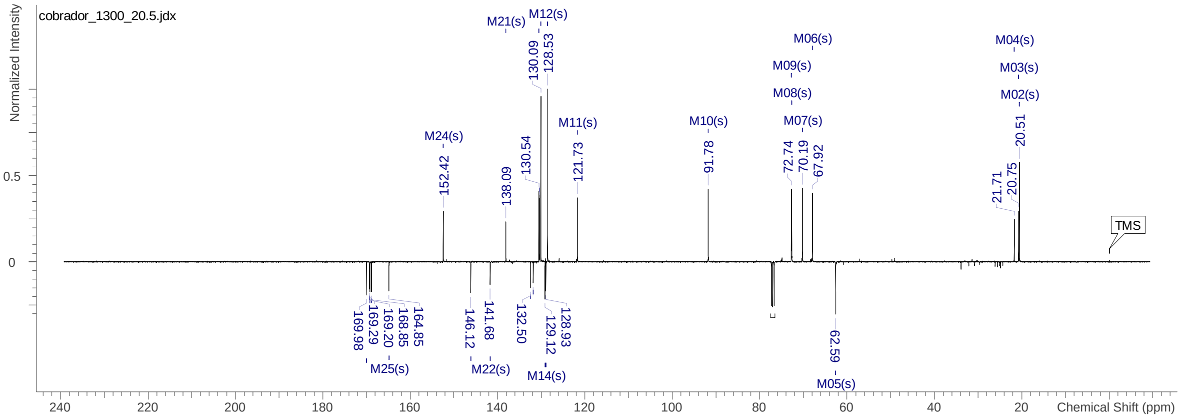
cobrador_1300_20.5.jdx
Normalized Intensity
Chemical Shift (ppm)
TMS

cobrador_1300_20.2.jdx

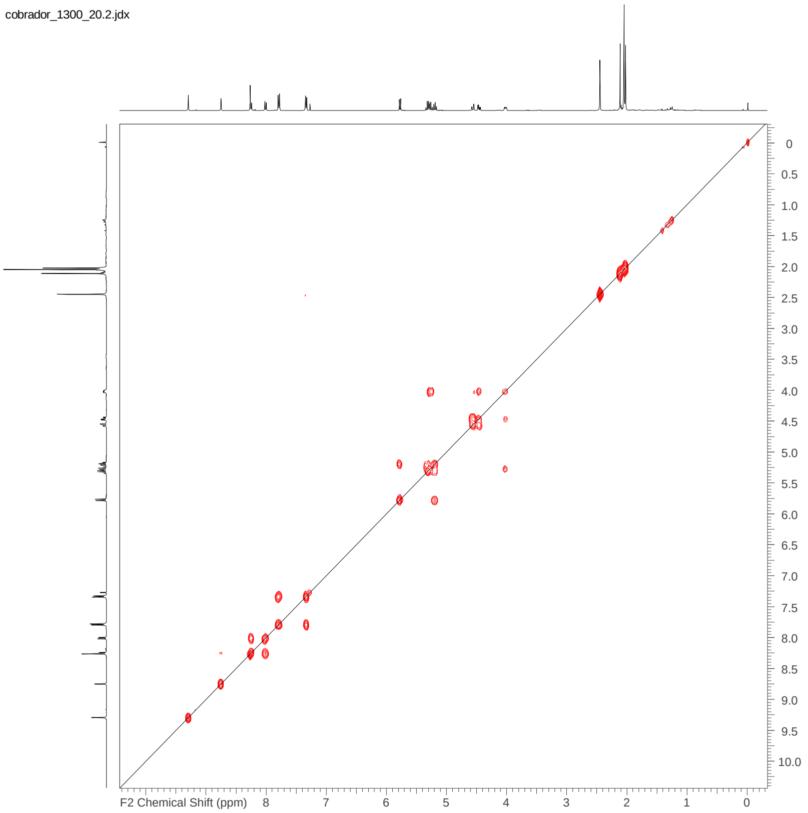

cobrador_1300_20.3.jdx

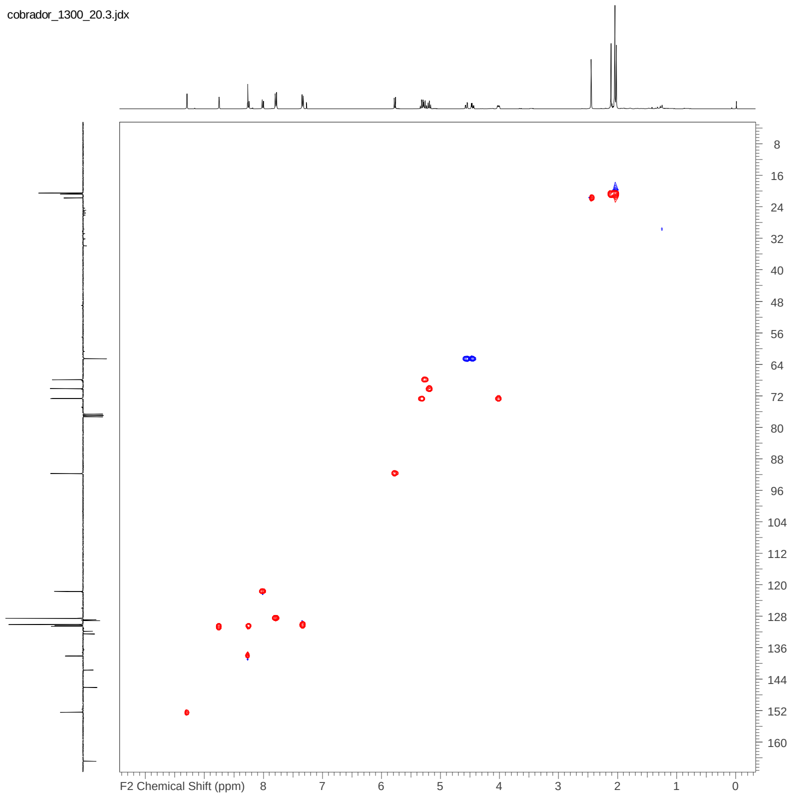

cobrador_1300_20.4.jdx

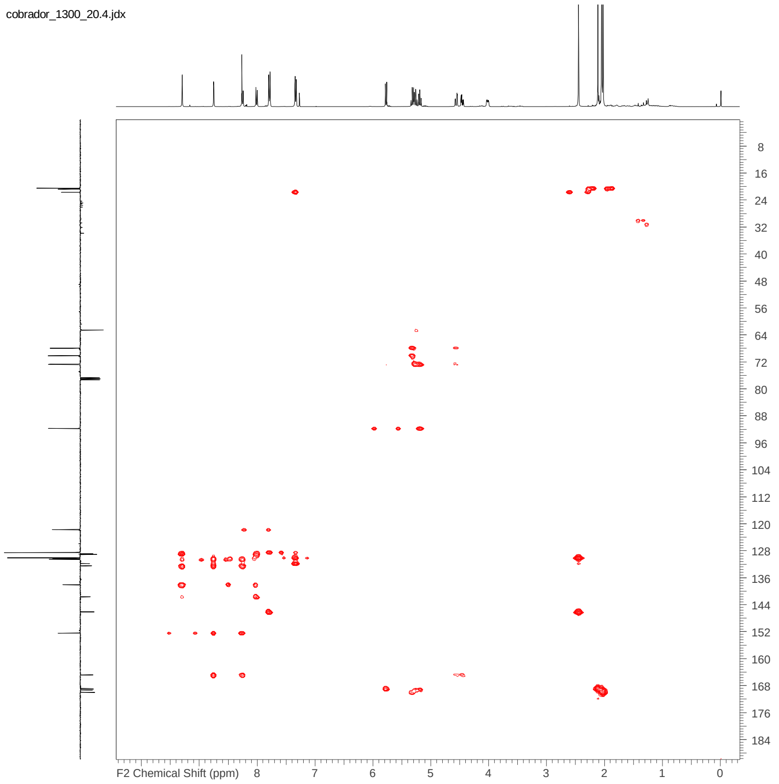

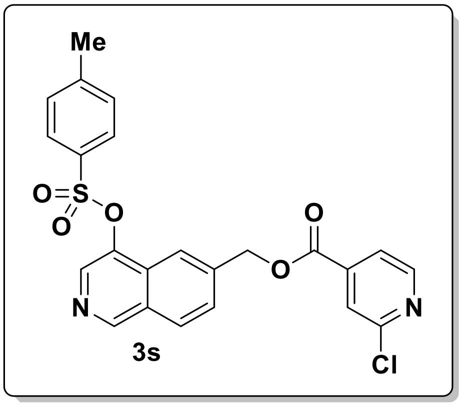
Me
O=S
O
O
O
O
N
N
3s
Cl

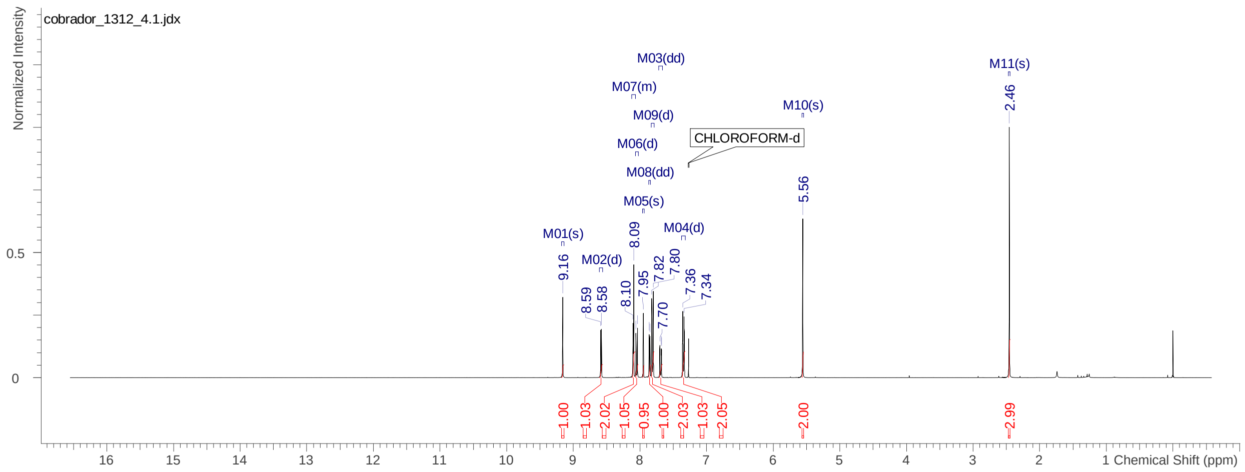
cobrador_1312_4.1.jdx
Normalized Intensity
Chemical Shift (ppm)
CHLOROFORM-d
M01(s) 9.16
M02(d) 8.59 8.58
M03(dd)
M04(d) 7.36 7.34
M05(s)
M06(d)
M07(m)
M08(dd)
M09(d)
M10(s) 5.56
M11(s) 2.46
8.10 8.09 7.95 7.82 7.80 7.70
1.00 1.03 2.02 1.05 0.95 1.00 2.03 1.03 2.05 2.00 2.99

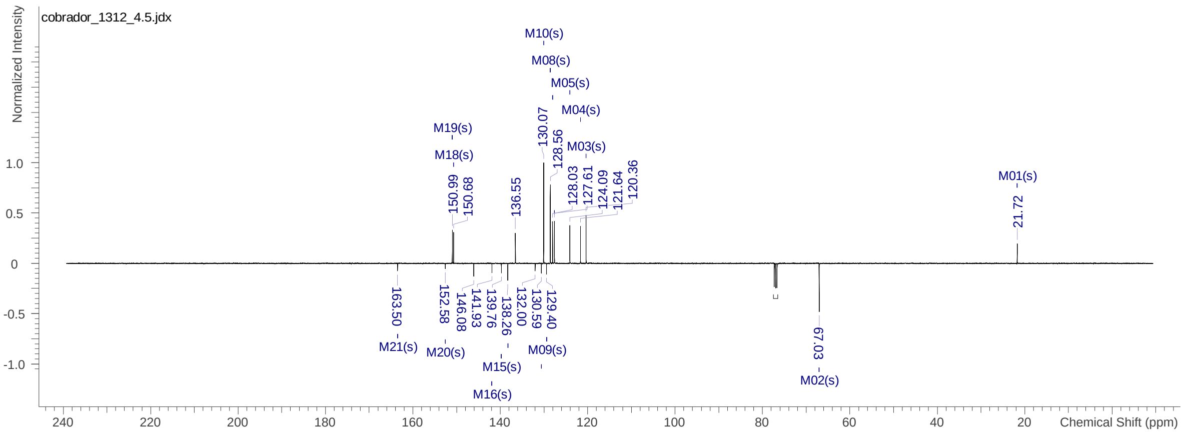
cobrador_1312_4.5.jdx
Normalized Intensity
Chemical Shift (ppm)
M01(s) 21.72
M02(s) 67.03
M03(s) M04(s) M05(s) M08(s) M09(s) M10(s)
M15(s) M16(s) M18(s) M19(s) M20(s) M21(s)
163.50 152.58 150.99 150.68 146.08 141.93 139.76 138.26 136.55 132.00 130.59 130.07 129.40 128.56 128.03 127.61 124.09 121.64 120.36

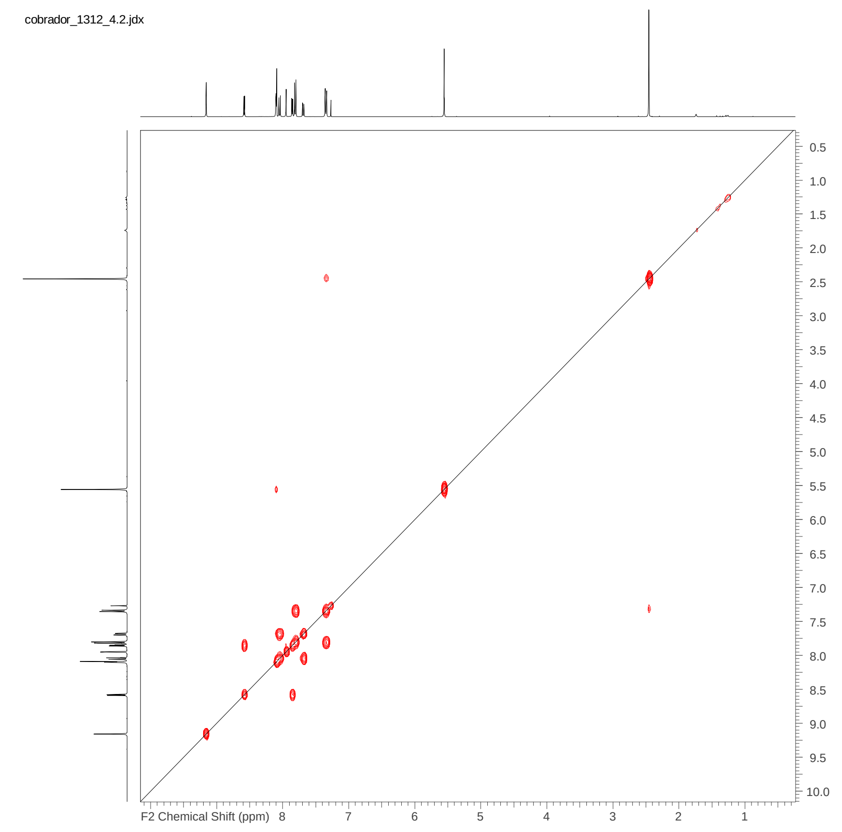
cobrador_1312_4.2.jdx
F2 Chemical Shift (ppm)

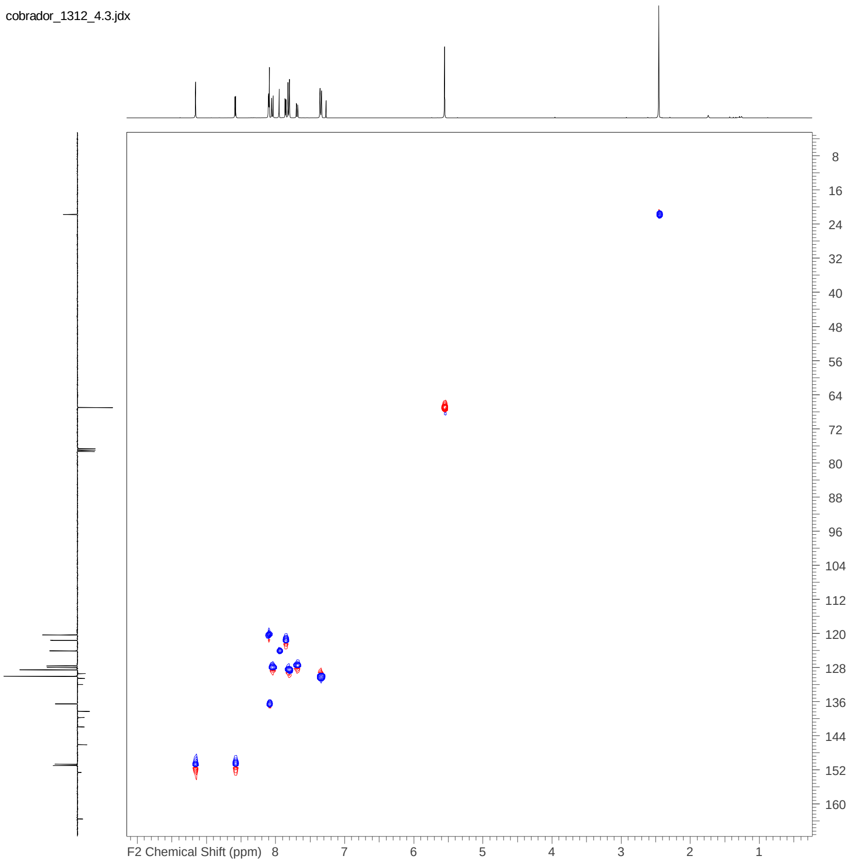
cobrador_1312_4.3.jdx
F2 Chemical Shift (ppm)

cobrador_1312_4.4.jdx

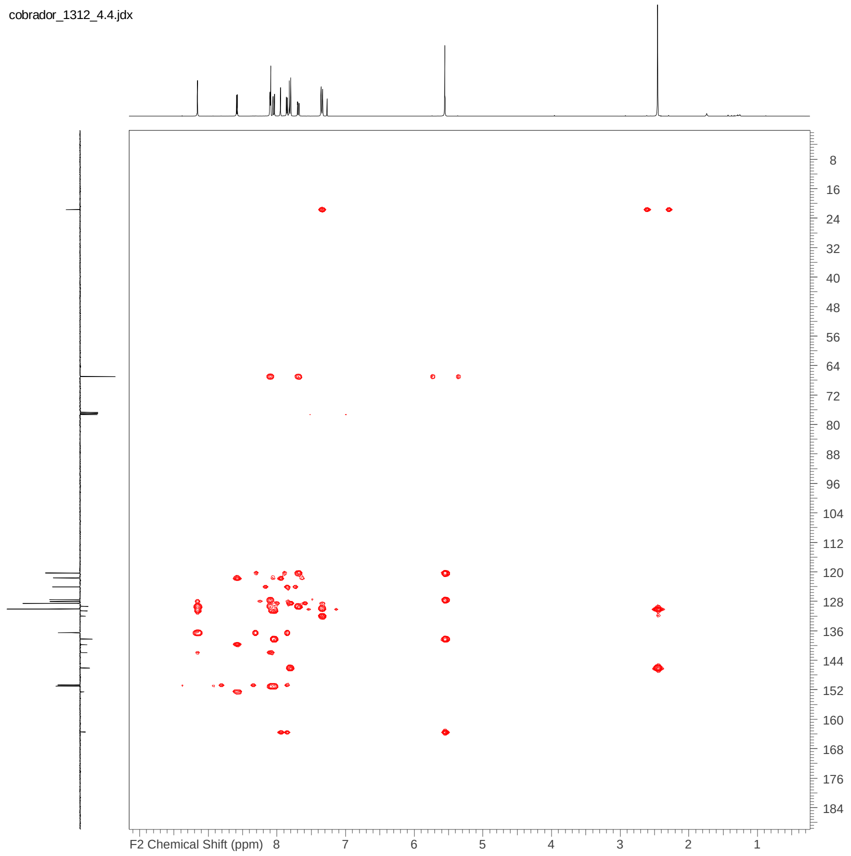

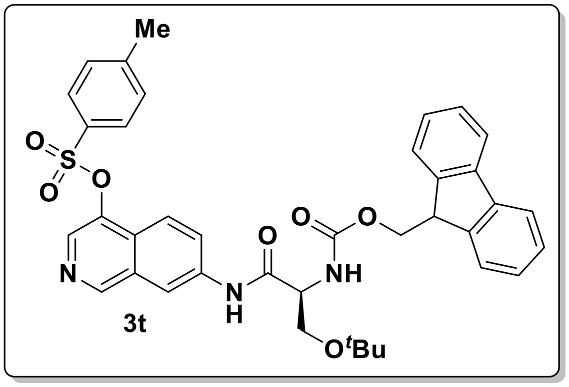
Me
O
S
O
O
N
N
H
O
NH
O
O
O^{t}Bu

3t

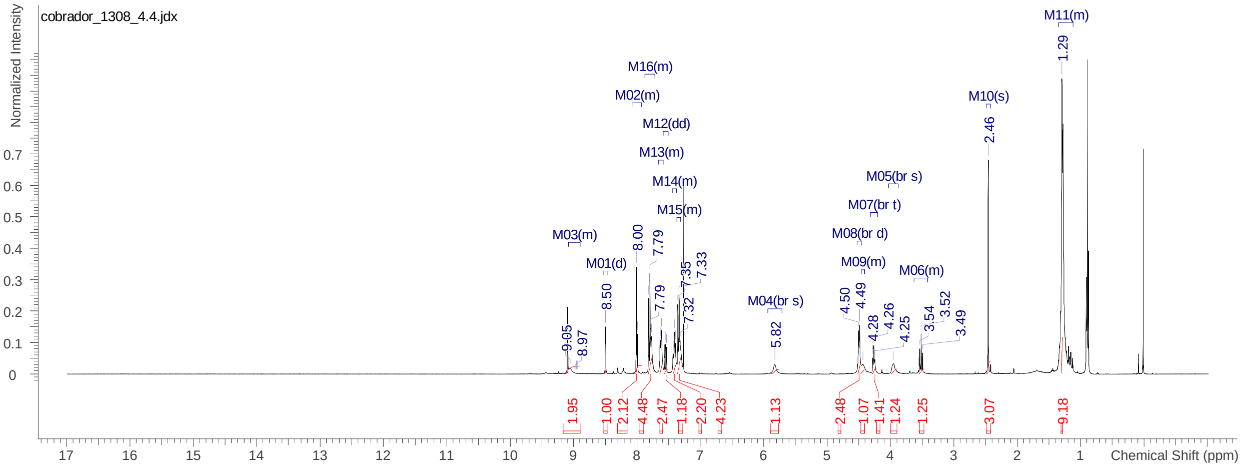
cobrador_1308_4.4.jdx
Normalized Intensity
Chemical Shift (ppm)
M01(d)
M02(m)
M03(m)
M04(br s)
M05(br s)
M06(m)
M07(br t)
M08(br d)
M09(m)
M10(s)
M11(m)
M12(dd)
M13(m)
M14(m)
M15(m)
M16(m)
9.05
8.97
8.50
8.00
7.79
7.79
7.35
7.33
7.32
5.82
4.50
4.49
4.28
4.26
4.25
3.54
3.52
3.49
2.46
1.29
1.95
1.00
2.12
4.48
2.47
1.18
2.20
4.23
1.13
2.48
1.07
1.41
1.24
1.25
3.07
9.18

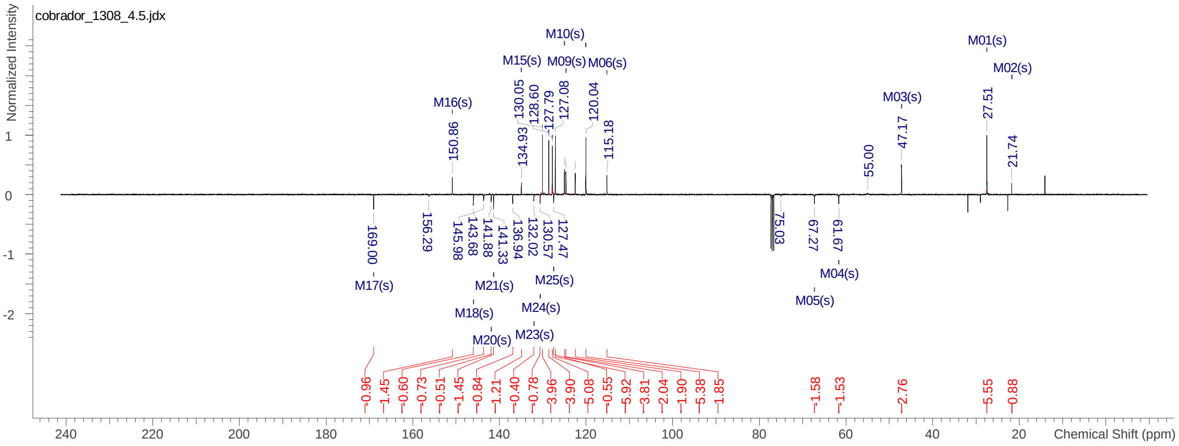
cobrador_1308_4.5.jdx
Normalized Intensity
Chemical Shift (ppm)
M01(s)
M02(s)
M03(s)
M04(s)
M05(s)
M06(s)
M09(s)
M10(s)
M15(s)
M16(s)
M17(s)
M18(s)
M20(s)
M21(s)
M23(s)
M24(s)
M25(s)
150.86
134.93
130.05
128.60
127.79
127.08
120.04
115.18
55.00
47.17
27.51
21.74
169.00
156.29
145.98
143.68
141.88
141.33
136.94
132.02
130.57
127.47
75.03
67.27
61.67

cobrador_1308_4.2.jdx

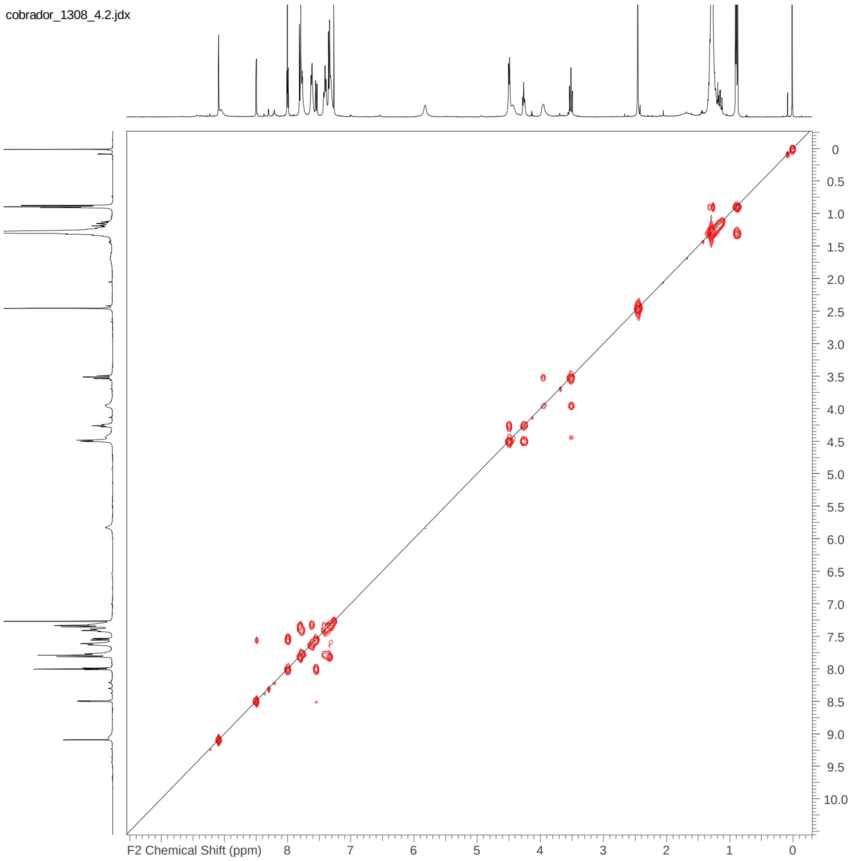

cobrador_1308_4.3.jdx

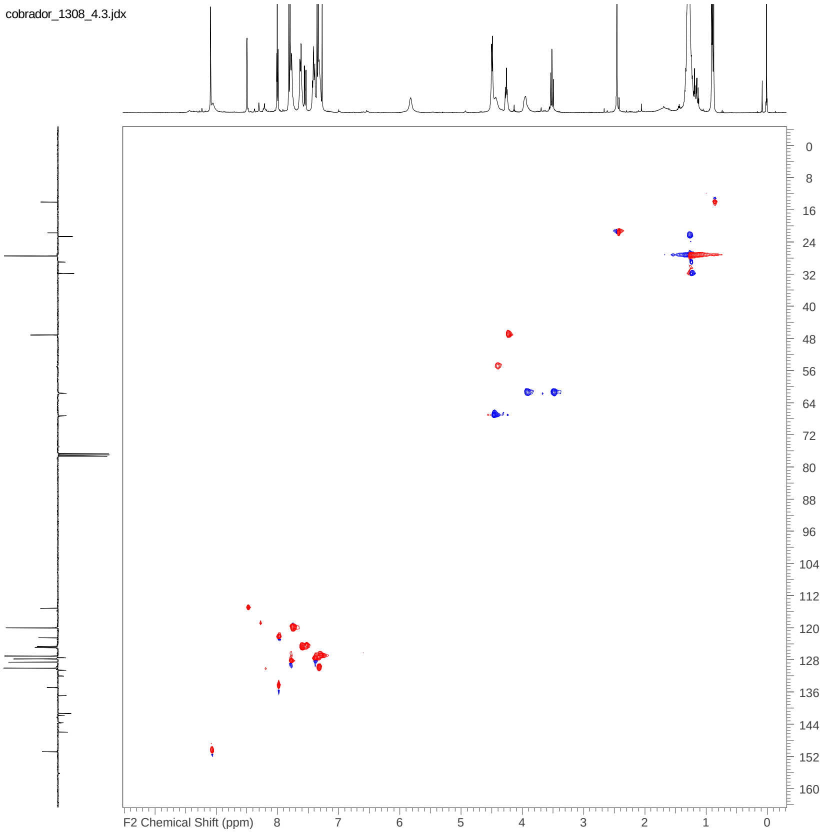

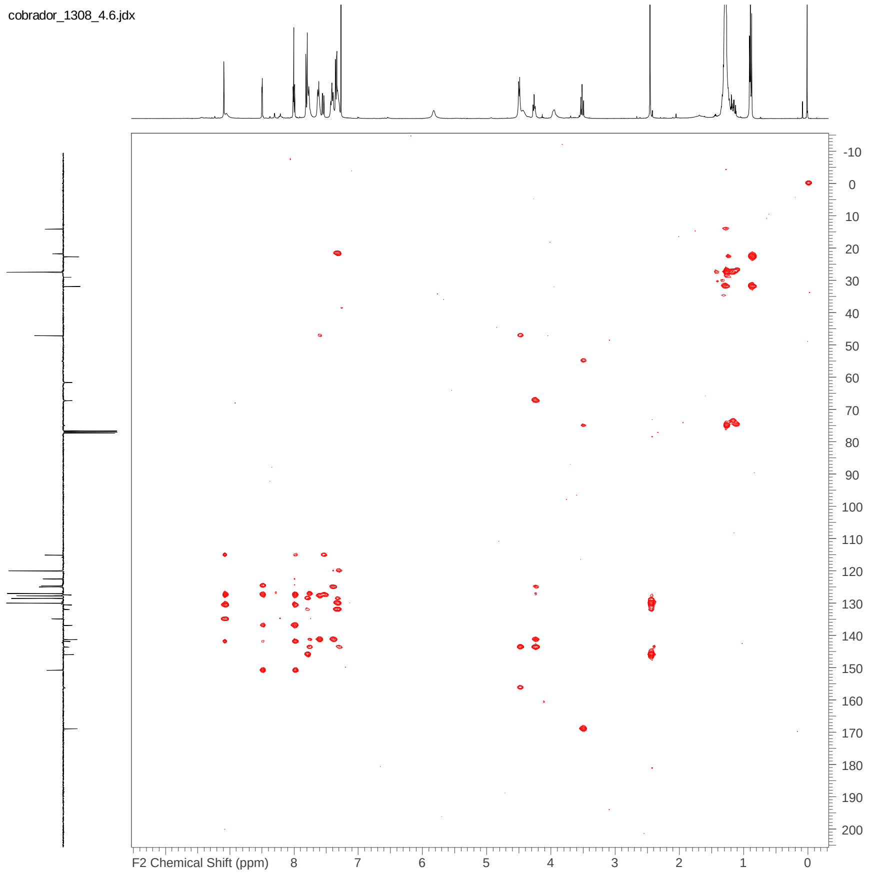
cobrador_1308_4.6.jdx
F2 Chemical Shift (ppm)

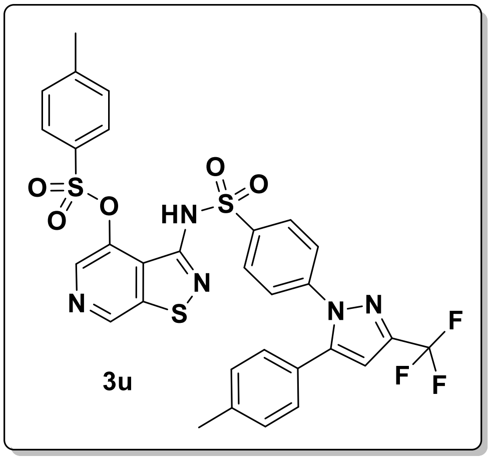
3u

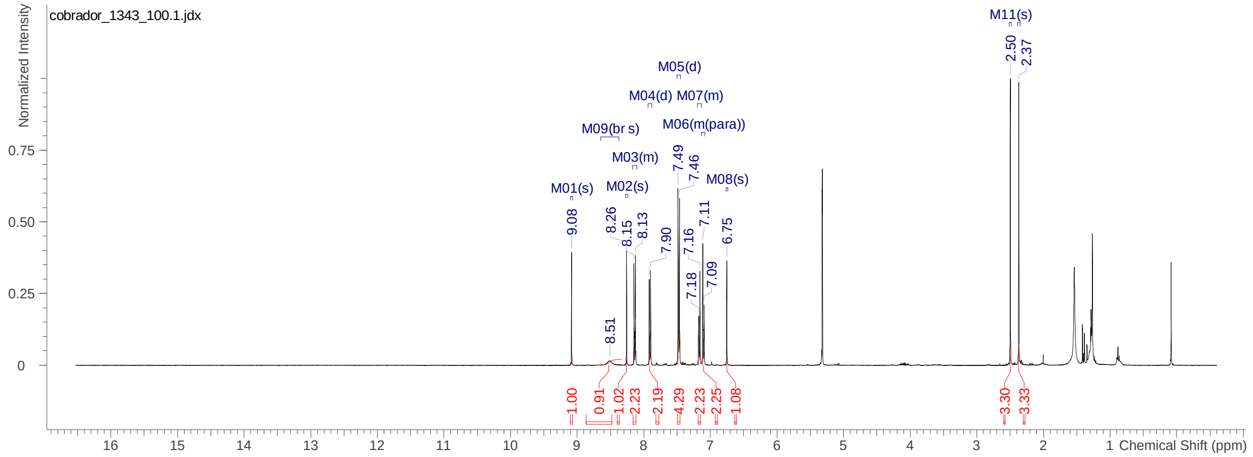
cobrador_1343_100.1.jdx
Normalized Intensity
Chemical Shift (ppm)
M01(s)
M02(s)
M03(m)
M04(d)
M05(d)
M06(m(para))
M07(m)
M08(s)
M09(br s)
M11(s)
9.08
8.51
8.26
8.15
8.13
7.90
7.49
7.46
7.18
7.16
7.11
7.09
6.75
2.50
2.37
1.00
0.91
1.02
2.23
2.19
4.29
2.23
2.25
1.08
3.30
3.33

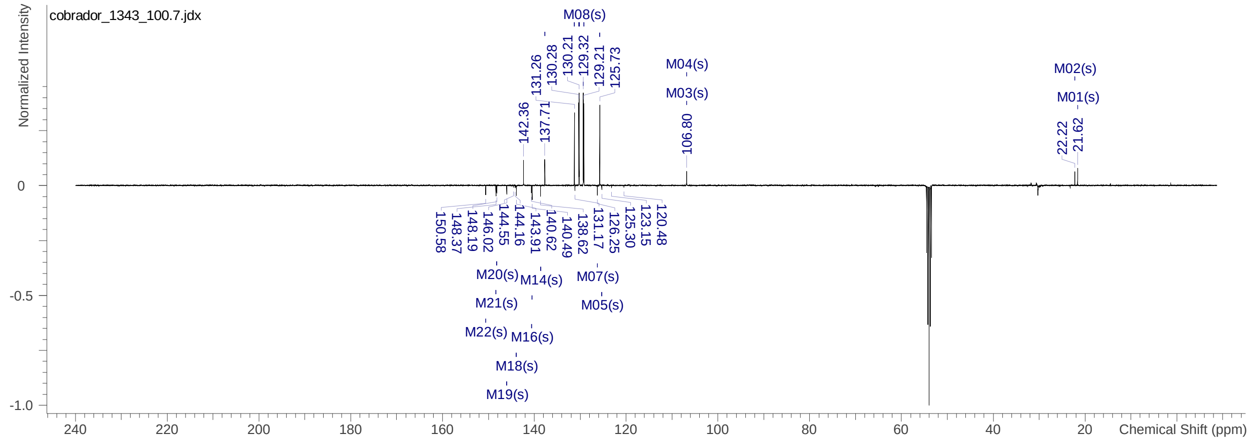
cobrador_1343_100.7.jdx
Normalized Intensity
Chemical Shift (ppm)
142.36
137.71
131.26
130.28
130.21
129.32
129.21
125.73
106.80
22.22
21.62
150.58
148.37
148.19
146.02
144.55
144.16
143.91
140.62
140.49
138.62
131.17
126.25
125.30
123.15
120.48
M08(s)
M04(s)
M03(s)
M02(s)
M01(s)
M20(s)
M14(s)
M07(s)
M21(s)
M05(s)
M22(s)
M16(s)
M18(s)
M19(s)

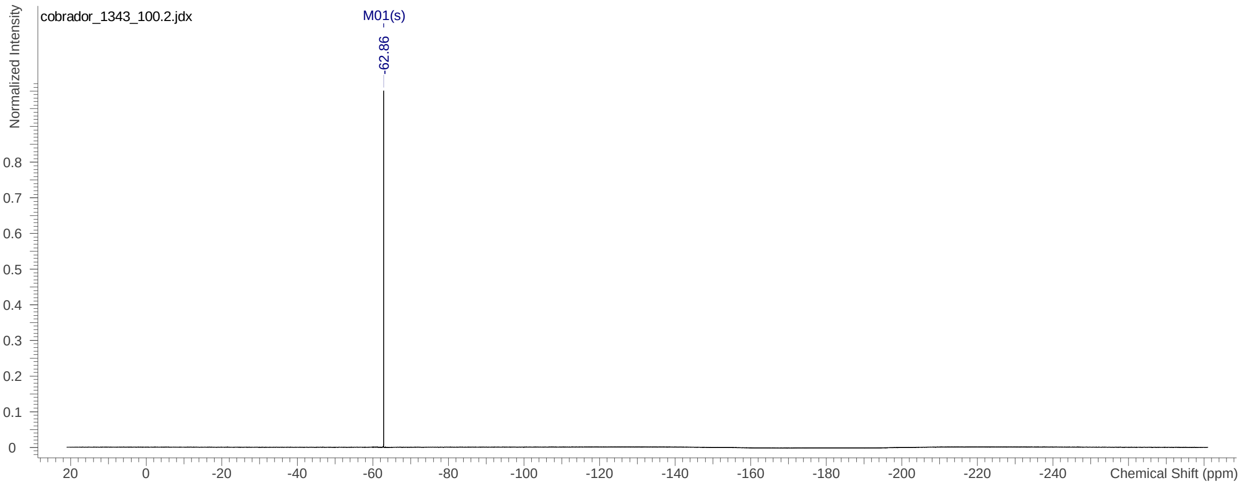
cobrador_1343_100.2.jdx
M01(s)
-62.86
Normalized Intensity
0.8
0.7
0.6
0.5
0.4
0.3
0.2
0.1
0
20
0
-20
-40
-60
-80
-100
-120
-140
-160
-180
-200
-220
-240
Chemical Shift (ppm)

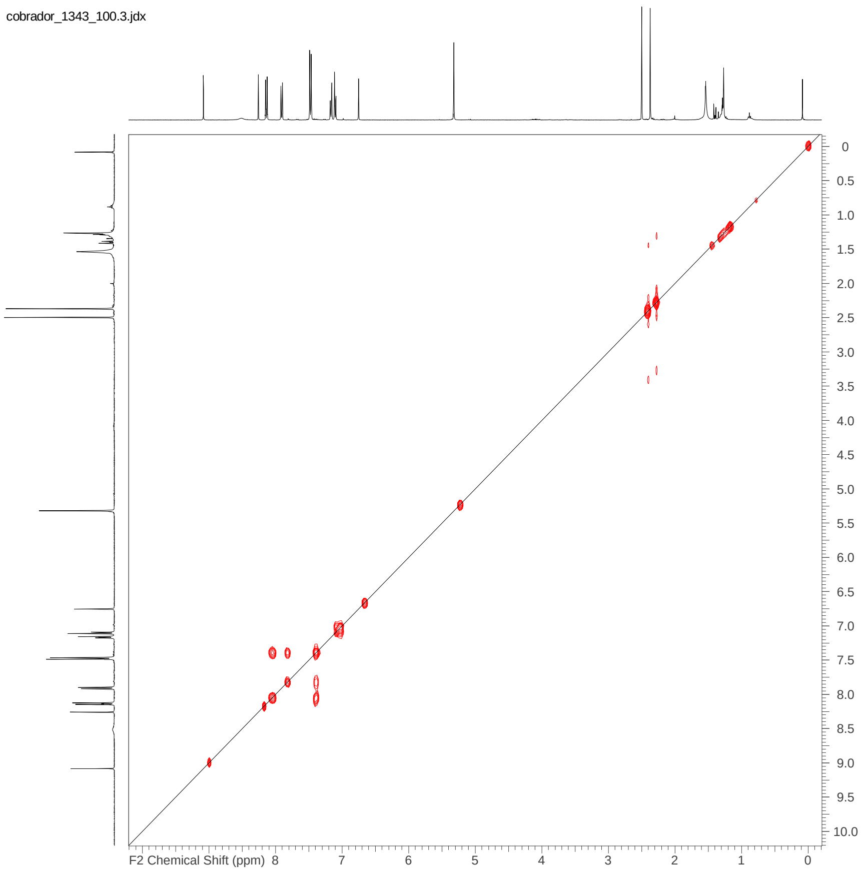
cobrador_1343_100.3.jdx
F2 Chemical Shift (ppm)
8
7
6
5
4
3
2
1
0
0.5
1.0
1.5
2.0
2.5
3.0
3.5
4.0
4.5
5.0
5.5
6.0
6.5
7.0
7.5
8.0
8.5
9.0
9.5
10.0

cobrador_1343_100.4.jdx

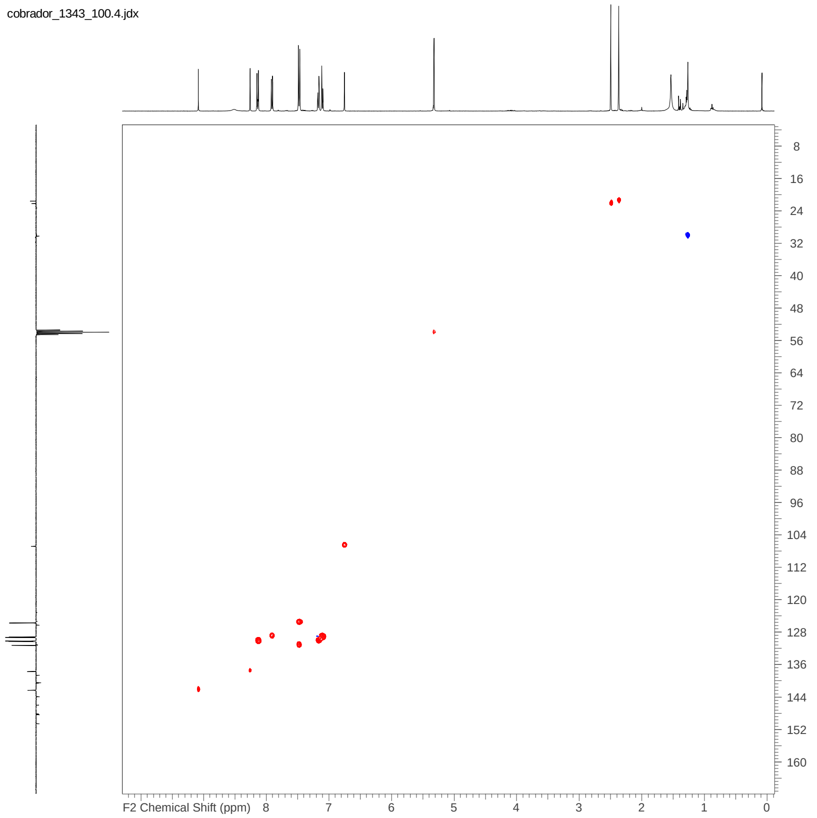

cobrador_1343_100.5.jdx

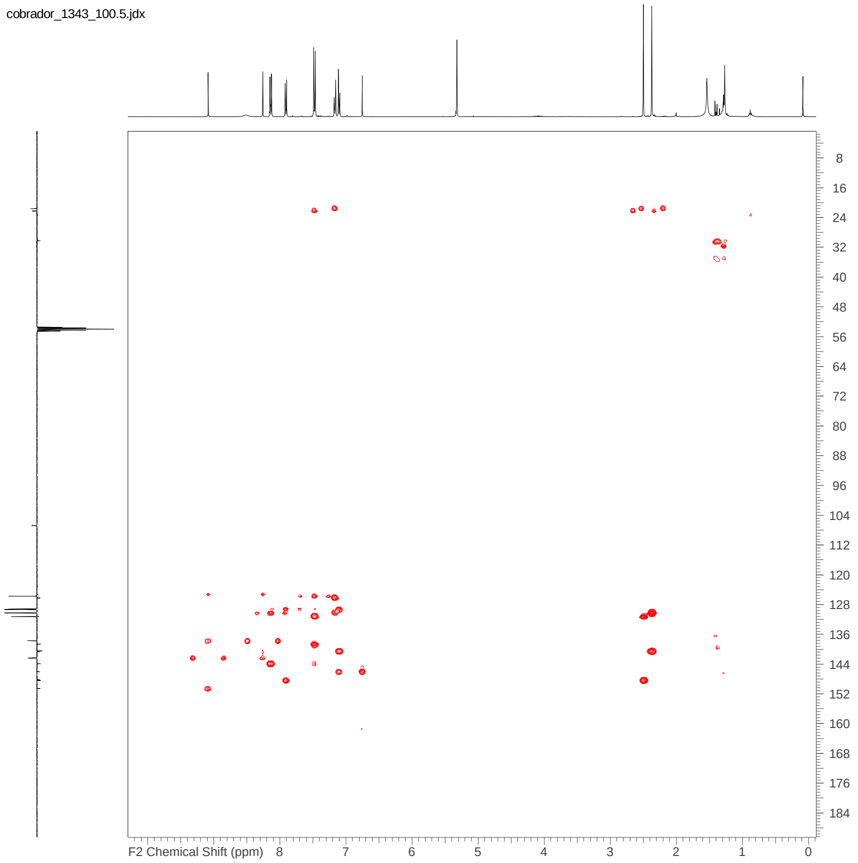

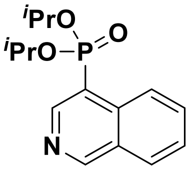

^{i}PrO
^{i}PrO
P
O
N

8

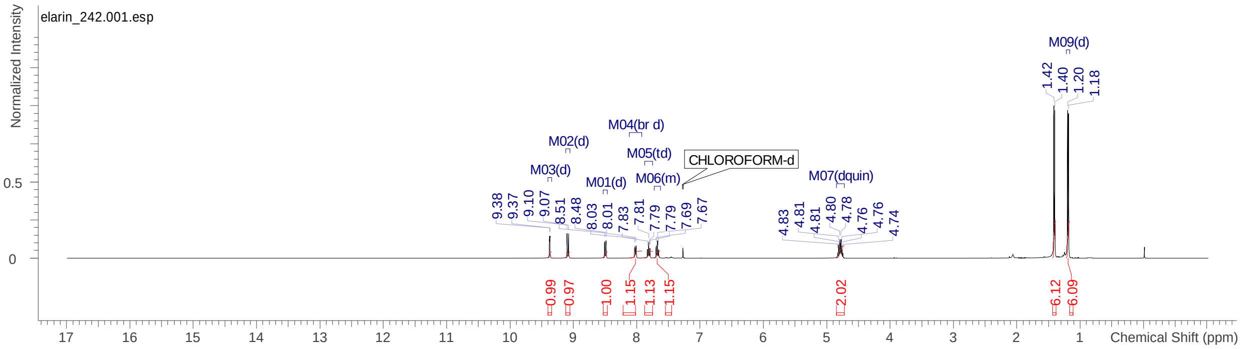

elarin_242.001.esp
Normalized Intensity
M09(d)
M04(br d)
M02(d)
M05(td)
CHLOROFORM-d
M03(d)
M01(d)
M06(m)
M07(dquin)
9.38 9.37 9.10 9.07 8.51 8.48 8.03 8.01 7.83 7.81 7.79 7.79 7.69 7.67
4.83 4.81 4.81 4.80 4.78 4.76 4.76 4.74
1.42 1.40 1.20 1.18
0.99 0.97 1.00 1.15 1.13 1.15 2.02 6.12 6.09
Chemical Shift (ppm)

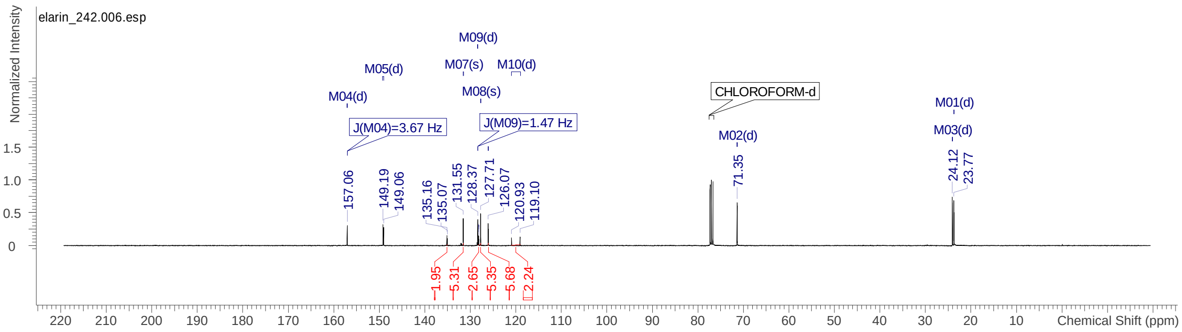

elarin_242.006.esp
Normalized Intensity
M09(d)
M05(d)
M07(s)
M10(d)
M08(s)
M04(d)
CHLOROFORM-d
M01(d)
J(M04)=3.67 Hz
J(M09)=1.47 Hz
M03(d)
M02(d)
157.06 149.19 149.06 135.16 135.07 131.55 128.37 127.71 126.07 120.93 119.10
71.35
24.12 23.77
1.95 5.31 2.65 5.35 5.68 2.24
Chemical Shift (ppm)

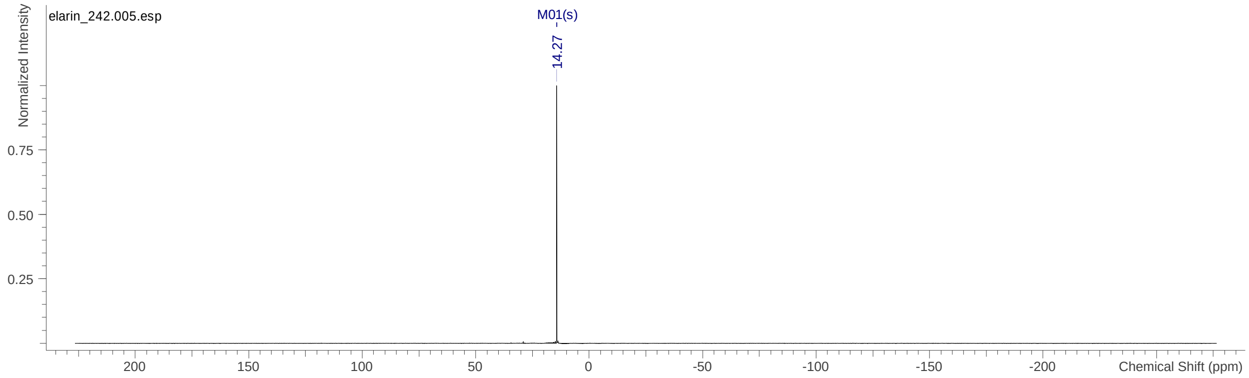

elarin_242.005.esp
Normalized Intensity
M01(s)
14.27
Chemical Shift (ppm)

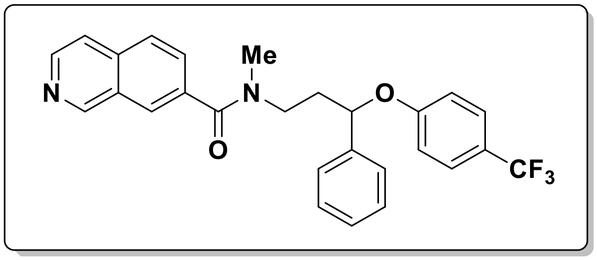
N
Me
N
O
O
CF3

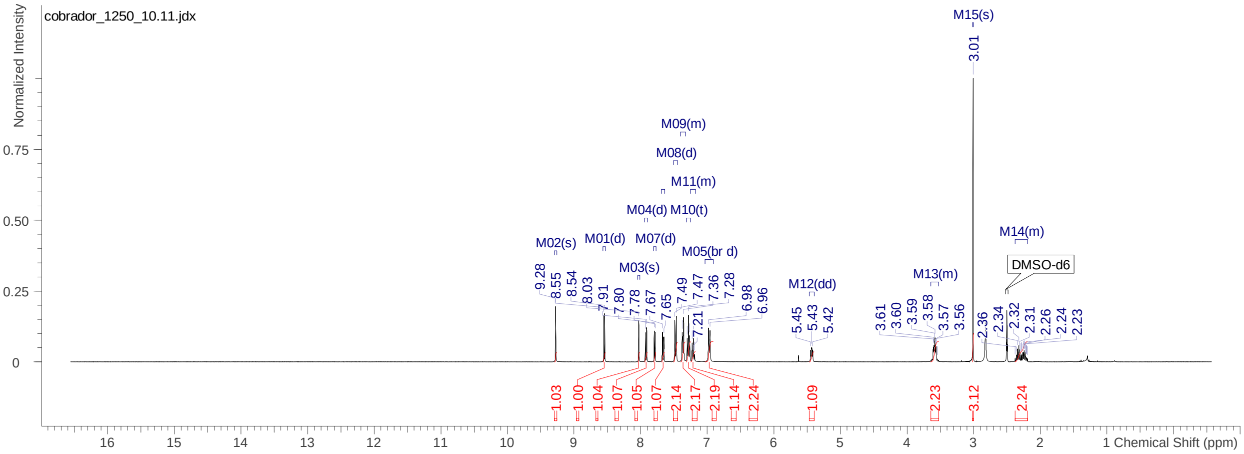
cobrador_1250_10.11.jdx
Normalized Intensity
M15(s)
M09(m)
M08(d)
M11(m)
M04(d) M10(t)
M02(s) M01(d) M07(d)
M05(br d)
M03(s)
M12(dd)
M13(m)
M14(m)
DMSO-d6
Chemical Shift (ppm)

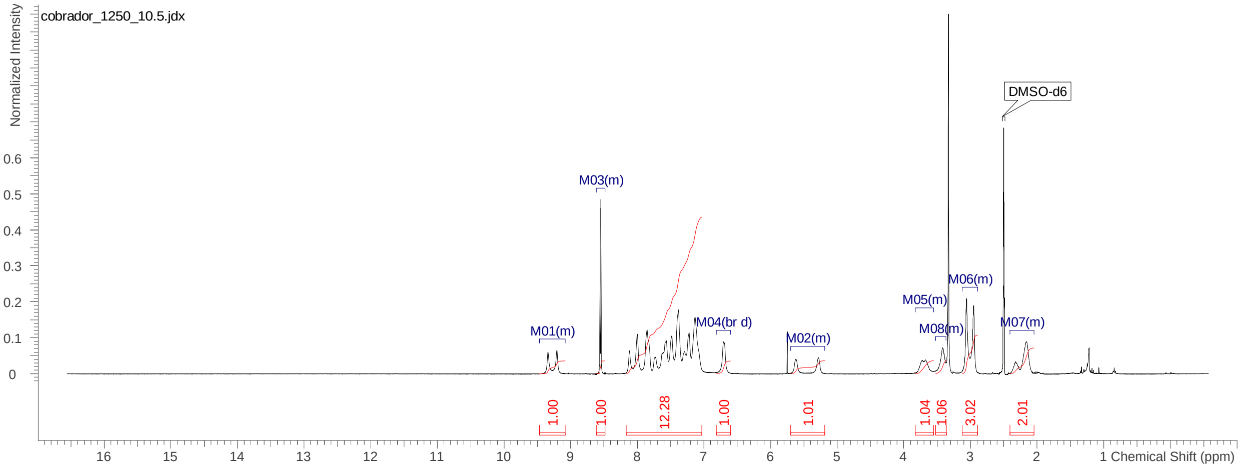
cobrador_1250_10.5.jdx
Normalized Intensity
DMSO-d6
M03(m)
M06(m)
M05(m)
M04(br d)
M08(m)
M07(m)
M01(m)
M02(m)
Chemical Shift (ppm)

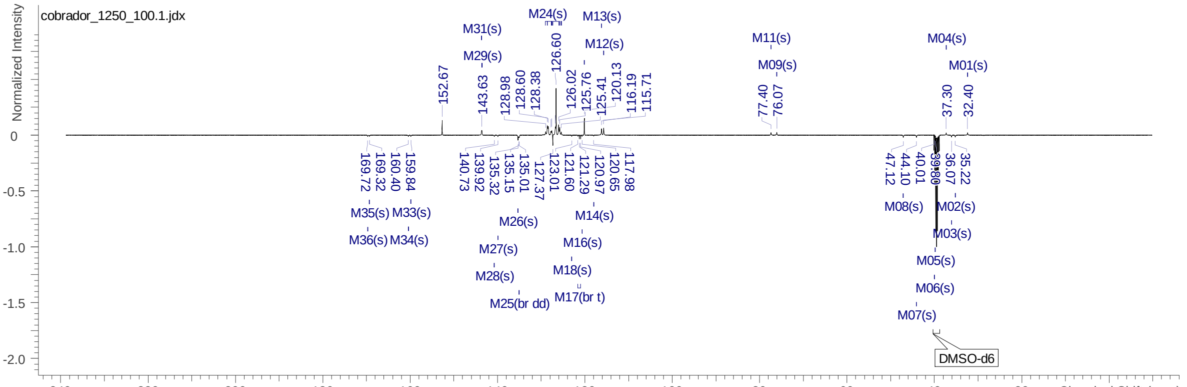
cobrador_1250_100.1.jdx
Normalized Intensity
Chemical Shift (ppm)
DMSO-d6

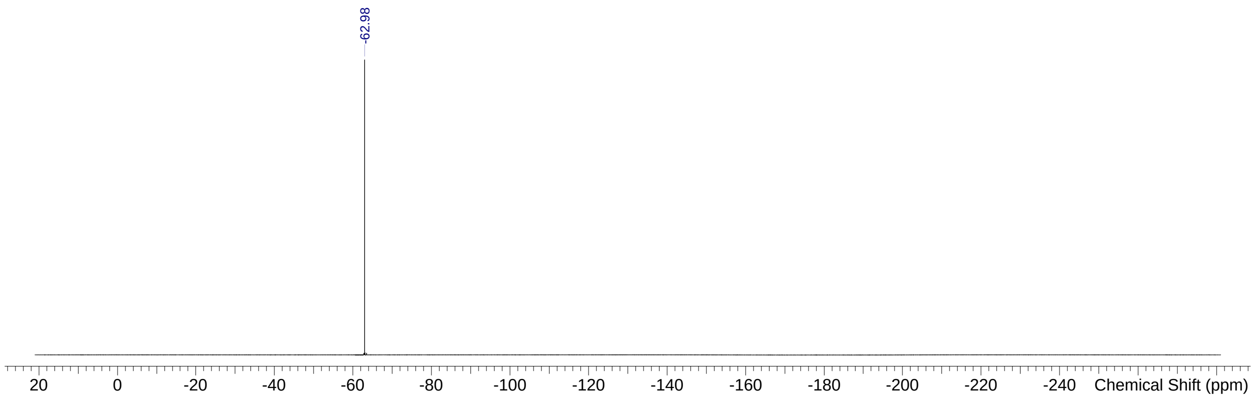
-62.98
Chemical Shift (ppm)

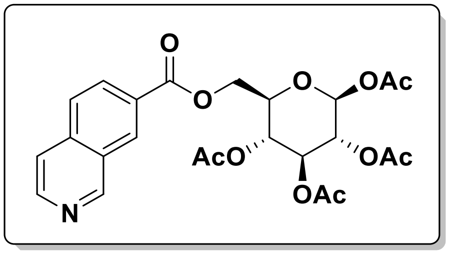
O
O
O
OAc
AcO
OAc
OAc
N

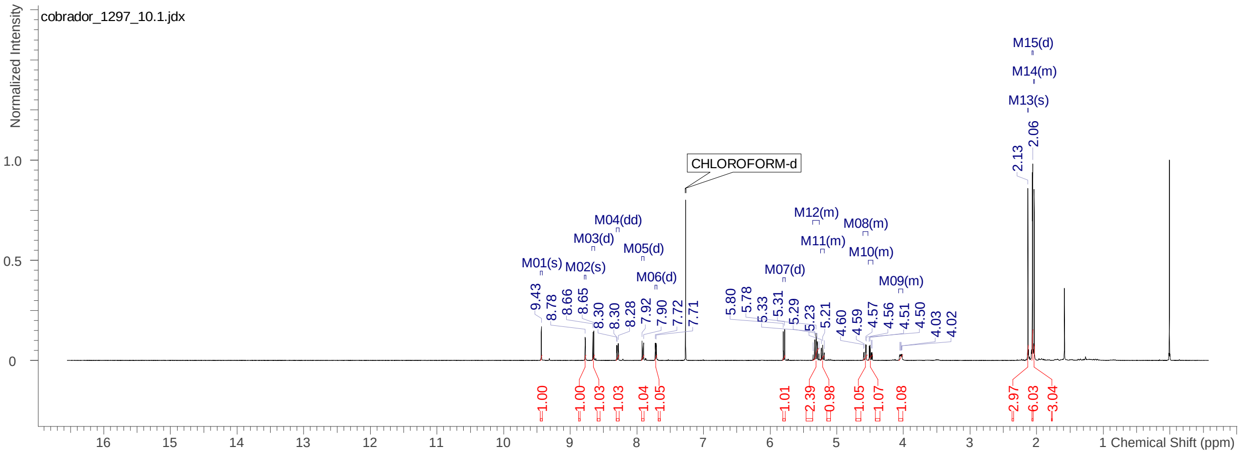
Normalized Intensity
cobrador_1297_10.1.jdx
CHLOROFORM-d
M01(s)
M02(s)
M03(d)
M04(dd)
M05(d)
M06(d)
M07(d)
M08(m)
M09(m)
M10(m)
M11(m)
M12(m)
M13(s)
M14(m)
M15(d)
9.43
8.78
8.66
8.65
8.30
8.30
8.28
7.92
7.90
7.72
7.71
5.80
5.78
5.33
5.31
5.29
5.23
5.21
4.60
4.59
4.57
4.56
4.51
4.50
4.03
4.02
2.13
2.06
1.00
1.00
1.03
1.03
1.04
1.05
1.01
2.39
0.98
1.05
1.07
1.08
2.97
6.03
3.04
Chemical Shift (ppm)

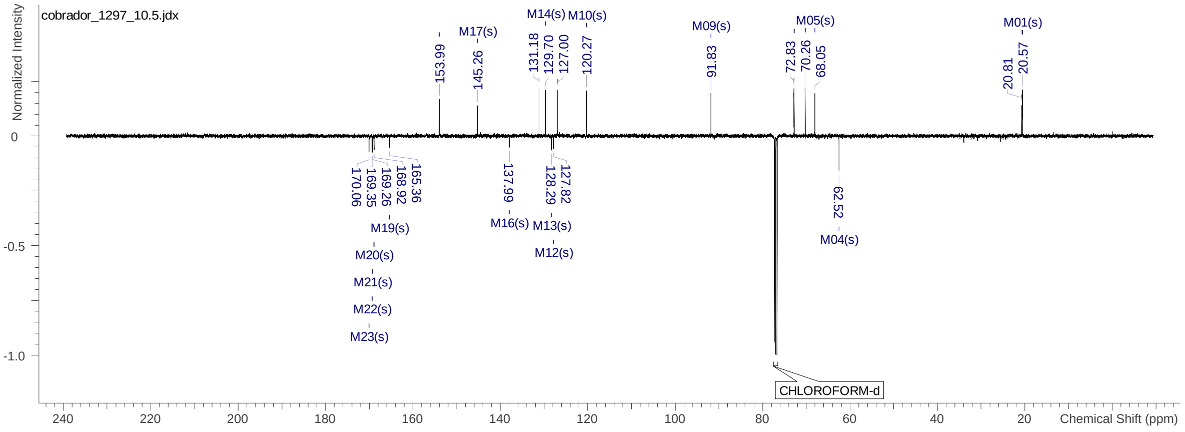
Normalized Intensity
cobrador_1297_10.5.jdx
CHLOROFORM-d
153.99
145.26
131.18
129.70
127.00
120.27
91.83
72.83
70.26
68.05
20.81
20.57
170.06
169.35
169.26
168.92
165.36
137.99
128.29
127.82
62.52
M01(s)
M04(s)
M05(s)
M09(s)
M10(s)
M12(s)
M13(s)
M14(s)
M16(s)
M17(s)
M19(s)
M20(s)
M21(s)
M22(s)
M23(s)
Chemical Shift (ppm)

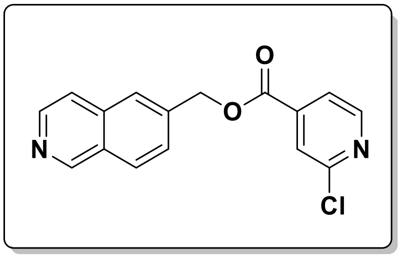
O
O
N
N
Cl

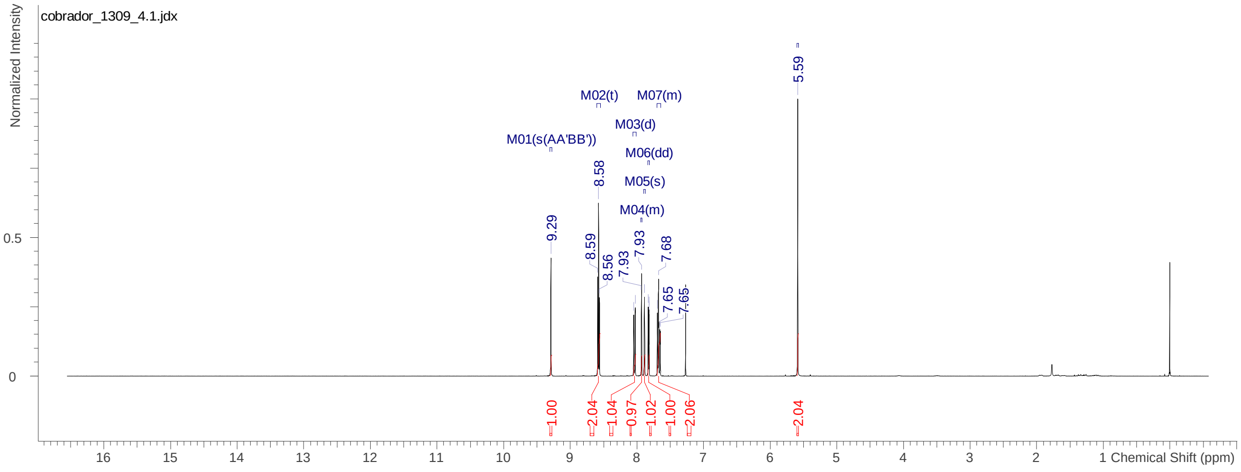
cobrador_1309_4.1.jdx
Normalized Intensity
Chemical Shift (ppm)
M01(s(AA'BB'))
M02(t)
M03(d)
M04(m)
M05(s)
M06(dd)
M07(m)
9.29
8.59
8.58
8.56
7.93
7.93
7.68
7.65
7.65
5.59
1.00
2.04
1.04
0.97
1.02
1.00
2.06
2.04

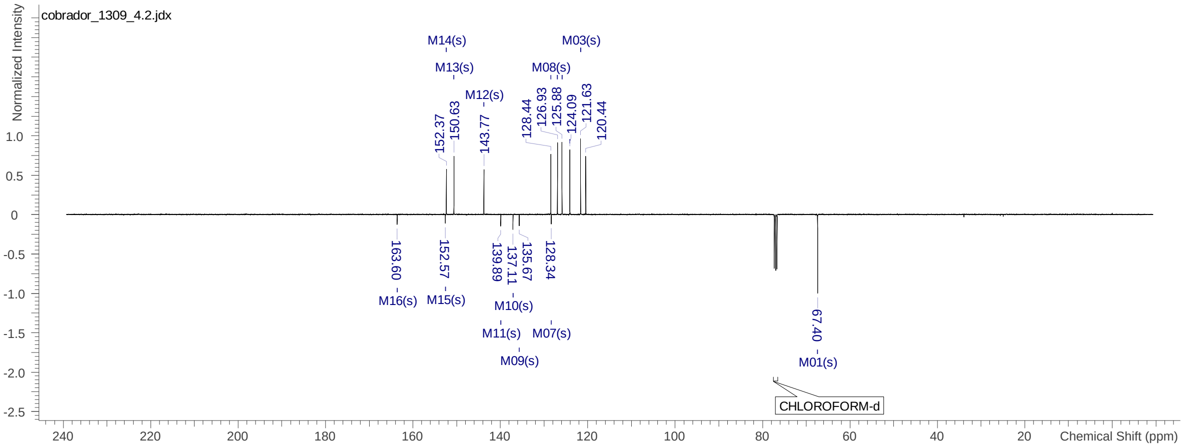
cobrador_1309_4.2.jdx
Normalized Intensity
Chemical Shift (ppm)
M14(s)
M13(s)
M12(s)
M08(s)
M03(s)
152.37
150.63
143.77
128.44
126.93
125.88
124.09
121.63
120.44
163.60
152.57
139.89
137.11
135.67
128.34
M16(s)
M15(s)
M10(s)
M11(s)
M07(s)
M09(s)
67.40
M01(s)
CHLOROFORM-d

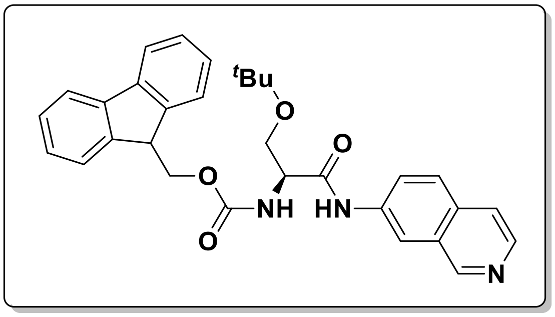
tBu
O
O
O
NH
HN
O
N

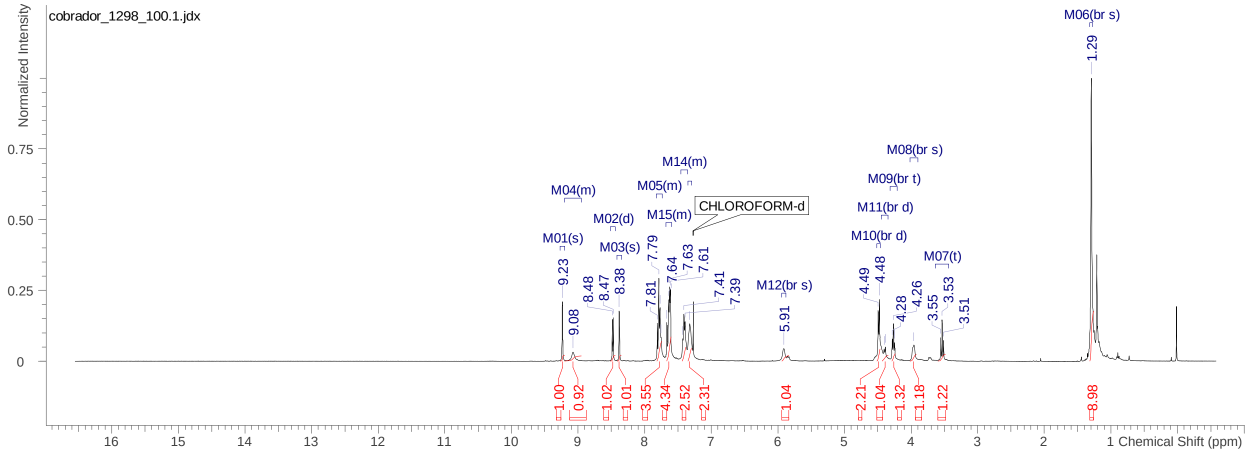
cobrador_1298_100.1.jdx
Normalized Intensity
M06(br s)
1.29
M08(br s)
M09(br t)
M11(br d)
M10(br d)
M07(t)
M14(m)
M05(m)
M15(m)
CHLOROFORM-d
M04(m)
M02(d)
M03(s)
M01(s)
M12(br s)
9.23
9.08
8.48
8.47
8.38
7.81
7.79
7.64
7.63
7.61
7.41
7.39
5.91
4.49
4.48
4.28
4.26
3.55
3.53
3.51
1.00
0.92
1.02
1.01
3.55
4.34
2.52
2.31
1.04
2.21
1.04
1.32
1.18
1.22
8.98
0.75
0.50
0.25
0
16
15
14
13
12
11
10
9
8
7
6
5
4
3
2
1 Chemical Shift (ppm)

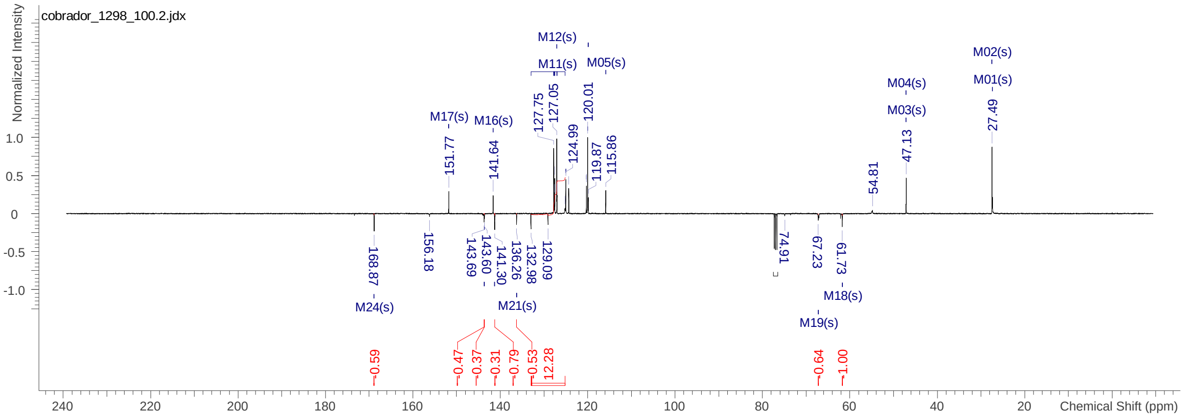
cobrador_1298_100.2.jdx
Normalized Intensity
M12(s)
M11(s)
M05(s)
M17(s)
M16(s)
M04(s)
M03(s)
M02(s)
M01(s)
151.77
141.64
127.75
127.05
124.99
120.01
119.87
115.86
54.81
47.13
27.49
168.87
156.18
143.69
143.60
141.30
136.26
132.98
129.09
74.91
67.23
61.73
M24(s)
M21(s)
M18(s)
M19(s)
-0.59
-0.47
-0.37
-0.31
-0.79
-0.53
12.28
-0.64
-1.00
1.0
0.5
0
-0.5
-1.0
240
220
200
180
160
140
120
100
80
60
40
20
Chemical Shift (ppm)

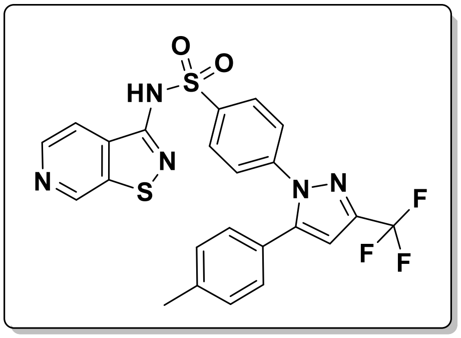

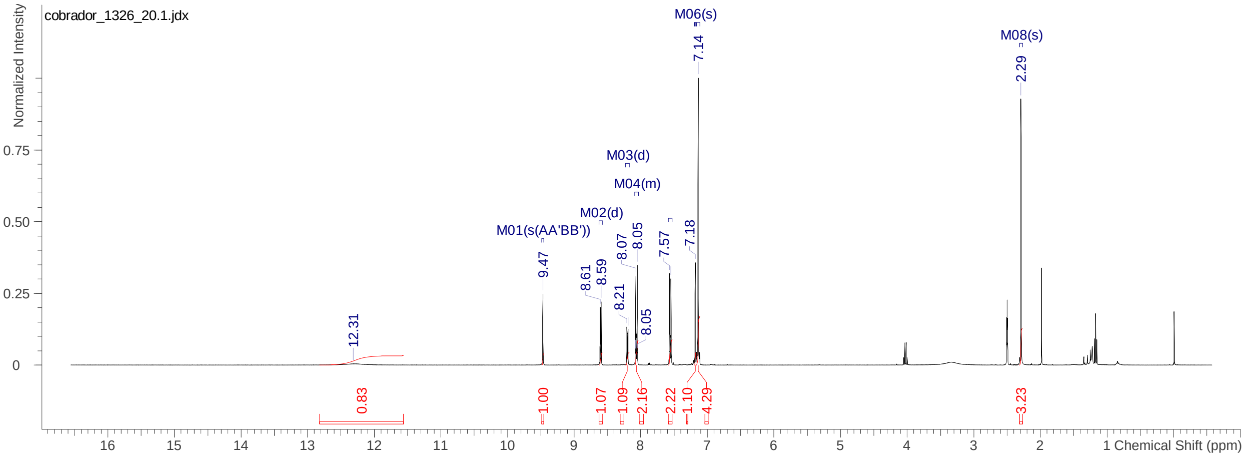
cobrador_1326_20.1.jdx
Normalized Intensity
Chemical Shift (ppm)
M01(s(AA'BB'))
M02(d)
M03(d)
M04(m)
M06(s)
M08(s)
12.31
9.47
8.61
8.59
8.21
8.07
8.05
8.05
7.57
7.18
7.14
2.29
0.83
1.00
1.07
1.09
2.16
2.22
1.10
4.29
3.23

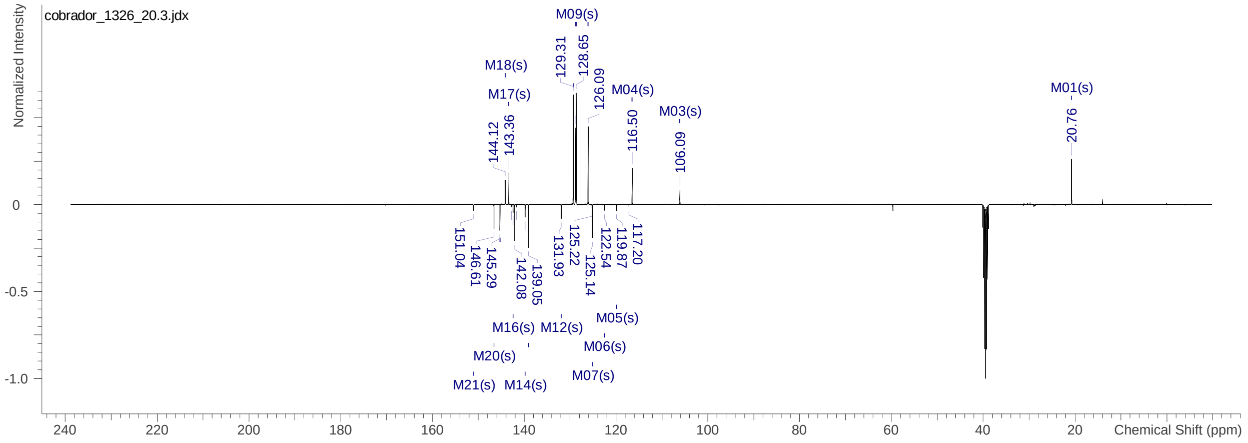
cobrador_1326_20.3.jdx
Normalized Intensity
Chemical Shift (ppm)
M09(s)
M18(s)
M17(s)
M04(s)
M03(s)
M01(s)
129.31
128.65
126.09
144.12
143.36
116.50
106.09
20.76
151.04
146.61
145.29
142.08
139.05
131.93
125.22
125.14
122.54
119.87
117.20
M16(s)
M12(s)
M05(s)
M20(s)
M06(s)
M07(s)
M21(s)
M14(s)

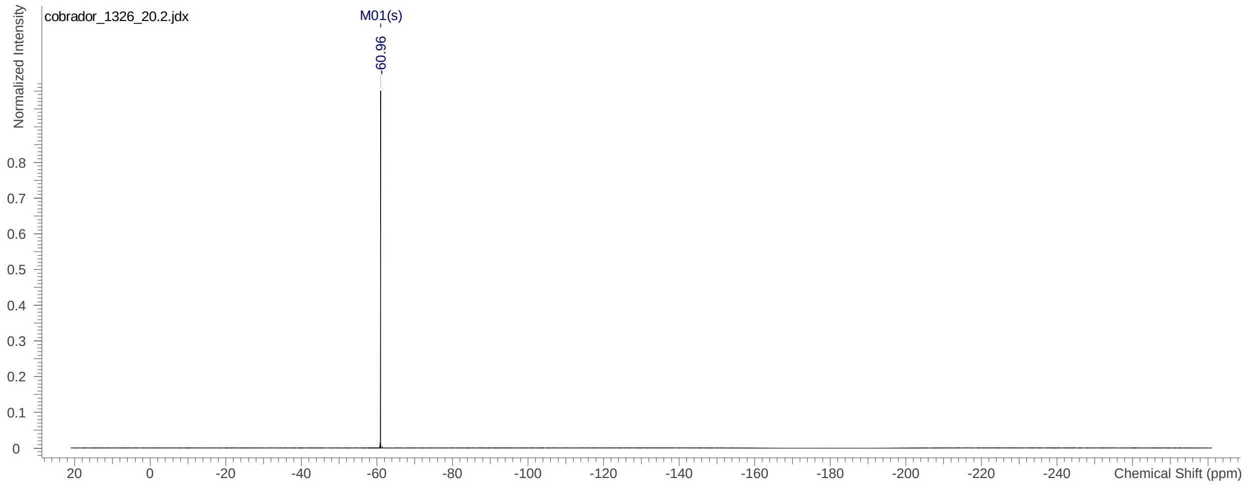
cobrador_1326_20.2.jdx
M01(s)
-60.96
Normalized Intensity
0.8
0.7
0.6
0.5
0.4
0.3
0.2
0.1
0
20
0
-20
-40
-60
-80
-100
-120
-140
-160
-180
-200
-220
-240
Chemical Shift (ppm)
